# NatF Contributes to an Evolutionary Shift in Protein N-Terminal Acetylation and Is Important for Normal Chromosome Segregation

**DOI:** 10.1371/journal.pgen.1002169

**Published:** 2011-07-07

**Authors:** Petra Van Damme, Kristine Hole, Ana Pimenta-Marques, Kenny Helsens, Joël Vandekerckhove, Rui G. Martinho, Kris Gevaert, Thomas Arnesen

**Affiliations:** 1 Department of Medical Protein Research, Ghent University, Ghent, Belgium; 2 Department of Biochemistry, Ghent University, Ghent, Belgium; 3 Department of Molecular Biology, University of Bergen, Bergen, Norway; 4 Department of Surgical Sciences, University of Bergen, Bergen, Norway; 5 Instituto Gulbenkian de Ciência, Oeiras, Portugal; 6 Department of Surgery, Haukeland University Hospital, Bergen, Norway; Stanford University School of Medicine, United States of America

## Abstract

N-terminal acetylation (N-Ac) is a highly abundant eukaryotic protein modification. Proteomics revealed a significant increase in the occurrence of N-Ac from lower to higher eukaryotes, but evidence explaining the underlying molecular mechanism(s) is currently lacking. We first analysed protein N-termini and their acetylation degrees, suggesting that evolution of substrates is not a major cause for the evolutionary shift in N-Ac. Further, we investigated the presence of putative N-terminal acetyltransferases (NATs) in higher eukaryotes. The purified recombinant human and *Drosophila* homologues of a novel NAT candidate was subjected to *in vitro* peptide library acetylation assays. This provided evidence for its NAT activity targeting Met-Lys- and other Met-starting protein N-termini, and the enzyme was termed Naa60p and its activity NatF. Its *in vivo* activity was investigated by ectopically expressing human Naa60p in yeast followed by N-terminal COFRADIC analyses. hNaa60p acetylated distinct Met-starting yeast protein N-termini and increased general acetylation levels, thereby altering yeast *in vivo* acetylation patterns towards those of higher eukaryotes. Further, its activity in human cells was verified by overexpression and knockdown of hNAA60 followed by N-terminal COFRADIC. NatF's cellular impact was demonstrated in *Drosophila* cells where NAA60 knockdown induced chromosomal segregation defects. In summary, our study revealed a novel major protein modifier contributing to the evolution of N-Ac, redundancy among NATs, and an essential regulator of normal chromosome segregation. With the characterization of NatF, the co-translational N-Ac machinery appears complete since all the major substrate groups in eukaryotes are accounted for.

## Author Summary

Small chemical groups are commonly attached to proteins in order to control their activity, localization, and stability. An abundant protein modification is N-terminal acetylation, in which an N-terminal acetyltransferase (NAT) catalyzes the transfer of an acetyl group to the very N-terminal amino acid of the protein. When going from lower to higher eukaryotes there is a significant increase in the occurrence of N-terminal acetylation. We demonstrate here that this is partly because higher eukaryotes uniquely express NatF, an enzyme capable of acetylating a large group of protein N-termini including those previously found to display an increased N-acetylation potential in higher eukaryotes. Thus, the current study has possibly identified the last major component of the eukaryotic machinery responsible for co-translational N-acetylation of proteins. All eukaryotic proteins start with methionine, which is co-translationally cleaved when the second amino acid is small. Thereafter, NatA may acetylate these newly exposed N-termini. Interestingly, NatF also has the potential to act on these types of N-termini where the methionine was not cleaved. At the cellular level, we further found that NatF is essential for normal chromosome segregation during cell division.

## Introduction

N-terminal acetylation (N-Ac) is a common modification of proteins, but its general role has remained rather enigmatic. For specific proteins, N-Ac is recognized as an important regulator of function and localization [1]–[4]. Recently, it was suggested that it may act as a general destabilization signal for some yeast proteins, [5] while other reports imply that it might serve as a stabilizer, for instance by blocking N-terminal ubiquitination mediated degradation [6]. N-Ac in eukaryotes mainly occurs co-translationally when 25–50 amino acids protrude from the ribosome, by the action of ribosome associated N-terminal acetyltransferases (NATs) [7]–[12]. N-Ac may occur on the initiator Met (iMet) or on the first residue after iMet excision by methionine aminopeptidases (MAPs) [13], [14]. Three major NAT complexes conserved from yeast to humans are thought to be responsible for the majority of N-terminal acetylation events: NatA, NatB and NatC [15]. Each complex is composed of specific catalytic and auxiliary subunits. NatA, the first NAT defined by Sternglanz and co-workers [16], potentially acetylates Ser-, Ala-, Thr-, Val-, Gly-, and Cys- N-termini after iMet-cleavage [17]–[19]. NatB and NatC potentially acetylate Met- N-termini when the second residue is either acidic or hydrophobic respectively [19]–[21]. In yeast, NatD was described to acetylate the Ser- N-termini of histones 2A and 4 *in vitro* and *in vivo* [22], while no such activity has yet been presented for higher eukaryotes. NatE is another NAT of which the *in vitro* activity was described for the human hNaa50p towards some Met-Leu- N-termini [23], but direct evidence of *in vivo* activity is still lacking. Thus, each hitherto *in vivo* characterized NAT appears to acetylate a distinct subset of substrates defined by the very first N-terminal amino acids. Phenotypes induced by loss or reduction of NATs suggest that these enzymes, and thus probably N-Ac, are implicated in a number of cellular processes. In higher eukaryotes, depletion of NatA, NatB or NatC is associated with cell cycle arrest or apoptosis [20], [21], [24]–[28] while sister chromatid cohesion defects are observed upon NatE depletion [29]–[31].

N-Ac occurs on more than 50% and 80% of cytosolic yeast and human proteins, respectively [18]. The reason for the major difference in occurrence of N-Ac between yeast and humans to date is not known. Furthermore, the fact that specific subsets of protein N-termini, like those initiated by Met-Lys-, are often acetylated in humans and fruit fly while rarely being acetylated in yeast, is also an unsolved issue [18], [32]. Further, such substrates do not match the predicted substrate specificity of any of the known NATs. Potential explanations for this evolutionary shift from lower to higher eukaryotes include: i) evolution towards more acetylation-prone N-termini in higher eukaryotes, ii) a shift in the substrate specificity between species-specific NATs, iii) the presence of novel, yet uncharacterized NATs in higher eukaryotes, and iv) the presence of species-specific co-factors or chaperones such as HYPK [33]. However, so far, no evidence for any of these hypotheses was presented.

In the current investigation, we sought to elucidate the mechanistic explanations for the evolutionary shift in N-terminal acetylation from lower to higher eukaryotes. To this end we investigated the potential evolution of acetylation prone N-termini, but found this to be a trivial contributing factor. We further explored the presence of novel NATs in higher eukaryotes as a possible explanation. *In silico* analysis revealed the existence of an uncharacterized human protein with a significant sequence similarity to known catalytic NAT subunits. Indeed, multiple lines of *in vitro* and *in vivo* evidence clearly demonstrate that this candidate protein conserved among animals is a major NAT displaying distinct substrate specificity, denoted Naa60p (NatF). Our data collectively suggest that Naa60p contributes to the increased occurrence of N-terminal acetylation in higher versus lower eukaryotes, and additionally revealed a novel regulator of chromosome segregation.

## Results

### Analyses of yeast and human N-termini reveal deviations of the residue contact order but provide no evidence for a significant evolution to more acetylation-prone N-termini in higher eukaryotes

**Figure 1:**
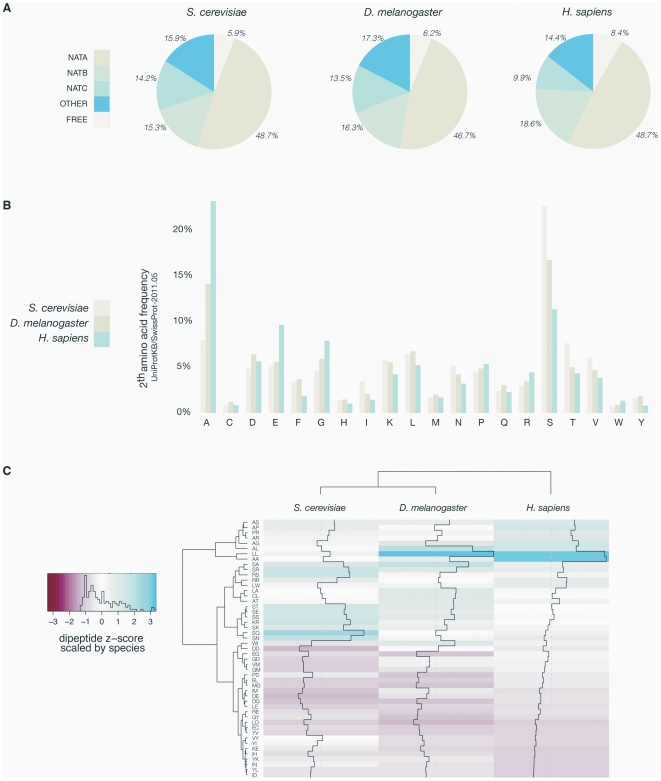
Overview of yeast, fruit fly, and human N-termini in NAT-classes and amino acid prevalence. A. Comparative analyses of the distribution of all methionine-starting yeast (6561), fruit fly (3120) and human SwissProt entries (20238) (SwissProt version 2011-05) according to their Nat-type. For simplicity, methionine processing was assumed to occur for (M)A-, (M)S-, (M)T-, (M)V-, (M)C-, (M)G- and (M)P- starting N-termini, while the X-P- rule was used to assign unacetylated database entries [32]. B. Bar charts of the amino acid occurrence at position 2 of yeast, fruit fly and human SwissProt protein entries. C. Heatmap of the ten highest and lowest ranking dipeptide z-scores across *H. sapiens*, *D. melanogaster* and *S. cerevisiae*. Z-scores are scaled by species, with the highest and lowest ranking z-score colored with the same intensity in blue and red respectively. The species (X-axis) and the dipeptides (Y-axis) were grouped by hierarchical clustering using the euclidian distance matrix of the z-scores.

We first investigated whether an evolution towards more acetylation-prone N-termini in higher eukaryotes could help explain the higher acetylation levels observed. Upon comparing the yeast, fruit fly and human proteomes, it is evident that the general distribution of N-termini is largely unaltered between the different classes, ‘NatA’, ‘NatB’, ‘NatC’ and ‘Other’ (Figure 1A). However, when considering all different subgroups based on the first two N-terminal amino acids, some significant alterations (p<0.01) appeared. Besides the general difference of the amino acid usage in yeast versus human N-termini in agreement with recent observations [34], the occurrence of (Met-)Ala- N-termini increased from 8% in yeast to 23% in humans, while Met-Glu- N-termini increased from 5% to 10%. On the other hand, (Met-)Ser- N-termini have decreased in occurrence from 23% in yeast to 11% in humans (Figure 1B). Interestingly, for these major trends, the occurrences in fruit-fly are intermediate between yeast and humans, indicating that these might be characteristic of the evolution to multicellular and more complex organisms. The next question is thus whether these changes in N-terminal sequences are causing a shift in N-Ac. In the current work, we performed COFRADIC-based N-terminal acetylation analyses of yeast and HeLa proteomes and present datasets covering 868 and 1,497 unique yeast and human N-termini, respectively (Tables S1 and S2). An overview of the occurrence of N-Ac of the different classes of assigned N-termini in the yeast (n = 648) and human (n = 1345) control samples is presented in Table 1. When relating the occurrence of N-Ac in yeast to the distribution of human N-termini and *vice versa* (based on the first two amino acids of the identified N-termini), we found no overall significant changes in N-Ac levels (Table S3). Thus, alteration in usage of the first two N-terminal amino acids, which are the major determinants for N-Ac, is not a significant cause for the observed shift from lower to higher eukaryotes.

**Table 1:** Overview of N-terminal acetylation of yeast and human proteins.*

	hNat			yNat		
	No.	completely, %	completely and partially, %	No.	completely, %	completely and partially, %
**NatA substrates**						
Ala-	495	91.6	95.3	52	25.0	48.1
Cys-	4	75.0	75.0	2	0.0	0.0
Gly-	10	0.0	0.0	12	0.0	8.3
Ser-	188	95.4	98.0	186	89.2	97.3
Thr-	39	75.9	89.7	36	19.4	55.6
Val-	39	3.2	19.3	31	0.0	9.7
**NatB substrates**						
Met-Asp-	93	95.7	98.9	59	93.2	100.0
Met-Glu-	165	97.6	100.0	35	94.3	100.0
Met-Asn-	32	100.0	100.0	46	89.1	100.0
**NatC substrates**						
Met-Ile	10	30.0	50.0	14	26.7	33.3
Met-Leu-	32	56.3	75.0	30	26.7	33.3
Met-Phe-	18	83.3	83.3	10	60.0	60.0
**Other**						
Asp-	1	100.0	100.0			
Ile-	1	0.0	0.0	1	0.0	0.0
Glu-	1	100.0	100.0			
Pro-	68	0.0	0.0	25	0.0	0.0
Met-Ala	14	64.3	92.9	1	0.0	100.0
Met-Cys-	1	100.0	100.0			
Met-Gly-	3	33.3	100.0	3	0.0	100.0
Met-Lys-	46	13.0	47.8	46	4.3	13.0
Met-Met-	12	83.3	100.0	6	16.7	83.3
Met-Pro-	6	0.0	0.0			
Met-Gln-	16	81.3	93.8	13	30.8	84.6
Met-Ser-	9	88.9	88.9	14	7.1	64.3
Met-Thr-	18	83.3	94.4	7	0.0	28.6
Met-Val-	20	50.0	85.0	11	0.0	45.5
Met-Tyr-	4	75.0	100.0	8	25.0	62.5
**Total**	**1345**	**79.1**	**85.2**	**648**	**52.5**	**67.9**

Quantitative COFRADIC-based analysis of N-terminal acetylation in yeast (*S. cerevisiae*) and HeLa proteomes determined the acetylation status of 648 and 1345 unique N-termini in the two species, respectively. Overall, 67.9% and 85.2% of the yeast and HeLa proteomes, respectively, are N-terminally acetylated (fully or partially). The analysed proteins are categorized based on their N-terminal sequences (substrate classes).

*Only N-termini of which the degree of N-Ac could be univocally calculated/determined in the control yeast (648) and control human (1345) setups were used for the overall calculation of N-Ac.

Since it was shown that amino acid usage at protein N-termini differs significantly from what is expected [34], and differences in dipeptide composition have been used to predict protein expression levels [35], thermostability [36] and subcellular localization [37], we further characterized the residue contact order at protein N-terminal parts by studying dipeptide frequencies in the theoretical proteomes of *Homo sapiens*, *Drosophila melanogaster* and *Saccharomyces cerevisiae* (UniProt/SwissProt entries (version 2011-05)). Therefore, the occurrence of the 400 possible dipeptides from the 20 amino acids in all proteins was estimated for randomly selected human dipeptides and N-terminal (amino acids 2–11) dipeptides by Monte-Carlo sampling. Further, a z-score was applied to correct for differences in database size. Contacting residues in a random, non-N-terminal set correlate well with the expected theoretical contact order (data not shown). In sharp contrast, the overall dipeptide composition deviates significantly for database-annotated N-termini. A heatmap visualization centered and scaled by species mean and standard deviation for *Homo sapiens*, *Drosophila melanogaster* and *Saccharomyces cerevisiae* is shown for the ten dipeptides with the highest and lowest z-scores (union of n = 49) (Figure 1C). Overall, these data strengthen the observation that N-terminal sequences not only display altered patterns of amino acid frequencies but deviate extensively in their residue contact order in a species-specific manner, which might additionally impose yet undetected constraints in determining N-Ac.

When considering each type of N-terminus, it is evident that several of these are more acetylated in humans while some are mainly unchanged, but none are less N-Ac. The major groups of protein N-termini with an increase in N-Ac in humans as compared to yeast include (Met-)Ala-, (Met-)Val-, and Met-Lys- N-termini and thus represent major contributors to the overall evolutionary shift (Table 1 and Table S3).

Another potential cause for the evolution towards the higher level of N-Ac is a shift in the substrate specificity between species-specific NATs. For NatA, which is responsible for N-Ac of two of the important N-terminal types mentioned above, (Met-)Ala- and (Met-)Val- this seemed not to be the case as both human NatA and yeast NatA acetylated the very same subset of N-termini in yeast [18]. For the final group, Met-Lys- N-termini, no information is available since such N-termini have not been linked to any of the NAT classes previously characterized.

### Naa60p is a novel NAT displaying a unique substrate specificity *in vitro*

**Figure 2:**
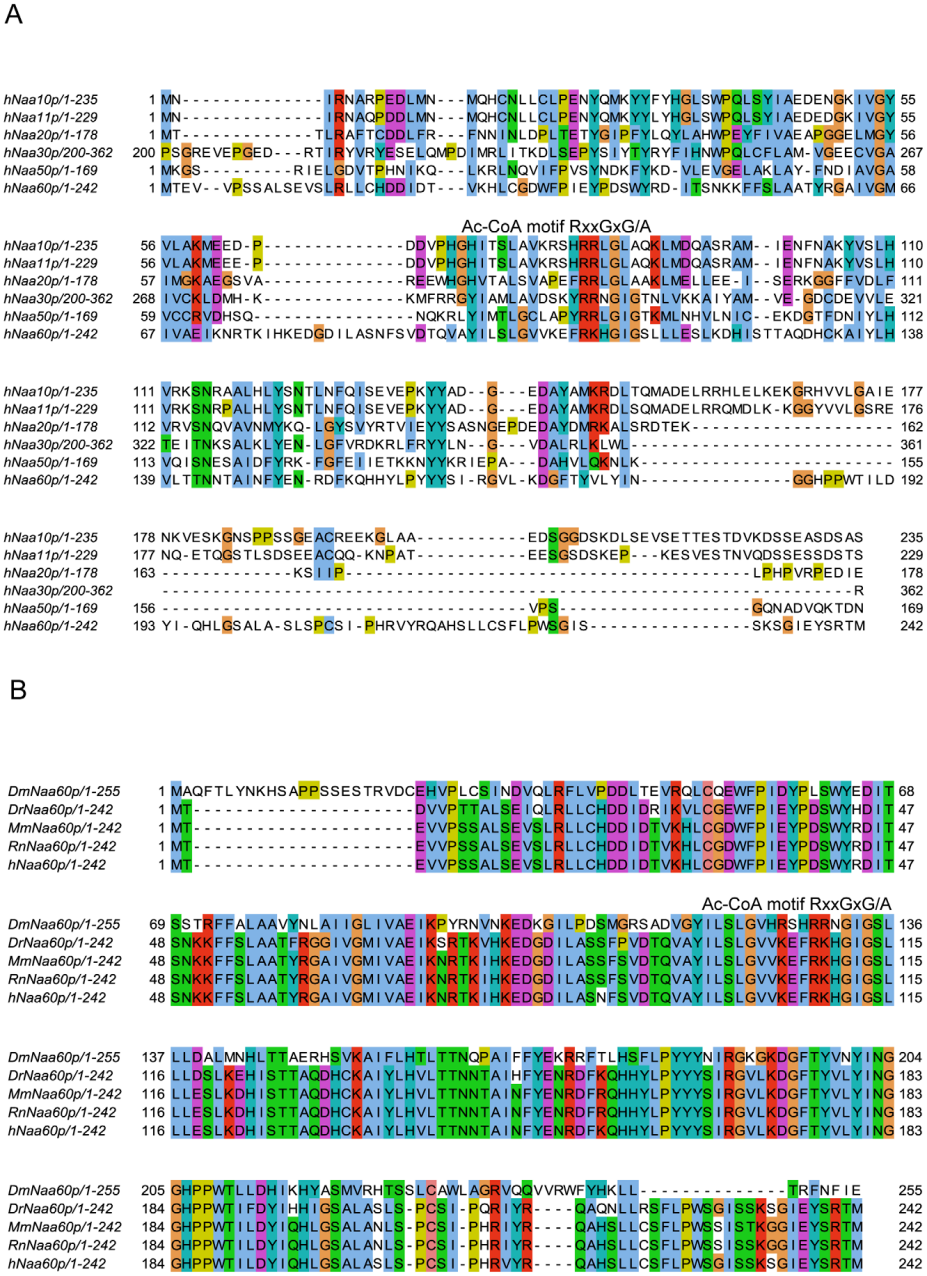
Amino acid sequence alignments of hNaa60p and other NATs. A. Amino acid sequence alignment of NAT15/hNaa60p and known human NATs. Only amino acid 200–362 of hNaa30p was included in the alignment. The consensus Acetyl Coenzyme A (AcCoA) binding motif RxxGxG/A, where x can be any amino acid, is indicated. T-Coffee (http://www.ebi.ac.uk/Tools/t-coffee/index.html) was used to make the alignment. Purple background indicates acidic residues, red indicates basic residues, orange indicates glycine, yellow indicates proline, blue indicates hydrophobic residues, green indicates polar residues, and turquoise indicates histidine and tyrosine. B. Amino acid sequence alignment of Naa60p from *Drosophila melanogaster* (Dm), *Danio rerio* (Dr), *Mus musculus* (Mm), *Rattus norvegicus* (Rn) and *Homo sapiens* (Hs). The consensus Acetyl Coenzyme A (AcCoA) binding motif RxxGxG/A, where x can be any amino acid, is indicated. Colour codes are used as in A.

**Figure 3:**
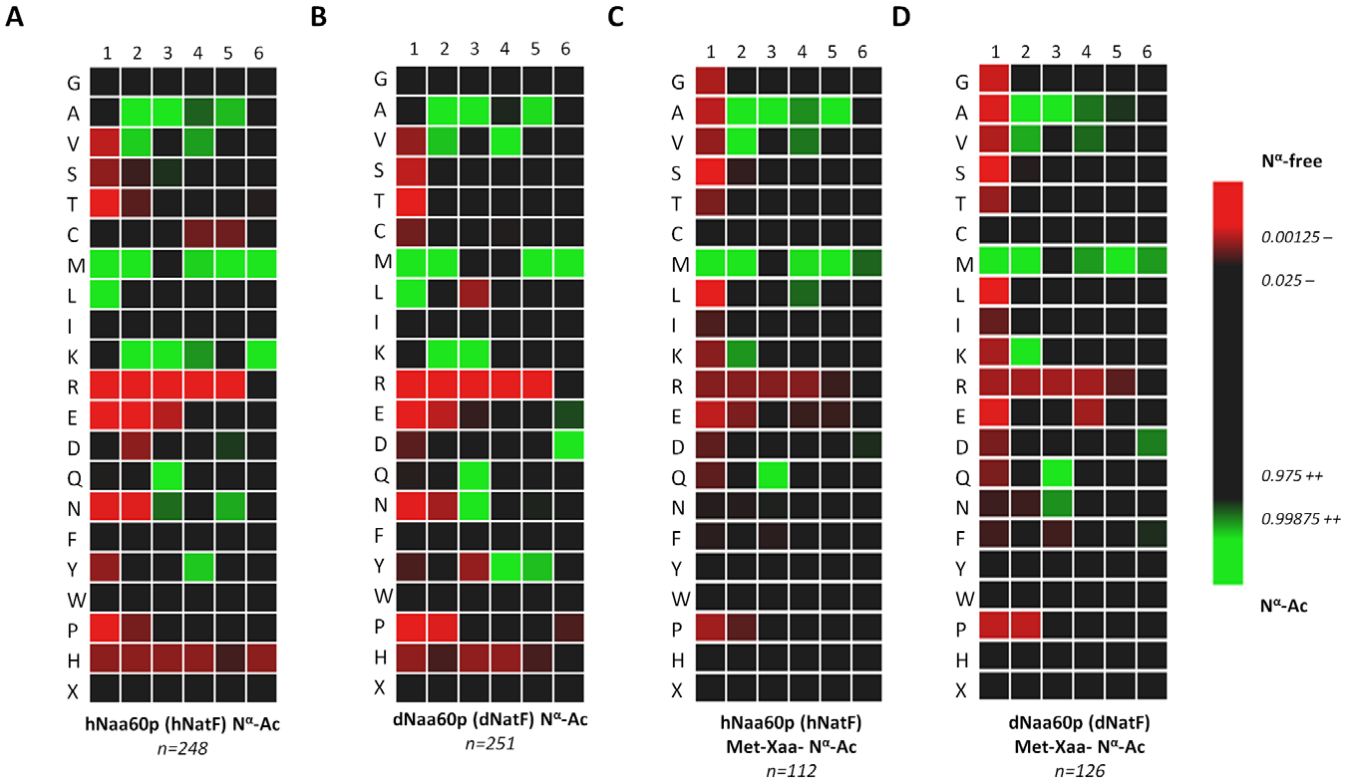
Heatmap visualization reflecting the *in vitro* substrate specificity of hNaa60p and dNaa60p. A. Heatmap of the 248 unique hNaa60p-specific oligopeptide substrates (353 substrate peptides in total). B. Heatmap of the 251 unique dNaa60p-specific oligopeptide substrates (345 substrate peptides in total). C. Heatmap of the subset of 112 unique hNaa60p-specific methionine-starting oligopeptide substrates. D. Heatmap of the subset of 126 unique dNaa60p-specific methionine-starting oligopeptide substrates. Data was normalized against the natural positional amino acid composition of SwissProt (version 57.8) [iterative rounds (n = 100) of randomly selected sequences (n = 100) were taken as to correct for the statistical variations (SD = standard deviation) intrinsically present at each position in the experimental datasets ranging from amino acid 1 to 6]. The significance threshold was set at 0.01. Red color shades are negatively correlated with the occurrence in Naa60p peptide-substrates as compared to random sequences in SwissProt, while green shades are positively correlated.

In search of novel human NATs, we used the sequences of known human NATs in NCBI BLAST queries (search set: Swiss-Prot database restricted to human proteins). We identified one protein with a significant similarity to several of the known NATs, namely NAT15/Q9H7X0/Naa60p (Figure 2). NAT15/Naa60p is highly conserved among animals (Figure 2B) and homologues are also potentially present in plants (for instance At5g16800). In order to assess whether NAT15 was an N-terminal acetyltransferase, the *NAT15* ORF was recombinantly expressed and purified from *Escherichia coli* and applied to a newly developed *in vitro* proteome-derived peptide library N-terminal acetylation assay [38]. In brief, natural proteomes are used to generate N^α^-free peptide substrate pools (libraries) by enrichment with strong cation exchange (SCX). When such a peptide library is incubated with a NAT enzyme, the newly N^α^-acetylated peptides are enriched by a second SCX fractionation step, resulting in a positive selection of NAT-specific peptide substrates. Subsequently, the NAT-oligopeptide substrates are identified by LC-MS/MS, and the *in vitro* substrate specificity profile of the NAT in question is analyzed using IceLogo [39], an analytical tool that uses probability theory to visualize significant conserved sequence patterns in multiple peptide sequence alignments by comparing against a chosen background (reference) sequence set. Using this proteome-derived peptide assay, NAT15 N^α^-acetylated numerous peptides *in vitro* and displayed a distinct substrate specificity profile (Figure 3A). Thus, according to the revised NAT-nomenclature system [15], we named this protein Naa60p and its activity NatF. Remarkably, the preferred N-termini included Met-Lys-, Met-Ala-, Met-Val-, and Met-Met-, categories for which there are currently no known N-terminal acetyltransferase(s). Of particular interest, recent data revealed that several Met-Lys- N-termini were acetylated in humans and fruit fly while no such N-Ac events of Met-Lys- N-termini were found in yeast, pointing to the presence of (a) NAT(s) specific for higher eukaryotes or an altered specificity profile of (a) higher eukaryotic NAT(s) as compared to yeast NAT(s) [18], [32]. To expand these observations to higher eukaryotes in general, we purified the predicted fruit fly homologue dNaa60p (CG18177) and confirmed this protein to be a NAT with a nearly indistinguishable specificity profile as compared to hNaa60p (Figure 3B). As deduced from the *in vitro* specificity profile, besides Met-; Leu- was also preferred at the first position, which, as we described previously [38], is expected since both Met and Leu share similar physiochemical characteristics [40], [41]. However, for co-translational N^α^-acetylation, Leu at the first position appears physiologically irrelevant as it is not expected as the first amino acid, since when it follows the initiator methionine, its size precludes the removal of this initiator methionine by MAPs [14]. When only including Met residues at the first position, the specificity profile remains largely unchanged (Figure 3C, 3D and Figure S1). Given its *in vitro* specificity, we considered Naa60p a qualified candidate for the Met-Lys- acetylation activities observed in higher eukaryotes.

### Naa60p is a NAT *in vivo*, and ectopic expression in yeast shifts the global N-Ac patterns

**Figure 4:**
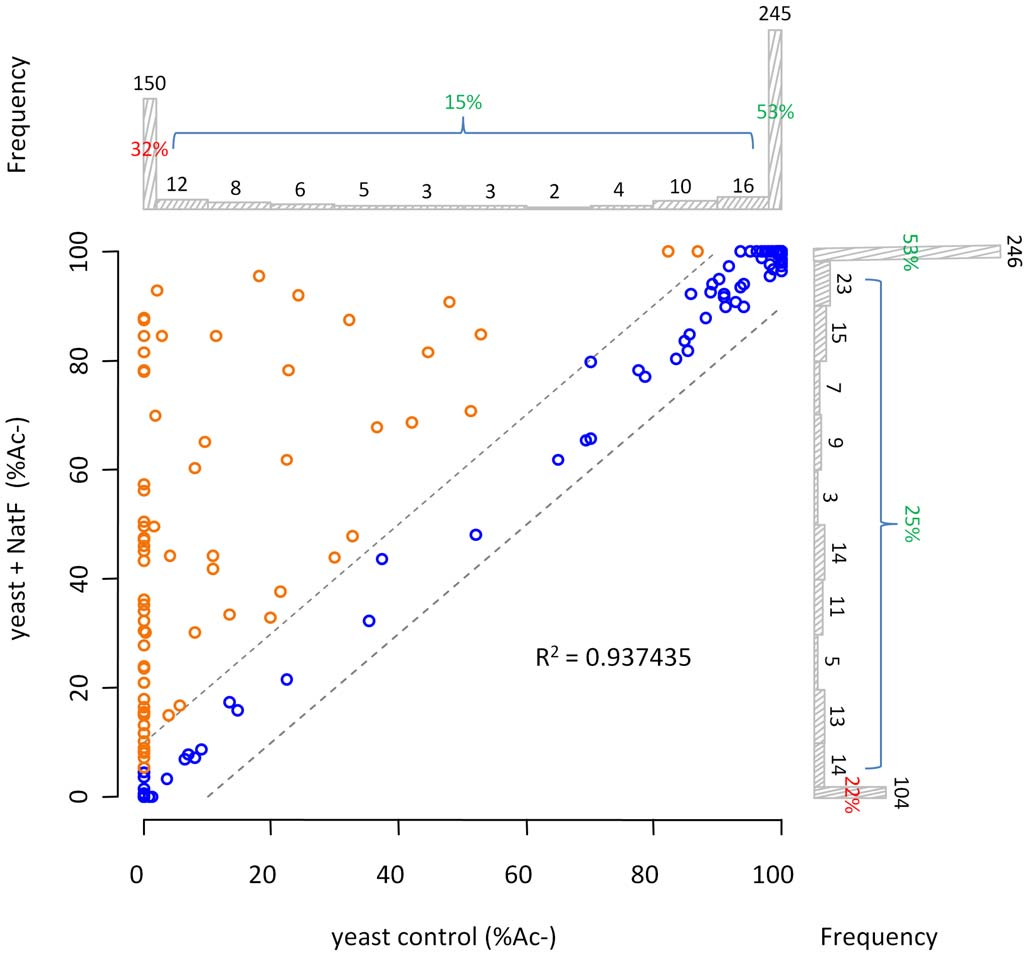
NatF N-terminally acetylates yeast substrates *in vivo*. Scatterplot displaying the correlation of the degrees of N^α^-acetylation when comparing a control (X-axis) and a human NatF (hNaa60p)-expressing (Y-axis) yeast N-terminome dataset. The correlation was calculated with the R statistical package to be R^2^ = 0.937. The N-termini displaying a significant variation in the degree of N^α^-acetylation (see Materials and Methods) are highlighted in orange. The frequency histograms of the number of matching data points are also shown.

**Figure 5:**
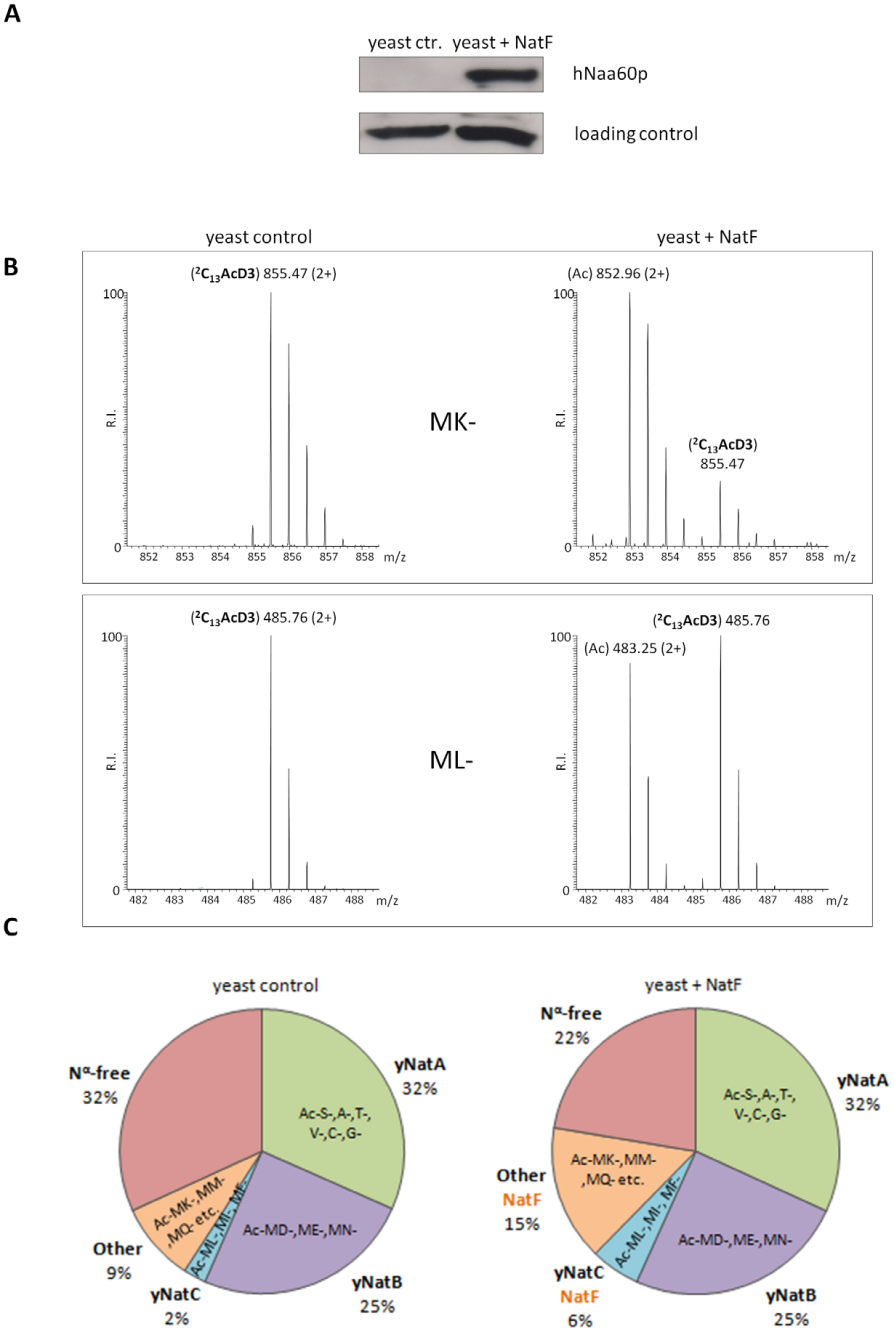
NatF expression shifts the overall status of the yeast N-terminal acetylome. A. A yeast strain expressing hNaa60p/NatF ‘Yeast+NatF’ was generated (see Materials and Methods), and processed by SDS-PAGE and Western blotting along with the control strain ‘Yeast ctr.’ containing an empty control plasmid. Anti-hNaa60p verified expression of hNaa60p in the Yeast+NatF strain (an unspecific band served as loading control). B. MS-spectra from the MK- starting N-terminal peptide (doubly charged precursor) of the Smr domain-containing protein YPL199C (^1^MKGTGGVVVGTQNPVR^16^) reveals two distinguishable isotopic envelopes in the hNaa60p expressing yeast strain [i.e. the acetylated (Ac) and ^2^C_13_ and trideutero-acetylated forms (^2^C_13_AcD3), right upper panel] indicative for the fact that this N-terminus is 82% *in vivo* N^α^-acetylated while being N^α^-free in the control sample (left upper panel). The lower panels show MS-spectra of the ML-starting N-terminal peptide (doubly charged precursor) of the uncharacterized protein YGR130C (^1^MLFNINR^7^) in the control sample (0% N^α^-acetylated, left lower panel) or hNAA60 sample (48% acetylated, right lower panel). C. Nat-category specific distribution of experimentally identified yeast N-termini in the yeast control or hNaa60p-expressing yeast strain. Only those N-termini of which the N-Ac status could univocally be assigned (n = 464) were considered.

In order to assess whether hNaa60p represents a NAT *in vivo* and to address its potential role in the evolutionary N-Ac shift, we generated a yeast strain expressing hNaa60p. We were not able to observe any differences in growth rates or plating efficiencies between yeast control strains and yeast strains expressing hNaa60p (data not shown). Since yeast does not have an obvious homolog of hNaa60p, ectopic expression was expected to reveal whether hNaa60p endows yeast with a greater acetylating potential. Indeed, when comparing N-terminal acetylation in the proteome of control yeast (yeast control) to the yeast expressing hNaa60p (yeast+NatF), significant alterations in the N^α^-acetylome were observed (Figure 4). For example the Smr domain-containing protein YPL199C and uncharacterized protein YGR130C, with respectively Met-Lys- and Met-Leu- N-termini, were unacetylated in control yeast while 82% and 48% acetylated in the strain expressing hNaa60p/NatF (Figure 5). In total, for 464 of the 544 (or 85%) unique N-termini identified in both proteomes, the N-acetylation status could univocally be determined. Of these, 72 N-termini were more acetylated in the hNaa60p expressing strain, while none were less acetylated, indicating that at least 16% of the identified yeast proteome was acetylated by hNaa60p (Figure 4 and Table S4). 44 of the 72 hNaa60p acetylated N-termini were completely unacetylated in control yeast, while 28 were partially acetylated. For the latter group, hNaa60p increased the degree of acetylation with at least 10%. It should be noted that this may represent an underestimation of hNaa60p's capacity since fully acetylated N-termini (53%) in the control strain may also represent targets, which would be masked by redundancy with the yeast NAT-machinery. The hNaa60p yeast substrates identified *in vivo* were in agreement with the *in vitro* determined substrate specificities. The most common *in vivo* substrate classes were Met-Lys- (n = 14), Met-Ser- (n = 9), Met-Val- (n = 8), Met-Leu- (n = 8), Met-Gln- (n = 6), Met-Ile- (n = 5), Met-Tyr- (n = 5), and Met-Thr- (n = 5) (Table 2).

**Table 2:** Types of N-termini acetylated by NatF using different methods.

*In vitro* peptide library assay specificity of recombinant hNaa60p(Figure 3 and Figure S1)	*In vivo* yeast substrates of ectopically expressed hNaa60p#(Figure 4, Figure 5, and Table S4)	HeLa substrates affected by knockdown or overexpression of hNaa60p#(Figure 6 and Table S5)	*In vitro* assay using synthetic peptides to verify selected substrates of recombinant hNaa60p(Figure 7)
Met-Lys-	Met-Lys-	Met-Lys-	positive
Met-Ala-	Met-Ala-	Met-Ala-	positive
Met-Val-	Met-Val-	Met-Val-	n.d.
Met-Met-	Met-Met-	Met-Met-	n.d.
	Met-Ser-	Met-Ser-	n.d.
	Met-Leu-	Met-Leu-	positive
	Met-Gln-	Met-Gln-	n.d.
	Met-Ile-		n.d.
	Met-Tyr-		n.d.
	Met-Thr-		n.d.
	Met-Phe-		n.d.
	Met-Gly-		n.d.
	Ser-(Glu-)		negative
	Ala-(Gly-)	Ala-(Ala-)	n.d.
		Thr-(Asp-)	n.d.

NatF/hNaa60p was shown to acetylate several types of N-terminal sequences by the different *in vitro* and *in vivo* methods applied in this study. This overview displays which N-termini are acetylated by NatF using the different methods. Met-Lys-, Met-Ala-, Met-Val- and Met-Met- are among the N-terminal sequences that appear to represent the preferred NatF substrates.

Underlined N-termini were most commonly represented (n>4).

# Underlined N-termini have at least two independent hits. N.d., not determined.

Among those acetylated by hNaa60p were proteins with Met-Lys- starting N-termini, which are of particular interest because these are acetylated in humans by an unknown NAT, while only rarely acetylated in yeast [18]. When considering the yeast control dataset, only 13% of the Met-Lys- N-termini are fully or partially acetylated, while the corresponding number for the yeast+NatF strain increases to 48%. In striking resemblance, 40% to 70% of Met-Lys- N-termini are N-terminally acetylated in human cell lines as respectively demonstrated previously [18] and in the current dataset (Table 1). Met-Leu-, Met-Ile-, and Met-Phe- starting N-termini, a class of N-termini considered NatC substrates, are other types of N-termini frequently found to be acetylated by hNaa60p. Finally, many substrate N-termini without a proper NAT-classification (including initiator Met-retaining N-termini of which the iMet is only partially removed) were acetylated: Met-Ser-, Met-Val-, Met-Thr- and Met-Met-, and Met-Gln-. Thus, hNaa60p acetylates both N-free besides partially acetylated protein N-termini in yeast, some without any known corresponding yeast NAT, as well as N-termini for which there is a putative NAT (NatC). This indicates that Naa60p may mediate a significant part of the shift in N-terminal acetylation from lower to higher eukaryotes. Furthermore, in contrast to the current opinion, this also strongly suggests redundancy in the N^α^-acetylation system, meaning that different NATs may have (partially) overlapping substrates. The effect of hNaa60p on overall N-terminal acetylation in yeast is shown in Figure 5C. Overall, the expression of hNaa60p increased the fraction of N^α^-acetylated yeast proteins from 68% to 78%, in particular affecting the groups ‘yNatC’ and ‘Other’ (Figure 4 and Figure 5).

### Overexpression or knockdown of h*NAA60* affects N-terminal acetylation in HeLa cells

**Figure 6:**
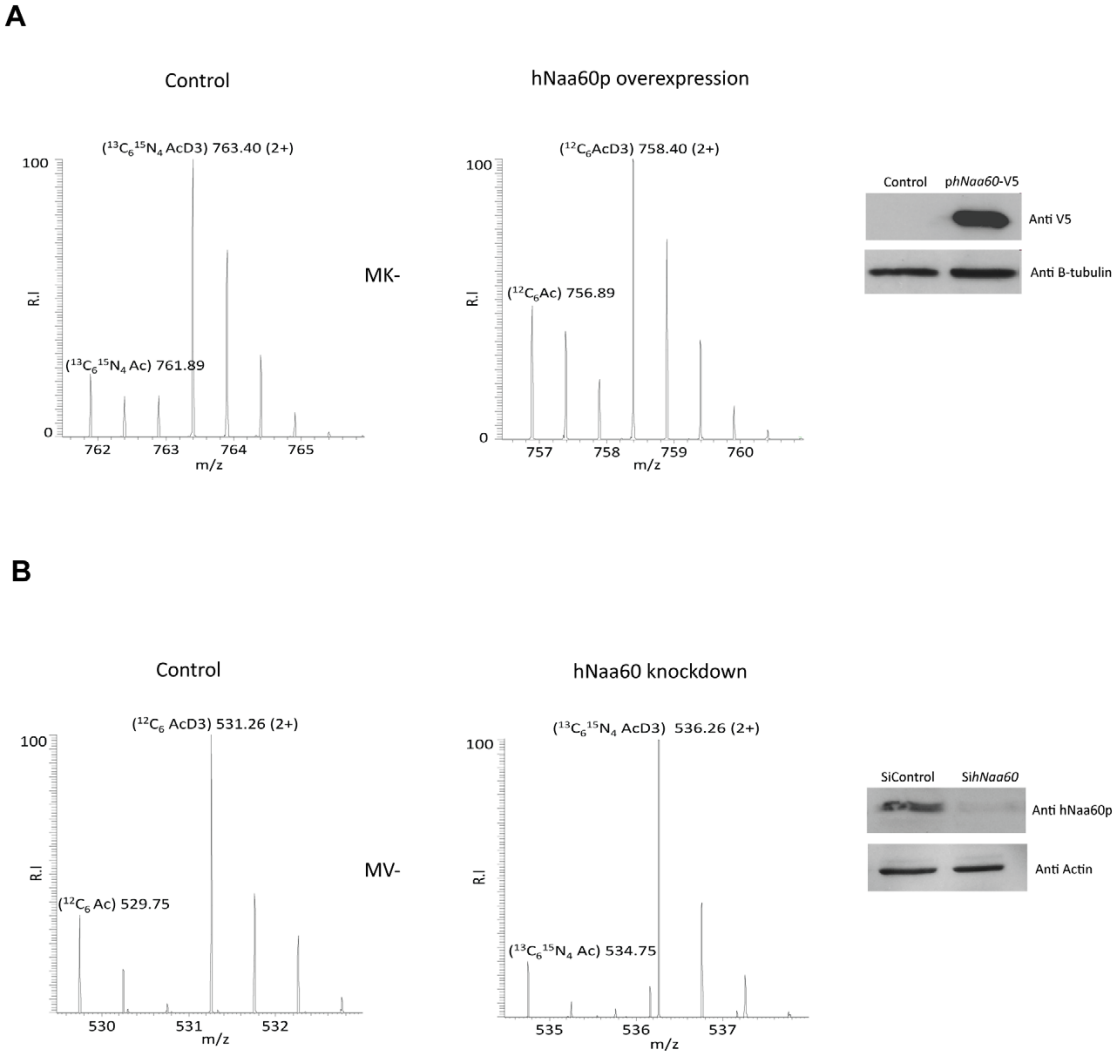
Knockdown and overexpression of hNaa60p affects N-terminal acetylation in HeLa cells. A. HeLa cells cultivated in ^13^C_6_^15^N_4_ L-arginine were transfected with control vector and cells cultivated in ^12^C_6_ L-arginine were transfected with ph*NAA60*-V5. After 48 hours of transfection the cells were harvested, lysed and subjected to COFRADIC and MS and MS/MS- analysis. MS spectra of the peptide ^1^MKGKEEKEGGAR^12^, originating from the STIP1 homology and U-box containing protein1 is shown. The protein is more acetylated when hNaa60p is overexpressed (32% N^α^-acetylated) as compared to the control (18% N^α^-acetylated). Aliquots were processed by SDS-PAGE and Western blotting using anti-V5 and anti-β-tubulin antibodies. B. Control cells cultivated in ^12^C_6_ L-arginine were transfected with 50 nM siNon-targeting control, and cells cultivated in ^13^C_6_^15^N_4_ L-arginine were transfected with 50 nM sih*NAA60* pool. After 84 hours of transfection the cells were harvested and subjected to COFRADIC and MS analysis. MS spectra of the peptide ^1^MVPGSEGPAR^10^, originating from the protein Mediator of RNA polymerase II transcription subunit 25 is shown. The peptide was partially acetylated in both control (26% N^α^-acetylated) and knockdown setup (17% N^α^-acetylated), however the peptide was less N^α^-acetylated when the levels of hNaa60p was reduced. Aliquots were processed for SDS-PAGE and Western blotting using anti-hNaa60p and anti-actin antibodies.

Overexpression or knockdown of h*NAA60* in HeLa cells was found to increase or decrease, respectively, the N-terminal acetylation of proteins matching the above defined *in vitro* and *in vivo* substrate specificity of hNaa60p (Table S5). Examples include the proteins STIP1 homology and U-box containing protein1 (^1^MKGKEEKEGGAR^12^) and mediator of RNA polymerase II transcription subunit 25 (^1^MVPGSEGPAR^10^) where the N^α^-acetylation status is shifted as a consequence of h*NAA60* overexpression (from 18% to 32% acetylation) or knockdown (from 26% to 17% acetylation), respectively (Figure 6). These data strongly point to the fact that hNaa60p in human cells can act on the classes of N-termini deduced from the *in vitro* and *in vivo* yeast analyses described above (Table 2). Obviously, overexpression analysis will be limited by the redundancy among NATs and by the fact that naturally hNaa60p-acetylated N-termini may be fully acetylated and as such do not appear as substrates for the overexpressed hNaa60p. Furthermore and in line with previous knockdown analyses of NatA in HeLa cells, the semi-effective nature of siRNA-mediated knockdown as well as the long time period needed for a clear effect on N-terminal acetylations to occur, make such analyses indicative rather that providing the full picture of acetylation events mediated via a specific NAT and as shown previously, primarily affects the least efficiently acetylated N-termini [18]. Thus, the real number of Naa60p substrates in human cells is likely to be significantly higher as compared to the substrates identified in these particular analyses.

**Figure 7:**
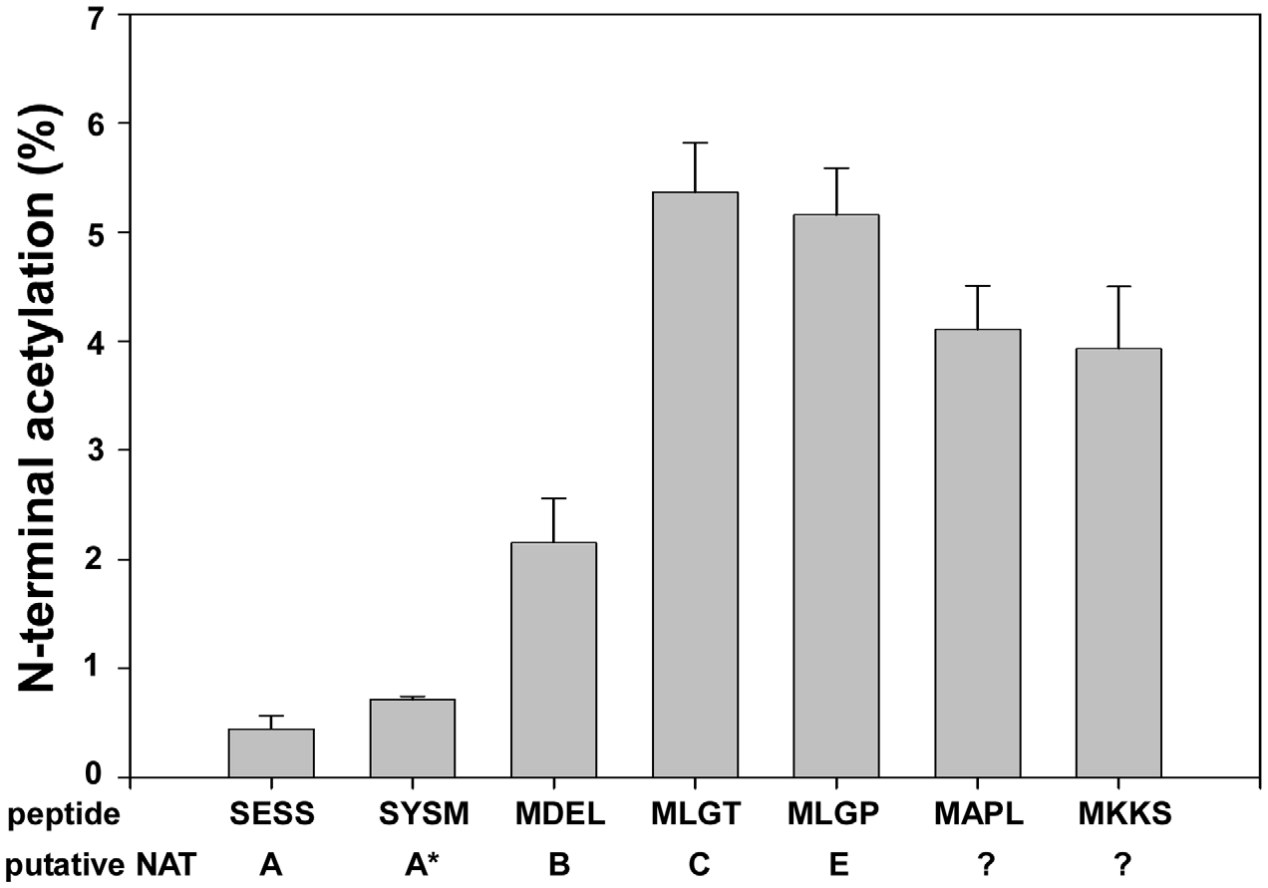
NAT-activity of recombinant hNaa60p towards synthetic N-terminal oligopeptides. MBP-hNaa60p was incubated with the indicated oligopeptide substrates (200 µM) and acetyl-Coenzyme A (300 µM) in acetylation buffer for 35 minutes at 37°C. Peptide acetylation was determined by RP-HPLC peptide separation. The NATs responsible for acetylating the different peptides are shown. Question marks indicate that no NAT has yet been identified to acetylate these peptides. *SYSM represents the ACTH N-terminus which is an artificial *in vitro* substrate of NatA. The four first amino acids in the oligopeptides are indicated, for further details see Materials and Methods.

Finally, two of the acetylated N-termini of the predicted NatF class picked up from the HeLa dataset (Table S2) were tested by a direct *in vitro* approach. Synthetic peptides derived from the Met-Lys- and Met-Ala- N-termini of Septin 9 and Protein phosphatase 6, respectively, were subjected to an *in vitro* acetylation assay with purified hNaa60p followed by an HPLC-based analysis of acetylated and unacetylated peptides. In agreement with the human and yeast *in vivo* data and *in vitro* substrate profiles obtained above, hNaa60p acetylated both these peptides, as well as representatives of NatC and NatE class substrates (Figure 7). Thus, we confirmed the N-terminal acetylation of human substrates as well as the potential redundancy with NatC and NatE enzyme classes (Table 2).

### dNaa60p is required for chromosome segregation during anaphase

**Figure 8:**
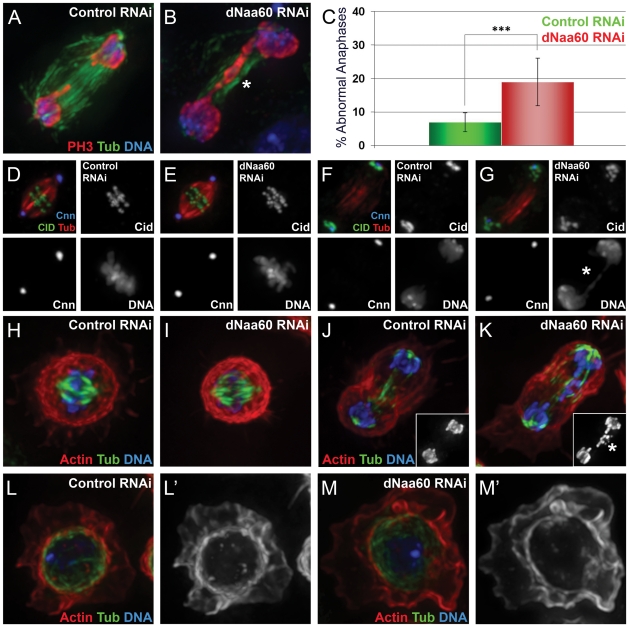
dNaa60p is required for chromosome segregation during anaphase. Control dsRNA treated cells (A,D,F,H,J and L). d*NAA60* dsRNA treated cells (B,E,G,I,K and M). d*NAA60*-depleted cells exhibited chromosome segregation defects during anaphase (A–C). These defects included lagging chromosomes (K, highlighted by asterisk) and chromosome bridges (B and G, highlighted by asterisk). Quantification of chromosome segregation defects in d*NAA60*-depleted cells (n = 278) and control cells (n = 179) (***p<0,001 Student's test) (C). Histone 3 phosphorylated on Serine 10 (red), α-tubulin (green) and DNA (blue). d*NAA60*-depleted cells showed no significant defects in the localization of both Cnn and Cid proteins (D–G). Both control and d*NAA60*-depleted cells exhibited bypolar spindles with correct alignment of chromosomes at the metaphase plate (D,E). Anaphase cells with chromosome segregation defects in d*NAA60*-depleted cells showed no obvious defects in Cnn and Cid localization (F,G). α-tubulin (red), Cid (green) and Cnn (blue). Control and d*NAA60*-depleted cells exhibit proper chromosome alignment during metaphase with no detectable defects in the actin and microtubule cytoskeleton (H,I). d*NAA60*-depleted cells undergoing anaphase with chromosome segregation defects also showed a normal actin and microtubule cytoskeleton (J,K; details show histone 3 phosphorylated on serine 10 staining). d*NAA60*-depleted cells in interphase show no detectable defects regarding the actin and microtuble cytoskeleton (L–M). Actin (red), α-Tubulin (green) and Histone 3 phosphorylated on Serine 10 (blue).

In order to assess the cellular function of dNaa60p, its expression was knocked down in *Drosophila* Dmel2 cells by RNAi. Similarly to d*NAA50*-depleted cells [30], [31] (data not shown), d*NAA60*-depleted cells showed chromosomal segregation defects during anaphase (Figure 8A–C, 8F, 8G, 8J, 8K). However, while d*NAA50*-depleted cells exhibit abnormal metaphases with an obvious mitotic arrest, control and d*NAA60*-depleted cells exhibited normal metaphases, with all chromosomes perfectly aligned within the spindle equator and without any mitotic arrest (Figure 8D, 8E, 8H, 8I and Figure S2). In contrast, during anaphase we consistently observed chromosome segregation defects in d*NAA60*-depleted cells, which included lagging chromosomes (Figure 8K, highlighted by asterisk) and chromosomal bridges (Figure 8B, 8G, highlighted by asterisk; quantification of abnormal anaphases is shown in Figure 8C). Chromosome lagging and bridging in d*NAA60*-depleted cells may be explained by kinetochore abnormalities; however we failed to detect any obvious defect in the localization of the Centromere identifier protein (Cid) during metaphase or anaphase (Figure 8D–G). We also failed to detect any obvious cohesion defect since the distance between kinetochores during metaphase was normal according to Cid localization (Figure 8D, 8E). Chromosome lagging could also be explained by centrosome/mitotic spindle defects. Yet, we did not detect any obvious defect in the localization of Centrosomin (Cnn), and the mitotic spindle was bipolar and correctly attached to chromosomes and centrosomes (Figure 8D–G). Furthermore, d*NAA60*-depleted cells showed no obvious defects in the actin and microtubule cytoskeleton in both mitotic and interphase cells (Figure 8H–M). Since d*NAA60*-depleted cells were otherwise normal, our data suggest that dNaa60p is required for chromosome segregation during anaphase. Naa60p-dependent N-terminal acetylation of one or more substrates is therefore likely to be required for chromosome segregation *in vivo*.

## Discussion

**Figure 9:**
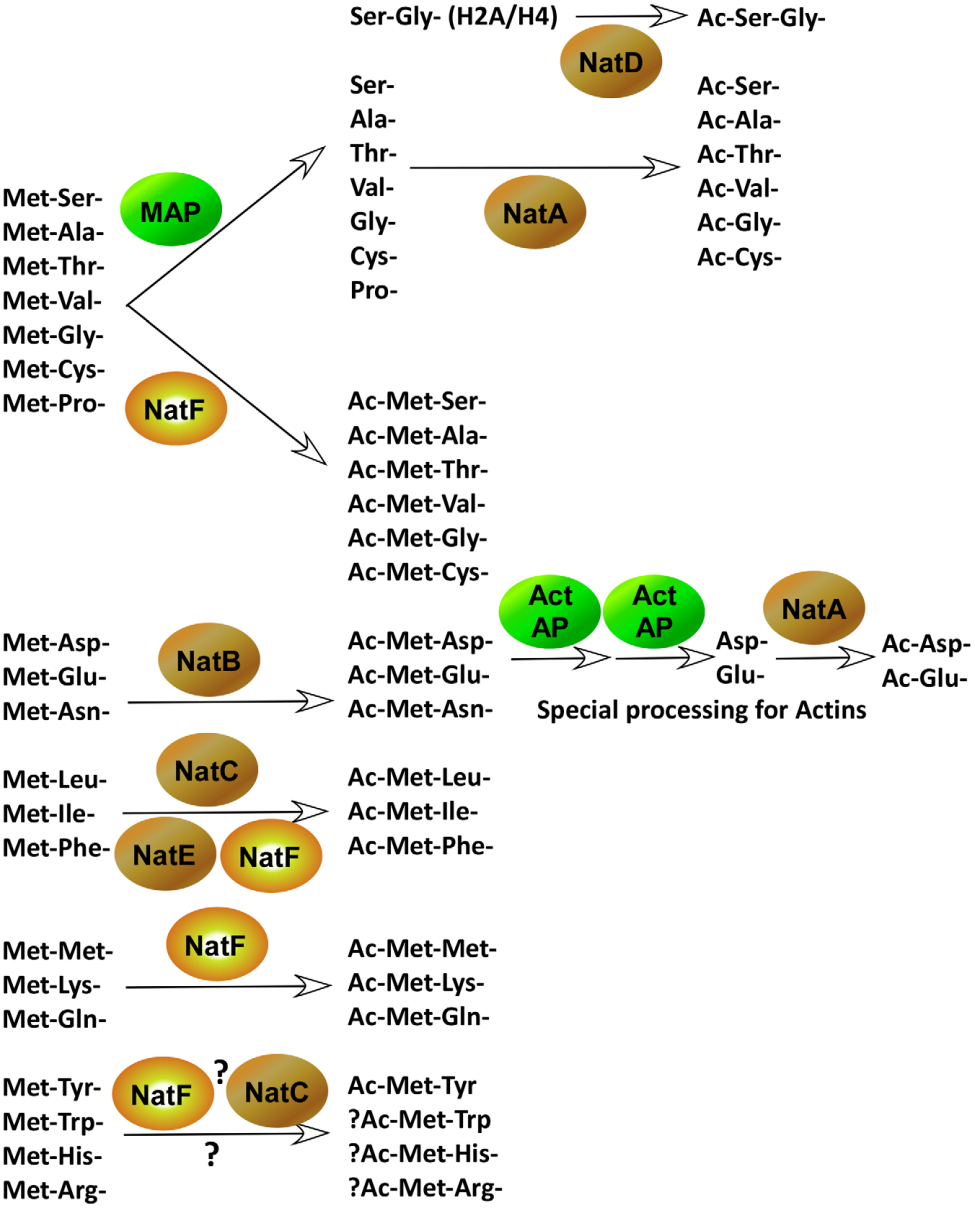
The major pathways of protein N-terminal processing in higher eukaryotes. N-termini of which the iMet is followed by one of the small amino acids, Ser-, Ala-, Thr-, Val-, Gly-, Cys-, and Pro- often undergo iMet cleavage performed by a methionine aminopeptidase (MAP). These N-termini, with the exception of Pro-, are often further acetylated by NatA, or in the case of Histone H2A and H4, by NatD (Hole K. *et al.*, unpublished). However, this group of N-termini may also be acetylated by NatF. Met-Asp-, Met-Glu- and Met-Asn- are acetylated by NatB. Actins are further processed in one or more steps by unidentified Actin aminopeptidases (Act AP). The acidic actin N-termini are then acetylated by at NAT, presumably NatA [38]. Hydrophobic Met-Leu-, Met-Ile- and Met-Phe- are acetylated by NatC, but also by NatF as well as by NatE *in vitro*, suggesting potential redundancy between these NATs. Met-Met-, Met-Lys- and Met-Gln- are acetylated by NatF and potentially other NATs. Information about Met-His-, Met-Arg-, Met-Trp- and Met-Tyr- is limited, but it is likely that some of these N-termini are acetylated as well, by NatF and perhaps NatC.

The basic co-translational machinery performing N-Ac in eukaryotes was believed to be fully identified and mostly characterized, with five NATs, NatA-NatE, each of which composed of specific subunits and acetylating its own subset of substrates [15]. However, the significant shift in occurrence of N-Ac from lower to higher eukaryotes, clearly points to the fact that species-specific factors are major determinants for N-Ac. Indeed, in the current study we revealed that higher eukaryotes express NatF/Naa60p, a unique NAT responsible for N-Ac of a large subset of eukaryotic proteins. These N-termini include Met-Lys-, Met-Met-, Met-Val- and Met-Ser- to which so far no NAT has been assigned. Also N-termini like Met-Leu- and Met-Ile-, previously believed to be solely NatC substrates, may be acetylated by NatF. Thus, the previous clear-cut classification between Nat substrate classes based on the N-terminal sequences should be re-evaluated when *in vivo* datasets are considered. The current knowledge on the NATs of higher eukaryotes and their corresponding substrates is presented in Figure 9.

In contrast to the N-termini acetylated by NatF, for the increased N-Ac of the processed (Met-)Ala- and (Met-)Val- N-termini there is presently no explanation. The intrinsic enzymatic properties of human and yeast NatA appeared to be very similar when expressed in yeast [18]. Co-determining factors that should be elaborated upon concerning the NatA substrates are interaction partners specific for NatA of higher eukaryotes, like HYPK which was demonstrated to modulate N-terminal acetylation [33]. Notwithstanding the generally lower expression levels, the existence of higher eukaryotic paralogues of Naa15p and Naa10p, being Naa16p and Naa11p respectively [42], [43], might additionally account for modulators of the observed N^α^-acetylome. However, information regarding their potential proteome-wide contribution to N-Ac is currently lacking.

We found that evolution of N-Ac prone N-termini most likely contributes only to a very small degree to the overall evolutionary shift in the occurrence of N-Ac. Furthermore, there might be a shift in the substrate specificity between species-specific NATs, for instance for the NatB, NatC and NatE activities, requiring further experimental validation. However viewing their strict evolutionary conservation, this may be rather unlikely.

The current data are more comprehensive as compared to previous analyses [18], and overall the 648 unique yeast and 1345 unique human N-termini identified were analysed for their acetylation status (Table 1, Tables S1 and S2). 68% of the yeast N-termini and 85% of the human N-termini are partially or fully N-terminally acetylated. Previously, we determined that 57% of yeast proteins and 84% of human proteins were N-terminally acetylated, thus implicating some shift in the N-Ac of the yeast N-termini between experiments. We believe the current dataset likely holds a more representative picture since more N-termini were sampled and since yeast was grown under slightly different deprivating (SILAC) conditions in the previous setup. Nevertheless, still a significant difference between yeast (68%) and humans (85%) can be observed and as demonstrated, this difference is significantly diminished in yeast expressing NatF (78%) (Figure 4 and Figure 5).

The current study provides to the best of our knowledge, the first evidence shedding light on the molecular basis of the evolutionary shift in the N^α^-acetylome from lower to higher eukaryotes. With the presence of NatF, higher eukaryotes are enforced in their capacity to acetylate Met-Lys-, Met-Leu- and other Met- starting N-termini, thus explaining in part the increased occurrence of N-Ac. This additional NAT may have evolved to meet the increased demands of more complex proteomes with a higher level of regulation. In light of the recent suggestion that N-Ac generates degrons and thus acts as a destabilizer [5], these issues will be of particular importance. Our results suggested that dNaa60 activity is likely to be specifically required for chromosome segregation during anaphase, as cells depleted for dNaa60 showed normal alignment of chromosomes during metaphase plates and progressed normally through mitosis, without any obvious cell cycle arrest (Figure 8 and Figure S2). With an increasing support for N-Ac in controlling protein stability, function and subcellular localization, it is very likely that Naa60p will emerge as a key regulator for several proteins. Future investigations will aim at elucidating these specific Naa60p substrates.

## Materials and Methods

### N-terminal dipeptide frequency analysis for *H. sapiens*, *D. melanogaster*, and *S. cerevisiae*

The random dipeptide frequencies (n = 400) were estimated by Monte Carlo sampling of one randomly selected decapeptide per protein in the databases of; *Homo sapiens*, *Drosophila melanogaster* and *Saccharomyces cerevisiae* (UniProt/SwissProt entries (version 2011-05)). After 100 sampling rounds, the mean and standard deviation for each dipeptide were estimated. Thereafter, the N-terminal dipeptide frequency of all decapeptides from position 2 to 11 were calculated, and the obtained frequencies compared with the random frequencies. The corresponding species-specific z-score, reflecting the amino acid dipetide frequency differences between the protein N-terminal and overall protein sequence were calculated as follows:
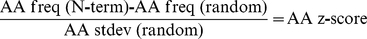



### Searching for novel human NATs

Sequences of the known human catalytic NAT units/subunits, hNaa10p (P41227), hNaa11p (Q9BSU3), hNaa20p (P61599), hNaa30p (Q147X3) and hNaa50p (Q9GZZ1), were used in the search of novel human NATs by making use of NCBI BLAST (blastp) queries (search set: ‘Swiss-Prot protein sequences’ restricted to organism: ‘Homo sapiens’ and otherwise the predefined parameters). Besides the known human NATs, there was in particular one significant hit, the uncharacterized NAT15 (Q9H7X0), which held sequence similarity to all query NATs with E-values between 3×10^−6^ and 0.24. NAT15 is an automatically annotated name due to the presence of a N-acetyltransferase domain (pfam00583) in the protein sequence. When using hNaa30p and hNaa50p as query sequences, NAT15 scored even better than some of the known human NATs (hNaa20p and hNaa10p/hNaa11p/hNaa20p, respectively). When using hNaa30p as query sequence, some other human proteins scored equally well as NAT15: NAT8 (Q9UHE5), NAT8B (Q9UHF3), NAT8L (Q8N9F0) and ATAC2/CRP2BP (Q9H8E8) with E-values ranging from 7×10^−5^ to 0.034. However, all these candidates were biochemically characterized as members of the GNAT family (pfam00583) with functions distinct from protein N-terminal acetylation. NAT8 is a cysteinyl-*S*-conjugate N-acetyltransferase catalyzing the last step of mercapturic acid formation while NAT8B is a likely pseudogene of NAT8 [44]. NAT8L catalyses the synthesis of N-acetylaspartate [45] and ATAC2 catalyses lysine acetylation on histone H4 [46]. Thus, NAT15 was the only uncharacterized protein with a significant similarity to the known human NATs (Figure 2) and was therefore further pursued.

### Construction of plasmids

Plasmid encoding V5-tagged NAT15/hNaa60p (Gene ID: 79903) used for mammalian expression was constructed from human HEK293 cDNA by use of Transcriptor Reverse Transcriptase (Roche). The PCR product containing the CDS plus four 5′ nucleotides (gaga) was inserted into the TOPO TA vector pcDNA 3.1/V5-His TOPO Invitrogen) according to the instruction manual. An *E. coli* expression vector encoding MBP-His-tagged hNaa60p was constructed by subcloning h*NAA60* from ph*NAA60*-V5 to the pETM-41 vector using the *Acc65I* and *NcoI* sites. pETM-41-d*NAA60*, encoding the predicted fruit fly Naa60p was made by subcloning the CG18177 CDS from pOT2-CG18177 (clone LD27619 from the Drosophila Genomics Resource Centre, Indiana University) to pETM-41. pETM-41 was generously provided by G. Stier, EMBL, Heidelberg. A yeast expression vector, pBEVY-U-h*NAA60* encoding untagged hNaa60p was constructed by subcloning h*NAA60* from ph*NAA60*-V5 to the pBEVY-U vector [47] using the *BamHI* and *SalI* sites.

### *E. coli* protein expression and purification

The plasmid pETM-41-h*NAA60* or pETM-41-d*NAA60* was transformed into *E. coli* BL21 Star™ (DE3) cells (Invitrogen) by heat shock. A 200 ml cell culture was grown in LB (Luria Bertani) medium to an OD_600_ nm of 0.6 at 37°C and subsequently transferred to 20°C. After 30 min of incubation, protein expression was induced by IPTG (1 mM). After 17 h of incubation, the cultures were harvested by centrifugation and the pellets stored at −20°C. *E. coli* pellets containing recombinant proteins were thawed at 4°C and the cells lysed by sonication in lysis buffer (1 mM DTT, 50 mM Tris-HCl (pH 7.5 or 8.3 for MBP-dNAA60p and MBP-hNAA60p, respectively), 300 mM NaCl, 1 tablet EDTA-free protease Inhibitor cocktail per 50 ml (Roche)). Following centrifugation, the cell extracts were applied on a metal affinity FPLC column (HisTrap HP, GE Healthcare, Uppsala, Sweden). MBP-hNaa60p and MBP-dNaa60p were eluted with 300 mM Imidazole in 50 mM Tris (pH 7.5 or 8.3 for MBP-dNAA60 or MBP-hNAA60, respectively), 300 mM NaCl and 1 mM DTT. Fractions containing recombinant protein were pooled and further purified using size exclusion chromatography (Superdex™ 75, GE Healthcare) until apparent purity as analysed by Coomassie stained SDS-PAGE gels. The protein concentrations were determined by OD_280_ nm measurements.

### *In vitro* peptide library-based NAT assay using hNaa60p and dNaa60p

Preparation of proteome derived peptide libraries. Proteome-derived peptide libraries were generated from human K-562 cells. Cells were repeatedly (3×) washed in D-PBS and then re-suspended at 7×10^6^ cells per ml in lysis buffer (50 mM sodium phosphate buffer pH 7.5, 100 mM NaCl, 1% CHAPS and 0.5 mM EDTA) in the presence of protease inhibitors (Complete protease inhibitor cocktail tablet (Roche Diagnostics, Mannheim, Germany)). After lysis for 10 min on ice, the lysate was cleared by centrifugation for 10 min at 16,000× g and solid guanidinum hydrochloride was added to the supernatant to a final concentration of 4 M. The protein samples were reduced and *S*-alkylated, followed by tri-deuteroacetylation of primary amines and digestion with trypsin as described previously [48], [49]. The resulting peptide mixtures were vacuum dried. The dried peptides were re-dissolved in 500 µl 50% acetonitrile. The sample was acidified to pH 3.0 using a stock solution of 1% TFA in 50% acetonitrile and further diluted with 10 mM sodium phosphate in 50% acetonitrile to a final volume of 1 ml. This peptide mixture was then loaded onto an AccuBONDII SCX SPE cartridge (Agilent Technologies, Waldbronn, Germany) and SCX separation (SCX fractionation 1) of N^α^-blocked N-terminal peptides (and C-terminal peptides) from N^α^-free peptides was performed as described previously [48], [50]. The flow-through containing the N^α^-blocked N-terminal peptides and C-terminal peptides was discarded and the SCX-bound fraction (containing the N^α^-free peptides) was collected by elution with 4 ml of 400 mM NaCl and 10 mM sodium phosphate in 40% of acetonitrile (pH 3.0). Eluted peptides were vacuum dried and re-dissolved in 1 ml of HPLC solvent A (10 mM ammonium acetate in 2% acetonitrile, pH 5.5). C18 solid-phase extraction (SPE desalting step) of the N^α^-free peptides was performed by loading the peptide mixture onto a AccuBONDII ODS-C18 SPE cartridge (1 ml tube, 100 mg, Agilent Technologies). This cartridge has a binding capacity of 1 mg of peptides and thus for each mg of material, a separate cartridge was used. Prior to sample loading, the cartridges were washed with 2 ml of 50% acetonitrile and then washed with 5 ml of HPLC solvent A. Sample loading was followed by washing the C18 cartridge with 5 ml of 2% acetonitrile. Peptides were eluted with 3 ml of 70% acetonitrile and subsequently vacuum dried.

*In vitro* peptide library-based NAT assay. 100 nmol of the desalted N^α^-free peptide pool was reconstituted in acetylation buffer (50 mM Tris-HCl (pH 8.5), 1 mM DTT, 800 µM EDTA, 10% glycerol) together with equimolar amounts of a stable isotope encoded variant of acetyl-CoA, ^13^C_2_-acetyl CoA, (99% ^13^C_2_-acetyl CoA, ISOTEC-Sigma (lithium salt)) and 1 nmol of enzyme (i.e. recombinant hNaa60p or dNaa60p) was added to a final reaction volume of 1 ml. The reaction was allowed to proceed for 1 h at 37°C and stopped by addition of acetic acid to a 5% final concentration. SPE was then performed as described above.

NAT oligopeptide-substrate recovery and RP-HPLC based separation. Peptides starting with pyroglutamate were unblocked prior to the second SCX fractionation step. Here, 25 µl of pGAPase (25 U/ml) (TAGZyme kit, Qiagen, Hilden, Germany) was activated for 10 min at 37°C by addition of 1 µl of 50 mM EDTA (pH 8.0), 1 µl of 800 mM NaCl, and 11 µl of freshly prepared 50 mM cysteamine-HCl. 25 µl of Qcyclase (50 U/ml, TAGZyme) was then added to the pGAPase solution. The dried peptides were re-dissolved in 212 µl of buffer containing 16 mM NaCl, 0.5 mM EDTA, 3 mM cysteamine, and 50 µM aprotinin. The activated pGAPase and Q-cyclase mixture was added to the peptide sample and the mixture (275 µl total volume) was incubated for 60 min at 37°C. 275 µl acetonitrile was then added and the sample was acidified to pH 3 using a 1% TFA stock solution in 50% acetonitrile. The sample was further diluted with 10 mM sodium phosphate in 50% acetonitrile to a final volume of 1 ml. SCX enrichment of N^α^-blocked N-terminal peptides was performed as described [48] (SCX fractionation 2). The SCX fraction containing the newly blocked N-terminal peptides was vacuum dried and re-dissolved in 100 µl of HPLC solvent A. To prevent oxidation of methionine between the primary and secondary RP-HPLC separations (and thus unwanted segregation of methionyl peptides [51], methionines were uniformly oxidized to sulfoxides prior to the primary RP-HPLC run by adding 2 µl of 30% (w/v) H_2_O_2_ (final concentration of 0.06%) for 30 min at 30°C. This peptide mixture was injected onto a RP-column (Zorbax 300SB-C18 Narrowbore, 2.1 mm (internal diameter)×150 mm length, 5 µm particles, Agilent Technologies) and the RP-HPLC separation was performed as described previously [48]. Fractions of 30 s wide were collected from 20 to 80 min after sample injection (120 fractions). To reduce LC-MS/MS analysis time, fractions eluting 12 min apart were pooled, vacuum dried and re-dissolved in 40 µl of 2% acetonitrile. In total, 24 pooled fractions per setup were subjected to LC-MS/MS analysis (see below).

LC-MS/MS analysis. LC-MS/MS analysis was performed using an Ultimate 3000 HPLC system (Dionex, Amsterdam, The Netherlands) in-line connected to a LTQ Orbitrap XL mass spectrometer (Thermo Electron, Bremen, Germany) and, per LC-MS/MS analysis, 2 µl of sample was consumed. LC-MS/MS analysis and generation of MS/MS peak lists were performed as described [52]. These MS/MS peak lists were then searched with Mascot using the Mascot Daemon interface (version 2.2.0, Matrix Science). The Mascot search parameters were set as follows. Searches were performed in the Swiss-Prot database with taxonomy set to human (UniProtKB/SwissProt database version 2010_05 containing 20,286 human protein sequences). Trideutero-acetylation at lysines, carbamidomethylation of cysteine and methionine oxidation to methionine-sulfoxide were set as fixed modifications. Variable modifications were trideutero-acetylation, acetylation and ^13^C_2_-acetylation of protein N-termini and pyroglutamate formation of N-terminal glutamine. Endoproteinase Arg-C/P (Arg-C specificity with arginine-proline cleavage allowed) was set as enzyme allowing no missed cleavages. The mass tolerance on the precursor ion was set to 10 ppm and on fragment ions to 0.5 Da. The estimated false discovery rate by searching decoy databases was typically found to lie between 2 and 4% on the spectrum level [48]. Quantification of the degree of N-Ac was done as previously explained [18].

### *In vitro* N-terminal acetylation assay using purified MBP-hNaa60p and synthetic peptides

Purified MBP-hNaa60p (500 nM) was mixed with selected oligopeptide substrates (200 µM) and 300 µM of acetyl-CoA in a total volume of 50 µl acetylation buffer (50 mM Tris (pH 8.5), 800 µM EDTA, 10% glycerol, 1 mM DTT) and incubated at 37°C for 35 min. The enzyme activities were quenched by the addition of 5 µl of 10% TFA. Peptide acetylation was quantified using RP-HPLC as described previously [23].

### Synthetic peptide sequences

Peptides were custom-made (Biogenes) to a purity of 80–95%. All peptides contain 7 unique amino acids at their N-terminus, as these are the major determinants influencing N-terminal acetylation. The next 17 amino acids are essentially identical to the ACTH peptide sequence (RWGRPVGRRRRPVRVYP) however; lysines were replaced by arginines to minimize any potential interference by N^ε^-acetylation. Oligopeptide sequences:

SYSM-RRR (ACTH (aa138–161, P01189): [H] SYSMDHF*RWGRPVGRRRRPVRVYP* [OH]; MDEL-RRR (NF-kκB p65, Q04206): [H] MDELFPL*RWGRPVGRRRRPVRVYP* [OH]; MLGT-RRR (hnRNP H, P31943): [H] MLGTEGG*RWGRPVGRRRRPVRVYP* [OH]; MAPL-RRR (Prot phosphatase 6, O00743): [H] MAPLDLD*RWGRPVGRRRRPVRVYP* [OH]; MLGP-RRR (hnRNP F, P52597): [H] MLGPEGG*RWGRPVGRRRRPVRVYP* [OH]; SESS-RRR (High mob. gr. prot A1, P17096): [H] SESSSKS*RWGRPVGRRRRPVRVYP* [OH]; MKKS-RRR (Septin 9, Q9UHD8): [H] MKKSYSG*RWGRPVGRRRRPVRVYP* [OH].

### Yeast strain generation and cultivation for *in vivo* N-terminal acetylation analysis

The *S. cerevisiae* MATalpha strain BY4742 (Euroscarf) was transformed with pBEVY-U or pBEVY-U-h*NAA60* and transformants were selected on plates lacking uracil. The two strains generated, BY4742-pBEVY-U (yeast normal) and BY4742-pBEVY-U-h*NAA60* (yeast+NatF), were cultivated in 300 ml synthetic medium lacking uracil (Sigma) to an OD600nm of ∼3. After harvesting, cells were washed twice in lysis buffer (50 mM Tris, 12 mM EDTA, 250 mM NaCl, 140 mM Na_2_HPO_4_ (pH 7.6) supplemented with a complete protease inhibitor mixture tablet (1 tablet per 100 mL) (Roche Diagnostics) and glass beads were added before several rounds of vortex/ice (10×). One milliliter of lysis buffer was used for a pellet resulting from 300 mL of yeast culture. The lysates were centrifuged at 5000× *g* for 10 min and the retained supernatants were analyzed by COFRADIC analyses. Aliquots were analysed by SDS-PAGE and Western blotting using anti-hNaa60p. Solid guanidinium hydrochloride was added to a final concentration of 4 M in order to inactivate proteases and denature all proteins. Subsequently, proteins were reduced and alkylated simultaneously, using TCEP.HCl (1 mM final concentration (f.c.)) and IAA (2 mM f.c.) respectively, for 1 h at 30°C. Subsequent steps of the N-terminal COFRADIC protocol were performed as described previously [48]. Aliquots were analysed by SDS-PAGE and Western blotting using anti-hNaa60p.

### Human cell culture and transfection

HeLa cells (epithelial cervix adenocarcinoma, ATCC CCL-2) were cultured in Glutamax-containing DMEM medium supplemented with 10% dialyzed foetal bovine serum (Invitrogen, Carlsbad, CA, USA), 100 units/ml penicillin (Invitrogen) and 100 µg/ml streptomycin (Invitrogen). Cells were grown in media containing either natural (^12^C_6_) or ^13^C_6_^15^N_4_ L-arginine (Cambridge Isotope Labs, Andover, MA, USA) [53] at a concentration of 80 µM (i.e. 20% of the suggested concentration present in DMEM at which L-arginine to proline conversion was not detectable for HeLa cells). Cells were cultured for at least six population doublings to ensure complete incorporation of the labeled arginine. Human K-562 cells (ATCC CCL-243) were grown in Glutamax-containing RPMI-1640 medium supplemented with 10% foetal calf serum, 100 units/ml penicillin and 100 µg/ml streptomycin. Cells were cultured at 37°C and in 5% CO_2_.

Plasmid transfections were performed using Fugene6 (Roche) according to the instruction manual. siRNA transfections were performed using Dharmafect 1 (Dharmacon). In the overexpression experiment, 10×10 cm dishes of cells cultivated in ^13^C_6_^15^N_4_ L-arginine were transfected with control vector and cells cultivated in ^12^C_6_ L-arginine were transfected with ph*NAA60*-V5. Cells were harvested 48 hours post-transfection. Aliquots were analysed by SDS-PAGE and Western blotting using anti-V5 (Invitrogen) to confirm efficient overexpression (See Figure 6A). In the knockdown experiment, 10×10 cm dishes of control control cells cultivated in ^12^C_6_ L-arginine were transfected with 50 nM si-non-targeting control (D-001810, Dharmacon) and cells cultivated in ^13^C_6_^15^N_4_ L-arginine were transfected with 50 nM sih*NAA60* pool (D-014479, Dharmacon). After 48 hours of transfection, the medium was replaced by new SILAC medium containing 5 µM zVAD-fmk. After 84 hours, cells were harvested, lysed and subjected to COFRADIC analysis as described previously [18]. Aliquots were analysed by SDS-PAGE and Western blotting using anti-hNaa60p (Custom made affinity purified rabbit antibody targeting a peptide corresponding to aa 69–82 of hNaa60p, Biogenes) to confirm efficient knockdown (See Figure 6B). Each sample of the knockdown- and overexpression experiments resulted from 10×10 cm dishes of cells and was processed further for N-terminal COFRADIC analyses as described previously [18].

### Quantification of the degree of N^α^-acetylation

The ratios of N^α^-acetylation for all N-termini were quantified using MASCOT Distiller. The extent of N^α^-acetylation was calculated after extracting the corresponding peak intensities (extracted from the resulting rov-files). The modified peptide sequences were used to calculate the theoretical isotope peak distribution using the MS-isotope pattern calculator (http://prospector.ucsf.edu). For both variants (i.e., in vivo N^α^-acetylated (peak at *m*/*z*) and *in vitro* ^13^C_2_D_3_-N^α^-acetylated (peak at *m*/*z*+5 Da)), the predicted intensity of the 5^th^ contributing isotope was subtracted from the measured intensity of the corresponding monoisotopic peak of the other overlapping isotopic envelopes in order to correct for overlapping isotopic envelopes. Only the corresponding highest scoring MS/MS-spectra were withheld and inspected to evaluate the calculated N^α^-acetylation degree (in case of inconsistencies, whenever possible the second, third or next highest scoring MS/MS-spectra were inspected to evaluate the calculated N^α^-acetylation degree, if inconclusive the status was set as “N.D.”). When unclear MS-spectra were observed, the N-Ac status was also documented as “N.D.”. When no clear isotopic envelope was present for one of the possible variants, the status was set at 0% and 100% or 100% and 0% respectively. Further, if the N^α^-acetylation calculated was ≤2% of ≥98%, the overall N-Ac status was accounted for as being free or fully N-Ac respectively.

When comparing the degrees of N^α^-acetylation from two independent control experiments (with the degrees of N^α^-acetylation of more than 1,000 unique N-termini calculated) and taking into account a [x−10%, x+10%] interval around the calculated x-value (the x-value being the degree (%) of N^α^-acetylation for the calculated data point in one dataset), the p-value was calculated to be p<0.01, indicating that upon setting these limits, less than 1% of all measured N-Ac values differed more than 10%. Therefore, a significant variation in the degree of N^α^-acetylation was set to 10% or more. In the case of free N-termini identified in a control setup however, significance was set to 5% since in this case two isotopic envelopes could clearly be distinguished.

### RNA interference and immunofluorescence microscopy of *Drosophila* Dmel2 cells

Dmel2 cells were cultured at 25°C and RNAi was performed according to standard procedures. To deplete dNaa60 (CG18177), Dmel2 cells were separately transfected with two different double-stranded RNAs (dsRNA) corresponding to fragments of dNaa60 defined by the set of primers (Forward-1) CAACAAACACAGTGCGCC and (Reverse-1) CACATTTCGATAGGGTTTGATTTC or (Forward-2) GACTCGATGGGTCGTTCCGC and (Reverse-2) GTGGATGGCCGCCGTTAAT. GFP-targeting dsRNA was used as control. Each primer incorporates a T7 RNA polymerase binding site. All PCR products were used as template to synthesize dsRNA by use of the T7 RiboMAX Express kit (Promega). *Drosophila* Dmel2 cells were grown in SFM Medium (GIBCO) supplemented with 1× glutamine and 1× PenStrep (GIBCO). Cells were counted and diluted to 2×10^6^ cells/ml in SFM medium supplemented with glutamine. Cells were incubated during 1 h with 40 µg for each dsRNA at a concentration of 1 µg/µl. After 1 h incubation with dsRNA, 3 ml of SFM media supplemented with glutamine and PenStrep (GIBCO) was added back. After 93 h dsRNA treatment, 2×10^6^ cells were added to coverslips by 1 h incubation at 25°C. Cells were fixed with 4% formaldehyde, 0.03 M PIPES, 0.11 M HEPES, 0.01 M EGTA and 4 mM MgSO_4_ for 10 min, followed by two washes in 1× PBS. Permeabilization and blocking was performed for 1 h with PBS-TB (PBS, 0.1% Triton X-100, 1% fetal bovine serum). Primary antibody incubations were done in blocking solution for 2 h at room temperature or overnight at 4°C, followed by three 5 min washes in PBS-TB. Secondary antibody incubations were performed as described for the primary antibodies, including three 5 min washes. Primary antibodies included mouse anti-α-tubulin DM1A (1∶500; Sigma), rabbit anti-pSer10-Histone H3 (1∶500; Upstate Biotechnology), chicken anti-Cid (1∶500; kindly provided by David Glover's laboratory) and rabbit anti-Cnn (1∶500; kindly provided by Jordan Raff). F-actin was stained with rhodamine-conjugated phalloidin (Sigma) and DNA was stained with DAPI at 1∶1000 (stock concentration 1 mg/ml), with the addition of 5 µg/ml RNAse A. Visualization of fixed cells was performed using a Delta Vision Core System (Applied Precision) using a 100× UplanSApo objective and a cascade2 EMCCD camera (Photometrics). Images were acquired as a series of z-sections separated by 0.2-µm intervals. Deconvolution was performed using the conservative ratio method in softWoRx software. Phenotypic quantification was performed using a regular Epifluorescent microscope Leica DMRA2.

## Supporting Information

Figure S1 Amino acid frequencies at position 2 of hNaa60p and dNaa60p substrates. Bar charts of the amino acid frequencies at the 2^nd^ position in the Met- (black bars) and Leu-starting (red bars) oligopeptide substrates identified in proteome-derived peptide library screens of hNaa60p (upper panel) and dNaa60p (lower panel).
(TIF)

Figure S2 d*NAA50* but not d*NAA60* dsRNAi treated cells arrest in mitosis. Graph showing mitotic index in control, *dNAA60* and *dNAA50* dsRNA treated Dmel2 cells. Mitotic index is the percentage of cells positive for phospho-Histone H3 (pSer10).
(TIF)

Table S1 List of 868 unique N-terminal peptides (start position 1 or 2) identified in the proteome of the control yeast strain and/or the yeast strain expressing hNaa60p.
(DOC)

Table S2 List of 1,497 human N-terminal peptides (start position 1 or 2) identified in the hNaa60p overexpression or knockdown experiments in HeLa cells.
(DOC)

Table S3 Relating the occurrence of N-Ac and different N-termini in yeast and humans. An unbiased estimation of N-Ac for all methionine-starting yeast (6613) and human SwissProt entries (20102) (SwissProt version 57.8) was performed based on the nature of the N-terminal amino acids and the N-terminal acetylation status uncovered in this study.
(DOC)

Table S4 List of the 72 unique *in vivo* hNaa60p substrate N-termini identified in yeast. S4A. hNaa60p yeast substrate N-termini (44) which were completely unacetylated in the control setup analyzed. S4B. hNaa60p yeast substrate N-termini (28) which were partially N-Ac in the control setup analyzed.
(DOC)

Table S5 List of N-termini affected in their N-Ac status by knockdown or overexpression of hNaa60p in HeLa cells.
(DOC)
