# Supplementary figures and images for: NatF Contributes to an Evolutionary Shift in Protein N-Terminal Acetylation and Is Important for Normal Chromosome Segregation
Source: PLoS Genet. 2011 Jul 7;7(7):e1002169. doi: 10.1371/journal.pgen.1002169 (PMC3131286; doi:10.1371/journal.pgen.1002169)

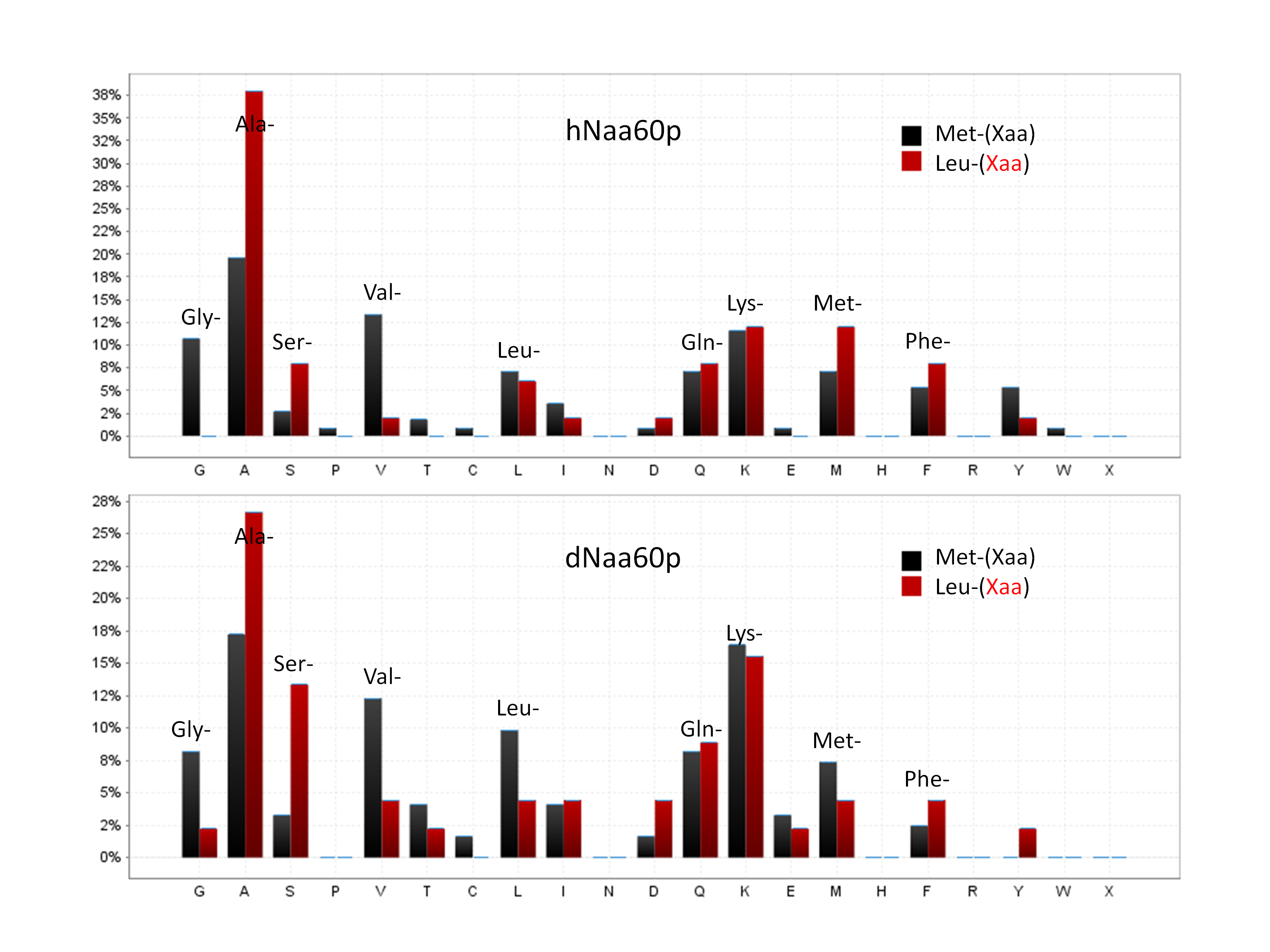

Supplement: Figure S1 — Amino acid frequencies at position 2 of hNaa60p and dNaa60p substrates. Bar charts of the amino acid frequencies at the 2nd position in the Met- (black bars) and Leu-starting (red bars) oligopeptide substrates identified in proteome-derived peptide library screens of hNaa60p (upper panel) and dNaa60p (lower panel). (TIF) [file pgen.1002169.s001.tif]

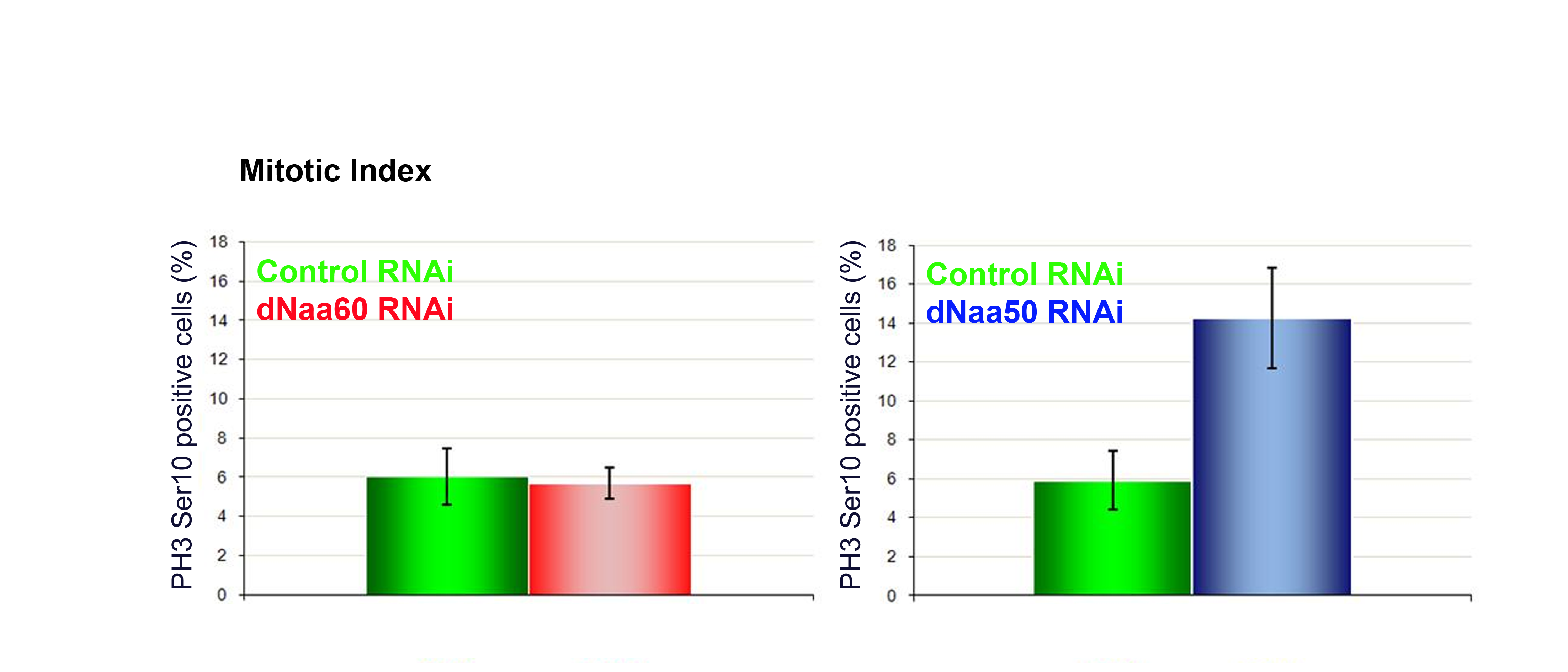

Supplement: Figure S2 — dNAA50 but not dNAA60 dsRNAi treated cells arrest in mitosis. Graph showing mitotic index in control, dNAA60 and dNAA50 dsRNA treated Dmel2 cells. Mitotic index is the percentage of cells positive for phospho-Histone H3 (pSer10). (TIF) [file pgen.1002169.s002.tif]
