# Supplementary material for: NatF Contributes to an Evolutionary Shift in Protein N-Terminal Acetylation and Is Important for Normal Chromosome Segregation
Source: PLoS Genet. 2011 Jul 7;7(7):e1002169. doi: 10.1371/journal.pgen.1002169 (PMC3131286; doi:10.1371/journal.pgen.1002169)
Supplement: Table S1 — List of 868 unique N-terminal peptides (start position 1 or 2) identified in the proteome of the control yeast strain and/or the yeast strain expressing hNaa60p. (DOC) [file pgen.1002169.s003.doc]

| **Table S1. List of 868 unique N-terminal peptides (start position 1 or 2) identified in the proteome of the control yeast strain and/or the yeast strain expressing hNaa60p.** Start and end positions, N-term modification status/states confirmed by MS/MS, corresponding peptide sequence identified, global N-term modification status in control yeast (N-termini found to be less than 2% Ac and more than 98%, were considered as 100% free and 100% Ac respectively when considering their global Ac-status), %Ac of the N-terminus in the control yeast strain, %Ac of the N-terminus in the yeast strain expressing hNaa60p, difference in %Ac, UniProt database primary accession number, Uniprot name, sample(s) in which the N-terminus was/were identified, protein description, number of identified spectra (count), highest Mascot ion score, minimum Mascot identity-threshold and, whenever a peptide matched to multiple members of a protein family (redundancy), isoforms are given for all uniquely identified yeast N-termini. N-termini are categorized according to their N-terminal amino acid residue identified and alphabetically ranked according to their identified peptide sequence. Whenever the N-terminus was not identified in one of the two proteomes analyzed this is indicated with N.I. (not identified), whenever the (overall) Ac-status could not be calculated, the (overall) Ac-status s being set as N.D. (not determined).  ______________________________________________________________________________________________________________________________________________________________________________________ | | | | | | | | | | | | | | | | | | | | | |
| --- | --- | --- | --- | --- | --- | --- | --- | --- | --- | --- | --- | --- | --- | --- | --- | --- | --- | --- | --- | --- | --- |
|  |  | | |  | |  | |  |  |  |  |  |  | |  |  |  |  |  |  |  |
| **start** | **end** | | | **N-term modifications** | | **AA 1-2** | | **sequence** | **N-term modification status control yeast** | **avg. %Ac control** | **avg %Ac. hNaa60p yeast** | **∆ Ac** | **accession** | | **Uniprot** | **sample ID** | **Description** | **isoforms** | **count(*)** | **max(MACOT score)** | **ID-threshold** |
| **1. Ala-** | | | |  | |  | |  |  |  |  |  |  | |  |  |  |  |  |  |  |
| 2 | | 30 | | AcD3C13 | | AA | | AAEKILTPESQLKKSKAQQKTAEQVAAER | partial Ac- | 10% | N.I. | N.D. | P05737 | | RL7A_YEAST | control | **60S ribosomal protein L7-A** |  | 12 | 77 | 32 |
| 2 | | 17 | | AcD3C13 | | AA | | AAGKLKKEQQNQSAER | partial Ac- | 4% | N.I. | N.D. | P33339 | | TFC4_YEAST | control | **Transcription factor tau 131 kDa subunit** |  | 1 | 43 | 37 |
| 2 | | 23 | | AcD3C13 | | AA | | AAGVPKIDALESLGNPLEDAKR | partial Ac- | 6% | 7% | 0% | A6ZNU5 | | RPIA_YEAST | hNaa60p,control | **Ribose-5-phosphate isomerase** | Q12189 (2-23) | 2 | 47 | 36 |
| 2 | | 14 | | Ace | | AA | | AANSVGKMSEKLR | 100% Ac | 91% | 92% | 1% | P32912 | | VAM7_YEAST | control,hNaa60p | **Vacuolar morphogenesis protein 7** |  | 2 | 89 | 37 |
| 2 | | 8 | | AcD3C13 | | AA | | AAQKSFR | 100% free | 0% | 0% | 0% | P04650 | | RL39_YEAST | control,hNaa60p | **60S ribosomal protein L39** |  | 71 | 62 | 35 |
| 2 | | 12 | | AcD3C13,Ace | | AA | | AAQNAFEQKKR | N.D. | N.I. | 13% | N.D. | P53177 | | TYW3_YEAST | hNaa60p | **tRNA wybutosine-synthesizing protein 3** |  | 2 | 53 | 37 |
| 2 | | 27 | | Ace | | AA | | AARPQQPPMEMPDLSNAIVAQDEMGR | partial Ac- | 89% | N.D. | N.D. | P40413 | | TCPE_YEAST | hNaa60p,control | **T-complex protein 1 subunit epsilon** |  | 2 | 65 | 33 |
| 2 | | 16 | | AcD3C13 | | AA | | AATKEAKQPKEPKKR | 100% free | 0% | N.I. | N.D. | P11633 | | NHP6B_YEAST | control | **Non-histone chromosomal protein 6B** |  | 7 | 60 | 30 |
| 2 | | 12 | | Ace,AcD3C13 | | AD | | ADLQKQENSSR | partial Ac- | 91% | 90% | -1% | Q03790 | | NUP53_YEAST | hNaa60p,control | **Nucleoporin NUP53** |  | 3 | 69 | 37 |
| 2 | | 17 | | Ace,AcD3C13 | | AD | | ADQENSPLHTVGFDAR | partial Ac- | 30% | N.D. | N.D. | Q01519 | | COX12_YEAST | control,hNaa60p | **Cytochrome c oxidase polypeptide VIb** |  | 3 | 79 | 34 |
| 2 | | 19 | | AcD3C13 | | AD | | ADYSSLTVVQLKDLLTKR | partial Ac- | 37% | N.I. | N.D. | P40040 | | THO1_YEAST | control | **Protein THO1** |  | 3 | 79 | 34 |
| 2 | | 21 | | Ace | | AE | | AEASIEKTQILQKYLELDQR | N.D. | N.D. | 100% | N.D. | P32288 | | GLNA_YEAST | control,hNaa60p | **Glutamine synthetase** |  | 35 | 96 | 37 |
| 2 | | 15 | | Ace | | AE | | AEESTDFSQFEEER | 100% Ac | 100% | 100% | 0% | Q12434 | | GDIR_YEAST | control,hNaa60p | **Rho GDP-dissociation inhibitor** |  | 5 | 82 | 27 |
| 2 | | 10 | | Ace | | AE | | AEKEEAIFR | 100% Ac | 100% | 100% | 0% | P32563 | | VPH1_YEAST | hNaa60p,control | **V-type proton ATPase subunit a, vacuolar isoform** |  | 2 | 65 | 37 |
| 2 | | 11 | | AcD3C13 | | AF | | AFLNIFKQKR | N.D. | N.D. | 8% | N.D. | P38281 | | APD1_YEAST | control,hNaa60p | **Actin patches distal protein 1** |  | 4 | 78 | 32 |
| 2 | | 15 | | AcD3C13 | | AF | | AFQKDAKSSAYSSR | 100% free | 0% | 0% | 0% | P26321 | | RL5_YEAST | hNaa60p,control | **60S ribosomal protein L5** |  | 133 | 91 | 37 |
| 2 | | 9 | | Ace | | AG | | AGAIENAR | 100% Ac | 100% | 100% | 0% | P17255 | | VATA_YEAST | control,hNaa60p | **V-type proton ATPase catalytic subunit A** |  | 36 | 74 | 36 |
| 2 | | 31 | | AcD3C13 | | AG | | AGAPAPPPPPPPPALGGSAPKPAKSVMQGR | 100% free | 0% | 6% | 6% | P37370 | | VRP1_YEAST | control,hNaa60p | **Verprolin** |  | 3 | 49 | 36 |
| 2 | | 10 | | AcD3C13,Ace | | AG | | AGATSSIIR | partial Ac- | 8% | 30% | 22% | P40474 | | YIM1_YEAST | control,hNaa60p | **Uncharacterized transporter YIL121W** |  | 3 | 72 | 35 |
| 2 | | 14 | | AcD3C13 | | AG | | AGENPKKEGVDAR | partial Ac- | 4% | 3% | 0% | Q06344 | | ESF1_YEAST | control,hNaa60p | **Pre-rRNA-processing protein ESF1** |  | 6 | 94 | 37 |
| 2 | | 19 | | AcD3C13 | | AG | | AGKKIAGVLGATGSVGQR | 100% free | 0% | 0% | 0% | P13663 | | DHAS_YEAST | hNaa60p,control | **Aspartate-semialdehyde dehydrogenase** |  | 20 | 152 | 32 |
| 2 | | 8 | | AcD3C13 | | AG | | AGKKSPR | partial Ac- | 25% | N.D. | N.D. | P39520 | | IFH1_YEAST | hNaa60p,control | **Protein IFH1** |  | 3 | 54 | 32 |
| 2 | | 10 | | AcD3C13 | | AG | | AGLKDVVTR | 100% free | 0% | 0% | 0% | P0C2H8 | | RL31A_YEAST | hNaa60p,control | **60S ribosomal protein L31-A** | P0C2H9 (2-10) | 95 | 59 | 35 |
| 2 | | 11 | | AcD3C13 | | AG | | AGLSFDNYQR | N.D. | N.I. | 17% | N.D. | P25043 | | PSB7_YEAST | hNaa60p | **Proteasome component PUP1** |  | 1 | 44 | 36 |
| 2 | | 14 | | Ace | | AG | | AGNANSVDEEVTR | partial Ac- | 88% | 88% | 0% | Q02256 | | PVH1_YEAST | hNaa60p,control | **Tyrosine-protein phosphatase YVH1** |  | 2 | 83 | 34 |
| 2 | | 16 | | AcD3C13 | | AG | | AGQVLDGKACAQQFR | 100% free | 0% | 0% | 0% | P07245 | | C1TC_YEAST | control,hNaa60p | **C-1-tetrahydrofolate synthase, cytoplasmic** |  | 3 | 78 | 38 |
| 2 | | 16 | | AcD3C13 | | AG | | AGSQLKNLKAALKAR | 100% free | 0% | N.I. | N.D. | Q99207 | | NOP14_YEAST | control | **Nucleolar complex protein 14** |  | 1 | 94 | 28 |
| 2 | | 10 | | AcD3C13 | | AG | | AGVKAYELR | 100% free | 0% | 0% | 0% | P39741 | | RL35_YEAST | hNaa60p,control | **60S ribosomal protein L35** |  | 240 | 80 | 36 |
| 2 | | 10 | | AcD3C13,Ace | | AI | | AIENIYIAR | N.D. | N.D. | 9% | N.D. | P53929 | | YNK8_YEAST | hNaa60p,control | **Uncharacterized protein YNL108C** |  | 10 | 80 | 36 |
| 2 | | 13 | | AcD3C13 | | AI | | AIQKVSNKDLSR | N.D. | N.I. | 0% | N.D. | P40505 | | SDS3_YEAST | hNaa60p | **Transcriptional regulatory protein SDS3** |  | 2 | 45 | 35 |
| 2 | | 15 | | AcD3C13 | | AI | | AISKNLPILKNHFR | 100% free | 0% | N.I. | N.D. | P40212 | | RL13B_YEAST | control | **60S ribosomal protein L13-B** | Q12690 (2-15) | 2 | 71 | 31 |
| 2 | | 9 | | AcD3C13 | | AK | | AKKAIDSR | N.D. | N.I. | 4% | N.D. | P53914 | | YNN2_YEAST | hNaa60p | **UPF0202 protein YNL132W** |  | 1 | 44 | 34 |
| 2 | | 9 | | AcD3C13 | | AK | | AKKKSKSR | 100% free | 0% | 0% | 0% | Q04500 | | UTP14_YEAST | hNaa60p,control | **U3 small nucleolar RNA-associated protein 14** |  | 9 | 52 | 24 |
| 2 | | 15 | | AcD3C13 | | AK | | AKKNKKDKEAKKAR | 100% free | 0% | N.I. | N.D. | Q08979 | | KEL3_YEAST | control | **Kelch repeat-containing protein 3** |  | 1 | 63 | 22 |
| 2 | | 12 | | AcD3C13 | | AK | | AKQSLDVSSDR | 100% free | 0% | 0% | 0% | P05743 | | RL26A_YEAST | hNaa60p,control | **60S ribosomal protein L26-A** | P53221 (2-12) | 101 | 90 | 37 |
| 2 | | 14 | | AcD3C13 | | AK | | AKSKSSQGASGAR | 100% free | 0% | 0% | 0% | Q12206 | | WTM2_YEAST | hNaa60p,control | **Transcriptional modulator WTM2** |  | 5 | 91 | 37 |
| 2 | | 9 | | AcD3C13 | | AK | | AKTIKVIR | N.D. | N.I. | 10% | N.D. | P53217 | | YG1I_YEAST | hNaa60p | **Uncharacterized membrane protein YGR026W** |  | 1 | 53 | 25 |
| 2 | | 9 | | AcD3C13 | | AK | | AKTLAQGR | 100% free | 0% | N.I. | N.D. | P13483 | | DATI_YEAST | control | **Oligo(A)/oligo(T)-binding protein** |  | 1 | 49 | 35 |
| 2 | | 13 | | Ace | | AL | | ALEPIDYTTHSR | 100% Ac | 100% | 100% | 0% | P15891 | | ABP1_YEAST | control,hNaa60p | **Actin-binding protein** |  | 4 | 76 | 36 |
| 2 | | 15 | | AcD3C13 | | AL | | ALGNEINITNKLKR | 100% free | 0% | 0% | 0% | P38805 | | RPF1_YEAST | hNaa60p,control | **Ribosome production factor 1** |  | 2 | 61 | 33 |
| 2 | | 15 | | AcD3C13,Ace | | AL | | ALPIEGKLSMANNR | N.D. | N.I. | N.D. | N.D. | P38835 | | YHT1_YEAST | hNaa60p | **PH domain-containing protein YHR131C** |  | 2 | 48 | 37 |
| 2 | | 13 | | Ace | | AM | | AMNTGGFDSMQR | 100% Ac | 100% | 100% | 0% | P48563 | | MON2_YEAST | hNaa60p,control | **Protein MON2** |  | 4 | 82 | 28 |
| 2 | | 9 | | Ace,AcD3C13 | | AN | | ANDLVQAR | N.D. | N.D. | N.D. | N.D. | P39516 | | RS14B_YEAST | control,hNaa60p | **40S ribosomal protein S14-B** |  | 17 | 72 | 36 |
| 2 | | 18 | | AcD3C13 | | AN | | ANDQDPNKSLINDALTR | N.D. | N.I. | 64% | N.D. | Q08966 | | PCL8_YEAST | hNaa60p | **PHO85 cyclin-8** |  | 1 | 44 | 38 |
| 2 | | 12 | | AcD3C13 | | AN | | ANPTTGKSSIR | 100% free | 0% | N.I. | N.D. | P13259 | | PCY1_YEAST | control | **Choline-phosphate cytidylyltransferase** |  | 1 | 66 | 37 |
| 2 | | 8 | | AcD3C13 | | AN | | ANQKQMR | N.D. | N.I. | 5% | N.D. | Q12044 | | RCN2_YEAST | hNaa60p | **Regulator of calcineurin 2** |  | 1 | 39 | 37 |
| 2 | | 10 | | Ace | | AN | | ANSKFGYVR | N.D. | N.I. | 100% | N.D. | P53215 | | THG1_YEAST | hNaa60p | **tRNA(His) guanylyltransferase** |  | 1 | 51 | 37 |
| 2 | | 23 | | AcD3C13 | | AP | | APGKKVAPAPFGAKSTKSNKTR | 100% free | 0% | N.I. | N.D. | P17076 | | RL8A_YEAST | control | **60S ribosomal protein L8-A** |  | 1 | 39 | 30 |
| 2 | | 12 | | AcD3C13 | | AP | | APLTKKTNGKR | 100% free | 0% | 0% | 0% | Q04373 | | PUF6_YEAST | control,hNaa60p | **Pumilio homology domain family member 6** |  | 3 | 82 | 30 |
| 2 | | 27 | | AcD3C13 | | AP | | APSAKATAAKKAVVKGTNGKKALKVR | 100% free | 0% | 0% | 0% | P04456 | | RL25_YEAST | control,hNaa60p | **60S ribosomal protein L25** |  | 53 | 63 | 16 |
| 2 | | 18 | | AcD3C13 | | AP | | APTNLTKKPSQYKQSSR | 100% free | 0% | 0% | 0% | Q12080 | | YP146_YEAST | hNaa60p,control | **Uncharacterized protein YPL146C** |  | 2 | 61 | 36 |
| 2 | | 15 | | AcD3C13 | | AP | | APTQLDIKVKALKR | 100% free | 0% | 0% | 0% | P48606 | | TBCA_YEAST | control,hNaa60p | **Tubulin-specific chaperone A** |  | 47 | 58 | 26 |
| 2 | | 8 | | AcD3C13,Ace | | AS | | ASEKEIR | N.D. | N.D. | N.D. | N.D. | P08524 | | FPPS_YEAST | control,hNaa60p | **Farnesyl pyrophosphate synthetase** |  | 6 | 57 | 37 |
| 2 | | 12 | | Ace | | AS | | ASIDAFSDLER | N.D. | N.I. | 100% | N.D. | P40014 | | SPC25_YEAST | hNaa60p | **Kinetochore protein SPC25** |  | 1 | 62 | 36 |
| 2 | | 9 | | Ace | | AS | | ASIGSQVR | 100% Ac | 100% | N.I. | N.D. | P43535 | | GCN20_YEAST | control | **Protein GCN20** |  | 1 | 52 | 37 |
| 2 | | 10 | | Ace | | AS | | ASNEVLVLR | 100% Ac | 100% | 100% | 0% | P38011 | | GBLP_YEAST | hNaa60p,control | **Guanine nucleotide-binding protein subunit beta-like protein** |  | 21 | 82 | 36 |
| 2 | | 23 | | Ace | | AS | | ASTSNTFPPSQSNSSNNLPTSR | 100% Ac | 100% | N.I. | N.D. | A6ZLF4 | | SDS24_YEAST | control | **Protein SDS24** | P38314 (2-23) | 2 | 70 | 34 |
| 2 | | 15 | | Ace | | AT | | ATFNPQNEMENQAR | 100% Ac | 100% | 100% | 0% | Q12515 | | YD173_YEAST | hNaa60p,control | **Uncharacterized protein YDL173W** |  | 12 | 84 | 31 |
| 2 | | 20 | | Ace | | AT | | ATIAPTAPITPPMESTGDR | 100% Ac | 100% | 100% | 0% | P07259 | | PYR1_YEAST | control,hNaa60p | **Protein URA1** |  | 4 | 77 | 36 |
| 2 | | 8 | | Ace | | AT | | ATKQLLR | 100% Ac | 100% | N.I. | N.D. | P40099 | | FTHC_YEAST | control | **5-formyltetrahydrofolate cyclo-ligase** |  | 1 | 52 | 34 |
| 2 | | 33 | | Ace | | AT | | ATLEELDAQTLPGDDELDQEILNLSTQELQTR | N.D. | N.D. | N.I. | N.D. | P33297 | | PRS6A_YEAST | control | **26S protease regulatory subunit 6A** |  | 1 | 48 | 36 |
| 2 | | 12 | | Ace | | AT | | ATQALEDITER | N.D. | N.I. | 100% | N.D. | P25591 | | VAC17_YEAST | hNaa60p | **vacuole-related protein 17** |  | 1 | 41 | 36 |
| 2 | | 24 | | Ace | | AT | | ATYQPYNEYSSVTGGGFENSESR | 100% Ac | 98% | N.I. | N.D. | P26754 | | RFA2_YEAST | control | **Replication factor A protein 2** |  | 1 | 42 | 28 |
| 2 | | 9 | | AcD3C13 | | AV | | AVEEIASR | N.D. | N.I. | 0% | 0% | P22213 | | SLY1_YEAST | control | **Protein SLY1** |  | 1 | 38 | 37 |
| 2 | | 8 | | AcD3C13 | | AV | | AVGKNKR | 100% free | 0% | 0% | 0% | P23248 | | RS3B_YEAST | control,hNaa60p | **40S ribosomal protein S1-B** | P33442 (2-8) | 55 | 46 | 29 |
| 2 | | 9 | | AcD3C13 | | AV | | AVIKKGAR | 100% free | 0% | 0% | 0% | A6ZZJ1 | | MYO3_YEAST | hNaa60p,control | **Myosin-3** | P36006 (2-9) | 2 | 67 | 30 |
| 2 | | 9 | | AcD3C13 | | AV | | AVIPAKKR | N.D. | N.I. | N.I. | N.D. | Q06339 | | TFC6_YEAST | hNaa60p | **Transcription factor tau 91 kDa subunit** |  | 1 | 26 | 24 |
| 2 | | 19 | | AcD3C13 | | AV | | AVKGLGKPDQVYDGSKIR | 100% free | 0% | 0% | 0% | P50861 | | RIB4_YEAST | hNaa60p,control | **6,7-dimethyl-8-ribityllumazine synthase** |  | 5 | 58 | 35 |
| 2 | | 14 | | Ace | | AV | | AVNPELAPFTLSR | N.D. | N.I. | 97% | N.D. | P38042 | | CDC27_YEAST | hNaa60p | **Anaphase-promoting complex subunit CDC27** |  | 1 | 57 | 38 |
| 2 | | 11 | | AcD3C13 | | AV | | AVPGYYELYR | 100% free | 0% | N.I. | N.D. | P32774 | | TOA2_YEAST | control | **Transcription initiation factor IIA small subunit** |  | 1 | 50 | 37 |
| 2 | | 9 | | AcD3C13 | | AV | | AVSKVYAR | 100% free | 1% | 0% | -1% | P00924 | | ENO1_YEAST | hNaa60p,control | **Enolase 1** | P00925 (2-9) | 279 | 76 | 34 |
| 2 | | 15 | | AcD3C13,Ace | | AV | | AVSNNNNNNNSKER | N.D. | N.D. | 28% | N.D. | Q3E795 | | YL361_YEAST | control,hNaa60p | **Uncharacterized protein YLR361C-A** |  | 5 | 75 | 35 |
| 2 | | 10 | | AcD3C13,Ace | | AY | | AYNQEDSKR | partial Ac- | 69% | 65% | -4% | P38337 | | SHG1_YEAST | control,hNaa60p | **COMPASS component SHG1** |  | 4 | 61 | 35 |
| **2. Cys-** | | | | | | | | | | | | | | | | | | | |  |  |
| 2 | 14 | | AcD3C13 | | CE | | CESSNKTENDIVR | | N.D. | N.D. | N.I. | N.D. | P40083 | YEW7_YEAST | | control | **Uncharacterized protein YEL137C** |  | 1 | 59 | 36 |
| 2 | 9 | | AcD3C13 | | CG | | CGIFAAFR | | 100% free | 0% | N.I. | N.D. | P49089 | ASNS1_YEAST | | control | **Asparagine synthetase [glutamine-hydrolyzing] 1** |  | 2 | 73 | 37 |
| 2 | 8 | | AcD3C13 | | CI | | CILMATR | | 100% free | 0% | N.I. | N.D. | P53275 | YG37_YEAST | | control | **Uncharacterized protein YGR127W** |  | 1 | 44 | 36 |
| **3. Gly-** | | | | | | | | | | | | | | | | | | | |  |  |
| 2 | 17 | | AcD3C13 | | GA | | GAKSVTASSSKKIKNR | | 100% free | 0% | 0% | 0% | P53632 | PAP2_YEAST | | hNaa60p,control | **Poly(A) RNA polymerase protein 2** |  | 3 | 45 | 31 |
| 2 | 17 | | AcD3C13 | | GE | | GEEHKPLLDASGVDPR | | 100% free | 0% | 0% | 0% | P25694 | CDC48_YEAST | | control,hNaa60p | **Cell division control protein 48** |  | 3 | 47 | 37 |
| 2 | 29 | | AcD3C13 | | GG | | GGILKNPLALSPEQLAQQDPETLEEFRR | | partial Ac- | 7% | 8% | 1% | P41818 | GLC8_YEAST | | control,hNaa60p | **Protein GLC8** |  | 2 | 53 | 37 |
| 2 | 11 | | AcD3C13 | | GI | | GIQEKTLGIR | | 100% free | 0% | 0% | 0% | Q06616 | YP174_YEAST | | control,hNaa60p | **Nuclear envelope protein YPR174C** |  | 2 | 64 | 34 |
| 2 | 14 | | AcD3C13 | | GI | | GISSKNGPKKMGR | | N.D. | N.I. | 0% | N.D. | P05085 | ARGR2_YEAST | | hNaa60p | **Arginine metabolism regulation protein II** |  | 1 | 38 | 36 |
| 2 | 23 | | AcD3C13 | | GK | | GKDNKEHKESKESKTVDNYEAR | | N.D. | N.I. | 0% | N.D. | P32495 | NHP2_YEAST | | hNaa60p | **H/ACA ribonucleoprotein complex subunit 2** |  | 1 | 48 | 37 |
| 2 | 9 | | AcD3C13 | | GK | | GKFEQKER | | 100% free | 0% | N.I. | N.D. | P38284 | ICS2_YEAST | | control | **Increased copper sensitivity protein 2** |  | 1 | 40 | 38 |
| 2 | 19 | | AcD3C13 | | GK | | GKGAAKYGFKSGVFPTTR | | N.D. | N.D. | 6% | N.D. | P17558 | RTPT_YEAST | | hNaa60p,control | **37S ribosomal protein PET123, mitochondrial** |  | 4 | 55 | 36 |
| 2 | 11 | | AcD3C13 | | GK | | GKGTPSFGKR | | 100% free | 0% | 0% | 0% | P49166 | RL37A_YEAST | | control,hNaa60p | **60S ribosomal protein L37-A** | P51402 (2-11) | 82 | 83 | 36 |
| 2 | 13 | | AcD3C13 | | GK | | GKKNTKGGKKGR | | 100% free | 0% | 0% | 0% | P38912 | IF1A_YEAST | | control,hNaa60p | **Eukaryotic translation initiation factor 1A** |  | 32 | 72 | 27 |
| 2 | 11 | | AcD3C13 | | GK | | GKSAVIFVER | | N.D. | N.I. | N.D. | N.D. | P34162 | MED20_YEAST | | hNaa60p | **Mediator of RNA polymerase II transcription subunit 20** |  | 2 | 75 | 35 |
| 2 | 9 | | AcD3C13 | | GK | | GKSSKDKR | | 100% free | 0% | 0% | 0% | P38238 | TRM7_YEAST | | control,hNaa60p | **tRNA (uridine-2'-O-)-methyltransferase TRM7** |  | 2 | 48 | 34 |
| 2 | 12 | | AcD3C13 | | GK | | GKTQKKNSKGR | | 100% free | 1% | 0% | -1% | P25582 | SPB1_YEAST | | hNaa60p,control | **AdoMet-dependent rRNA methyltransferase SPB1** |  | 2 | 70 | 30 |
| 2 | 40 | | AcD3C13 | | GL | | GLFDKVKQFANSNNNNNDSGNNNQGDYVTKAENMIGEDR | | 100% free | 0% | 0% | 0% | P18899 | DDR48_YEAST | | hNaa60p,control | **Stress protein DDR48** |  | 3 | 103 | 32 |
| 2 | 9 | | AcD3C13 | | GV | | GVEQILKR | | 100% free | 0% | 0% | 0% | P14540 | ALF_YEAST | | hNaa60p,control | **Fructose-bisphosphate aldolase** |  | 101 | 63 | 33 |
| 2 | 8 | | AcD3C13 | | GV | | GVIKKKR | | 100% free | 0% | 0% | 0% | P18888 | SNF6_YEAST | | control,hNaa60p | **Transcription regulatory protein SNF6** |  | 2 | 46 | 20 |
| 2 | 21 | | AcD3C13 | | GV | | GVKQTPPVQVKVSDADSTNR | | N.D. | N.I. | 0% | N.D. | P38306 | YB47_YEAST | | hNaa60p | **Uncharacterized protein YBR197C** |  | 1 | 61 | 37 |
| 2 | 12 | | AcD3C13 | | GV | | GVSANLFVKQR | | N.D. | N.I. | 69% | N.D. | P53063 | DOM3Z_YEAST | | hNaa60p | **RAT1-interacting protein** |  | 1 | 54 | 35 |
| **4. Met-Ala-** | | | | | | | | | | | | | | | | | | | |  |  |
| 1 | 10 | | Ace | | MA | | MAGLKDVVTR | | N.D. | N.I. | 87% | N.D. | P0C2H8 | RL31A_YEAST | | hNaa60p | **60S ribosomal protein L31-A** | P0C2H9 (1-10) | 1 | 72 | 37 |
| 1 | 12 | | Ace | | MA | | MAKQSLDVSSDR | | N.D. | N.I. | 57% | N.D. | P05743 | RL26A_YEAST | | hNaa60p | **60S ribosomal protein L26-A** | P53221 (1-12) | 1 | 36 | 36 |
| 1 | 10 | | Ace | | MA | | MASNEVLVLR | | N.D. | N.I. | 87% | N.D. | P38011 | GBLP_YEAST | | hNaa60p | **Guanine nucleotide-binding protein subunit beta-like protein** |  | 1 | 71 | 37 |
| 1 | 8 | | Ace,AcD3C13 | | MA | | MAVGKNKR | | N.D. | N.I. | 69% | N.D. | P23248 | RS3B_YEAST | | hNaa60p | **40S ribosomal protein S1-B** | P33442 (1-8) | 2 | 51 | 35 |
| 1 | 9 | | Ace,AcD3C13 | | MA | | MAVSKVYAR | | partial Ac- | 2% | 50% | 48% | P00924 | ENO1_YEAST | | control,hNaa60p | **Enolase 1** | P00925 (1-9) | 3 | 68 | 37 |
| **5. Met-Asp- & Met-Glu-** | | | | | | | | | | | | | | | | | | | |  |  |
| 1 | 11 | | Ace | | MD | | MDAFSLKKDNR | | 100% Ac | 100% | N.D. | N.D. | P40022 | YEM4_YEAST | | control,hNaa60p | **Uncharacterized protein YER034W** |  | 37 | 93 | 37 |
| 1 | 11 | | Ace | | MD | | MDAGLSTMATR | | 100% Ac | 100% | 100% | 0% | P39523 | YM11_YEAST | | control,hNaa60p | **Uncharacterized protein YMR124W** |  | 5 | 98 | 31 |
| 1 | 12 | | Ace | | MD | | MDALLTKFNEDR | | N.D. | N.D. | N.D. | N.D. | P46957 | DPOD2_YEAST | | control,hNaa60p | **DNA polymerase delta small subunit** |  | 8 | 106 | 36 |
| 1 | 26 | | Ace | | MD | | MDALNSKEQQEFQKVVEQKQMKDFMR | | N.D. | N.D. | N.D. | N.D. | O74700 | TIM9_YEAST | | hNaa60p,control | **Mitochondrial import inner membrane translocase subunit TIM9** |  | 2 | 48 | 35 |
| 1 | 9 | | Ace | | MD | | MDASEEAKR | | 100% Ac | 100% | 100% | 0% | P17065 | SEC2_YEAST | | hNaa60p,control | **Rab guanine nucleotide exchange factor SEC2** |  | 9 | 86 | 34 |
| 1 | 17 | | Ace | | MD | | MDATTPLLTVANSHPAR | | 100% Ac | 100% | 98% | -2% | Q99385 | VCX1_YEAST | | control,hNaa60p | **Vacuolar calcium ion transporter** |  | 2 | 102 | 37 |
| 1 | 7 | | Ace | | MD | | MDAYSTR | | 100% Ac | 100% | 100% | 0% | P07149 | FAS1_YEAST | | hNaa60p,control | **Fatty acid synthase subunit beta** |  | 7 | 53 | 29 |
| 1 | 16 | | Ace | | MD | | MDDALHALSSLTAKKR | | N.D. | N.I. | 100% | N.D. | P25588 | MRC1_YEAST | | hNaa60p | **Mediator of replication checkpoint protein 1** |  | 1 | 69 | 38 |
| 1 | 15 | | Ace | | MD | | MDDIETAKNLTVKAR | | 100% Ac | 100% | 100% | 0% | P03872 | REP2_YEAST | | hNaa60p,control | **Partitioning protein REP2** |  | 16 | 82 | 37 |
| 1 | 14 | | Ace | | MD | | MDDYHVNTAFSMGR | | N.D. | N.I. | 100% | N.D. | P13185 | KIN1_YEAST | | hNaa60p | **Protein kinase KIN1** |  | 2 | 47 | 26 |
| 1 | 27 | | Ace | | MD | | MDEASHLPNGSLKNMEFTPVGFIKSKR | | partial Ac- | 56% | N.I. | N.D. | P32380 | NUF1_YEAST | | control | **Protein NUF1** |  | 1 | 51 | 37 |
| 1 | 9 | | Ace | | MD | | MDEMDNVIR | | 100% Ac | 100% | 100% | 0% | Q12530 | RMP1_YEAST | | hNaa60p,control | **Ribonuclease MRP protein subunit RMP1** |  | 2 | 67 | 29 |
| 1 | 10 | | Ace | | MD | | MDENEFDNQR | | 100% Ac | 100% | 100% | 0% | P53934 | YNJ2_YEAST | | hNaa60p,control | **UPF0586 protein YNL092W** |  | 2 | 35 | 24 |
| 1 | 13 | | Ace | | MD | | MDESVDPVELLLR | | 100% Ac | 100% | N.I. | N.D. | P21705 | DAL82_YEAST | | control | **Protein DAL82** |  | 1 | 41 | 37 |
| 1 | 9 | | Ace | | MD | | MDEVLPLFR | | 100% Ac | 100% | 100% | 0% | P53079 | COG1_YEAST | | hNaa60p,control | **Conserved oligomeric Golgi complex subunit 1** |  | 2 | 46 | 37 |
| 1 | 15 | | Ace | | MD | | MDEYSSIYSQPKTPR | | 100% Ac | 100% | 100% | 0% | P28708 | KKL6_YEAST | | control,hNaa60p | **Probable serine/threonine-protein kinase YKL116C** |  | 2 | 79 | 35 |
| 1 | 13 | | Ace | | MD | | MDFETNEDINGVR | | 100% Ac | 100% | 100% | 0% | P15303 | SEC23_YEAST | | control,hNaa60p | **Protein transport protein SEC23** |  | 22 | 107 | 29 |
| 1 | 12 | | Ace | | MD | | MDFTAKIKELER | | 100% Ac | 100% | 100% | 0% | P46670 | TBCC_YEAST | | control,hNaa60p | **Tubulin-specific chaperone C** |  | 2 | 98 | 37 |
| 1 | 16 | | Ace | | MD | | MDFTKPETVLNLQNIR | | 100% Ac | 100% | 100% | 0% | P32178 | CHMU_YEAST | | control,hNaa60p | **Chorismate mutase** |  | 6 | 70 | 38 |
| 1 | 15 | | Ace | | MD | | MDFYKLDEKLKELKR | | 100% Ac | 100% | 100% | 0% | P53277 | SYF2_YEAST | | hNaa60p,control | **Pre-mRNA-splicing factor SYF2** |  | 3 | 65 | 37 |
| 1 | 23 | | Ace | | MD | | MDGMFAMPGAAAGAASPQQPKSR | | 100% Ac | 100% | 100% | 0% | P33448 | TOM6_YEAST | | hNaa60p,control | **Mitochondrial import receptor subunit TOM6** |  | 18 | 81 | 32 |
| 1 | 11 | | Ace | | MD | | MDGQIDKMEKR | | 100% Ac | 100% | N.I. | N.D. | P47134 | BIR1_YEAST | | control | **Protein BIR1** |  | 1 | 55 | 36 |
| 1 | 8 | | Ace | | MD | | MDIILGIR | | 100% Ac | 100% | 100% | 0% | P22141 | PSB2_YEAST | | control,hNaa60p | **Proteasome component C11** |  | 23 | 77 | 36 |
| 1 | 6 | | Ace | | MD | | MDILKR | | 100% Ac | 100% | 100% | 0% | Q08986 | SAM3_YEAST | | hNaa60p,control | **S-adenosylmethionine permease SAM3** |  | 5 | 44 | 37 |
| 1 | 18 | | Ace | | MD | | MDINELIIGAQSADKHTR | | N.D. | N.I. | 100% | N.D. | P53067 | IMB5_YEAST | | hNaa60p | **Importin subunit beta-5** |  | 2 | 81 | 37 |
| 1 | 25 | | Ace | | MD | | MDISKPVGSEITSVDFGILTAKEIR | | 100% Ac | 100% | N.I. | N.D. | P10964 | RPA1_YEAST | | control | **DNA-directed RNA polymerase I subunit RPA1** |  | 1 | 39 | 37 |
| 1 | 5 | | Ace | | MD | | MDKIR | | 100% Ac | 100% | 100% | 0% | P17536 |  | | hNaa60p,control | **no description** | P32357 (123-127) | 12 | 38 | 37 |
| 1 | 15 | | Ace | | MD | | MDKKKDLLENEQFLR | | 100% Ac | 99% | 100% | 0% | P53629 | ARE2_YEAST | | control,hNaa60p | **Sterol O-acyltransferase 2** |  | 9 | 91 | 38 |
| 1 | 16 | | Ace | | MD | | MDKNSVNKDSEEKDER | | 100% Ac | 100% | N.D. | N.D. | P36047 | SDS22_YEAST | | hNaa60p,control | **Protein phosphatase 1 regulatory subunit SDS22** |  | 5 | 84 | 33 |
| 1 | 9 | | Ace | | MD | | MDKSMIKKR | | 100% Ac | 100% | 100% | 0% | Q08471 | YO066_YEAST | | control,hNaa60p | **Putative transcriptional activator YOR066W** |  | 2 | 46 | 37 |
| 1 | 7 | | Ace | | MD | | MDKYTNR | | 100% Ac | 100% | 100% | 0% | P53389 | HOL1_YEAST | | hNaa60p,control | **Protein HOL1** |  | 3 | 55 | 34 |
| 2 | 12 | | Ace | | MD | | MDLDKIIASLR | | partial Ac- | 89% | 93% | 4% | P32345 | PP4C_YEAST | | hNaa60p,control | **Serine/threonine-protein phosphatase 4 catalytic subunit** |  | 2 | 79 | 37 |
| 1 | 6 | | Ace | | MD | | MDLFKR | | 100% Ac | 100% | 100% | 0% | P32566 | SMI1_YEAST | | control,hNaa60p | **Cell wall assembly regulator SMI1** |  | 14 | 48 | 36 |
| 1 | 19 | | Ace | | MD | | MDLLAASVESTLKNLQDKR | | N.D. | N.I. | 100% | N.D. | P43573 | BUD27_YEAST | | hNaa60p | **Bud site selection protein 27** |  | 2 | 144 | 37 |
| 1 | 10 | | Ace | | MD | | MDLPKDKSDR | | N.D. | N.I. | 100% | N.D. | Q08553 | SYC1_YEAST | | hNaa60p | **Protein SYC1** |  | 2 | 55 | 37 |
| 1 | 10 | | Ace | | MD | | MDLVNHLTDR | | 100% Ac | 100% | 100% | 0% | P00498 | HIS1_YEAST | | control,hNaa60p | **ATP phosphoribosyltransferase** |  | 2 | 54 | 36 |
| 1 | 23 | | Ace | | MD | | MDMDTQEAELSSQLENLTINSPR | | N.D. | N.I. | N.D. | N.D. | P32336 | NUD1_YEAST | | hNaa60p | **Protein NUD1** |  | 1 | 53 | 31 |
| 1 | 18 | | Ace | | MD | | MDMKVEKLSEDEIALYDR | | N.D. | N.D. | 100% | N.D. | Q06624 | RHC31_YEAST | | control,hNaa60p | **DNA damage tolerance protein RHC31** |  | 4 | 65 | 36 |
| 1 | 18 | | Ace | | MD | | MDNFESTAEGNLSIGNKR | | N.D. | N.D. | 100% | N.D. | Q06104 | YPR09_YEAST | | control,hNaa60p | **Uncharacterized membrane protein YPR109W** |  | 2 | 90 | 34 |
| 1 | 17 | | Ace | | MD | | MDNGTDSSTSKFVPEYR | | 100% Ac | 99% | 100% | 1% | Q02821 | IMA1_YEAST | | control,hNaa60p | **Importin subunit alpha** |  | 3 | 53 | 32 |
| 1 | 18 | | Ace | | MD | | MDNKTPVTLAKVIKVLGR | | 100% Ac | 100% | 100% | 0% | Q3E7X9 | RS28A_YEAST | | control,hNaa60p | **40S ribosomal protein S28-A** |  | 34 | 99 | 32 |
| 1 | 21 | | Ace | | MD | | MDNQQESISEDITGDLAAAVR | | 100% Ac | 100% | 100% | 0% | P46958 | IDS2_YEAST | | control,hNaa60p | **IME2-dependent-signaling protein** |  | 3 | 84 | 33 |
| 1 | 12 | | Ace | | MD | | MDNTTNINTNER | | 100% Ac | 100% | N.I. | N.D. | A6ZMM3 | MSS11_YEAST | | control | **Transcription activator MSS11** | Q03825 (1-12) | 1 | 53 | 31 |
| 1 | 9 | | Ace | | MD | | MDPELQAIR | | 100% Ac | 100% | 100% | 0% | Q04773 | YMW4_YEAST | | control,hNaa60p | **Uncharacterized protein YMR074C** |  | 49 | 87 | 36 |
| 1 | 20 | | Ace | | MD | | MDPHNPIVLDQGTGFVKIGR | | 100% Ac | 100% | 99% | -1% | P32381 | ARP2_YEAST | | hNaa60p,control | **Actin-related protein 2** |  | 7 | 96 | 37 |
| 1 | 25 | | Ace | | MD | | MDPLSSVQPASYVGFDTITNQIEHR | | partial Ac- | 96% | N.D. | N.D. | P25342 | CDC10_YEAST | | control,hNaa60p | **Cell division control protein 10** |  | 2 | 87 | 35 |
| 1 | 13 | | Ace | | MD | | MDPNSNSSSETLR | | N.D. | N.I. | 100% | N.D. | P10870 | SNF3_YEAST | | hNaa60p | **High-affinity glucose transporter SNF3** |  | 1 | 45 | 30 |
| 1 | 14 | | Ace | | MD | | MDQKKDPSNNLTER | | N.D. | N.D. | 100% | N.D. | P38305 | YB44_YEAST | | control,hNaa60p | **Uncharacterized protein YBR194W** |  | 6 | 84 | 37 |
| 1 | 13 | | Ace | | MD | | MDQSVEDLFGALR | | 100% Ac | 100% | 100% | 0% | Q12035 | FCF2_YEAST | | hNaa60p,control | **rRNA-processing protein FCF2** |  | 3 | 100 | 35 |
| 1 | 28 | | Ace | | MD | | MDSEVAALVIDNGSGMCKAGFAGDDAPR | | N.D. | N.D. | 90% | N.D. | P60010 | ACT_YEAST | | control,hNaa60p | **Actin** |  | 43 | 119 | 29 |
| 1 | 12 | | Ace | | MD | | MDSIDEQIAIKR | | 100% Ac | 100% | 100% | 0% | P36162 | DAD2_YEAST | | control,hNaa60p | **DASH complex subunit DAD2** |  | 6 | 93 | 37 |
| 1 | 26 | | Ace | | MD | | MDSIIPAGVKLDDLQVILAKNENETR | | partial Ac- | 94% | N.I. | N.D. | P40356 | MED3_YEAST | | control | **Mediator of RNA polymerase II transcription subunit 3** |  | 2 | 98 | 37 |
| 1 | 10 | | Ace | | MD | | MDSITVKKPR | | 100% Ac | 100% | 100% | 0% | P25611 | YCZ6_YEAST | | control,hNaa60p | **Uncharacterized transcriptional regulatory protein YCR106W** |  | 2 | 60 | 37 |
| 1 | 17 | | Ace | | MD | | MDSIVNVVEDDVKYAQR | | N.D. | N.I. | 100% | N.D. | Q03868 | SPO71_YEAST | | hNaa60p | **Sporulation-specific protein 71** |  | 2 | 41 | 36 |
| 1 | 14 | | Ace | | MD | | MDSKDTQKLLKEHR | | 100% Ac | 100% | 100% | 0% | P43638 | MHP1_YEAST | | control,hNaa60p | **MAP-homologous protein 1** |  | 2 | 58 | 37 |
| 1 | 18 | | Ace | | MD | | MDSKTPVTLAKVIKVLGR | | 100% Ac | 100% | 100% | 0% | P0C0X0 | RS28B_YEAST | | hNaa60p,control | **40S ribosomal protein S28-B** |  | 28 | 74 | 31 |
| 1 | 7 | | Ace | | MD | | MDSLEAR | | 100% Ac | 100% | 100% | 0% | P46963 | CTK3_YEAST | | hNaa60p,control | **CTD kinase subunit gamma** |  | 3 | 42 | 33 |
| 1 | 14 | | Ace | | MD | | MDSQPVDVDNIIDR | | 100% Ac | 100% | 100% | 0% | P32598 | PP12_YEAST | | control,hNaa60p | **Serine/threonine-protein phosphatase PP1-2** |  | 8 | 93 | 34 |
| 1 | 14 | | Ace | | MD | | MDSQVPNNNESLNR | | 100% Ac | 100% | 100% | 0% | Q02457 | TBF1_YEAST | | control,hNaa60p | **Protein TBF1** |  | 2 | 68 | 32 |
| 1 | 11 | | Ace | | MD | | MDSSPNKKTYR | | 100% Ac | 100% | 100% | 0% | P40450 | BNR1_YEAST | | hNaa60p,control | **BNI1-related protein 1** |  | 2 | 64 | 36 |
| 1 | 8 | | Ace | | MD | | MDSVIQKR | | 100% Ac | 100% | 100% | 0% | Q08287 | NOP8_YEAST | | control,hNaa60p | **60S ribosome subunit biogenesis protein NOP8** |  | 5 | 79 | 37 |
| 1 | 20 | | Ace | | MD | | MDSVMVKKEVLENPDHDLKR | | N.D. | N.D. | 100% | N.D. | P53973 | HDA1_YEAST | | control,hNaa60p | **Histone deacetylase HDA1** |  | 5 | 69 | 38 |
| 1 | 28 | | Ace | | MD | | MDSVSNLKSTNFQNNNDPKESVEEAVLR | | N.D. | N.I. | 100% | N.D. | Q12457 | YD026_YEAST | | hNaa60p | **Myb domain-containing protein YDR026C** |  | 1 | 50 | 35 |
| 1 | 19 | | Ace | | MD | | MDSYSITNVKYLDPTELHR | | 100% Ac | 100% | N.D. | N.D. | P42937 | YG4E_YEAST | | control,hNaa60p | **Uncharacterized protein YGR203W** |  | 4 | 90 | 36 |
| 1 | 12 | | Ace | | MD | | MDTEALANYLLR | | 100% Ac | 100% | 100% | 0% | Q2V2P8 | YD74A_YEAST | | hNaa60p,control | **Uncharacterized protein YDR374W-A** |  | 3 | 50 | 36 |
| 1 | 17 | | Ace | | MD | | MDTEEKKKTTASVEHAR | | N.D. | N.I. | 99% | N.D. | Q2V2P1 | YL146_YEAST | | hNaa60p | **Coiled-coil domain-containing protein YLR146W-A** |  | 1 | 46 | 37 |
| 1 | 13 | | Ace | | MD | | MDTLFNSTEKNAR | | 100% Ac | 100% | 100% | 0% | P48363 | PFD3_YEAST | | control,hNaa60p | **Prefoldin subunit 3** |  | 76 | 100 | 35 |
| 1 | 10 | | Ace | | MD | | MDTSKKDTTR | | 100% Ac | 100% | 100% | 0% | Q04779 | RCO1_YEAST | | control,hNaa60p | **Transcriptional regulatory protein RCO1** |  | 4 | 64 | 36 |
| 1 | 16 | | Ace | | MD | | MDTVGTDAAAASINER | | 100% Ac | 100% | 100% | 0% | Q04007 | YDR86_YEAST | | hNaa60p,control | **Uncharacterized protein YDR186C** |  | 4 | 113 | 33 |
| 1 | 8 | | Ace | | MD | | MDTVNNYR | | 100% Ac | 100% | 100% | 0% | P07250 | IPMK_YEAST | | control,hNaa60p | **Inositol polyphosphate multikinase** |  | 2 | 58 | 31 |
| 1 | 11 | | Ace | | MD | | MDTVTVLNELR | | 100% Ac | 100% | 100% | 0% | Q08236 | AVO1_YEAST | | hNaa60p,control | **Target of rapamycin complex 2 subunit AVO1** |  | 3 | 81 | 37 |
| 1 | 11 | | Ace | | MD | | MDVDSDVNVSR | | 100% Ac | 100% | 100% | 0% | P40318 | SSM4_YEAST | | hNaa60p,control | **E3 ubiquitin-protein ligase Doa10** |  | 4 | 120 | 31 |
| 1 | 23 | | Ace | | MD | | MDVEKDVLDVYIKNLENQIGNKR | | N.D. | N.I. | 97% | N.D. | P47167 | MCM22_YEAST | | hNaa60p | **Central kinetochore subunit MCM22** |  | 1 | 61 | 37 |
| 1 | 20 | | Ace | | MD | | MDVLKLGYELDQLSDLVEER | | 100% Ac | 100% | 100% | 0% | P31377 | STX8_YEAST | | control,hNaa60p | **Syntaxin-8** |  | 2 | 76 | 37 |
| 1 | 23 | | Ace | | MD | | MDVVQQKQDKQLQHQTQEQQQIR | | N.D. | N.D. | 100% | N.D. | Q03823 | YM35_YEAST | | hNaa60p,control | **Uncharacterized protein YMR160W** |  | 2 | 83 | 37 |
| 1 | 23 | | Ace | | MD | | MDYKEEQKQELEVLESIYPDELR | | N.D. | N.D. | 100% | N.D. | Q03768 | GIR2_YEAST | | control,hNaa60p | **Protein GIR2** |  | 2 | 74 | 36 |
| 1 | 11 | | Ace | | MD | | MDYLKPAQKGR | | 100% Ac | 100% | 100% | 0% | P32855 | SEC8_YEAST | | hNaa60p,control | **Exocyst complex component SEC8** |  | 2 | 62 | 37 |
| 1 | 11 | | Ace | | MD | | MDYMKLGLKSR | | 100% Ac | 100% | 100% | 0% | P35201 | MIF2_YEAST | | hNaa60p,control | **Protein MIF2** |  | 2 | 64 | 38 |
| 1 | 10 | | Ace | | MD | | MDYPDPDTIR | | 100% Ac | 100% | 100% | 0% | P32829 | MRE11_YEAST | | control,hNaa60p | **Double-strand break repair protein MRE11** |  | 2 | 48 | 31 |
| 1 | 11 | | Ace | | ME | | MEADDHVSLFR | | 100% Ac | 100% | 100% | 0% | Q12016 | VPS68_YEAST | | hNaa60p,control | **Vacuolar protein sorting-associated protein 68** |  | 3 | 62 | 32 |
| 1 | 15 | | Ace | | ME | | MEAHNQFLKTFQKER | | 100% Ac | 100% | 100% | 0% | P49723 | RIR4_YEAST | | control,hNaa60p | **Ribonucleoside-diphosphate reductase small chain 2** |  | 4 | 93 | 37 |
| 1 | 8 | | Ace | | ME | | MEAISQLR | | 100% Ac | 100% | 100% | 0% | Q12150 | CSF1_YEAST | | hNaa60p,control | **Protein CSF1** |  | 2 | 63 | 36 |
| 1 | 18 | | Ace | | ME | | MEDIEGYEPHITQELIDR | | 100% Ac | 100% | N.D. | N.D. | P53296 | ATF2_YEAST | | control,hNaa60p | **Alcohol O-acetyltransferase 2** |  | 2 | 87 | 33 |
| 1 | 7 | | Ace | | ME | | MEDKLDR | | 100% Ac | 100% | 100% | 0% | Q04004 | PLP1_YEAST | | hNaa60p,control | **Phosducin-like protein 1** |  | 2 | 57 | 36 |
| 1 | 22 | | Ace | | ME | | MEEEELSKLLANVKIDPSLTSR | | 100% Ac | 98% | N.I. | N.D. | P40555 | PSMD9_YEAST | | control | **Probable 26S proteasome regulatory subunit p27** |  | 2 | 76 | 37 |
| 1 | 14 | | Ace | | ME | | MEELNIDFDVFKKR | | N.D. | N.I. | 100% | N.D. | P32558 | SPT16_YEAST | | hNaa60p | **FACT complex subunit SPT16** |  | 1 | 41 | 37 |
| 1 | 8 | | Ace | | ME | | MEESKTKR | | 100% Ac | 100% | 100% | 0% | Q06152 | YL271_YEAST | | hNaa60p,control | **Uncharacterized protein YLR271W** |  | 7 | 63 | 37 |
| 1 | 7 | | Ace | | ME | | MEFVAER | | 100% Ac | 100% | 100% | 0% | P06245 | KAPB_YEAST | | control,hNaa60p | **cAMP-dependent protein kinase type 2** |  | 2 | 46 | 34 |
| 1 | 15 | | Ace | | ME | | MEGEQSPQGEKSLQR | | 100% Ac | 99% | 100% | 1% | P38179 | ALG3_YEAST | | hNaa60p,control | **Dolichyl-P-Man:Man(5)GlcNAc(2)-PP-dolichyl mannosyltransferase** |  | 2 | 82 | 35 |
| 1 | 24 | | Ace | | ME | | MEGSTGFDGDATTFFAPDAVFGDR | | 100% Ac | 100% | N.I. | N.D. | P24279 | MCM3_YEAST | | control | **DNA replication licensing factor MCM3** |  | 1 | 43 | 27 |
| 1 | 17 | | Ace | | ME | | MEGSYLSAQENQPIPER | | 100% Ac | 100% | N.I. | N.D. | P33304 | AFR1_YEAST | | control | **Protein AFR1** |  | 1 | 67 | 33 |
| 1 | 18 | | Ace | | ME | | MEGVYFNIDNGFIEGVVR | | 100% Ac | 100% | N.I. | N.D. | P32366 | VA0D_YEAST | | control,hNaa60p | **V-type proton ATPase subunit d** |  | 2 | 56 | 34 |
| 1 | 12 | | Ace | | ME | | MEHVDSDFAPIR | | 100% Ac | 100% | N.I. | N.D. | Q06208 | RIF2_YEAST | | control | **Protein RIF2** |  | 1 | 40 | 33 |
| 1 | 9 | | Ace | | ME | | MEIKEVDDR | | 100% Ac | 100% | 100% | 0% | P38863 | SPC97_YEAST | | hNaa60p,control | **Spindle pole body component SPC97** |  | 5 | 70 | 36 |
| 1 | 11 | | Ace | | ME | | MEIVYKPLDIR | | 100% Ac | 100% | 100% | 0% | Q03503 | MAK3_YEAST | | hNaa60p,control | **N-terminal acetyltransferase C complex catalytic subunit MAK3** |  | 3 | 77 | 38 |
| 1 | 6 | | Ace | | ME | | MEIYIR | | 100% Ac | 100% | 100% | 0% | Q04697 | GSF2_YEAST | | control,hNaa60p | **Glucose-signaling factor 2** |  | 2 | 44 | 36 |
| 1 | 16 | | Ace | | ME | | MEKALELDGEYPESLR | | 100% Ac | 100% | 100% | 0% | Q05979 | KYNU_YEAST | | control,hNaa60p | **Kynureninase** |  | 2 | 75 | 36 |
| 1 | 9 | | Ace | | ME | | MEKDALEVR | | 100% Ac | 100% | 100% | 0% | Q03760 | PML39_YEAST | | hNaa60p,control | **Pre-mRNA leakage protein 39** |  | 2 | 71 | 37 |
| 1 | 16 | | Ace | | ME | | MEKESVYNLALKCAER | | N.D. | N.I. | 100% | N.D. | P53114 | MED5_YEAST | | hNaa60p | **Mediator of RNA polymerase II transcription subunit 5** |  | 2 | 59 | 37 |
| 1 | 17 | | Ace | | ME | | MEKSIAKGLSDKLYEKR | | 100% Ac | 100% | 100% | 0% | Q06708 | VAC14_YEAST | | hNaa60p,control | **Vacuole morphology and inheritance protein 14** |  | 4 | 69 | 37 |
| 1 | 9 | | Ace | | ME | | MELDECLER | | N.D. | N.I. | 60% | N.D. | P32838 | PP2A4_YEAST | | hNaa60p | **Serine/threonine-protein phosphatase PP2A-like PPG1** |  | 1 | 48 | 29 |
| 1 | 19 | | Ace | | ME | | MELILNSLISDDLTEEQKR | | N.D. | N.I. | 100% | N.D. | Q04632 | COG8_YEAST | | hNaa60p | **Conserved oligomeric Golgi complex subunit 8** |  | 3 | 87 | 38 |
| 1 | 9 | | Ace | | ME | | MELLPQGQR | | 100% Ac | 100% | 100% | 0% | P53900 | PFD4_YEAST | | hNaa60p,control | **Prefoldin subunit 4** |  | 35 | 87 | 36 |
| 1 | 19 | | Ace | | ME | | MELPSINSTTSISDNQELR | | 100% Ac | 100% | N.I. | N.D. | Q06251 | YL177_YEAST | | control | **Uncharacterized protein YLR177W** |  | 1 | 58 | 35 |
| 1 | 11 | | Ace | | ME | | MELSPTYQTER | | 100% Ac | 100% | 100% | 0% | P52891 | NUP84_YEAST | | control,hNaa60p | **Nucleoporin NUP84** |  | 3 | 61 | 34 |
| 1 | 15 | | Ace | | ME | | MENDKGQLVELYVPR | | 100% Ac | 100% | 100% | 0% | P0C0V8 | RS21A_YEAST | | hNaa60p,control | **40S ribosomal protein S21-A** | Q3E754 (1-15) | 66 | 85 | 37 |
| 1 | 17 | | Ace | | ME | | MENITQPTQQSTQATQR | | 100% Ac | 100% | 100% | 0% | P22216 | RAD53_YEAST | | control,hNaa60p | **Serine/threonine-protein kinase RAD53** |  | 7 | 102 | 34 |
| 1 | 25 | | Ace | | ME | | MEPESIGDVGNHAQDDSASIVSGPR | | N.D. | N.I. | 94% | N.D. | P18410 | SPO7_YEAST | | hNaa60p | **Sporulation-specific protein SPO7** |  | 1 | 35 | 32 |
| 1 | 9 | | Ace | | ME | | MEQINSNSR | | 100% Ac | 100% | 100% | 0% | Q12482 | AGC1_YEAST | | hNaa60p,control | **Mitochondrial aspartate-glutamate transporter AGC1** |  | 2 | 57 | 33 |
| 1 | 8 | | Ace | | ME | | MEQNAEKR | | 100% Ac | 100% | 100% | 0% | A7A1S5 | DUS3_YEAST | | hNaa60p,control | **tRNA-dihydrouridine synthase 3** | Q06053 (1-8) | 6 | 53 | 35 |
| 1 | 10 | | Ace | | ME | | MEQNIISTIR | | partial Ac- | 97% | 99% | 2% | P32571 | UBP4_YEAST | | control,hNaa60p | **Ubiquitin carboxyl-terminal hydrolase 4** |  | 2 | 72 | 37 |
| 1 | 10 | | Ace | | ME | | MESFENLSIR | | 100% Ac | 100% | 100% | 0% | P38770 | BRL1_YEAST | | hNaa60p,control | **Nucleus export protein BRL1** |  | 2 | 72 | 34 |
| 1 | 8 | | Ace | | ME | | MESNKQPR | | 100% Ac | 100% | 100% | 0% | P40035 | PIC2_YEAST | | control,hNaa60p | **Mitochondrial phosphate carrier protein 2** |  | 6 | 55 | 36 |
| 1 | 9 | | Ace | | ME | | METIDIQNR | | 100% Ac | 100% | 100% | 0% | P38713 | OSH3_YEAST | | control,hNaa60p | **Oxysterol-binding protein homolog 3** |  | 5 | 74 | 35 |
| 1 | 12 | | Ace | | ME | | METIDSKQNINR | | 100% Ac | 100% | 100% | 0% | A6ZLH6 | PRP5_YEAST | | control,hNaa60p | **Pre-mRNA-processing ATP-dependent RNA helicase PRP5** | P21372 (1-12) | 2 | 76 | 36 |
| 1 | 15 | | Ace | | ME | | METPLDLLKLNLDER | | partial Ac- | 92% | N.D. | N.D. | P57743 | LSM3_YEAST | | hNaa60p,control | **U6 snRNA-associated Sm-like protein LSm3** |  | 35 | 96 | 37 |
| 1 | 15 | | Ace | | ME | | METSNFVKQLSSNNR | | 100% Ac | 100% | 100% | 0% | P35178 | RRP1_YEAST | | hNaa60p,control | **Ribosomal RNA-processing protein 1** |  | 9 | 99 | 36 |
| 1 | 26 | | Ace | | ME | | MEVAPALSTTQSDVAFQKVETHEIDR | | N.D. | N.I. | N.D. | N.D. | P32461 | DPH2_YEAST | | hNaa60p | **Diphthamide biosynthesis protein 2** |  | 1 | 58 | 36 |
| 1 | 7 | | Ace | | ME | | MEVDNKR | | 100% Ac | 100% | 100% | 0% | P43589 | SAD1_YEAST | | hNaa60p,control | **Pre-mRNA-splicing factor SAD1** |  | 2 | 44 | 36 |
| 1 | 8 | | Ace | | ME | | MEVLKNIR | | 100% Ac | 100% | 100% | 0% | Q12318 | PSY3_YEAST | | hNaa60p,control | **Platinum sensitivity protein 3** |  | 3 | 76 | 37 |
| **6. Met-Phe-** | | | | | | | | | | | | | | | | | | | |  |  |
| 1 | 12 | | Ace | | MF | | MFDGFSNNKGKR | | 100% Ac | 100% | 100% | 0% | Q03780 | YD239_YEAST | | control,hNaa60p | **Uncharacterized protein YDR239C** |  | 6 | 76 | 36 |
| 1 | 11 | | Ace | | MF | | MFEGFGPNKKR | | 100% Ac | 100% | 100% | 0% | P40348 | RFC2_YEAST | | hNaa60p,control | **Replication factor C subunit 2** |  | 3 | 59 | 37 |
| 1 | 9 | | AcD3C13 | | MF | | MFEIKLNDR | | N.D. | N.I. | 0% | N.D. | P32341 | VPH2_YEAST | | hNaa60p | **Vacuolar ATPase assembly integral membrane protein VPH2** |  | 2 | 43 | 37 |
| 1 | 15 | | Ace,AcD3C13 | | MF | | MFESVNLDENSPEDR | | 100% free | 0% | 45% | 45% | P40971 | LYS14_YEAST | | control,hNaa60p | **Lysine biosynthesis regulatory protein LYS14** |  | 3 | 84 | 29 |
| 2 | 16 | | Ace | | MF | | MFGKKKNNGGSSTAR | | 100% Ac | 100% | 100% | 0% | P21951 | DPOE_YEAST | | hNaa60p,control | **DNA polymerase epsilon catalytic subunit A** |  | 3 | 59 | 38 |
| 1 | 15 | | Ace,AcD3C13 | | MF | | MFGLPQQEVSEEEKR | | 100% free | 0% | 88% | 88% | P80967 | TOM5_YEAST | | hNaa60p,control | **Mitochondrial import receptor subunit TOM5** |  | 11 | 88 | 35 |
| 1 | 6 | | Ace | | MF | | MFGSKR | | N.D. | N.I. | 100% | N.D. | Q12038 | SNI1_YEAST | | hNaa60p | **Protein SNI1** |  | 1 | 38 | 36 |
| 1 | 13 | | Ace | | MF | | MFGVKDAIFKIKR | | N.D. | N.I. | 100% | N.D. | Q08952 | OXR1_YEAST | | hNaa60p | **Oxidation resistance protein 1** |  | 1 | 53 | 35 |
| 1 | 11 | | AcD3C13 | | MF | | MFIKNDHAGDR | | 100% free | 0% | N.I. | N.D. | P14843 | AROF_YEAST | | control | **Phospho-2-dehydro-3-deoxyheptonate aldolase, phenylalanine-inhibited** |  | 1 | 51 | 36 |
| 1 | 7 | | Ace | | MF | | MFKKFTR | | 100% Ac | 100% | 97% | -3% | P89886 | TMA20_YEAST | | control,hNaa60p | **Translation machinery-associated protein 20** |  | 28 | 54 | 36 |
| 1 | 22 | | Ace | | MF | | MFKSKTSTLSYDETPNSNEGDR | | N.D. | N.I. | 100% | N.D. | P32330 | YKM1_YEAST | | hNaa60p | **WD repeat-containing protein YKL121W** |  | 1 | 42 | 33 |
| 1 | 7 | | AcD3C13 | | MF | | MFSFVQR | | 100% free | 0% | 30% | 30% | Q12133 | SPC3_YEAST | | hNaa60p,control | **Signal peptidase complex subunit SPC3** |  | 2 | 55 | 36 |
| 1 | 23 | | Ace | | MF | | MFTGQEYHSVDSNSNKQKDNNKR | | 100% Ac | 100% | N.I. | N.D. | P40535 | ACA2_YEAST | | control | **ATF/CREB activator 2** |  | 1 | 73 | 34 |
| 1 | 17 | | Ace | | MF | | MFVSPPPATSKNQVLQR | | 100% Ac | 100% | N.I. | N.D. | P35198 | MTH1_YEAST | | control | **Protein MTH1** |  | 1 | 52 | 37 |
| **7. Met-Gly-** | | | | | | | | | | | | | | | | | | | |  |  |
| 1 | 12 | | AcD3C13 | | MG | | MGAYKYLEELQR | | partial Ac- | 35% | 32% | -3% | P05748 | RL15A_YEAST | | control,hNaa60p | **60S ribosomal protein L15-A** |  | 2 | 47 | 37 |
| 1 | 11 | | Ace,AcD3C13 | | MG | | MGKGTPSFGKR | | partial Ac- | 4% | 15% | 11% | P49166 | RL37A_YEAST | | hNaa60p,control | **60S ribosomal protein L37-A** | P51402 (1-11) | 3 | 55 | 37 |
| 1 | 17 | | Ace | | MG | | MGTEDAIALPNSTLEPR | | N.D. | N.I. | 81% | N.D. | Q12382 | HSD1_YEAST | | hNaa60p | **Protein HSD1** |  | 1 | 87 | 36 |
| 1 | 9 | | Ace,AcD3C13 | | MG | | MGVEQILKR | | partial Ac- | 13% | 17% | 4% | P14540 | ALF_YEAST | | control,hNaa60p | **Fructose-bisphosphate aldolase** |  | 16 | 82 | 36 |
| **8. Met-His-** | | | | | | | | | | | | | | | | | | | |  |  |
| 1 | 13 | | AcD3C13 | | MH | | MHLMYTLGPDGKR | | N.D. | N.D. | 18% | N.D. | Q6Q547 | NOP10_YEAST | | hNaa60p,control | **H/ACA ribonucleoprotein complex subunit 3** |  | 2 | 69 | 37 |
| **9. Met-Ile-** | | | | | | | | | | | | | | | | | | | |  |  |
| 1 | 11 | | Ace,AcD3C13 | | MI | | MIALPVEKAPR | | 100% free | 0% | 78% | 78% | P27614 | CBPS_YEAST | | hNaa60p,control | **Carboxypeptidase S** |  | 2 | 94 | 36 |
| 1 | 8 | | Ace | | MI | | MIAPDSQR | | partial Ac- | 24% | N.D. | N.D. | P32909 | SMY2_YEAST | | hNaa60p,control | **Protein SMY2** |  | 3 | 41 | 35 |
| 1 | 9 | | AcD3C13 | | MI | | MIASEIFER | | partial Ac- | 14% | 33% | 20% | P32488 | MBR3_YEAST | | control,hNaa60p | **Protein MBR3** |  | 2 | 46 | 37 |
| 1 | 7 | | AcD3C13,Ace | | MI | | MIEKDKR | | N.D. | N.I. | 80% | N.D. | P43636 | ALG2_YEAST | | hNaa60p | **Alpha-1,3-mannosyltransferase ALG2** |  | 2 | 50 | 37 |
| 1 | 16 | | AcD3C13,Ace | | MI | | MIETAIYGKTVDDQSR | | partial Ac- | 7% | N.D. | N.D. | P36078 | HOT13_YEAST | | hNaa60p,control | **Helper of Tim protein 13** |  | 5 | 116 | 36 |
| 1 | 9 | | AcD3C13 | | MI | | MIEVVVNDR | | 100% free | 0% | 0% | 0% | Q6Q546 | HUB1_YEAST | | hNaa60p,control | **Ubiquitin-like modifier HUB1** |  | 79 | 84 | 37 |
| 1 | 11 | | AcD3C13 | | MI | | MIFSLDEELHR | | 100% free | 0% | 47% | 47% | P38070 | KBN8_YEAST | | hNaa60p,control | **Probable serine/threonine-protein kinase YBR028C** |  | 2 | 63 | 37 |
| 1 | 11 | | AcD3C13 | | MI | | MIIFVSEEPER | | N.D. | N.I. | 0% | N.D. | Q12271 | INP53_YEAST | | hNaa60p | **Inositol-1,4,5-trisphosphate 5-phosphatase 3** |  | 1 | 70 | 37 |
| 1 | 9 | | AcD3C13 | | MI | | MIKSTLIYR | | 100% free | 0% | 4% | 4% | P22214 | SEC22_YEAST | | hNaa60p,control | **Protein transport protein SEC22** |  | 4 | 71 | 35 |
| 1 | 9 | | Ace,AcD3C13 | | MI | | MINESVSKR | | partial Ac- | 2% | 70% | 68% | P47050 | CUL8_YEAST | | hNaa60p,control | **Cullin-8** |  | 4 | 73 | 37 |
| 1 | 8 | | AcD3C13 | | MI | | MINEYVAR | | 100% free | 0% | N.D. | N.D. | P40080 | YEV8_YEAST | | hNaa60p,control | **Uncharacterized protein YER128W** |  | 2 | 45 | 36 |
| 1 | 10 | | Ace | | MI | | MIPALTPEER | | N.D. | N.I. | 77% | N.D. | Q3E7Y6 | YH199_YEAST | | hNaa60p | **Uncharacterized protein YHR199C-A** |  | 1 | 37 | 37 |
| 1 | 12 | | AcD3C13,Ace | | MI | | MIPAPLDASLLR | | N.D. | N.I. | 36% | N.D. | P25361 | YCT3_YEAST | | hNaa60p | **Uncharacterized protein YCR043C** |  | 2 | 54 | 36 |
| 1 | 7 | | AcD3C13 | | MI | | MIPSNKR | | N.D. | N.I. | 17% | N.D. | Q12438 | GLRX6_YEAST | | hNaa60p | **Monothiol glutaredoxin-6** |  | 1 | 46 | 37 |
| 1 | 9 | | Ace | | MI | | MIQNSAGYR | | N.D. | N.I. | 100% | N.D. | P35688 | LRG1_YEAST | | hNaa60p | **Rho-GTPase-activating protein LRG1** |  | 1 | 53 | 35 |
| 1 | 11 | | Ace | | MI | | MIQTQSTAIKR | | 100% Ac | 100% | 100% | 0% | P22215 | SLY41_YEAST | | hNaa60p,control | **Uncharacterized transporter SLY41** |  | 4 | 80 | 37 |
| 1 | 11 | | AcD3C13 | | MI | | MISGVLVYSSR | | N.D. | N.I. | 0% | N.D. | Q99186 | AP2M_YEAST | | hNaa60p | **AP-2 complex subunit mu** |  | 1 | 46 | 37 |
| 1 | 8 | | Ace | | MI | | MISPSKKR | | partial Ac- | 85% | 82% | -3% | Q08981 | ACM1_YEAST | | hNaa60p,control | **APC/C-CDH1 modulator 1** |  | 2 | 51 | 36 |
| 1 | 10 | | AcD3C13 | | MI | | MISTTISGKR | | partial Ac- | 41% | N.I. | N.D. | Q12749 | SMC6_YEAST | | control | **Structural maintenance of chromosomes protein 6** |  | 1 | 56 | 36 |
| 1 | 8 | | AcD3C13 | | MI | | MITGKELR | | 100% free | 0% | 0% | 0% | P20107 | ZRC1_YEAST | | control,hNaa60p | **Zinc/cadmium resistance protein** |  | 2 | 63 | 36 |
| 1 | 10 | | AcD3C13,Ace | | MI | | MIVDYEKDPR | | 100% free | 0% | 36% | 36% | Q12347 | HRT3_YEAST | | control,hNaa60p | **F-box protein HRT3** |  | 3 | 59 | 36 |
| 1 | 15 | | AcD3C13 | | MI | | MIVPTYGDVLDASNR | | N.D. | N.D. | 22% | N.D. | P36007 | SRY1_YEAST | | control,hNaa60p | **Threo-3-hydroxyaspartate ammonia-lyase** |  | 3 | 77 | 37 |
| **10. Met-Lys-** | | | | | | | | | | | | | | | | | | | |  |  |
| 1 | 19 | | AcD3C13 | | MK | | MKAIDKMTDNPPQEGLSGR | | 100% free | 0% | 32% | 32% | P27882 | ERV1_YEAST | | control,hNaa60p | **Mitochondrial FAD-linked sulfhydryl oxidase ERV1** |  | 9 | 73 | 37 |
| 1 | 14 | | AcD3C13,Ace | | MK | | MKAKPLSQDPGSKR | | 100% free | 0% | N.D. | N.D. | A6ZXD2 | TRMB_YEAST | | hNaa60p,control | **tRNA (guanine-N(7)-)-methyltransferase** | B3LH81 (1-14)^AQ12009 (1-14) | 10 | 79 | 36 |
| 1 | 12 | | AcD3C13,Ace | | MK | | MKANGLDNDPAR | | 100% free | 0% | N.D. | N.D. | P53925 | YNL5_YEAST | | control,hNaa60p | **Uncharacterized vacuolar membrane protein YNL115C** |  | 9 | 93 | 36 |
| 1 | 11 | | AcD3C13,Ace | | MK | | MKDLQKKSSVR | | 100% free | 0% | 18% | 18% | Q02831 | YP077_YEAST | | control,hNaa60p | **Uncharacterized protein YPL077C** |  | 4 | 70 | 35 |
| 1 | 5 | | Ace | | MK | | MKDLR | | 100% Ac | 100% | N.I. | N.D. | Q06644 |  | | control | **no description** |  | 2 | 38 | 37 |
| 1 | 19 | | Ace | | MK | | MKEELSKVSSMQNFEMIQR | | N.D. | N.I. | 67% | N.D. | Q08760 | RAX1_YEAST | | hNaa60p | **Bud site selection protein RAX1** |  | 1 | 55 | 36 |
| 1 | 12 | | AcD3C13 | | MK | | MKEVVVSETPKR | | 100% free | 0% | 0% | 0% | P04051 | RPC1_YEAST | | hNaa60p,control | **DNA-directed RNA polymerase III subunit RPC1** |  | 6 | 80 | 36 |
| 1 | 10 | | AcD3C13 | | MK | | MKFGKYLEAR | | 100% free | 0% | N.D. | N.D. | P17442 | PHO81_YEAST | | control,hNaa60p | **Phosphate system positive regulatory protein PHO81** |  | 2 | 68 | 37 |
| 1 | 11 | | AcD3C13 | | MK | | MKFIDELDIER | | 100% free | 0% | N.I. | N.D. | P41910 | MAF1_YEAST | | control | **Repressor of RNA polymerase III transcription MAF1** |  | 1 | 47 | 37 |
| 1 | 13 | | AcD3C13 | | MK | | MKGLILVGGYGTR | | 100% free | 0% | 0% | 0% | P41940 | MPG1_YEAST | | control,hNaa60p | **Mannose-1-phosphate guanyltransferase** |  | 71 | 130 | 36 |
| 1 | 16 | | AcD3C13,Ace | | MK | | MKGTGGVVVGTQNPVR | | 100% free | 0% | 82% | 82% | Q08954 | YP199_YEAST | | control,hNaa60p | **Smr domain-containing protein YPL199C** |  | 11 | 106 | 37 |
| 1 | 20 | | AcD3C13 | | MK | | MKHPYEEFPTGSKSPYNMSR | | N.D. | N.I. | 36% | N.D. | Q08831 | VTS1_YEAST | | hNaa60p | **Protein VTS1** |  | 1 | 57 | 34 |
| 1 | 8 | | AcD3C13 | | MK | | MKIKTIKR | | 100% free | 0% | 0% | 0% | P33750 | SOF1_YEAST | | control,hNaa60p | **Protein SOF1** |  | 7 | 68 | 26 |
| 1 | 11 | | AcD3C13 | | MK | | MKILLSKQQTR | | 100% free | 0% | 0% | 0% | P50942 | INP52_YEAST | | hNaa60p,control | **Inositol-1,4,5-trisphosphate 5-phosphatase 2** |  | 2 | 67 | 33 |
| 1 | 13 | | AcD3C13 | | MK | | MKILTQDEIEAHR | | 100% free | 0% | 0% | 0% | P53721 | AIM38_YEAST | | hNaa60p,control | **Altered inheritance rate of mitochondria protein 38** |  | 2 | 81 | 37 |
| 1 | 13 | | AcD3C13 | | MK | | MKIQTNAVNVLQR | | 100% free | 0% | 1% | 1% | A6ZVI4 | RT25_YEAST | | hNaa60p,control | **37S ribosomal protein S25, mitochondrial** | P40496 (1-13) | 5 | 102 | 36 |
| 1 | 11 | | AcD3C13 | | MK | | MKITEKLEQHR | | N.D. | N.I. | 0% | N.D. | P53128 | MTHR2_YEAST | | hNaa60p | **Methylenetetrahydrofolate reductase 2** |  | 1 | 39 | 37 |
| 2 | 12 | | AcD3C13 | | MK | | MKKKPKCQIAR | | 100% free | 0% | N.I. | N.D. | A6ZZE3 | LOT5_YEAST | | control | **Protein LOT5** | P34234 (2-12) | 1 | 38 | 33 |
| 1 | 9 | | AcD3C13,Ace | | MK | | MKKTFEQFR | | 100% free | 0% | 24% | 24% | P25576 | YCE7_YEAST | | control,hNaa60p | **UPF0647 protein YCL047C** |  | 3 | 63 | 37 |
| 1 | 12 | | AcD3C13 | | MK | | MKLDIKKTFSNR | | 100% free | 0% | 0% | 0% | P41811 | COPB2_YEAST | | control,hNaa60p | **Coatomer subunit beta'** |  | 24 | 91 | 35 |
| 1 | 13 | | AcD3C13 | | MK | | MKLDSGIYSEAQR | | 100% free | 0% | N.I. | N.D. | P50088 | YG4Z_YEAST | | control | **Uncharacterized protein YGR236C** |  | 1 | 77 | 37 |
| 1 | 8 | | AcD3C13 | | MK | | MKLKSVFR | | 100% free | 0% | 0% | 0% | P38244 | YBS4_YEAST | | hNaa60p,control | **Uncharacterized zinc metalloprotease YBR074W** |  | 2 | 68 | 34 |
| 1 | 11 | | AcD3C13 | | MK | | MKLLKDLLVDR | | 100% free | 0% | 0% | 0% | Q12308 | TFC8_YEAST | | hNaa60p,control | **Transcription factor tau 60 kDa subunit** |  | 4 | 79 | 33 |
| 1 | 13 | | AcD3C13 | | MK | | MKLNEQIPKDLLR | | 100% free | 0% | N.I. | N.D. | Q06199 | YL456_YEAST | | control | **Pyridoxamine 5'-phosphate oxidase YLR456W homolog** |  | 1 | 53 | 35 |
| 1 | 23 | | AcD3C13 | | MK | | MKLNISYPVNGSQKTFEIDDEHR | | 100% free | 0% | 0% | 0% | P02365 | RS6_YEAST | | hNaa60p,control | **40S ribosomal protein S6** |  | 72 | 106 | 36 |
| 1 | 22 | | AcD3C13 | | MK | | MKLPVAQYSAPDGVEKSFAPIR | | 100% free | 0% | 8% | 8% | P46984 | GON7_YEAST | | control,hNaa60p | **Protein GON7** |  | 3 | 92 | 37 |
| 1 | 26 | | AcD3C13 | | MK | | MKLPVAQYSAPDGVEKSFAPIRDDPR | | 100% free | 0% | 7% | 7% | P46984 | GON7_YEAST | | control,hNaa60p | **Protein GON7** |  | 2 | 74 | 37 |
| 1 | 11 | | AcD3C13 | | MK | | MKLVNFLKKLR | | N.D. | N.I. | N.I. | N.D. | Q02260 | SMD1_YEAST | | hNaa60p | **Small nuclear ribonucleoprotein Sm D1** |  | 1 | 28 | 26 |
| 1 | 13 | | AcD3C13 | | MK | | MKMLTKFESKSTR | | 100% free | 0% | N.D. | N.D. | P53622 | COPA_YEAST | | control,hNaa60p | **Coatomer subunit alpha** |  | 8 | 77 | 37 |
| 1 | 14 | | AcD3C13,Ace | | MK | | MKNDNKLQKEALMR | | 100% free | 0% | N.D. | N.D. | Q05568 | PEX10_YEAST | | control,hNaa60p | **Peroxisome assembly protein 10** |  | 2 | 56 | 38 |
| 1 | 18 | | Ace,AcD3C13 | | MK | | MKNLTTIKQTNKNVKQER | | partial Ac- | 8% | 60% | 52% | Q08282 | LCMT2_YEAST | | hNaa60p,control | **Leucine carboxyl methyltransferase 2** |  | 2 | 69 | 35 |
| 1 | 10 | | AcD3C13,Ace | | MK | | MKNSTAASSR | | N.D. | N.I. | 72% | N.D. | P30606 | ITR2_YEAST | | hNaa60p | **Myo-inositol transporter 2** |  | 2 | 57 | 36 |
| 1 | 9 | | AcD3C13,Ace | | MK | | MKPPLNMSR | | 100% free | 0% | 57% | 57% | Q12407 | YD199_YEAST | | control,hNaa60p | **Putative metabolite transport protein YDL199C** |  | 2 | 41 | 37 |
| 1 | 7 | | AcD3C13 | | MK | | MKQIVKR | | 100% free | 0% | 0% | 0% | Q12284 | ERV2_YEAST | | control,hNaa60p | **FAD-linked sulfhydryl oxidase ERV2** |  | 4 | 53 | 31 |
| 1 | 13 | | AcD3C13 | | MK | | MKQKKFNSKKSNR | | N.D. | N.I. | 4% | N.D. | P07272 | PPR1_YEAST | | hNaa60p | **Pyrimidine pathway regulatory protein 1** |  | 2 | 73 | 34 |
| 1 | 7 | | AcD3C13,Ace | | MK | | MKSFITR | | partial Ac- | 15% | 16% | 1% | A6ZRW3 | TOM70_YEAST | | hNaa60p,control | **Mitochondrial import receptor subunit TOM70** | P07213 (1-7) | 10 | 60 | 37 |
| 1 | 8 | | Ace | | MK | | MKSIFKVR | | 100% Ac | 98% | 100% | 2% | P32787 | MG101_YEAST | | control,hNaa60p | **Mitochondrial genome maintenance protein MGM101** |  | 2 | 57 | 35 |
| 1 | 13 | | AcD3C13 | | MK | | MKSSIPITEVLPR | | 100% free | 0% | 16% | 16% | Q3E841 | YN034_YEAST | | control,hNaa60p | **Uncharacterized protein YNR034W-A** |  | 27 | 88 | 36 |
| 1 | 14 | | Ace,AcD3C13 | | MK | | MKSTFKSEYPFEKR | | partial Ac- | 13% | N.D. | N.D. | A6ZKM4 | ATG8_YEAST | | control,hNaa60p | **Autophagy-related protein 8** | P38182 (1-14) | 8 | 101 | 37 |
| 1 | 15 | | AcD3C13 | | MK | | MKTIIISDFDETITR | | 100% free | 0% | 9% | 9% | P25616 | YCQ5_YEAST | | hNaa60p,control | **UPF0655 protein YCR015C** |  | 2 | 80 | 37 |
| 1 | 10 | | Ace | | MK | | MKTNKKISKR | | partial Ac- | 93% | 91% | -2% | Q12334 | SCM3_YEAST | | control,hNaa60p | **Protein SCM3** |  | 8 | 66 | 33 |
| 1 | 9 | | AcD3C13,Ace | | MK | | MKVDLPESR | | 100% free | 0% | 35% | 35% | P47048 | YJE9_YEAST | | hNaa60p,control | **Uncharacterized protein YJL049W** |  | 3 | 52 | 37 |
| 1 | 17 | | AcD3C13 | | MK | | MKVDYEQLCKLYDDTCR | | N.D. | N.I. | 0% | N.D. | P38628 | AGM1_YEAST | | hNaa60p | **Phosphoacetylglucosamine mutase** |  | 2 | 68 | 34 |
| 1 | 17 | | AcD3C13 | | MK | | MKVEIDSFSGAKIYPGR | | 100% free | 0% | 0% | 0% | P04449 | RL24A_YEAST | | hNaa60p,control | **60S ribosomal protein L24-A** |  | 39 | 95 | 38 |
| 1 | 15 | | AcD3C13 | | MK | | MKVEKSSKGLEVLVR | | 100% free | 0% | 0% | 0% | P47173 | YJ9J_YEAST | | control,hNaa60p | **Uncharacterized protein YJR142W** |  | 5 | 85 | 33 |
| 1 | 17 | | AcD3C13 | | MK | | MKVEVDSFSGAKIYPGR | | 100% free | 0% | 0% | 0% | P24000 | RL24B_YEAST | | control,hNaa60p | **60S ribosomal protein L24-B** |  | 68 | 101 | 37 |
| 1 | 9 | | AcD3C13 | | MK | | MKVGGIEDR | | 100% free | 0% | 10% | 10% | Q06549 | CDD_YEAST | | control,hNaa60p | **Cytidine deaminase** |  | 4 | 68 | 37 |
| 1 | 11 | | AcD3C13 | | MK | | MKVGIIMGSVR | | 100% free | 0% | 0% | 0% | Q07923 | LOT6_YEAST | | hNaa60p,control | **NAD(P)H-dependent FMN reductase LOT6** |  | 2 | 98 | 37 |
| 1 | 8 | | AcD3C13 | | MK | | MKVKKSTR | | 100% free | 0% | 0% | 0% | P33200 | PDR3_YEAST | | control,hNaa60p | **Transcription factor PDR3** |  | 2 | 55 | 32 |
| 1 | 7 | | Ace,AcD3C13 | | MK | | MKVLEER | | 100% free | 0% | 12% | 12% | P47076 | RPC9_YEAST | | control,hNaa60p | **DNA-directed RNA polymerase III subunit RPC9** |  | 16 | 56 | 37 |
| 1 | 9 | | Ace,AcD3C13 | | MK | | MKVQITNSR | | 100% free | 0% | 15% | 15% | P13574 | STE12_YEAST | | control,hNaa60p | **Protein STE12** |  | 3 | 71 | 37 |
| 1 | 13 | | AcD3C13 | | MK | | MKVVKEFSVCGGR | | 100% free | 0% | 0% | 0% | P40363 | SFGH_YEAST | | hNaa60p,control | **S-formylglutathione hydrolase** |  | 4 | 83 | 38 |
| 1 | 14 | | AcD3C13 | | MK | | MKYTQYPNSSKLKR | | 100% free | 0% | N.I. | N.D. | P38852 | LIN1_YEAST | | control | **Protein LIN1** |  | 1 | 71 | 37 |
| **11. Met-Leu-** | | | | | | | | | | | | | | | | | | | |  |  |
| 1 | 10 | | Ace | | ML | | MLAAKNILNR | | 100% Ac | 100% | 100% | 0% | P12398 | HSP77_YEAST | | hNaa60p,control | **Heat shock protein SSC1, mitochondrial** |  | 10 | 77 | 37 |
| 1 | 8 | | Ace | | ML | | MLAASFKR | | N.D. | N.I. | 99% | N.D. | P16387 | ODPA_YEAST | | hNaa60p | **Pyruvate dehydrogenase E1 component subunit alpha, mitochondrial** |  | 1 | 55 | 37 |
| 1 | 7 | | AcD3C13 | | ML | | MLAEKTR | | 100% free | 0% | 5% | 5% | P39676 | FHP_YEAST | | hNaa60p,control | **Flavohemoprotein** |  | 35 | 59 | 36 |
| 1 | 10 | | AcD3C13 | | ML | | MLALADNILR | | 100% free | 0% | 0% | 0% | Q12207 | NCE2_YEAST | | hNaa60p,control | **Non-classical export protein 2** |  | 2 | 76 | 37 |
| 1 | 11 | | Ace | | ML | | MLCAIKSTGYR | | N.D. | N.I. | 100% | N.D. | P38345 | FMP21_YEAST | | hNaa60p | **Protein FMP21, mitochondrial** |  | 1 | 61 | 37 |
| 1 | 11 | | Ace,AcD3C13 | | ML | | MLCAISGKVPR | | partial Ac- | 70% | 66% | -4% | P32523 | PRP19_YEAST | | control,hNaa60p | **Pre-mRNA-splicing factor 19** |  | 5 | 74 | 37 |
| 1 | 8 | | Ace | | ML | | MLCQQMIR | | N.D. | N.I. | 84% | N.D. | P04039 | COX8_YEAST | | hNaa60p | **Cytochrome c oxidase polypeptide VIII, mitochondrial** |  | 1 | 39 | 33 |
| 1 | 19 | | AcD3C13 | | ML | | MLDINQFIEDKGGNPELIR | | 100% free | 0% | 0% | 0% | P07284 | SYSC_YEAST | | control,hNaa60p | **Seryl-tRNA synthetase, cytoplasmic** |  | 16 | 124 | 37 |
| 1 | 14 | | AcD3C13 | | ML | | MLEAKFEEASLFKR | | 100% free | 0% | 0% | 0% | P15873 | PCNA_YEAST | | hNaa60p,control | **Proliferating cell nuclear antigen** |  | 9 | 94 | 38 |
| 1 | 7 | | AcD3C13 | | ML | | MLEALFR | | 100% free | 0% | N.I. | N.D. | P38349 | UBX7_YEAST | | control | **UBX domain-containing protein 7** |  | 1 | 55 | 37 |
| 1 | 17 | | Ace,AcD3C13 | | ML | | MLEEGNNVYEIQDLEKR | | 100% free | 0% | 78% | 78% | P17064 | FCY2_YEAST | | hNaa60p,control | **Purine-cytosine permease FCY2** |  | 6 | 115 | 36 |
| 1 | 12 | | AcD3C13 | | ML | | MLESLAANLLNR | | 100% free | 0% | 0% | 0% | Q07878 | VPS13_YEAST | | control,hNaa60p | **Vacuolar protein sorting-associated protein 13** |  | 3 | 82 | 37 |
| 1 | 6 | | AcD3C13 | | ML | | MLETLR | | 100% free | 0% | 13% | 13% | P34077 | NIC96_YEAST | | control,hNaa60p | **Nucleoporin NIC96** |  | 2 | 51 | 36 |
| 1 | 8 | | AcD3C13 | | ML | | MLFDNKNR | | N.D. | N.I. | 38% | N.D. | P33327 | DHE2_YEAST | | hNaa60p | **NAD-specific glutamate dehydrogenase** |  | 1 | 54 | 37 |
| 1 | 7 | | Ace,AcD3C13 | | ML | | MLFNINR | | 100% free | 0% | 48% | 48% | P53278 | YG3A_YEAST | | hNaa60p,control | **Uncharacterized protein YGR130C** |  | 3 | 59 | 36 |
| 1 | 7 | | Ace | | ML | | MLGNLLR | | N.D. | N.I. | 89% | N.D. | A6ZZE9 | LST4_YEAST | | hNaa60p | **Protein LST4** | P34239 (1-7) | 1 | 43 | 37 |
| 1 | 9 | | AcD3C13 | | ML | | MLIENTNDR | | 100% free | 0% | 0% | 0% | P40009 | YND1_YEAST | | hNaa60p,control | **Golgi apyrase** |  | 4 | 56 | 36 |
| 1 | 7 | | Ace,AcD3C13 | | ML | | MLKDLVR | | 100% free | 0% | 15% | 15% | P47133 | YJ58_YEAST | | control,hNaa60p | **Uncharacterized protein YJR088C** |  | 5 | 58 | 35 |
| 1 | 8 | | AcD3C13 | | ML | | MLKEPSVR | | N.D. | N.I. | 6% | N.D. | Q04749 | AVO2_YEAST | | hNaa60p | **Target of rapamycin complex 2 subunit AVO2** |  | 1 | 46 | 37 |
| 1 | 10 | | Ace | | ML | | MLKNFKLSKR | | 100% Ac | 100% | N.I. | N.D. | P29366 | BEM1_YEAST | | control | **Bud emergence protein 1** |  | 1 | 66 | 34 |
| 1 | 8 | | AcD3C13,Ace | | ML | | MLKSLKSR | | 100% free | 0% | 24% | 24% | P40549 | MNT3_YEAST | | hNaa60p,control | **Alpha-1,3-mannosyltransferase MNT3** |  | 3 | 55 | 33 |
| 1 | 8 | | Ace | | ML | | MLKYLVQR | | N.D. | N.I. | N.I. | N.D. | P53193 | JAC1_YEAST | | hNaa60p | **J-type co-chaperone JAC1, mitochondrial** |  | 1 | 40 | 37 |
| 1 | 14 | | AcD3C13 | | ML | | MLLLALSDAHIPDR | | 100% free | 0% | 0% | 0% | P38759 | PEP11_YEAST | | hNaa60p,control | **Vacuolar protein sorting-associated protein 29** |  | 2 | 70 | 37 |
| 1 | 11 | | AcD3C13 | | ML | | MLLPLTKLKPR | | 100% free | 0% | N.I. | N.D. | P40012 | PPOX_YEAST | | control | **Protoporphyrinogen oxidase** |  | 1 | 38 | 28 |
| 1 | 12 | | AcD3C13 | | ML | | MLLSAPVNSTVR | | 100% Ac | 100% | N.I. | N.D. | Q04930 | CRF1_YEAST | | control | **Transcription factor CRF1** |  | 1 | 49 | 37 |
| 1 | 6 | | AcD3C13 | | ML | | MLLTKR | | N.D. | N.I. | 0% | N.D. | P38069 | TTP1_YEAST | | hNaa60p | **Protein TTP1** |  | 1 | 41 | 33 |
| 1 | 10 | | AcD3C13,Ace | | ML | | MLLTPAKTTR | | partial Ac- | 45% | 81% | 37% | P53222 | YG1O_YEAST | | hNaa60p,control | **Uncharacterized protein YGR035C** |  | 3 | 54 | 36 |
| 1 | 8 | | Ace,AcD3C13 | | ML | | MLMPKEDR | | 100% free | 0% | N.D. | N.D. | Q08745 | RS10A_YEAST | | control,hNaa60p | **40S ribosomal protein S10-A** |  | 72 | 63 | 35 |
| 1 | 8 | | AcD3C13,Ace | | ML | | MLMPKQER | | 100% free | 0% | 15% | 15% | P46784 | RS10B_YEAST | | control,hNaa60p | **40S ribosomal protein S10-B** |  | 58 | 63 | 36 |
| 1 | 9 | | Ace | | ML | | MLNFTGQTR | | 100% Ac | 100% | 100% | 0% | P53119 | HUL5_YEAST | | control,hNaa60p | **Probable E3 ubiquitin-protein ligase HUL5** |  | 3 | 52 | 36 |
| 1 | 12 | | AcD3C13 | | ML | | MLNILVLGNGAR | | 100% free | 0% | 0% | 0% | P07244 | PUR2_YEAST | | hNaa60p,control | **Bifunctional purine biosynthetic protein ADE5,7** |  | 6 | 111 | 36 |
| 1 | 7 | | Ace | | ML | | MLPTFKR | | 100% Ac | 100% | N.I. | N.D. | P38071 | ETR1_YEAST | | control | **Enoyl-[acyl-carrier protein] reductase [NADPH, B-specific], mitochondrial** |  | 1 | 38 | 37 |
| 1 | 12 | | Ace,AcD3C13 | | ML | | MLQDNNGPAVKR | | 100% free | 0% | N.D. | N.D. | Q12383 | TRM13_YEAST | | control,hNaa60p | **tRNA guanosine-2'-O-methyltransferase TRM13** |  | 3 | 68 | 37 |
| 1 | 9 | | Ace | | ML | | MLQQGSSSR | | 100% Ac | 100% | N.I. | N.D. | P40095 | YEY8_YEAST | | control | **Uncharacterized protein YER158C** |  | 1 | 46 | 34 |
| 1 | 10 | | Ace | | ML | | MLSATKQTFR | | 100% Ac | 100% | 100% | 0% | P41921 | GSHR_YEAST | | control,hNaa60p | **Glutathione reductase** |  | 5 | 74 | 37 |
| 1 | 11 | | AcD3C13 | | ML | | MLSDCLLNNFR | | N.D. | N.I. | 46% | N.D. | Q12505 | SKS1_YEAST | | hNaa60p | **Serine/threonine-protein kinase SKS1** |  | 1 | 38 | 36 |
| 1 | 23 | | AcD3C13 | | ML | | MLSSEDFGSSGKKETSPDSISIR | | N.D. | N.D. | N.I. | N.D. | P38084 | BAP2_YEAST | | control | **Leu/Val/Ile amino-acid permease** |  | 1 | 47 | 36 |
| 1 | 10 | | Ace | | ML | | MLTSILIKGR | | N.D. | N.I. | 100% | N.D. | P15424 | MS116_YEAST | | hNaa60p | **ATP-dependent RNA helicase MSS116, mitochondrial** |  | 1 | 64 | 35 |
| 1 | 12 | | Ace | | ML | | MLVFGPNSSFVR | | 100% Ac | 100% | 100% | 0% | P33749 | MSN4_YEAST | | hNaa60p,control | **Zinc finger protein MSN4** |  | 4 | 67 | 37 |
| 1 | 8 | | AcD3C13 | | ML | | MLVKTISR | | 100% free | 0% | 0% | 0% | Q12245 | POC4_YEAST | | hNaa60p,control | **Proteasome chaperone 4** |  | 2 | 70 | 34 |
| 1 | 10 | | AcD3C13 | | ML | | MLYELIGLVR | | 100% free | 0% | 0% | 0% | P28778 | RT06_YEAST | | hNaa60p,control | **37S ribosomal protein MRP17, mitochondrial** |  | 2 | 46 | 35 |
| **12. Met-Met-** | | | | | | | | | | | | | | | | | | | |  |  |
| 1 | 17 | | AcD3C13,Ace | | MM | | MMAKNNKTTEAKMSKKR | | partial Ac- | 32% | 88% | 55% | Q04660 | ERB1_YEAST | | control,hNaa60p | **Eukaryotic ribosome biogenesis protein 1** |  | 6 | 59 | 38 |
| 1 | 24 | | Ace,AcD3C13 | | MM | | MMASTSNDEEKLISTTDKYFIEQR | | partial Ac- | 24% | 92% | 68% | Q12248 | DAD1_YEAST | | hNaa60p,control | **DASH complex subunit DAD1** |  | 3 | 52 | 34 |
| 1 | 12 | | AcD3C13 | | MM | | MMDLDKIIASLR | | N.D. | N.I. | 0% | N.D. | P32345 | PP4C_YEAST | | hNaa60p | **Serine/threonine-protein phosphatase 4 catalytic subunit** |  | 2 | 62 | 37 |
| 1 | 12 | | Ace,AcD3C13 | | MM | | MMKKKPKCQIAR | | partial Ac- | 42% | 69% | 27% | A6ZZE3 | LOT5_YEAST | | hNaa60p,control | **Protein LOT5** | P34234 (1-12) | 5 | 49 | 36 |
| 1 | 13 | | Ace,AcD3C13 | | MM | | MMNNNESEAENQR | | N.D. | N.D. | 93% | N.D. | P38165 | RTG3_YEAST | | hNaa60p,control | **Retrograde regulation protein 3** |  | 8 | 58 | 22 |
| 1 | 17 | | Ace | | MM | | MMNNNGNQVSNLSNALR | | partial Ac- | 70% | N.D. | N.D. | P23202 | URE2_YEAST | | control,hNaa60p | **Protein URE2** |  | 7 | 75 | 32 |
| 1 | 10 | | Ace,AcD3C13 | | MM | | MMSDLTPIFR | | 100% free | 0% | 46% | 46% | P41834 | UFE1_YEAST | | hNaa60p,control | **Syntaxin UFE1** |  | 2 | 55 | 35 |
| 1 | 11 | | Ace | | MM | | MMSFSKNATPR | | N.D. | N.I. | 100% | N.D. | P23255 | TAF2_YEAST | | hNaa60p | **Transcription initiation factor TFIID subunit 2** |  | 1 | 44 | 34 |
| 1 | 11 | | Ace | | MM | | MMSNVANASQR | | 100% Ac | 100% | 100% | 0% | P32831 | NGR1_YEAST | | hNaa60p,control | **Negative growth regulatory protein NGR1** |  | 10 | 82 | 32 |
| **13. Met-Asn-** | | | | | | | | | | | | | | | | | | | |  |  |
| 1 | 10 | | Ace | | MN | | MNDQTQFTER | | 100% Ac | 100% | 100% | 0% | P31539 | HS104_YEAST | | control,hNaa60p | **Heat shock protein 104** |  | 17 | 81 | 30 |
| 1 | 11 | | AcD3C13 | | MN | | MNDTLSSFLNR | | partial Ac- | 17% | N.I. | N.D. | P25619 | HSP30_YEAST | | control | **30 kDa heat shock protein** |  | 1 | 49 | 36 |
| 1 | 17 | | Ace | | MN | | MNDYHLEDTTSELEALR | | 100% Ac | 100% | 100% | 0% | P38820 | UBA4_YEAST | | hNaa60p,control | **E1-like URM1 activator protein** |  | 2 | 91 | 32 |
| 1 | 14 | | Ace | | MN | | MNEADVTKFVNNAR | | 100% Ac | 100% | 100% | 0% | A7A1U4 | ATG17_YEAST | | control,hNaa60p | **Autophagy-related protein 17** | Q06410 (1-14) | 2 | 66 | 36 |
| 1 | 9 | | Ace | | MN | | MNEDLFYDR | | partial Ac- | 94% | 100% | 6% | P47163 | YJ99_YEAST | | hNaa60p,control | **Uncharacterized protein YJR129C** |  | 2 | 67 | 28 |
| 1 | 14 | | Ace | | MN | | MNEDLPKEYFELIR | | 100% Ac | 100% | 100% | 0% | P14736 | RAD4_YEAST | | hNaa60p,control | **DNA repair protein RAD4** |  | 2 | 61 | 36 |
| 1 | 34 | | Ace | | MN | | MNESENSPQHNEVTVPMVEDTSSNADIPMEQIQR | | N.D. | N.D. | N.D. | N.D. | Q03323 | SDC1_YEAST | | control,hNaa60p | **COMPASS component SDC1** |  | 2 | 49 | 26 |
| 1 | 13 | | Ace | | MN | | MNETTTKQPLKKR | | 100% Ac | 100% | N.D. | N.D. | P40480 | HOS4_YEAST | | hNaa60p,control | **Protein HOS4** |  | 6 | 82 | 37 |
| 1 | 10 | | Ace | | MN | | MNEVPTTPVR | | 100% Ac | 100% | 100% | 0% | Q08581 | SLK19_YEAST | | control,hNaa60p | **Kinetochore protein SLK19** |  | 4 | 79 | 36 |
| 1 | 14 | | Ace | | MN | | MNFDAVADQQMTDR | | 100% Ac | 100% | 100% | 0% | P32789 | ALK2_YEAST | | control,hNaa60p | **Serine/threonine-protein kinase Haspin homolog ALK2** |  | 2 | 57 | 28 |
| 1 | 21 | | Ace | | MN | | MNFSLSKQSSEKQSSYTDKSR | | N.D. | N.I. | 100% | N.D. | Q12145 | YP013_YEAST | | hNaa60p | **Zinc finger protein YPR013C** |  | 1 | 73 | 36 |
| 1 | 10 | | Ace | | MN | | MNGIQVDINR | | 100% Ac | 100% | 100% | 0% | P38624 | PSB6_YEAST | | control,hNaa60p | **Proteasome component PRE3** |  | 2 | 68 | 36 |
| 1 | 12 | | Ace | | MN | | MNGKEVSSGSGR | | partial Ac- | 90% | 95% | 5% | P36062 | AVT3_YEAST | | hNaa60p,control | **Vacuolar amino acid transporter 3** |  | 5 | 92 | 35 |
| 1 | 18 | | Ace | | MN | | MNGLVLGATGLCGGGFLR | | 100% Ac | 100% | 100% | 0% | P40008 | FMP52_YEAST | | hNaa60p,control | **Protein FMP52, mitochondrial** |  | 3 | 65 | 36 |
| 1 | 11 | | Ace | | MN | | MNGPPTFTQYR | | 100% Ac | 100% | 100% | 0% | P40522 | YIF6_YEAST | | control,hNaa60p | **Uncharacterized protein YIL056W** |  | 2 | 74 | 34 |
| 1 | 6 | | Ace,AcD3C13 | | MN | | MNIKDR | | partial Ac- | 70% | 80% | 10% | Q01590 | SED5_YEAST | | control,hNaa60p | **Integral membrane protein SED5** |  | 4 | 51 | 37 |
| 1 | 6 | | Ace | | MN | | MNIPQR | | 100% Ac | 100% | N.I. | N.D. | P22082 | SNF2_YEAST | | control | **Transcription regulatory protein SNF2** | P38856 (539-544) | 1 | 43 | 36 |
| 1 | 11 | | Ace | | MN | | MNISGTLNTLR | | N.D. | N.I. | 100% | N.D. | P38812 | YHQ0_YEAST | | hNaa60p | **UPF0660 protein YHR100C, mitochondrial** |  | 1 | 84 | 37 |
| 1 | 8 | | Ace | | MN | | MNIVKLQR | | 100% Ac | 100% | 100% | 0% | P32599 | FIMB_YEAST | | hNaa60p,control | **Fimbrin** |  | 66 | 70 | 36 |
| 1 | 12 | | Ace | | MN | | MNKDQAEKYQER | | 100% Ac | 100% | 100% | 0% | P40993 | RMRP_YEAST | | control,hNaa60p | **Ribonuclease MRP protein subunit SNM1** |  | 2 | 86 | 35 |
| 1 | 20 | | Ace | | MN | | MNKEELLGFLLDDSIDSQKR | | 100% Ac | 99% | 100% | 1% | P36103 | YKC3_YEAST | | control,hNaa60p | **Uncharacterized protein YKL023W** |  | 11 | 122 | 37 |
| 1 | 23 | | Ace | | MN | | MNKEEQEDPQQEQISTVQENDPR | | N.D. | N.D. | 100% | N.D. | Q03373 | DIG2_YEAST | | control,hNaa60p | **Down-regulator of invasive growth 2** |  | 2 | 74 | 31 |
| 1 | 22 | | Ace | | MN | | MNKFDEFIESNEKDLDVDTSTR | | N.D. | N.I. | N.D. | N.D. | P38854 | YHV9_YEAST | | hNaa60p | **Uncharacterized protein YHR159W** |  | 1 | 50 | 33 |
| 1 | 16 | | Ace | | MN | | MNKTENLSIEETNEIR | | 100% Ac | 100% | 100% | 0% | Q12420 | SNU66_YEAST | | hNaa60p,control | **66 kDa U4/U6.U5 small nuclear ribonucleoprotein component** |  | 6 | 115 | 36 |
| 1 | 10 | | Ace | | MN | | MNLDLGSTVR | | 100% Ac | 100% | 100% | 0% | P41695 | BUB1_YEAST | | control,hNaa60p | **Checkpoint serine/threonine-protein kinase BUB1** |  | 2 | 76 | 36 |
| 1 | 7 | | Ace | | MN | | MNLLETR | | 100% Ac | 100% | N.I. | N.D. | P19736 | PRP9_YEAST | | control | **Pre-mRNA-splicing factor PRP9** |  | 1 | 51 | 36 |
| 1 | 7 | | Ace | | MN | | MNLLIDR | | 100% Ac | 100% | N.I. | N.D. | Q99332 | HPH1_YEAST | | control | **Protein HPH1** |  | 1 | 67 | 37 |
| 1 | 20 | | Ace | | MN | | MNNAANTGTTNESNVSDAPR | | 100% Ac | 100% | 100% | 0% | P10961 | HSF_YEAST | | hNaa60p,control | **Heat shock factor protein** |  | 10 | 118 | 28 |
| 2 | 17 | | Ace | | MN | | MNNNGNQVSNLSNALR | | 100% Ac | 100% | 100% | 0% | P23202 | URE2_YEAST | | hNaa60p,control | **Protein URE2** |  | 2 | 47 | 35 |
| 1 | 12 | | Ace | | MN | | MNNNNVTEATSR | | 100% Ac | 100% | 100% | 0% | Q03327 | UGO1_YEAST | | control,hNaa60p | **Protein UGO1** |  | 6 | 99 | 31 |
| 1 | 10 | | Ace | | MN | | MNNNVEELLR | | 100% Ac | 100% | 100% | 0% | P42939 | MVB12_YEAST | | hNaa60p,control | **Multivesicular body sorting factor 12** |  | 6 | 83 | 36 |
| 1 | 21 | | Ace | | MN | | MNNSEDPFQQVVKDTKEQLNR | | 100% Ac | 100% | N.I. | N.D. | Q03322 | TLG1_YEAST | | control | **T-SNARE affecting a late Golgi compartment protein 1** |  | 1 | 50 | 36 |
| 1 | 12 | | Ace | | MN | | MNNSIITDDEVR | | 100% Ac | 100% | 100% | 0% | P53110 | YGP9_YEAST | | hNaa60p,control | **Uncharacterized protein YGL159W** |  | 2 | 75 | 34 |
| 1 | 9 | | Ace | | MN | | MNQEEAIFR | | 100% Ac | 100% | 100% | 0% | P37296 | STV1_YEAST | | control,hNaa60p | **V-type proton ATPase subunit a, Golgi isoform** |  | 3 | 78 | 34 |
| 1 | 9 | | Ace | | MN | | MNQILNAQR | | 100% Ac | 100% | 100% | 0% | P47148 | YJ81_YEAST | | hNaa60p,control | **Uncharacterized protein YJR111C** |  | 10 | 75 | 36 |
| 1 | 12 | | Ace | | MN | | MNQLGALAQVSR | | 100% Ac | 100% | 100% | 0% | P53633 | PRA1_YEAST | | hNaa60p,control | **Prenylated Rab acceptor 1** |  | 34 | 101 | 37 |
| 1 | 23 | | Ace | | MN | | MNSDLEYLEDGFDPNSMKVATLR | | 100% Ac | 100% | 100% | 0% | Q03707 | SRC1_YEAST | | control,hNaa60p | **Protein SRC1** |  | 5 | 80 | 33 |
| 1 | 12 | | Ace | | MN | | MNSDTNAFKDIR | | N.D. | N.I. | 100% | N.D. | P36097 | TTI1_YEAST | | hNaa60p | **TEL2-interacting protein 1** |  | 1 | 50 | 35 |
| 1 | 7 | | Ace | | MN | | MNSILDR | | 100% Ac | 100% | 100% | 0% | P33122 | TYE7_YEAST | | control,hNaa60p | **Serine-rich protein TYE7** |  | 2 | 58 | 36 |
| 1 | 21 | | Ace | | MN | | MNSLANNNKLSTEDEEIHSAR | | N.D. | N.D. | 93% | N.D. | P17106 | CBF1_YEAST | | control,hNaa60p | **Centromere-binding protein 1** |  | 7 | 101 | 33 |
| 1 | 15 | | Ace | | MN | | MNSLVTQYAAPLFER | | N.D. | N.I. | 93% | N.D. | P25358 | ELO2_YEAST | | hNaa60p | **Elongation of fatty acids protein 2** |  | 2 | 61 | 37 |
| 1 | 11 | | Ace | | MN | | MNSNEDIHEER | | 100% Ac | 100% | 100% | 0% | Q04322 | YM52_YEAST | | control,hNaa60p | **TBC domain-containing protein YMR192W** |  | 2 | 71 | 26 |
| 1 | 9 | | Ace | | MN | | MNSNELDLR | | 100% Ac | 100% | 100% | 0% | P53046 | ROM1_YEAST | | control,hNaa60p | **RHO1 GDP-GTP exchange protein 1** |  | 3 | 64 | 34 |
| 1 | 25 | | Ace | | MN | | MNSQGYDESSSSTAATSGPTSGDPR | | 100% Ac | 100% | 100% | 0% | P53933 | APP1_YEAST | | control,hNaa60p | **Actin patch protein 1** |  | 3 | 51 | 24 |
| 1 | 8 | | Ace | | MN | | MNSSYTQR | | 100% Ac | 100% | 100% | 0% | Q07471 | THI3_YEAST | | hNaa60p,control | **Thiamine metabolism regulatory protein THI3** |  | 2 | 39 | 29 |
| 1 | 25 | | Ace | | MN | | MNSTPDLISPQKSNSSNSYELESGR | | N.D. | N.I. | 100% | N.D. | P32465 | HXT1_YEAST | | hNaa60p | **Low-affinity glucose transporter HXT1** |  | 1 | 61 | 33 |
| 1 | 6 | | Ace | | MN | | MNSVKR | | 100% Ac | 100% | N.I. | N.D. | P40093 | YEY6_YEAST | | control | **UPF0160 protein YER156C** |  | 1 | 43 | 37 |
| 1 | 9 | | Ace | | MN | | MNSYSLLTR | | N.D. | N.I. | 90% | N.D. | Q02486 | ABF2_YEAST | | hNaa60p | **ARS-binding factor 2, mitochondrial** |  | 2 | 41 | 35 |
| 1 | 17 | | Ace | | MN | | MNTEGASLSEQLLDAAR | | 100% Ac | 100% | 100% | 0% | P53066 | YGZ2_YEAST | | hNaa60p,control | **Ankyrin repeat-containing protein YGL242C** |  | 61 | 129 | 35 |
| 1 | 8 | | Ace | | MN | | MNTLLFKR | | 100% Ac | 100% | 100% | 0% | Q00246 | RHO4_YEAST | | control,hNaa60p | **GTP-binding protein RHO4** |  | 4 | 68 | 37 |
| 1 | 7 | | Ace | | MN | | MNVLLKR | | N.D. | N.I. | 100% | N.D. | P32048 | SYKM_YEAST | | hNaa60p | **Lysyl-tRNA synthetase, mitochondrial** |  | 1 | 46 | 35 |
| 1 | 7 | | Ace,AcD3C13 | | MN | | MNVPKAR | | partial Ac- | 85% | 84% | -1% | P53305 | RT27_YEAST | | control,hNaa60p | **Mitochondral 37S ribosomal protein S27** |  | 5 | 53 | 37 |
| 1 | 11 | | Ace | | MN | | MNVSTSTFQTR | | 100% Ac | 100% | 100% | 0% | P20433 | RPB4_YEAST | | control,hNaa60p | **DNA-directed RNA polymerase II subunit RPB4** |  | 16 | 93 | 34 |
| 1 | 11 | | Ace | | MN | | MNVTTPEVAFR | | N.D. | N.D. | 100% | N.D. | P50874 | ORC5_YEAST | | control,hNaa60p | **Origin recognition complex subunit 5** |  | 2 | 56 | 36 |
| 1 | 15 | | Ace | | MN | | MNVTVTVYDKNVKYR | | 100% Ac | 100% | 100% | 0% | Q12421 | CIS1_YEAST | | hNaa60p,control | **Protein CIS1** |  | 2 | 102 | 38 |
| 1 | 10 | | Ace | | MN | | MNYNCEIQNR | | 100% Ac | 100% | 100% | 0% | P41817 | CUP9_YEAST | | control,hNaa60p | **Homeobox protein CUP9** |  | 2 | 53 | 28 |
| 1 | 14 | | Ace | | MN | | MNYNLSKYPDDVSR | | 100% Ac | 99% | 100% | 1% | Q00916 | RU17_YEAST | | hNaa60p,control | **U1 small nuclear ribonucleoprotein 70 kDa homolog** |  | 2 | 84 | 34 |
| 1 | 19 | | Ace | | MN | | MNYSADSGNTVYVGNIDPR | | 100% Ac | 100% | N.I. | N.D. | Q99181 | HSH49_YEAST | | control | **Protein HSH49** |  | 1 | 55 | 32 |
| **14. Met-Pro-** | | | | | | | | | | | | | | | | | | | |  |  |
| 1 | 14 | | AcD3C13 | | MP | | MPEQAQQGEQSVKR | | N.D. | N.I. | 0% | N.D. | P40467 | YIN0_YEAST | | hNaa60p | **Uncharacterized transcriptional regulatory protein YIL130W** |  | 1 | 46 | 37 |
| 1 | 8 | | AcD3C13 | | MP | | MPKLVLVR | | N.D. | N.I. | 0% | N.D. | P00950 | PMG1_YEAST | | hNaa60p | **Phosphoglycerate mutase 1** |  | 1 | 54 | 32 |
| **15. Met-Gln-** | | | | | | | | | | | | | | | | | | | |  |  |
| 1 | 14 | | Ace | | MQ | | MQAPVVFMNASQER | | 100% Ac | 100% | 100% | 0% | P39077 | TCPG_YEAST | | control,hNaa60p | **T-complex protein 1 subunit gamma** |  | 40 | 96 | 34 |
| 1 | 14 | | Ace | | MQ | | MQAQGSQSNVGSLR | | N.D. | N.I. | 98% | N.D. | P38227 | YBP3_YEAST | | hNaa60p | **Uncharacterized transporter YBR043C** |  | 1 | 76 | 35 |
| 1 | 8 | | AcD3C13,Ace | | MQ | | MQEGGFIR | | partial Ac- | 30% | 44% | 14% | P31244 | RAD16_YEAST | | control,hNaa60p | **DNA repair protein RAD16** |  | 3 | 57 | 35 |
| 1 | 9 | | Ace,AcD3C13 | | MQ | | MQGNKSTIR | | partial Ac- | 18% | 96% | 77% | P01119 | RAS1_YEAST | | hNaa60p,control | **Ras-like protein 1** |  | 4 | 92 | 37 |
| 1 | 15 | | Ace,AcD3C13 | | MQ | | MQIPKYENKPFKPPR | | N.D. | N.I. | 53% | N.D. | P38086 | RDH54_YEAST | | hNaa60p | **DNA repair and recombination protein RDH54** |  | 2 | 48 | 37 |
| 1 | 7 | | AcD3C13 | | MQ | | MQKGNIR | | partial Ac- | 20% | N.D. | N.D. | P28273 | YKV5_YEAST | | control,hNaa60p | **Uncharacterized protein YKL215C** |  | 3 | 52 | 36 |
| 1 | 6 | | Ace | | MQ | | MQKSVR | | 100% Ac | 100% | N.I. | N.D. | Q04233 | GIS4_YEAST | | control | **Protein GIS4** |  | 1 | 38 | 37 |
| 1 | 8 | | Ace | | MQ | | MQKTDGIR | | partial Ac- | 92% | 97% | 5% | Q06132 | SGD1_YEAST | | hNaa60p,control | **Suppressor of glycerol defect protein 1** |  | 4 | 59 | 37 |
| 1 | 10 | | AcD3C13,Ace | | MQ | | MQLVPLELNR | | 100% free | 0% | 21% | 21% | Q12010 | YO092_YEAST | | control,hNaa60p | **Uncharacterized membrane protein YOL092W** |  | 3 | 57 | 37 |
| 1 | 20 | | Ace | | MQ | | MQNAQIKSSSKGSGIDGTDR | | partial Ac- | 87% | 100% | 13% | P39734 | HPH2_YEAST | | control,hNaa60p | **Protein HPH2** |  | 3 | 55 | 37 |
| 1 | 13 | | Ace | | MQ | | MQNSQDYFYAQNR | | 100% Ac | 100% | 100% | 0% | Q04964 | SML1_YEAST | | hNaa60p,control | **Ribonucleotide reductase inhibitor protein SML1** |  | 15 | 103 | 27 |
| 1 | 9 | | Ace | | MQ | | MQSMNVQPR | | 100% Ac | 100% | 100% | 0% | P39008 | POP2_YEAST | | hNaa60p,control | **Protein POP2** |  | 4 | 56 | 32 |
| 1 | 13 | | Ace,AcD3C13 | | MQ | | MQSQDSCYGVAFR | | partial Ac- | 51% | 71% | 19% | P16861 | K6PF1_YEAST | | hNaa60p,control | **6-phosphofructokinase subunit alpha** |  | 4 | 69 | 28 |
| 1 | 9 | | AcD3C13 | | MQ | | MQSSLPLCR | | partial Ac- | 33% | 48% | 15% | Q02202 | ECM9_YEAST | | control,hNaa60p | **Protein ECM9** |  | 2 | 42 | 36 |
| 1 | 9 | | AcD3C13 | | MQ | | MQVKSIKMR | | 100% free | 0% | 0% | 0% | Q05584 | GLO2_YEAST | | control,hNaa60p | **Hydroxyacylglutathione hydrolase, cytoplasmic isozyme** |  | 69 | 64 | 36 |
| 1 | 8 | | Ace | | MQ | | MQVQKMVR | | N.D. | N.I. | 83% | N.D. | P25370 | GFD2_YEAST | | hNaa60p | **Good for full DBP5 activity protein 2** |  | 1 | 38 | 37 |
| **16. Met-Ser-** | | | | | | | | | | | | | | | | | | | |  |  |
| 1 | 10 | | Ace | | MS | | MSAKAQNPMR | | N.D. | N.I. | N.D. | N.D. | P0C0W9 | RL11A_YEAST | | hNaa60p | **60S ribosomal protein L11-A** |  | 1 | 50 | 35 |
| 1 | 23 | | Ace | | MS | | MSANEFYSSGQQGQYNQQNNQER | | N.D. | N.I. | 90% | N.D. | P40159 | YNU8_YEAST | | hNaa60p | **Uncharacterized protein YNL208W** |  | 1 | 102 | 26 |
| 1 | 11 | | Ace | | MS | | MSAPEAQQQKR | | N.D. | N.I. | 84% | N.D. | P25443 | RS2_YEAST | | hNaa60p | **40S ribosomal protein S2** |  | 1 | 77 | 36 |
| 1 | 8 | | AcD3C13 | | MS | | MSDAVTIR | | N.D. | N.I. | 20% | N.D. | P26782 | RS24_YEAST | | hNaa60p | **40S ribosomal protein S24** |  | 1 | 46 | 37 |
| 1 | 9 | | Ace,AcD3C13 | | MS | | MSDEAKEKR | | partial Ac- | 10% | 65% | 55% | A6ZQX9 | CHZ1_YEAST | | control,hNaa60p | **Histone H2A.Z-specific chaperone CHZ1** | P40019 (1-9) | 3 | 69 | 36 |
| 1 | 27 | | AcD3C13 | | MS | | MSDEEHTFETADAGSSATYPMQCSALR | | partial Ac- | 11% | 42% | 31% | P23301 | IF5A2_YEAST | | control,hNaa60p | **Eukaryotic translation initiation factor 5A-2** |  | 3 | 72 | 23 |
| 1 | 10 | | AcD3C13 | | MS | | MSDPVELLKR | | N.D. | N.I. | 0% | N.D. | P32602 | SEC17_YEAST | | hNaa60p | **Alpha-soluble NSF attachment protein** |  | 1 | 58 | 37 |
| 1 | 10 | | AcD3C13 | | MS | | MSDYVELLKR | | 100% free | 0% | N.I. | N.D. | P38079 | YRO2_YEAST | | control | **Protein YRO2** |  | 1 | 44 | 37 |
| 1 | 11 | | AcD3C13 | | MS | | MSEEGPQVKIR | | 100% free | 0% | 5% | 5% | P16370 | RPB3_YEAST | | control,hNaa60p | **DNA-directed RNA polymerase II subunit RPB3** |  | 2 | 52 | 37 |
| 1 | 9 | | Ace,AcD3C13 | | MS | | MSEEKTYKR | | partial Ac- | 3% | 85% | 82% | P33775 | PMT1_YEAST | | hNaa60p,control | **Dolichyl-phosphate-mannose--protein mannosyltransferase 1** |  | 3 | 66 | 37 |
| 1 | 14 | | Ace | | MS | | MSEEPPSDQVNSLR | | N.D. | N.I. | 83% | N.D. | P38166 | SFT2_YEAST | | hNaa60p | **Protein transport protein SFT2** |  | 1 | 67 | 33 |
| 1 | 9 | | AcD3C13 | | MS | | MSELEATIR | | 100% free | 0% | 0% | 0% | Q03771 | YD161_YEAST | | control | **UPF0661 TPR repeat-containing protein YDR161W** |  | 1 | 42 | 37 |
| 1 | 11 | | Ace,AcD3C13 | | MS | | MSEQESDEVKR | | partial Ac- | 11% | 85% | 73% | Q12191 | BUG1_YEAST | | hNaa60p,control | **Binder of USO1 and GRH1 protein 1** |  | 2 | 57 | 33 |
| 1 | 13 | | AcD3C13 | | MS | | MSEVIEGNVKIDR | | 100% free | 0% | 12% | 12% | P20081 | FKBP_YEAST | | control,hNaa60p | **FK506-binding protein 1** |  | 7 | 99 | 37 |
| 1 | 12 | | Ace | | MS | | MSGNGAQGTKFR | | N.D. | N.I. | 72% | N.D. | P04451 | RL23_YEAST | | hNaa60p | **60S ribosomal protein L23** |  | 1 | 71 | 35 |
| 1 | 10 | | Ace,AcD3C13 | | MS | | MSKITSSQVR | | partial Ac- | 37% | 68% | 31% | P53030 | RL1_YEAST | | hNaa60p,control | **60S ribosomal protein L1** |  | 4 | 72 | 37 |
| 1 | 6 | | Ace | | MS | | MSKVPR | | N.D. | N.I. | 100% | N.D. | P53152 | MMS2_YEAST | | hNaa60p | **Ubiquitin-conjugating enzyme variant MMS2** |  | 1 | 38 | 37 |
| 1 | 8 | | Ace | | MS | | MSNVVQAR | | N.D. | N.I. | N.D. | N.D. | P06367 | RS14A_YEAST | | hNaa60p | **40S ribosomal protein S14-A** |  | 2 | 52 | 36 |
| 1 | 17 | | AcD3C13 | | MS | | MSPMKVAVVGASGKVGR | | N.D. | N.I. | 0% | N.D. | Q04304 | YMY0_YEAST | | hNaa60p | **UPF0659 protein YMR090W** |  | 1 | 49 | 37 |
| 1 | 10 | | Ace | | MS | | MSQAVNAKKR | | partial Ac- | 86% | 92% | 6% | Q01855 | RS15_YEAST | | hNaa60p,control | **40S ribosomal protein S15** |  | 2 | 60 | 37 |
| 1 | 17 | | AcD3C13 | | MS | | MSQVYFDVEADGQPIGR | | partial Ac- | 6% | 17% | 11% | P14832 | CYPH_YEAST | | control,hNaa60p | **Peptidyl-prolyl cis-trans isomerase** |  | 3 | 97 | 35 |
| 1 | 8 | | Ace | | MS | | MSSSESIR | | 100% Ac | 100% | 97% | -3% | P07170 | KAD1_YEAST | | control,hNaa60p | **Adenylate kinase cytosolic** |  | 2 | 45 | 32 |
| 1 | 11 | | AcD3C13 | | MS | | MSSVESSPISR | | 100% free | 0% | N.I. | N.D. | Q12048 | ICY2_YEAST | | control | **Protein ICY2** |  | 1 | 40 | 36 |
| 1 | 11 | | AcD3C13,Ace | | MS | | MSTELTVQSER | | partial Ac- | 22% | 38% | 16% | P26781 | RS11_YEAST | | hNaa60p,control | **40S ribosomal protein S11** |  | 3 | 74 | 35 |
| **17. Met-Thr-** | | | | | | | | | | | | | | | | | | | |  |  |
| 1 | 18 | | AcD3C13 | | MT | | MTAIEDILQITTDPSDTR | | N.D. | N.I. | 15% | N.D. | P54860 | UFD2_YEAST | | hNaa60p | **Ubiquitin conjugation factor E4** |  | 1 | 59 | 37 |
| 1 | 19 | | AcD3C13 | | MT | | MTDPHLNTPQVSTSPTFER | | 100% free | 0% | N.I. | N.D. | P43598 | YFI7_YEAST | | control | **Uncharacterized protein YFR017C** |  | 1 | 58 | 36 |
| 1 | 11 | | AcD3C13 | | MT | | MTEDFISSVKR | | N.D. | N.I. | 76% | N.D. | P38967 | TAT2_YEAST | | hNaa60p | **Tryptophan permease** |  | 1 | 48 | 37 |
| 1 | 11 | | AcD3C13,Ace | | MT | | MTEDKSQVKIR | | 100% free | 0% | 28% | 28% | Q12024 | YTM1_YEAST | | hNaa60p,control | **Microtubule-associated protein YTM1** |  | 3 | 54 | 37 |
| 1 | 18 | | AcD3C13 | | MT | | MTEFELPPKYITAANDLR | | N.D. | N.I. | 23% | N.D. | P37303 | GLY1_YEAST | | hNaa60p | **Low specificity L-threonine aldolase** |  | 1 | 65 | 38 |
| 1 | 19 | | AcD3C13 | | MT | | MTEFKAGSAKKGATLFKTR | | N.D. | N.I. | 12% | N.D. | P00044 | CYC1_YEAST | | hNaa60p | **Cytochrome c iso-1** |  | 2 | 50 | 35 |
| 1 | 19 | | Ace | | MT | | MTGAATAAENSATQLEFYR | | N.D. | N.I. | 67% | N.D. | Q12517 | DCP1_YEAST | | hNaa60p | **mRNA-decapping enzyme subunit 1** |  | 1 | 88 | 33 |
| 1 | 12 | | AcD3C13 | | MT | | MTIAPITGTIKR | | 100% free | 0% | 9% | 9% | P07255 | COX9_YEAST | | hNaa60p,control | **Cytochrome c oxidase subunit 7A** |  | 2 | 59 | 34 |
| 1 | 11 | | Ace | | MT | | MTKEEIADKKR | | N.D. | N.I. | 76% | N.D. | A6ZUA1 | DBP3_YEAST | | hNaa60p | **ATP-dependent RNA helicase DBP3** | P20447 (1-11) | 2 | 64 | 38 |
| 1 | 11 | | Ace,AcD3C13 | | MT | | MTKSEQQADSR | | partial Ac- | 48% | 91% | 43% | P32582 | CBS_YEAST | | hNaa60p,control | **Cystathionine beta-synthase** |  | 4 | 67 | 34 |
| 1 | 9 | | AcD3C13 | | MT | | MTLAELLGR | | 100% free | 0% | 0% | 0% | Q02950 | RT51_YEAST | | control,hNaa60p | **37S ribosomal protein MRP51, mitochondrial** |  | 2 | 61 | 36 |
| 1 | 16 | | AcD3C13 | | MT | | MTLDDDDYIKQMELQR | | N.D. | N.I. | 48% | N.D. | P40546 | FAF1_YEAST | | hNaa60p | **Protein FAF1** |  | 1 | 45 | 34 |
| 1 | 11 | | Ace | | MT | | MTNKSSLKNNR | | N.D. | N.I. | 85% | N.D. | Q08438 | VHS3_YEAST | | hNaa60p | **Protein VHS3** |  | 1 | 69 | 37 |
| 1 | 8 | | Ace,AcD3C13 | | MT | | MTPEAKKR | | 100% free | 0% | 50% | 50% | P48415 | SEC16_YEAST | | control,hNaa60p | **COPII coat assembly protein SEC16** |  | 14 | 60 | 36 |
| 1 | 13 | | Ace,AcD3C13 | | MT | | MTPEQKAKLEANR | | partial Ac- | 11% | 44% | 33% | P28519 | RAD14_YEAST | | hNaa60p,control | **DNA repair protein RAD14** |  | 6 | 66 | 37 |
| 1 | 11 | | Ace | | MT | | MTSATDKSIDR | | N.D. | N.I. | 100% | N.D. | A6ZSP9 | LAG1_YEAST | | hNaa60p | **Sphingosine N-acyltransferase LAG1** | P38703 (1-11) | 1 | 62 | 35 |
| 1 | 9 | | Ace | | MT | | MTTNEEFIR | | N.D. | N.I. | 60% | N.D. | P32485 | HOG1_YEAST | | hNaa60p | **Mitogen-activated protein kinase HOG1** |  | 1 | 36 | 33 |
| **18. Met-Val-** | | | | | | | | | | | | | | | | | | | |  |  |
| 1 | 10 | | AcD3C13,Ace | | MV | | MVAFTVDQMR | | partial Ac- | 12% | N.D. | N.D. | P32324 | EF2_YEAST | | control,hNaa60p | **Elongation factor 2** |  | 12 | 70 | 34 |
| 1 | 9 | | Ace,AcD3C13 | | MV | | MVALISKKR | | partial Ac- | 23% | 78% | 55% | P05750 | RS3_YEAST | | control,hNaa60p | **40S ribosomal protein S3** |  | 4 | 86 | 31 |
| 1 | 11 | | Ace | | MV | | MVDGLNTSNIR | | N.D. | N.I. | 82% | N.D. | P47013 | DS1P1_YEAST | | hNaa60p | **Dihydrosphingosine 1-phosphate phosphatase LCB3** |  | 1 | 58 | 36 |
| 1 | 21 | | Ace | | MV | | MVELTEIKDDVVQLDEPQFSR | | N.D. | N.I. | 100% | N.D. | P49334 | TOM22_YEAST | | hNaa60p | **Mitochondrial import receptor subunit TOM22** |  | 1 | 59 | 37 |
| 1 | 7 | | AcD3C13 | | MV | | MVFDLKR | | 100% free | 0% | N.I. | N.D. | P07172 | HIS8_YEAST | | control | **Histidinol-phosphate aminotransferase** |  | 1 | 47 | 37 |
| 1 | 12 | | AcD3C13,Ace | | MV | | MVGQQYSSAPLR | | partial Ac- | 23% | 62% | 39% | P04050 | RPB1_YEAST | | control,hNaa60p | **DNA-directed RNA polymerase II subunit RPB1** |  | 2 | 66 | 36 |
| 1 | 17 | | Ace,AcD3C13 | | MV | | MVLVQDLLHPTAASEAR | | 100% free | 0% | 51% | 51% | P35997 | RS27A_YEAST | | control,hNaa60p | **40S ribosomal protein S27-A** | P38711 (1-17) | 9 | 120 | 37 |
| 1 | 10 | | Ace,AcD3C13 | | MV | | MVNELENVPR | | partial Ac- | 2% | 93% | 91% | P39010 | AKR1_YEAST | | hNaa60p,control | **Palmitoyltransferase AKR1** |  | 3 | 72 | 36 |
| 1 | 8 | | AcD3C13 | | MV | | MVNVPKTR | | N.D. | N.I. | 71% | N.D. | P02405 | RL44_YEAST | | hNaa60p | **60S ribosomal protein L42** |  | 1 | 47 | 36 |
| 1 | 19 | | Ace,AcD3C13 | | MV | | MVPAESNAVQAKLAKTLQR | | 100% free | 0% | 85% | 85% | Q12125 | YO164_YEAST | | hNaa60p,control | **UPF0363 protein YOR164C** |  | 2 | 78 | 36 |
| 1 | 11 | | AcD3C13,Ace | | MV | | MVQSAVLGFPR | | partial Ac- | 4% | 44% | 40% | P05694 | METE_YEAST | | hNaa60p,control | **5-methyltetrahydropteroyltriglutamate--homocysteine methyltransferase** |  | 3 | 80 | 37 |
| 1 | 15 | | Ace,AcD3C13 | | MV | | MVTSNVVLVSGEGER | | 100% free | 0% | 34% | 34% | P52286 | SKP1_YEAST | | hNaa60p,control | **Suppressor of kinetochore protein 1** |  | 7 | 112 | 37 |
| 1 | 10 | | AcD3C13 | | MV | | MVTVGVFSER | | 100% free | 0% | 30% | 30% | A6ZT71 | SOL3_YEAST | | hNaa60p,control | **6-phosphogluconolactonase 3** | B3LSS7 (1-10)^AB5VK90 (1-10)^AP38858 (1-10) | 2 | 70 | 37 |
| 1 | 6 | | AcD3C13 | | MV | | MVVKKR | | N.D. | N.I. | 25% | N.D. | Q02206 | RSC4_YEAST | | hNaa60p | **Chromatin structure-remodeling complex subunit RSC4** |  | 1 | 42 | 31 |
| 1 | 11 | | AcD3C13 | | MV | | MVVLDKKLLER | | 100% free | 0% | N.I. | N.D. | P38276 | YBY7_YEAST | | control | **UPF0303 protein YBR137W** |  | 1 | 44 | 33 |
| 1 | 10 | | Ace | | MV | | MVVVGKSEVR | | N.D. | N.I. | 87% | N.D. | Q04119 | PPN1_YEAST | | hNaa60p | **Endopolyphosphatase** |  | 1 | 79 | 37 |
| **19. Met-Tyr-** | | | | | | | | | | | | | | | | | | | |  |  |
| 1 | 12 | | Ace,AcD3C13 | | MY | | MYFDKDNSMSPR | | 100% free | 0% | 88% | 88% | Q12236 | KOK0_YEAST | | hNaa60p,control | **Probable serine/threonine-protein kinase YOL100W** |  | 3 | 83 | 29 |
| 1 | 13 | | AcD3C13,Ace | | MY | | MYFTDESSPAMNR | | 100% free | 0% | 43% | 43% | P52960 | PIP2_YEAST | | control,hNaa60p | **Peroxisome proliferation transcriptional regulator** |  | 3 | 70 | 26 |
| 1 | 15 | | AcD3C13 | | MY | | MYGDLGNKLVLEAKR | | N.D. | N.I. | 22% | N.D. | Q12488 | PSF1_YEAST | | hNaa60p | **DNA replication complex GINS protein PSF1** |  | 2 | 75 | 37 |
| 1 | 14 | | AcD3C13 | | MY | | MYIKAEQKPQQFER | | partial Ac- | 20% | 33% | 13% | P40036 | GIP2_YEAST | | hNaa60p,control | **GLC7-interacting protein 2** |  | 2 | 60 | 38 |
| 1 | 7 | | Ace | | MY | | MYPGSGR | | 100% Ac | 100% | 100% | 0% | A6ZP43 | MCA1_YEAST | | control,hNaa60p | **Metacaspase-1** | Q08601 (1-7) | 2 | 40 | 31 |
| 1 | 9 | | Ace | | MY | | MYSTPLKKR | | 100% Ac | 100% | 100% | 0% | P40064 | NU157_YEAST | | hNaa60p,control | **Nucleoporin NUP157** |  | 5 | 51 | 37 |
| 1 | 15 | | Ace,AcD3C13 | | MY | | MYTNYSLTSSDAMPR | | partial Ac- | 53% | 85% | 32% | Q03764 | EKI1_YEAST | | hNaa60p,control | **Ethanolamine kinase** |  | 3 | 106 | 28 |
| 1 | 12 | | Ace,AcD3C13 | | MY | | MYVDPMNNNEIR | | 100% free | 0% | 56% | 56% | P05986 | KAPC_YEAST | | hNaa60p,control | **cAMP-dependent protein kinase type 3** |  | 5 | 61 | 29 |
| 1 | 6 | | Ace,AcD3C13 | | MY | | MYVYKR | | partial Ac- | 83% | 80% | -3% | P21524 | RIR1_YEAST | | control,hNaa60p | **Ribonucleoside-diphosphate reductase large chain 1** |  | 4 | 52 | 37 |
| **20. Pro-** | | | | | | | | | | | | | | | | | | | |  |  |
| 2 | 12 | | AcD3C13 | | PA | | PAENQNTGQDR | | 100% free | 0% | N.I. | N.D. | P27895 | CIN8_YEAST | | control | **Kinesin-like protein CIN8** |  | 1 | 55 | 34 |
| 2 | 18 | | AcD3C13 | | PD | | PDNNTEQLQGSPSSDQR | | 100% free | 0% | 0% | 0% | Q04934 | IVY1_YEAST | | hNaa60p,control | **Protein IVY1** |  | 4 | 96 | 33 |
| 2 | 19 | | AcD3C13 | | PD | | PDSKYTMQGYNLVKLLKR | | 100% free | 0% | N.I. | N.D. | P17555 | CAP_YEAST | | control | **Adenylyl cyclase-associated protein** |  | 4 | 68 | 36 |
| 2 | 14 | | AcD3C13 | | PD | | PDYDNYTTPLSSR | | N.D. | N.I. | 0% | N.D. | Q05911 | PUR8_YEAST | | hNaa60p | **Adenylosuccinate lyase** |  | 2 | 54 | 35 |
| 2 | 18 | | AcD3C13 | | PE | | PEIYGPQPLKPLNTVMR | | 100% free | 0% | 0% | 0% | P38873 | KOG1_YEAST | | hNaa60p,control | **Target of rapamycin complex 1 subunit KOG1** |  | 4 | 75 | 37 |
| 2 | 11 | | AcD3C13 | | PE | | PELTEFQKKR | | 100% free | 0% | 0% | 0% | Q12510 | YD156_YEAST | | hNaa60p,control | **WD repeat-containing protein YDL156W** |  | 2 | 59 | 36 |
| 2 | 14 | | AcD3C13 | | PE | | PEQAQQGEQSVKR | | 100% free | 0% | 0% | 0% | P40467 | YIN0_YEAST | | control,hNaa60p | **Uncharacterized transcriptional regulatory protein YIL130W** |  | 2 | 87 | 37 |
| 2 | 12 | | AcD3C13 | | PE | | PEYTLLADNIR | | N.D. | N.I. | 0% | N.D. | P36029 | TPO5_YEAST | | hNaa60p | **Polyamine transporter TPO5** |  | 1 | 67 | 37 |
| 2 | 9 | | AcD3C13 | | PG | | PGFTAPTR | | N.D. | N.I. | 0% | N.D. | Q6Q560 | ISD11_YEAST | | hNaa60p | **Protein ISD11** |  | 1 | 38 | 38 |
| 2 | 24 | | AcD3C13 | | PI | | PIDQEKLAKLQKLSANNKVGGTR | | 100% free | 0% | 0% | 0% | A6ZWL1 | NACB1_YEAST | | hNaa60p,control | **Nascent polypeptide-associated complex subunit beta-1** | Q02642 (2-24) | 41 | 69 | 31 |
| 2 | 16 | | AcD3C13 | | PI | | PIGNPINTNDIKSNR | | 100% free | 0% | 0% | 0% | P53438 | SOK2_YEAST | | hNaa60p,control | **Protein SOK2** |  | 2 | 98 | 37 |
| 2 | 8 | | AcD3C13 | | PI | | PITIKSR | | 100% free | 0% | 0% | 0% | Q99190 | TSC13_YEAST | | control,hNaa60p | **Enoyl reductase TSC13** |  | 2 | 47 | 34 |
| 2 | 21 | | AcD3C13 | | PK | | PKESEVINSEFHVDVQDPER | | 100% free | 0% | N.I. | N.D. | P22517 | KCC2_YEAST | | control | **Calcium/calmodulin-dependent protein kinase II** |  | 1 | 96 | 37 |
| 2 | 34 | | AcD3C13 | | PK | | PKETPSKAAADALSDLEIKDSKSNLNKELETLR | | 100% free | 0% | N.I. | N.D. | P09938 | RIR2_YEAST | | control | **Ribonucleoside-diphosphate reductase small chain 1** |  | 1 | 47 | 35 |
| 2 | 38 | | AcD3C13 | | PK | | PKETPSKAAADALSDLEIKDSKSNLNKELETLREENR | | 100% free | 0% | N.I. | N.D. | P09938 | RIR2_YEAST | | control | **Ribonucleoside-diphosphate reductase small chain 1** |  | 1 | 145 | 36 |
| 2 | 8 | | AcD3C13 | | PK | | PKLVLVR | | 100% free | 0% | 0% | 0% | P00950 | PMG1_YEAST | | control,hNaa60p | **Phosphoglycerate mutase 1** |  | 414 | 63 | 19 |
| 2 | 9 | | AcD3C13 | | PL | | PLMSNSER | | 100% free | 0% | 0% | 0% | Q06817 | SYG2_YEAST | | control,hNaa60p | **Glycyl-tRNA synthetase 2** |  | 3 | 50 | 36 |
| 2 | 9 | | AcD3C13 | | PL | | PLNKSNIR | | 100% free | 0% | 0% | 0% | P01120 | RAS2_YEAST | | control,hNaa60p | **Ras-like protein 2** |  | 6 | 64 | 34 |
| 2 | 14 | | AcD3C13 | | PL | | PLPPSTLNQKSNR | | N.D. | N.I. | 0% | N.D. | P19454 | CSK22_YEAST | | hNaa60p | **Casein kinase II subunit alpha'** |  | 1 | 52 | 36 |
| 2 | 12 | | AcD3C13 | | PL | | PLSQKQIDQVR | | 100% free | 0% | 0% | 0% | P33441 | MFT1_YEAST | | hNaa60p,control | **THO complex subunit MFT1** |  | 3 | 92 | 35 |
| 2 | 9 | | AcD3C13 | | PN | | PNASQVYR | | 100% free | 0% | 0% | 0% | P16120 | THRC_YEAST | | control,hNaa60p | **Threonine synthase** |  | 7 | 65 | 37 |
| 2 | 10 | | AcD3C13 | | PN | | PNDNKTPNR | | N.D. | N.I. | 0% | N.D. | P11978 | SIR4_YEAST | | hNaa60p | **Regulatory protein SIR4** |  | 1 | 41 | 37 |
| 2 | 24 | | AcD3C13 | | PN | | PNVLSDDEELLNGLGSEIMKPSR | | N.D. | N.I. | 0% | N.D. | Q06137 | YL345_YEAST | | hNaa60p | **Putative 6-phosphofructo-2-kinase/fructose-2,6-biphosphatase YLR345W** |  | 2 | 69 | 37 |
| 2 | 9 | | AcD3C13 | | PP | | PPNSKSKR | | 100% free | 0% | 0% | 0% | A6ZRZ0 | NST1_YEAST | | control,hNaa60p | **Stress response protein NST1** | P53935 (2-9) | 3 | 40 | 35 |
| 2 | 11 | | AcD3C13 | | PP | | PPVSASKAKR | | 100% free | 0% | 0% | 0% | P40024 | ARB1_YEAST | | control,hNaa60p | **ABC transporter ATP-binding protein ARB1** |  | 3 | 77 | 33 |
| 2 | 16 | | AcD3C13 | | PQ | | PQKPLKVTKKAKDPR | | 100% free | 0% | N.I. | N.D. | Q3E747 | YL363_YEAST | | control | **Uncharacterized protein YLR363W-A** |  | 3 | 50 | 23 |
| 2 | 9 | | AcD3C13 | | PQ | | PQNDYIER | | 100% free | 0% | 0% | 0% | A6ZR80 | NSA2_YEAST | | hNaa60p,control | **Ribosome biogenesis protein NSA2** | P40078 (2-9) | 3 | 56 | 36 |
| 2 | 10 | | AcD3C13 | | PQ | | PQSFTSIAR | | 100% free | 0% | 0% | 0% | P00128 | QCR7_YEAST | | hNaa60p,control | **Cytochrome b-c1 complex subunit 7** |  | 2 | 64 | 37 |
| 2 | 12 | | AcD3C13 | | PQ | | PQSTPSQEVQR | | 100% free | 0% | N.I. | N.D. | P53134 | YGL4_YEAST | | control | **Putative oligopeptide transporter YGL114W** |  | 1 | 51 | 37 |
| 2 | 15 | | AcD3C13 | | PS | | PSKDPESVIDKEIR | | 100% free | 0% | 4% | 4% | P40060 | IES5_YEAST | | hNaa60p,control | **Ino eighty subunit 5** |  | 4 | 65 | 37 |
| 2 | 23 | | AcD3C13 | | PS | | PSTLTINGKAPIVAYAELIAAR | | N.D. | N.I. | 0% | N.D. | P46655 | SYEC_YEAST | | hNaa60p | **Glutamyl-tRNA synthetase, cytoplasmic** |  | 1 | 42 | 34 |
| 2 | 15 | | AcD3C13 | | PT | | PTNSIKLLAPDVHR | | N.D. | N.D. | N.I. | N.D. | P38689 | KPR3_YEAST | | control | **Ribose-phosphate pyrophosphokinase 3** |  | 1 | 77 | 35 |
| 2 | 17 | | AcD3C13 | | PT | | PTPMTPVKVGALACQR | | 100% free | 0% | 0% | 0% | P53960 | YNE0_YEAST | | control,hNaa60p | **Uncharacterized protein YNL040W** |  | 2 | 64 | 37 |
| **21. Ser-** | | | | | | | | | | | | | | | | | | | |  |  |
| 2 | 35 | | Ace | | SA | | SAAPLDYKKALEHLKTYSSKDGLSVQELMDSTTR | | N.D. | N.I. | N | N.D. | P50094 | IMDH4_YEAST | | hNaa60p | **Probable inosine-5'-monophosphate dehydrogenase IMD4** |  | 1 | 40 | 37 |
| 2 | 17 | | Ace | | SA | | SAAPVQDKDTLSNAER | | 100% Ac | 100% | N.I. | N.D. | P19659 | MED15_YEAST | | control | **Mediator of RNA polymerase II transcription subunit 15** |  | 1 | 38 | 37 |
| 2 | 24 | | Ace | | SA | | SAATATAAPVPPPVGISNLPNQR | | N.D. | N.D. | N.I. | N.D. | P32468 | CDC12_YEAST | | control | **Cell division control protein 12** |  | 1 | 68 | 37 |
| 2 | 45 | | Ace | | SA | | SAEDYKNLPVTVEKPIPVVYDLGNLAAFDSNVLDKNDLDSSNAR | | N.D. | N.D. | N.I. | N.D. | Q08746 | RRS1_YEAST | | control | **Regulator of ribosome biosynthesis** |  | 1 | 47 | 36 |
| 2 | 25 | | Ace | | SA | | SAEIEEATNAVNNLSINDSEQQPR | | N.D. | N.D. | 100% | N.D. | P10080 | SSBP1_YEAST | | hNaa60p,control | **Single-stranded nucleic acid-binding protein** |  | 40 | 142 | 33 |
| 2 | 25 | | Ace | | SA | | SAENISTGSPTGKQPSSEVNLGER | | partial Ac- | 97% | N.I. | N.D. | Q3E784 | SLO1_YEAST | | control | **SCOCO-like protein 1** |  | 1 | 63 | 37 |
| 2 | 18 | | Ace,AcD3C13 | | SA | | SAGDISAINIKSVKKNR | | N.D. | N.D. | 26% | N.D. | Q05942 | RSA3_YEAST | | control,hNaa60p | **Ribosome assembly protein 3** |  | 7 | 80 | 33 |
| 2 | 21 | | Ace | | SA | | SAIPENANVTVLNKNEKKAR | | 100% Ac | 98% | 95% | -3% | A6ZT99 | NACA_YEAST | | control,hNaa60p | **Nascent polypeptide-associated complex subunit alpha** | P38879 (2-21) | 15 | 87 | 36 |
| 2 | 13 | | Ace | | SA | | SAIYKLSIQGIR | | N.D. | N.I. | 100% | N.D. | P12753 | RAD50_YEAST | | hNaa60p | **DNA repair protein RAD50** |  | 1 | 43 | 35 |
| 2 | 33 | | Ace | | SA | | SAKAEKKPASKAPAEKKPAAKKTSTSTDGKKR | | 100% Ac | 100% | N.D. | N.D. | P02293 | H2B1_YEAST | | hNaa60p,control | **Histone H2B.1** |  | 6 | 72 | 26 |
| 2 | 10 | | Ace | | SA | | SAKAQNPMR | | 100% Ac | 100% | 100% | 0% | P0C0W9 | RL11A_YEAST | | hNaa60p,control | **60S ribosomal protein L11-A** |  | 111 | 69 | 37 |
| 2 | 24 | | Ace | | SA | | SANDKQYISYNNVHQLCQVSAER | | 100% Ac | 100% | 100% | 0% | Q04178 | HPRT_YEAST | | control,hNaa60p | **Hypoxanthine-guanine phosphoribosyltransferase** |  | 2 | 67 | 34 |
| 2 | 19 | | Ace | | SA | | SANDYYGGTAGEKSQYSR | | 100% Ac | 100% | 100% | 0% | P38216 | YBM6_YEAST | | hNaa60p,control | **Uncharacterized protein YBR016W** |  | 20 | 111 | 30 |
| 2 | 23 | | Ace | | SA | | SANEFYSSGQQGQYNQQNNQER | | N.D. | N.D. | N.D. | N.D. | P40159 | YNU8_YEAST | | control,hNaa60p | **Uncharacterized protein YNL208W** |  | 72 | 130 | 25 |
| 2 | 22 | | Ace | | SA | | SANLDKSLDEIIGSNKAGSNR | | 100% Ac | 100% | 100% | 0% | Q12159 | YRA1_YEAST | | control,hNaa60p | **RNA annealing protein YRA1** |  | 13 | 72 | 38 |
| 2 | 14 | | Ace | | SA | | SANNGVTGKLSSR | | 100% Ac | 100% | 100% | 0% | P53725 | MPP6_YEAST | | hNaa60p,control | **M-phase phosphoprotein 6 homolog** |  | 3 | 54 | 37 |
| 2 | 9 | | Ace | | SA | | SANSGVKR | | N.D. | N.I. | 78% | N.D. | P19541 | YP133_YEAST | | hNaa60p | **Uncharacterized transcriptional regulatory protein YPL133C** |  | 1 | 51 | 38 |
| 2 | 31 | | Ace | | SA | | SAPAANGEVPTFKLVLVGDGGTGKTTFVKR | | 100% Ac | 100% | N.I. | N.D. | P32835 | GSP1_YEAST | | control | **GTP-binding nuclear protein GSP1/CNR1** |  | 1 | 44 | 36 |
| 2 | 20 | | Ace | | SA | | SAPAQNYKIADISLAAFGR | | 100% Ac | 100% | 100% | 0% | P39954 | SAHH_YEAST | | control,hNaa60p | **Adenosylhomocysteinase** |  | 31 | 110 | 38 |
| 2 | 11 | | Ace | | SA | | SAPEAQQQKR | | 100% Ac | 100% | 100% | 0% | P25443 | RS2_YEAST | | hNaa60p,control | **40S ribosomal protein S2** |  | 50 | 72 | 37 |
| 2 | 18 | | Ace | | SA | | SAQDYYGNSASKQSYSR | | 100% Ac | 100% | 100% | 0% | Q12489 | YD012_YEAST | | hNaa60p,control | **Uncharacterized protein YDL012C** |  | 10 | 107 | 31 |
| 2 | 31 | | Ace | | SA | | SAQDYYGNSASKQSYSRPSAPPPGYETASR | | N.D. | N.I. | N.D. | N.D. | Q12489 | YD012_YEAST | | hNaa60p | **Uncharacterized protein YDL012C** |  | 1 | 37 | 32 |
| 2 | 8 | | Ace | | SA | | SASLINR | | 100% Ac | 100% | 100% | 0% | Q06449 | PIN3_YEAST | | control,hNaa60p | **[PSI+] inducibility protein 3** |  | 2 | 50 | 36 |
| 2 | 8 | | Ace | | SA | | SASLVNR | | 100% Ac | 100% | N.I. | N.D. | P53281 | LSB1_YEAST | | control | **LAS seventeen-binding protein 1** |  | 4 | 56 | 35 |
| 2 | 23 | | Ace | | SA | | SATLFNNIELLPPDALFGIKQR | | 100% Ac | 100% | 100% | 0% | P23542 | AATC_YEAST | | hNaa60p,control | **Aspartate aminotransferase, cytoplasmic** |  | 3 | 56 | 36 |
| 2 | 12 | | Ace | | SA | | SATLKDYLNKR | | 100% Ac | 100% | 97% | -3% | P47093 | LSM8_YEAST | | control,hNaa60p | **U6 snRNA-associated Sm-like protein LSm8** |  | 2 | 71 | 37 |
| 2 | 14 | | Ace | | SA | | SAVATYLKTLTAR | | 100% Ac | 100% | 100% | 0% | Q96VH4 | HBN1_YEAST | | control,hNaa60p | **Putative nitroreductase HBN1** |  | 2 | 77 | 36 |
| 2 | 14 | | Ace | | SD | | SDAQFDAALDLLR | | 100% Ac | 100% | N.I. | N.D. | P13517 | CAPZB_YEAST | | control | **F-actin-capping protein subunit beta** |  | 4 | 93 | 37 |
| 2 | 8 | | Ace | | SD | | SDAVTIR | | 100% Ac | 100% | 100% | 0% | P26782 | RS24_YEAST | | hNaa60p,control | **40S ribosomal protein S24** |  | 62 | 70 | 36 |
| 2 | 9 | | Ace | | SD | | SDEAKEKR | | 100% Ac | 100% | 100% | 0% | A6ZQX9 | CHZ1_YEAST | | hNaa60p,control | **Histone H2A.Z-specific chaperone CHZ1** | P40019 (2-9) | 12 | 66 | 37 |
| 2 | 27 | | Ace | | SD | | SDEEHTFETADAGSSATYPMQCSALR | | 100% Ac | 100% | 100% | 0% | P23301 | IF5A2_YEAST | | control,hNaa60p | **Eukaryotic translation initiation factor 5A-2** |  | 12 | 99 | 24 |
| 2 | 23 | | Ace | | SD | | SDFQKEKVEEQEQQQQQIIKIR | | 100% Ac | 100% | 100% | 0% | P38701 | RS20_YEAST | | hNaa60p,control | **40S ribosomal protein S20** |  | 23 | 80 | 37 |
| 2 | 20 | | Ace | | SD | | SDHDTPMESIQNGENSDER | | 100% free | 0% | N.D. | N.D. | Q02455 | MLP1_YEAST | | hNaa60p,control | **Protein MLP1** |  | 2 | 41 | 21 |
| 2 | 18 | | Ace | | SD | | SDIEEGTPTNNGQQKER | | N.D. | N.I. | 100% | N.D. | P11746 | MCM1_YEAST | | hNaa60p | **Pheromone receptor transcription factor** |  | 1 | 85 | 35 |
| 2 | 11 | | Ace | | SD | | SDILNVSQQR | | 100% Ac | 100% | 100% | 0% | Q05788 | PNPH_YEAST | | control,hNaa60p | **Purine nucleoside phosphorylase** |  | 3 | 68 | 38 |
| 2 | 15 | | Ace | | SD | | SDKIQEEILGLVSR | | 100% Ac | 100% | N.I. | N.D. | Q12387 | MDM20_YEAST | | control | **N-terminal acetyltransferase B complex subunit MDM20** |  | 1 | 43 | 37 |
| 2 | 18 | | Ace | | SD | | SDLNDVQENAKLNSETR | | 100% Ac | 100% | N.I. | N.D. | A6ZXL6 | ATG20_YEAST | | control | **Autophagy-related protein 20** | Q07528 (2-18) | 3 | 93 | 36 |
| 2 | 20 | | Ace | | SD | | SDLNQSKKMNVSEFADAQR | | N.D. | N.I. | 100% | N.D. | P54790 | ORC3_YEAST | | hNaa60p | **Origin recognition complex subunit 3** |  | 2 | 63 | 36 |
| 2 | 9 | | Ace | | SD | | SDLQKLQR | | 100% Ac | 100% | N.I. | N.D. | P40011 | YEK0_YEAST | | control | **Uncharacterized protein YER010C** |  | 1 | 53 | 37 |
| 2 | 20 | | Ace | | SD | | SDNLLSLENPVVPSHYELR | | 100% Ac | 100% | 100% | 0% | P40462 | TM108_YEAST | | hNaa60p,control | **Protein TMA108** |  | 4 | 115 | 37 |
| 2 | 10 | | Ace | | SD | | SDPVELLKR | | 100% Ac | 100% | N.I. | N.D. | P32602 | SEC17_YEAST | | control | **Alpha-soluble NSF attachment protein** |  | 1 | 56 | 36 |
| 2 | 12 | | Ace | | SD | | SDQPETPSNSR | | 100% Ac | 100% | N.I. | N.D. | Q03220 | CTL1_YEAST | | control | **Polynucleotide 5'-triphosphatase** |  | 1 | 45 | 32 |
| 2 | 28 | | Ace | | SD | | SDSNQGNNQQNYQQYSQNGNQQQGNNR | | N.D. | N.D. | N.D. | N.D. | P05453 | ERF3_YEAST | | control,hNaa60p | **Eukaryotic peptide chain release factor GTP-binding subunit** |  | 6 | 98 | 23 |
| 2 | 9 | | Ace | | SD | | SDSPLSKR | | 100% Ac | 100% | N.I. | N.D. | Q12267 | SMC4_YEAST | | control | **Structural maintenance of chromosomes protein 4** |  | 1 | 45 | 38 |
| 2 | 26 | | Ace | | SD | | SDSQQSIKVLEELFQKLSVATADNR | | 100% Ac | 100% | N.D. | N.D. | P16521 | EF3A_YEAST | | hNaa60p,control | **Elongation factor 3A** |  | 17 | 110 | 38 |
| 2 | 14 | | Ace | | SD | | SDVLSQYKGCSVR | | 100% Ac | 100% | 100% | 0% | P53095 | YGU6_YEAST | | hNaa60p,control | **Uncharacterized protein YGL196W** |  | 2 | 54 | 36 |
| 2 | 10 | | Ace | | SD | | SDVTQQKKR | | 100% Ac | 100% | N.I. | N.D. | Q06506 | RRP9_YEAST | | control | **Ribosomal RNA-processing protein 9** |  | 1 | 54 | 37 |
| 2 | 9 | | Ace | | SD | | SDVVSKDR | | N.D. | N.I. | 100% | N.D. | P53112 | PEX14_YEAST | | hNaa60p | **Peroxisomal membrane protein PEX14** |  | 1 | 47 | 37 |
| 2 | 12 | | Ace | | SE | | SEDKAKLGTTR | | 100% Ac | 100% | 100% | 0% | P53267 | DAM1_YEAST | | control,hNaa60p | **DASH complex subunit DAM1** |  | 2 | 53 | 37 |
| 2 | 11 | | Ace | | SE | | SEDLSPTSSR | | 100% Ac | 100% | 100% | 0% | Q9P305 | IGO2_YEAST | | hNaa60p,control | **Protein IGO2** |  | 2 | 79 | 34 |
| 2 | 11 | | Ace | | SE | | SEECIENPER | | N.D. | N.I. | 100% | N.D. | P35202 | THI80_YEAST | | hNaa60p | **Thiamine pyrophosphokinase** |  | 1 | 34 | 30 |
| 2 | 13 | | Ace | | SE | | SEEFIAVSTLAR | | 100% Ac | 100% | 100% | 0% | A6ZTA3 | RIX1_YEAST | | hNaa60p,control | **Pre-rRNA-processing protein RIX1** | P38883 (2-13) | 2 | 87 | 37 |
| 2 | 11 | | Ace | | SE | | SEEGPQVKIR | | 100% Ac | 100% | 100% | 0% | P16370 | RPB3_YEAST | | control | **DNA-directed RNA polymerase II subunit RPB3** |  | 3 | 74 | 37 |
| 2 | 9 | | Ace | | SE | | SEEKTYKR | | partial Ac- | 95% | 100% | 5% | P33775 | PMT1_YEAST | | hNaa60p,control | **Dolichyl-phosphate-mannose--protein mannosyltransferase 1** |  | 5 | 55 | 37 |
| 2 | 21 | | Ace | | SE | | SEENLRPAYDDQVNEDVYKR | | 100% Ac | 100% | N.I. | N.D. | Q12211 | PUS1_YEAST | | control | **tRNA pseudouridine synthase 1** |  | 1 | 88 | 35 |
| 2 | 14 | | Ace | | SE | | SEEPPSDQVNSLR | | 100% Ac | 100% | N.I. | N.D. | P38166 | SFT2_YEAST | | control | **Protein transport protein SFT2** |  | 1 | 41 | 35 |
| 2 | 24 | | Ace | | SE | | SEESLFESSPQKMEYEITNYSER | | 100% Ac | 100% | N | N.D. | Q00955 | ACAC_YEAST | | control,hNaa60p | **Acetyl-CoA carboxylase** |  | 6 | 76 | 30 |
| 2 | 35 | | Ace | | SE | | SEGITDIEESQIQTNYDKVVYKFDDMELDENLLR | | N.D. | N.D. | N.D. | N.D. | A6ZQJ1 | IF4A_YEAST | | control,hNaa60p | **ATP-dependent RNA helicase eIF4A** | P10081 (2-35) | 2 | 54 | 34 |
| 2 | 10 | | Ace | | SE | | SEILQDVQR | | 100% Ac | 100% | 100% | 0% | P46965 | SPC1_YEAST | | control,hNaa60p | **Signal peptidase complex subunit SPC1** |  | 3 | 72 | 37 |
| 2 | 13 | | Ace | | SE | | SEITLGKYLFER | | 100% Ac | 100% | 100% | 0% | P06169 | PDC1_YEAST | | control,hNaa60p | **Pyruvate decarboxylase isozyme 1** | P16467 (2-13)^AP26263 (2-13) | 146 | 93 | 37 |
| 2 | 16 | | Ace | | SE | | SEKPQQEEQEKPQSR | | 100% Ac | 100% | 100% | 0% | P33399 | LAH1_YEAST | | control,hNaa60p | **La protein homolog** |  | 4 | 49 | 37 |
| 2 | 14 | | Ace | | SE | | SELFGVLKSNAGR | | 100% Ac | 100% | 100% | 0% | P47083 | MPP10_YEAST | | hNaa60p,control | **U3 small nucleolar RNA-associated protein MPP10** |  | 6 | 102 | 37 |
| 2 | 22 | | Ace | | SE | | SELGAKYQQLQNELEEFIVAR | | 100% Ac | 100% | N.I. | N.D. | P52553 | PFD6_YEAST | | control | **Prefoldin subunit 6** |  | 1 | 82 | 38 |
| 2 | 24 | | Ace | | SE | | SELNALLKDINGSLTATSESLER | | 100% Ac | 100% | N.D. | N.D. | P40079 | LCP5_YEAST | | control,hNaa60p | **U3 small nucleolar ribonucleoprotein protein LCP5** |  | 3 | 106 | 38 |
| 2 | 10 | | Ace | | SE | | SELTEAEKR | | 100% Ac | 100% | 100% | 0% | P40056 | GET2_YEAST | | control,hNaa60p | **Golgi to ER traffic protein 2** |  | 6 | 62 | 37 |
| 2 | 17 | | Ace | | SE | | SENTTAPSDNITNEQR | | N.D. | N.I. | N.D. | N.D. | P46676 | SUM1_YEAST | | hNaa60p | **Suppressor of mar1-1 protein** |  | 1 | 89 | 32 |
| 2 | 11 | | Ace | | SE | | SEQESDEVKR | | 100% Ac | 100% | 100% | 0% | Q12191 | BUG1_YEAST | | control,hNaa60p | **Binder of USO1 and GRH1 protein 1** |  | 5 | 64 | 35 |
| 2 | 24 | | Ace | | SE | | SEQSQLDDSTIDKLIPQIFNEMR | | 100% Ac | 100% | N.I. | N.D. | P53168 | DUO1_YEAST | | control | **DASH complex subunit DUO1** |  | 1 | 40 | 36 |
| 2 | 9 | | Ace | | SE | | SESEAKSR | | 100% Ac | 100% | N.I. | N.D. | P12754 | EI2BD_YEAST | | control | **Translation initiation factor eIF-2B subunit delta** |  | 1 | 47 | 36 |
| 2 | 24 | | Ace | | SE | | SESPMFAANGMPKVNQGAEEDVR | | partial Ac- | 82% | 100% | 18% | P32449 | AROG_YEAST | | hNaa60p,control | **Phospho-2-dehydro-3-deoxyheptonate aldolase, tyrosine-inhibited** |  | 53 | 102 | 31 |
| 2 | 13 | | Ace | | SE | | SESVKENVTPTR | | 100% Ac | 100% | 100% | 0% | Q05580 | YD266_YEAST | | control,hNaa60p | **LIM domain and RING finger protein YDR266C** |  | 2 | 48 | 37 |
| 2 | 7 | | Ace | | SE | | SETELR | | 100% Ac | 100% | N.I. | N.D. | P25087 | ERG6_YEAST | | control | **Sterol 24-C-methyltransferase** |  | 1 | 36 | 35 |
| 2 | 12 | | Ace | | SE | | SETNVDSLGDR | | N.D. | N.I. | 100% | N.D. | P51862 | ROM2_YEAST | | hNaa60p | **RHO1 GDP-GTP exchange protein 2** |  | 1 | 48 | 33 |
| 2 | 17 | | Ace | | SE | | SEVAPEEIIENADGSR | | 100% Ac | 100% | 100% | 0% | Q04067 | EIF3G_YEAST | | hNaa60p,control | **Eukaryotic translation initiation factor 3 RNA-binding subunit** |  | 24 | 105 | 34 |
| 2 | 13 | | Ace | | SE | | SEVIEGNVKIDR | | 100% Ac | 100% | 100% | 0% | P20081 | FKBP_YEAST | | control,hNaa60p | **FK506-binding protein 1** |  | 220 | 115 | 37 |
| 2 | 10 | | Ace | | SE | | SEVITITKR | | 100% Ac | 100% | N.I. | N.D. | P38792 | RRP4_YEAST | | control | **Exosome complex exonuclease RRP4** |  | 1 | 43 | 35 |
| 2 | 23 | | Ace | | SE | | SEYASSIHSQMKQFDTKYSGNR | | N.D. | N.I. | 100% | N.D. | Q12402 | YOP1_YEAST | | hNaa60p | **Protein YOP1** |  | 2 | 50 | 34 |
| 2 | 10 | | Ace | | SE | | SEYMDDVDR | | 100% Ac | 100% | 100% | 0% | P53076 | VID30_YEAST | | hNaa60p,control | **Vacuolar import and degradation protein 30** |  | 2 | 46 | 24 |
| 2 | 12 | | Ace | | SF | | SFDDLHKATER | | N.D. | N.D. | 100% | N.D. | P17709 | HXKG_YEAST | | hNaa60p,control | **Glucokinase-1** |  | 2 | 46 | 36 |
| 2 | 13 | | Ace | | SF | | SFFGLENSGNAR | | 100% Ac | 100% | 100% | 0% | P25644 | PAT1_YEAST | | control,hNaa60p | **DNA topoisomerase 2-associated protein PAT1** |  | 5 | 97 | 35 |
| 2 | 47 | | Ace | | SF | | SFIKNLLFGGVKTSEDPTGLTGNGASNTNDSNKGSEPVVAGNFFPR | | N.D. | N.D. | N.I. | N.D. | Q12091 | DAP1_YEAST | | control | **Damage response protein 1** |  | 1 | 37 | 36 |
| 2 | 12 | | Ace | | SF | | SFKGFTKAVSR | | 100% Ac | 100% | 100% | 0% | P39743 | RV167_YEAST | | hNaa60p,control | **Reduced viability upon starvation protein 167** |  | 7 | 72 | 36 |
| 2 | 16 | | AcD3C13 | | SF | | SFLPSFILSDESKER | | partial Ac- | 21% | N.I. | N.D. | P53507 | TOM7_YEAST | | control | **Mitochondrial import receptor subunit TOM7** |  | 1 | 80 | 38 |
| 2 | 11 | | Ace | | SF | | SFLSKLSQIR | | 100% Ac | 100% | N.I. | N.D. | P06843 | SPT2_YEAST | | control | **Protein SPT2** |  | 3 | 74 | 36 |
| 2 | 24 | | Ace | | SF | | SFQIETVPTKPYEDQKPGTSGLR | | 100% Ac | 98% | 98% | 0% | P37012 | PGM2_YEAST | | hNaa60p,control | **Phosphoglucomutase-2** |  | 2 | 55 | 37 |
| 2 | 35 | | Ace | | SF | | SFSNGNMASYMTSSNGEEQSINNKNDIDDNSAYR | | N.D. | N.I. | 58% | N.D. | P53968 | CRZ1_YEAST | | hNaa60p | **Transcriptional regulator CRZ1** |  | 1 | 62 | 22 |
| 2 | 14 | | Ace | | SG | | SGAAAASAAGYDR | | 100% Ac | 100% | 100% | 0% | P21243 | PSA6_YEAST | | control,hNaa60p | **Proteasome component C7-alpha** |  | 6 | 89 | 34 |
| 2 | 10 | | Ace | | SG | | SGELANYKR | | 100% Ac | 100% | N.I. | N.D. | P00546 | CDC28_YEAST | | control | **Cell division control protein 28** |  | 1 | 44 | 37 |
| 2 | 11 | | Ace | | SG | | SGETFEFNIR | | 100% Ac | 100% | 100% | 0% | P43593 | UBP6_YEAST | | hNaa60p,control | **Ubiquitin carboxyl-terminal hydrolase 6** |  | 4 | 51 | 35 |
| 2 | 19 | | Ace | | SG | | SGGKGGKAGSAAKASQSR | | 100% Ac | 100% | 100% | 0% | P04911 | H2A1_YEAST | | hNaa60p,control | **Histone H2A.1** | P04912 (2-19) | 153 | 101 | 37 |
| 2 | 12 | | Ace | | SG | | SGIIDASSALR | | 100% Ac | 100% | 100% | 0% | P32458 | CDC11_YEAST | | hNaa60p,control | **Cell division control protein 11** |  | 4 | 79 | 37 |
| 2 | 12 | | Ace | | SG | | SGNGAQGTKFR | | 100% Ac | 100% | 100% | 0% | P04451 | RL23_YEAST | | control,hNaa60p | **60S ribosomal protein L23** |  | 25 | 72 | 37 |
| 2 | 19 | | Ace | | SG | | SGNQMAMGSEQQQTVGSR | | 100% Ac | 100% | N.D. | N.D. | A7A241 | YPI1_YEAST | | control,hNaa60p | **Type 1 phosphatases regulator YPI1** | P43587 (2-19) | 29 | 125 | 27 |
| 2 | 11 | | Ace | | SG | | SGYTGNNYSR | | N.D. | N.D. | 93% | N.D. | Q02796 | LGE1_YEAST | | control,hNaa60p | **Transcriptional regulatory protein LGE1** |  | 2 | 40 | 30 |
| 2 | 21 | | AcD3C13 | | SI | | SIDNKLFVTEEDEEDRTQDR | | N.D. | N.I. | 29% | N.D. | P36121 | RPC5_YEAST | | hNaa60p | **DNA-directed RNA polymerase III subunit RPC5** |  | 2 | 57 | 35 |
| 2 | 29 | | Ace | | SI | | SIENLKSFDPFADTGDDETATSNYIHIR | | 100% Ac | 100% | N.D. | N.D. | P32911 | SUI1_YEAST | | hNaa60p,control | **Eukaryotic translation initiation factor eIF-1** |  | 5 | 125 | 34 |
| 2 | 20 | | Ace | | SI | | SISSDEAKEKQLVEKAELR | | 100% Ac | 100% | N.I. | N.D. | P38737 | ECM29_YEAST | | control | **Proteasome component ECM29** |  | 1 | 46 | 37 |
| 2 | 17 | | Ace | | SI | | SITKTELDGILPLVAR | | 100% Ac | 100% | 100% | 0% | P27616 | PUR7_YEAST | | hNaa60p,control | **Phosphoribosylaminoimidazole-succinocarboxamide synthase** |  | 4 | 58 | 34 |
| 2 | 12 | | Ace | | SI | | SIVYNKTPLLR | | 100% Ac | 100% | 100% | 0% | P25379 | STDH_YEAST | | hNaa60p,control | **Catabolic L-serine/threonine dehydratase** |  | 2 | 42 | 35 |
| 2 | 20 | | Ace | | SK | | SKAKGTGFSSIDTEDENLR | | 100% Ac | 100% | N.I. | N.D. | P42934 | PMT6_YEAST | | control | **Dolichyl-phosphate-mannose--protein mannosyltransferase 6** |  | 2 | 91 | 37 |
| 2 | 9 | | Ace | | SK | | SKATYKER | | 100% Ac | 100% | N.I. | N.D. | P03962 | PYRF_YEAST | | control | **Orotidine 5'-phosphate decarboxylase** |  | 5 | 44 | 37 |
| 2 | 23 | | Ace | | SK | | SKAVGIDLGTTYSCVAHFANDR | | N.D. | N.I. | 100% | N.D. | P10591 | HSP71_YEAST | | hNaa60p | **Heat shock protein SSA1** | P22202 (2-23) | 1 | 42 | 36 |
| 2 | 9 | | Ace | | SK | | SKDLNSSR | | 100% Ac | 100% | 98% | -2% | P23287 | PP2B1_YEAST | | control,hNaa60p | **Serine/threonine-protein phosphatase 2B catalytic subunit A1** |  | 3 | 64 | 36 |
| 2 | 10 | | Ace | | SK | | SKITSSQVR | | 100% Ac | 100% | 100% | 0% | P53030 | RL1_YEAST | | hNaa60p,control | **60S ribosomal protein L1** |  | 70 | 76 | 36 |
| 2 | 12 | | Ace | | SK | | SKKFSSKNSQR | | 100% Ac | 100% | 100% | 0% | P34078 | LTV1_YEAST | | control,hNaa60p | **Protein LTV1** |  | 3 | 70 | 37 |
| 2 | 11 | | AcD3C13 | | SK | | SKKKETFTPR | | N.D. | N.I. | 21% | N.D. | Q03661 | ESC1_YEAST | | hNaa60p | **Silent chromatin protein ESC1** |  | 1 | 40 | 34 |
| 2 | 10 | | Ace | | SK | | SKKLSLEER | | N.D. | N.I. | 97% | N.D. | P47166 | SGM1_YEAST | | hNaa60p | **Protein SGM1** |  | 1 | 60 | 36 |
| 2 | 11 | | Ace | | SK | | SKNSNVNNNR | | 100% Ac | 100% | 100% | 0% | P24276 | SSD1_YEAST | | control,hNaa60p | **Protein SSD1** |  | 4 | 54 | 36 |
| 2 | 11 | | Ace | | SK | | SKSTNVSYER | | N.D. | N.I. | 100% | N.D. | Q04231 | RAD33_YEAST | | hNaa60p | **DNA repair protein RAD33** |  | 1 | 48 | 37 |
| 2 | 12 | | Ace | | SK | | SKVIKPPGQAR | | 100% Ac | 100% | 100% | 0% | P22138 | RPA2_YEAST | | hNaa60p,control | **DNA-directed RNA polymerase I subunit RPA2** |  | 2 | 70 | 33 |
| 2 | 14 | | Ace | | SK | | SKVMKPSNGKGSR | | 100% Ac | 94% | 94% | 0% | P53628 | SNF12_YEAST | | hNaa60p,control | **Transcription regulatory protein SNF12** |  | 2 | 76 | 37 |
| 2 | 31 | | Ace | | SL | | SLAVLLNKEDKDISDFSKTTAGKSAKKNSR | | N.D. | N.I. | N.D. | N.D. | A6ZU34 | INO80_YEAST | | hNaa60p | **Putative DNA helicase INO80** | P53115 (2-31) | 1 | 82 | 34 |
| 2 | 24 | | Ace | | SL | | SLDFNTLAQNFTQFYYNQFDTDR | | N.D. | N.D. | 100% | N.D. | P33331 | NTF2_YEAST | | control,hNaa60p | **Nuclear transport factor 2** |  | 3 | 76 | 31 |
| 2 | 10 | | Ace | | SL | | SLEAIVFDR | | 100% Ac | 100% | N.I. | N.D. | Q06489 | MTNA_YEAST | | control | **Methylthioribose-1-phosphate isomerase** |  | 3 | 77 | 37 |
| 2 | 18 | | Ace | | SL | | SLEATDSKAMVLLMGVR | | N.D. | N.I. | 100% | N.D. | P53290 | GTR2_YEAST | | hNaa60p | **GTP-binding protein GTR2** |  | 2 | 116 | 37 |
| 2 | 20 | | Ace | | SL | | SLEDTLANMSLYDAKKYFR | | N.D. | N.I. | 100% | N.D. | P47160 | ENT3_YEAST | | hNaa60p | **Epsin-3** |  | 2 | 60 | 37 |
| 2 | 17 | | Ace | | SL | | SLEEFDEVKYDHSTKR | | partial Ac- | 94% | 90% | -4% | Q08920 | NCBP2_YEAST | | control,hNaa60p | **Nuclear cap-binding protein subunit 2** |  | 4 | 98 | 36 |
| 2 | 10 | | Ace | | SL | | SLISAVEDR | | 100% Ac | 100% | 100% | 0% | Q05050 | YMS1_YEAST | | control,hNaa60p | **Uncharacterized protein YMR031C** |  | 2 | 52 | 37 |
| 2 | 19 | | Ace | | SL | | SLISILSPLITSEGLDSR | | N.D. | N.I. | 79% | N.D. | P53154 | GUP1_YEAST | | hNaa60p | **Glycerol uptake protein 1** |  | 1 | 104 | 37 |
| 2 | 14 | | Ace | | SL | | SLKEDDFGKDNSR | | 100% Ac | 100% | 100% | 0% | P18412 | TEC1_YEAST | | control,hNaa60p | **Ty transcription activator TEC1** |  | 2 | 62 | 36 |
| 2 | 19 | | Ace | | SL | | SLKEEQVSIKQDPEQEER | | 100% Ac | 100% | 100% | 0% | P32457 | CDC3_YEAST | | control,hNaa60p | **Cell division control protein 3** |  | 5 | 95 | 37 |
| 2 | 24 | | Ace | | SL | | SLLIDSVPTVAYKDQKPGTSGLR | | 100% Ac | 100% | N.I. | N.D. | P33401 | PGM1_YEAST | | control | **Phosphoglucomutase-1** |  | 2 | 55 | 36 |
| 2 | 27 | | Ace | | SL | | SLPATFDLTPEDAQLLLAANTHLGAR | | partial Ac- | 97% | N.I. | N.D. | P32905 | RS0A_YEAST | | control | **40S ribosomal protein S0-A** | P46654 (2-27) | 1 | 80 | 37 |
| 2 | 10 | | Ace | | SL | | SLPDGFYIR | | 100% Ac | 100% | N.I. | N.D. | P43577 | GNA1_YEAST | | control | **Glucosamine 6-phosphate N-acetyltransferase** |  | 1 | 71 | 37 |
| 2 | 12 | | Ace | | SL | | SLPGSKLEEAR | | N.D. | N.I. | 100% | N.D. | Q12377 | RPN6_YEAST | | hNaa60p | **26S proteasome regulatory subunit RPN6** |  | 2 | 67 | 38 |
| 2 | 14 | | Ace | | SL | | SLQLLNPKAESLR | | 100% Ac | 100% | 100% | 0% | P39079 | TCPZ_YEAST | | hNaa60p,control | **T-complex protein 1 subunit zeta** |  | 4 | 68 | 36 |
| 2 | 19 | | Ace | | SL | | SLQSNSVKPTEIPLSEIR | | 100% Ac | 100% | 100% | 0% | P36077 | SRX1_YEAST | | hNaa60p,control | **Sulfiredoxin** |  | 5 | 70 | 37 |
| 2 | 12 | | Ace | | SL | | SLSNKTVKELR | | N.D. | N.I. | 100% | N.D. | A6ZXB7 | CSN5_YEAST | | hNaa60p | **COP9 signalosome complex subunit 5** | B3LH96 (2-12)^AQ12468 (2-12) | 1 | 48 | 34 |
| 2 | 18 | | Ace | | SL | | SLSSKLSVQDLDLKDKR | | 100% Ac | 100% | 100% | 0% | P00560 | PGK_YEAST | | hNaa60p,control | **Phosphoglycerate kinase** |  | 57 | 102 | 36 |
| 2 | 26 | | Ace | | SL | | SLSVAEKSYLYDSLASTPSIRPDGR | | N.D. | N.I. | 100% | N.D. | Q12277 | RRP42_YEAST | | hNaa60p | **Exosome complex component RRP42** |  | 1 | 44 | 37 |
| 2 | 14 | | Ace | | SL | | SLTTAAPLLALLR | | 100% Ac | 100% | 100% | 0% | P32565 | RPN2_YEAST | | control,hNaa60p | **26S proteasome regulatory subunit RPN2** |  | 12 | 64 | 32 |
| 2 | 16 | | Ace | | SL | | SLVVQEQGSFQHILR | | N.D. | N.I. | 100% | N.D. | P35271 | RS18_YEAST | | hNaa60p | **40S ribosomal protein S18** |  | 1 | 68 | 38 |
| 2 | 11 | | Ace | | SM | | SMQIEIKDGR | | 100% Ac | 100% | 100% | 0% | P40045 | YEQ1_YEAST | | control,hNaa60p | **Uncharacterized protein YER071C** |  | 9 | 58 | 37 |
| 2 | 11 | | Ace | | SM | | SMSSTVIKFR | | 100% Ac | 100% | 100% | 0% | O13563 | RPN13_YEAST | | control,hNaa60p | **26S proteasome regulatory subunit RPN13** |  | 5 | 62 | 37 |
| 2 | 29 | | Ace | | SN | | SNDEGETFATEQTTQQVFQKLGSNMENR | | partial Ac- | 93% | 93% | 0% | P38682 | GLO3_YEAST | | control,hNaa60p | **ADP-ribosylation factor GTPase-activating protein GLO3** |  | 2 | 50 | 30 |
| 2 | 15 | | Ace | | SN | | SNDEINQNEEKVKR | | N.D. | N.D. | N.D. | N.D. | Q12400 | TRM10_YEAST | | control,hNaa60p | **tRNA (guanine-N(1)-)-methyltransferase TRM10** |  | 2 | 80 | 37 |
| 2 | 27 | | Ace | | SN | | SNDPSAVSELPDKDSLDNGISNDNER | | N.D. | N.D. | N.I. | N.D. | Q03497 | STE20_YEAST | | control | **Serine/threonine-protein kinase STE20** |  | 1 | 85 | 32 |
| 2 | 11 | | Ace | | SN | | SNIVGIEYNR | | 100% Ac | 100% | N.I. | N.D. | P07703 | RPAC1_YEAST | | control | **DNA-directed RNA polymerases I and III subunit RPAC1** |  | 1 | 86 | 37 |
| 2 | 11 | | Ace | | SN | | SNKASDQSAR | | 100% Ac | 100% | 100% | 0% | P17123 | SPO12_YEAST | | control,hNaa60p | **Sporulation-specific protein 12** |  | 2 | 59 | 36 |
| 2 | 7 | | Ace | | SN | | SNKLFR | | 100% Ac | 100% | 100% | 0% | P29952 | MPI_YEAST | | control,hNaa60p | **Mannose-6-phosphate isomerase** |  | 4 | 44 | 36 |
| 2 | 12 | | Ace | | SN | | SNLYKIGTETR | | 100% Ac | 100% | 100% | 0% | Q12156 | YD063_YEAST | | control,hNaa60p | **Uncharacterized protein YDR063W** |  | 6 | 75 | 37 |
| 2 | 18 | | Ace | | SN | | SNPIENTENSQNTSSSR | | 100% Ac | 100% | 100% | 0% | P24869 | CG22_YEAST | | control,hNaa60p | **G2/mitotic-specific cyclin-2** |  | 2 | 67 | 32 |
| 2 | 14 | | Ace | | SN | | SNQALYEKLEQTR | | 100% Ac | 100% | 100% | 0% | P32570 | MED22_YEAST | | hNaa60p,control | **Mediator of RNA polymerase II transcription subunit 22** |  | 2 | 81 | 37 |
| 2 | 8 | | Ace | | SN | | SNSLEER | | 100% Ac | 100% | N.I. | N.D. | P36080 | RRP14_YEAST | | control | **Ribosomal RNA-processing protein 14** |  | 1 | 62 | 35 |
| 2 | 19 | | Ace | | SN | | SNTLFDDIFQVSEVDPGR | | partial Ac- | 97% | 100% | 3% | P20436 | RPAB3_YEAST | | hNaa60p,control | **DNA-directed RNA polymerases I, II, and III subunit RPABC3** |  | 4 | 109 | 35 |
| 2 | 8 | | Ace | | SN | | SNVVQAR | | 100% Ac | 100% | 100% | 0% | P06367 | RS14A_YEAST | | hNaa60p,control | **40S ribosomal protein S14-A** |  | 40 | 64 | 36 |
| 2 | 16 | | AcD3C13 | | SP | | SPLNVGIVGTGIFAR | | 100% free | 0% | 0% | 0% | Q04869 | YM94_YEAST | | control,hNaa60p | **Uncharacterized protein YMR315W** |  | 3 | 47 | 35 |
| 2 | 17 | | AcD3C13 | | SP | | SPMKVAVVGASGKVGR | | 100% free | 0% | 0% | 0% | Q04304 | YMY0_YEAST | | hNaa60p,control | **UPF0659 protein YMR090W** |  | 11 | 87 | 35 |
| 2 | 11 | | AcD3C13 | | SP | | SPPVYSDISR | | 100% free | 0% | N.I. | N.D. | P04840 | VDAC1_YEAST | | control | **Mitochondrial outer membrane protein porin 1** |  | 2 | 47 | 37 |
| 2 | 10 | | AcD3C13 | | SP | | SPSPSVSPR | | 100% free | 0% | N.I. | N.D. | Q03208 | YML9_YEAST | | control | **Uncharacterized protein YML119W** |  | 1 | 37 | 36 |
| 2 | 10 | | Ace | | SQ | | SQAVNAKKR | | 100% Ac | 100% | 100% | 0% | Q01855 | RS15_YEAST | | hNaa60p,control | **40S ribosomal protein S15** |  | 183 | 72 | 35 |
| 2 | 45 | | Ace | | SQ | | SQDENIVKAVEESAEPAQVILGEDGKPLSKKALKKLQKEQEKQR | | partial Ac- | 83% | N.I. | N.D. | P04802 | SYDC_YEAST | | control | **Aspartyl-tRNA synthetase, cytoplasmic** |  | 1 | 45 | 32 |
| 2 | 20 | | Ace | | SQ | | SQDFVTLVSKDDKEYEISR | | partial Ac- | 97% | 100% | 3% | Q03071 | ELOC_YEAST | | hNaa60p,control | **Elongin-C** |  | 2 | 86 | 37 |
| 2 | 32 | | Ace | | SQ | | SQESSVLSESQEQLANNPKIEDTSPPSANSR | | N.D. | N.I. | N.D. | N.D. | Q08561 | IES4_YEAST | | hNaa60p | **Ino eighty subunit 4** |  | 1 | 57 | 35 |
| 2 | 21 | | Ace | | SQ | | SQFEKQKEQGNSLFKQGLYR | | 100% Ac | 100% | 100% | 0% | P25638 | TAH1_YEAST | | control,hNaa60p | **TPR repeat-containing protein associated with Hsp90** |  | 3 | 96 | 38 |
| 2 | 17 | | Ace | | SQ | | SQGQSKKLDVTVEQLR | | 100% Ac | 100% | 100% | 0% | Q12143 | NSL1_YEAST | | hNaa60p,control | **Kinetochore-associated protein NSL1** |  | 4 | 105 | 37 |
| 2 | 11 | | Ace | | SQ | | SQGTLYANFR | | 100% Ac | 100% | 100% | 0% | P29547 | EF1G1_YEAST | | control,hNaa60p | **Elongation factor 1-gamma 1** |  | 16 | 83 | 36 |
| 2 | 10 | | Ace | | SQ | | SQGTLYINR | | 100% Ac | 100% | 100% | 0% | P36008 | EF1G2_YEAST | | hNaa60p,control | **Elongation factor 1-gamma 2** |  | 64 | 73 | 37 |
| 2 | 13 | | Ace | | SQ | | SQIAQEMTVSLR | | 100% Ac | 100% | 100% | 0% | P46988 | PFD1_YEAST | | control,hNaa60p | **Prefoldin subunit 1** |  | 53 | 103 | 37 |
| 2 | 14 | | Ace | | SQ | | SQIVDFVEDKDSR | | 100% Ac | 100% | N.I. | N.D. | P38257 | MMS4_YEAST | | control | **Crossover junction endonuclease MMS4** |  | 1 | 68 | 36 |
| 2 | 19 | | Ace | | SQ | | SQKDNLLDNPVEFLKEVR | | 100% Ac | 100% | 100% | 0% | Q04477 | SPC24_YEAST | | hNaa60p,control | **Kinetochore protein SPC24** |  | 3 | 88 | 37 |
| 2 | 25 | | Ace | | SQ | | SQKNGIATLLQAEKEAHEIVSKAR | | partial Ac- | 96% | 100% | 4% | P48836 | VATG_YEAST | | hNaa60p,control | **V-type proton ATPase subunit G** |  | 21 | 117 | 36 |
| 2 | 14 | | Ace | | SQ | | SQLPTDFASLIKR | | 100% Ac | 100% | N.I. | N.D. | Q06151 | DCPS_YEAST | | control | **Scavenger mRNA-decapping enzyme DcpS** |  | 1 | 45 | 37 |
| 2 | 14 | | Ace | | SQ | | SQNNTISSMNPER | | 100% Ac | 100% | 100% | 0% | Q9URQ5 | HTL1_YEAST | | hNaa60p,control | **High temperature lethal protein 1** |  | 8 | 80 | 32 |
| 2 | 12 | | Ace | | SQ | | SQNVYIVSTAR | | 100% Ac | 100% | 100% | 0% | P41338 | THIL_YEAST | | hNaa60p,control | **Acetyl-CoA acetyltransferase** |  | 10 | 64 | 37 |
| 2 | 17 | | Ace | | SQ | | SQPVVVIDAKDHLLGR | | 100% Ac | 99% | 97% | -2% | P26785 | RL16B_YEAST | | hNaa60p,control | **60S ribosomal protein L16-B** |  | 59 | 120 | 36 |
| 2 | 10 | | Ace | | SQ | | SQQVGNSIR | | 100% Ac | 100% | 99% | -1% | P06780 | RHO1_YEAST | | hNaa60p,control | **GTP-binding protein RHO1** |  | 4 | 52 | 37 |
| 2 | 15 | | Ace | | SQ | | SQVNTSQGPVAQGR | | 100% Ac | 100% | 100% | 0% | P32356 | TREA_YEAST | | hNaa60p,control | **Neutral trehalase** |  | 7 | 101 | 36 |
| 2 | 29 | | Ace | | SQ | | SQVQSPLTATNSGLAVNNNTMNSQMPNR | | partial Ac- | 75% | N.D. | N.D. | P23292 | KC12_YEAST | | control,hNaa60p | **Casein kinase I homolog 2** |  | 6 | 93 | 33 |
| 2 | 17 | | Ace | | SQ | | SQVYFDVEADGQPIGR | | 100% Ac | 100% | 100% | 0% | P14832 | CYPH_YEAST | | hNaa60p,control | **Peptidyl-prolyl cis-trans isomerase** |  | 269 | 136 | 35 |
| 2 | 18 | | Ace | | SQ | | SQYIGKTISLISVTDNR | | 100% Ac | 100% | 100% | 0% | P45978 | SCD6_YEAST | | control,hNaa60p | **Protein SCD6** |  | 6 | 89 | 37 |
| 2 | 33 | | Ace | | SS | | SSAAEKKPASKAPAEKKPAAKKTSTSVDGKKR | | 100% Ac | 100% | 98% | -2% | P02294 | H2B2_YEAST | | hNaa60p,control | **Histone H2B.2** |  | 6 | 59 | 29 |
| 2 | 25 | | Ace | | SS | | SSAITALTPNQVNDELNKMQAFIR | | partial Ac- | 89% | 94% | 5% | P22203 | VATE_YEAST | | hNaa60p,control | **V-type proton ATPase subunit E** |  | 8 | 85 | 37 |
| 2 | 19 | | Ace | | SS | | SSCKTTLSEMVGSVTKDR | | 100% Ac | 100% | 100% | 0% | P05374 | PEM1_YEAST | | hNaa60p,control | **Phosphatidylethanolamine N-methyltransferase** |  | 3 | 60 | 36 |
| 2 | 19 | | Ace | | SS | | SSEIAYSNTNTNTENENR | | 100% Ac | 100% | 98% | -2% | P38956 | SNF11_YEAST | | control,hNaa60p | **Transcription regulatory protein SNF11** |  | 2 | 145 | 29 |
| 2 | 12 | | Ace | | SS | | SSGLVLENTAR | | 100% Ac | 100% | 100% | 0% | P26637 | SYLC_YEAST | | hNaa60p,control | **Leucyl-tRNA synthetase, cytoplasmic** |  | 11 | 83 | 37 |
| 2 | 15 | | Ace | | SS | | SSGSTIVSSDKSGR | | 100% Ac | 100% | N.I. | N.D. | P40017 | YEL4_YEAST | | control | **Putative uncharacterized O-acetyltransferase YER024W** |  | 1 | 85 | 37 |
| 2 | 18 | | Ace | | SS | | SSISLKEIIPPQPSTQR | | 100% Ac | 100% | 100% | 0% | P46680 | AIP1_YEAST | | hNaa60p,control | **Actin-interacting protein 1** |  | 2 | 43 | 37 |
| 2 | 11 | | Ace | | SS | | SSITSETGKR | | 100% Ac | 100% | N.I. | N.D. | P20095 | PRP2_YEAST | | control | **Pre-mRNA-splicing factor ATP-dependent RNA helicase-like protein PRP2** |  | 1 | 60 | 37 |
| 2 | 11 | | Ace | | SS | | SSIVNKSGTR | | 100% Ac | 100% | N.I. | N.D. | P46678 | TFC5_YEAST | | control | **Transcription factor TFIIIB component B''** |  | 1 | 45 | 37 |
| 2 | 32 | | Ace | | SS | | SSIYKALAGKSKDNKSEKKQGNVKQFMNKQR | | N.D. | N.I. | 100% | N.D. | Q08235 | BRX1_YEAST | | hNaa60p | **Ribosome biogenesis protein BRX1** |  | 1 | 69 | 34 |
| 2 | 16 | | Ace | | SS | | SSKPDTGSEISGPQR | | 100% Ac | 100% | 100% | 0% | P08678 | CYAA_YEAST | | hNaa60p,control | **Adenylate cyclase** |  | 2 | 73 | 37 |
| 2 | 12 | | Ace | | SS | | SSKSEKLEKLR | | 100% Ac | 100% | N.D. | N.D. | P13382 | DPOA_YEAST | | control,hNaa60p | **DNA polymerase alpha catalytic subunit A** |  | 4 | 72 | 34 |
| 2 | 8 | | Ace | | SS | | SSLCLQR | | N.D. | N.I. | 100% | N.D. | P50623 | UBC9_YEAST | | hNaa60p | **SUMO-conjugating enzyme UBC9** |  | 1 | 41 | 36 |
| 2 | 22 | | Ace | | SS | | SSLSDQLAQVASNNATVALDR | | 100% Ac | 100% | 99% | -1% | P42945 | UTP10_YEAST | | hNaa60p,control | **U3 small nucleolar RNA-associated protein 10** |  | 4 | 99 | 37 |
| 2 | 38 | | Ace | | SS | | SSNLTEEQIAEFKEAFALFDKDNNGSISSSELATVMR | | partial Ac- | 65% | N | N.D. | P06787 | CALM_YEAST | | hNaa60p,control | **Calmodulin** |  | 2 | 60 | 34 |
| 2 | 21 | | Ace | | SS | | SSNNSGLSAAGEIDESLYSR | | N.D. | N.D. | 100% | N.D. | P22515 | UBA1_YEAST | | control,hNaa60p | **Ubiquitin-activating enzyme E1 1** |  | 22 | 133 | 32 |
| 2 | 36 | | Ace | | SS | | SSNPENSGVNANNNTGTGNADAITGAQQNMVLQPR | | N.D. | N.D. | N.D. | N.D. | Q03761 | TAF12_YEAST | | control,hNaa60p | **Transcription initiation factor TFIID subunit 12** |  | 3 | 67 | 32 |
| 2 | 13 | | Ace | | SS | | SSNTSSVMSSPR | | 100% Ac | 100% | 100% | 0% | P20676 | NUP1_YEAST | | hNaa60p,control | **Nucleoporin NUP1** |  | 8 | 70 | 32 |
| 2 | 36 | | Ace | | SS | | SSPVIGITFGNTSSSIAYINPKNDVDVIANPDGER | | N.D. | N.I. | 89% | N.D. | P38788 | SSZ1_YEAST | | hNaa60p | **Ribosome-associated complex subunit SSZ1** |  | 2 | 42 | 36 |
| 2 | 11 | | Ace | | SS | | SSQPSFVTIR | | 100% Ac | 100% | 100% | 0% | P38736 | GOS1_YEAST | | control,hNaa60p | **Protein transport protein GOS1** |  | 2 | 53 | 38 |
| 2 | 9 | | Ace | | SS | | SSSAIKIR | | 100% Ac | 100% | N.I. | N.D. | P38753 | HSE1_YEAST | | control | **Class E vacuolar protein-sorting machinery protein HSE1** |  | 1 | 37 | 35 |
| 2 | 18 | | Ace | | SS | | SSSAPKYTTFQGSQNFR | | 100% Ac | 100% | 100% | 0% | Q08096 | RCL1_YEAST | | hNaa60p,control | **RNA 3'-terminal phosphate cyclase-like protein** |  | 5 | 107 | 36 |
| 2 | 17 | | Ace | | SS | | SSSDIFDVLNIKQKSR | | 100% Ac | 100% | 100% | 0% | P53201 | SWC4_YEAST | | hNaa60p,control | **SWR1-complex protein 4** |  | 2 | 110 | 37 |
| 2 | 8 | | Ace | | SS | | SSSESIR | | 100% Ac | 100% | N.I. | N.D. | P07170 | KAD1_YEAST | | control | **Adenylate kinase cytosolic** |  | 2 | 54 | 34 |
| 2 | 15 | | Ace | | SS | | SSSIFGPLTGFLER | | N.D. | N.I. | 100% | N.D. | P53723 | YN8B_YEAST | | hNaa60p | **UPF0674 endoplasmic reticulum membrane protein YNR021W** |  | 1 | 49 | 37 |
| 2 | 23 | | Ace | | SS | | SSSKFEEVINKIINDSPPGELR | | 100% Ac | 100% | N.D. | N.D. | P28495 | CAPZA_YEAST | | hNaa60p,control | **F-actin-capping protein subunit alpha** |  | 7 | 51 | 37 |
| 2 | 11 | | Ace | | SS | | SSSKKLAGLR | | 100% Ac | 100% | 100% | 0% | P32327 | PYC2_YEAST | | hNaa60p,control | **Pyruvate carboxylase 2** |  | 4 | 69 | 34 |
| 2 | 12 | | Ace | | SS | | SSSQQIAKNAR | | 100% Ac | 100% | 100% | 0% | P54885 | PROA_YEAST | | hNaa60p,control | **Gamma-glutamyl phosphate reductase** |  | 13 | 76 | 37 |
| 2 | 16 | | Ace | | SS | | SSSSSSSESSPNLSR | | 100% Ac | 100% | 100% | 0% | Q08269 | ALR1_YEAST | | hNaa60p,control | **Magnesium transporter ALR1** |  | 2 | 80 | 32 |
| 2 | 24 | | Ace | | SS | | SSSSSTGYSKNNAAHIKQENTLR | | N.D. | N.I. | 100% | N.D. | P31382 | PMT2_YEAST | | hNaa60p | **Dolichyl-phosphate-mannose--protein mannosyltransferase 2** |  | 1 | 59 | 36 |
| 2 | 21 | | Ace | | SS | | SSTQDSKAQTLNSNPEILLR | | 100% Ac | 100% | 100% | 0% | P40693 | RLP7_YEAST | | hNaa60p,control | **Ribosome biogenesis protein RLP7** |  | 6 | 87 | 38 |
| 2 | 8 | | Ace | | SS | | SSVQLSR | | N.D. | N.D. | N.I. | N.D. | P22336 | RFA1_YEAST | | control | **Replication factor A protein 1** |  | 1 | 65 | 36 |
| 2 | 22 | | Ace | | ST | | STAEFAQLLENSILSPDQNIR | | 100% Ac | 98% | 100% | 2% | Q06142 | IMB1_YEAST | | hNaa60p,control | **Importin subunit beta-1** |  | 5 | 91 | 37 |
| 2 | 12 | | Ace | | ST | | STDTMYFNSSR | | 100% Ac | 100% | N.D. | N.D. | P53080 | EDC1_YEAST | | control,hNaa60p | **Enhancer of mRNA-decapping protein 1** |  | 7 | 64 | 27 |
| 2 | 17 | | Ace | | ST | | STEEQNGGGQKSLDDR | | 100% Ac | 100% | N.I. | N.D. | P06244 | KAPA_YEAST | | control,hNaa60p | **cAMP-dependent protein kinase type 1** |  | 3 | 95 | 32 |
| 2 | 13 | | Ace | | ST | | STEEQNGVPLQR | | N.D. | N.I. | 100% | N.D. | P21965 | MCK1_YEAST | | hNaa60p | **Protein kinase MCK1** |  | 1 | 57 | 36 |
| 2 | 11 | | Ace | | ST | | STELTVQSER | | 100% Ac | 100% | 100% | 0% | P26781 | RS11_YEAST | | hNaa60p,control | **40S ribosomal protein S11** |  | 69 | 82 | 36 |
| 2 | 9 | | Ace | | ST | | STFGKLFR | | 100% Ac | 100% | 100% | 0% | P28777 | AROC_YEAST | | control,hNaa60p | **Chorismate synthase** |  | 5 | 52 | 37 |
| 2 | 13 | | Ace | | ST | | STFSASDFNSER | | 100% Ac | 100% | N.I. | N.D. | A6ZRD1 | TMT1_YEAST | | control | **Trans-aconitate 3-methyltransferase** | P32643 (2-13) | 2 | 72 | 30 |
| 2 | 11 | | Ace | | ST | | STGITYDEDR | | 100% Ac | 100% | 100% | 0% | P38197 | YBD6_YEAST | | hNaa60p,control | **UPF0001 protein YBL036C** |  | 3 | 60 | 31 |
| 2 | 16 | | Ace | | ST | | STIKPSPSNNNLKVR | | 100% Ac | 100% | 100% | 0% | A6ZZV7 | LAC1_YEAST | | control,hNaa60p | **Sphingosine N-acyltransferase LAC1** | P28496 (2-16) | 3 | 50 | 36 |
| 2 | 14 | | Ace | | ST | | STISTTTAPEFIR | | 100% Ac | 100% | 100% | 0% | Q07532 | IWR1_YEAST | | control,hNaa60p | **Putative transcription factor IWR1** |  | 2 | 73 | 37 |
| 2 | 10 | | Ace | | ST | | STKAQNPMR | | 100% Ac | 100% | 100% | 0% | Q3E757 | RL11B_YEAST | | control,hNaa60p | **60S ribosomal protein L11-B** |  | 42 | 68 | 37 |
| 2 | 19 | | Ace | | ST | | STLAEVYTIIEDAEQECR | | 100% Ac | 100% | 100% | 0% | Q05789 | YL211_YEAST | | hNaa60p,control | **Uncharacterized protein YLR211C** |  | 2 | 92 | 34 |
| 2 | 20 | | Ace | | ST | | STLIPPPSKKQKKEAQLPR | | 100% Ac | 100% | N.I. | N.D. | P25382 | YCW2_YEAST | | control | **WD repeat-containing protein YCR072C** |  | 1 | 52 | 31 |
| 2 | 17 | | Ace | | ST | | STLLKSAKSIVPLMDR | | 100% Ac | 100% | 100% | 0% | P38910 | CH10_YEAST | | hNaa60p,control | **10 kDa heat shock protein, mitochondrial** |  | 26 | 94 | 35 |
| 2 | 12 | | Ace | | ST | | STMSTPAAEQR | | 100% Ac | 100% | 100% | 0% | Q07508 | LUC7_YEAST | | control,hNaa60p | **Protein LUC7** |  | 6 | 57 | 33 |
| 2 | 22 | | Ace | | ST | | STNFEKHFQENVDECTLEQLR | | N.D. | N.I. | 100% | N.D. | P47120 | DOHH_YEAST | | hNaa60p | **Deoxyhypusine hydroxylase** |  | 1 | 55 | 33 |
| 2 | 20 | | Ace | | ST | | STPFGLDLGNNNSVLAVAR | | 100% Ac | 100% | 100% | 0% | P32589 | HSP7F_YEAST | | control,hNaa60p | **Heat shock protein homolog SSE1** | P32590 (2-20) | 43 | 110 | 38 |
| 2 | 12 | | Ace | | ST | | STTASTPSSLR | | 100% Ac | 100% | N.I. | N.D. | P05150 | OTC_YEAST | | control | **Ornithine carbamoyltransferase** |  | 1 | 46 | 37 |
| 2 | 17 | | Ace | | ST | | STTVEKIKAIEDEMAR | | 100% Ac | 100% | 100% | 0% | P39729 | RBG1_YEAST | | control,hNaa60p | **GTP-binding protein RBG1** |  | 15 | 87 | 38 |
| 2 | 8 | | Ace | | ST | | STVNVLR | | 100% Ac | 100% | N.I. | N.D. | P81449 | ATPJ_YEAST | | control | **ATP synthase subunit e, mitochondrial** |  | 1 | 41 | 35 |
| 2 | 10 | | Ace | | SV | | SVEDIKKAR | | 100% Ac | 100% | N.D. | N.D. | P38088 | SYG_YEAST | | control,hNaa60p | **Glycyl-tRNA synthetase 1** |  | 7 | 56 | 36 |
| 2 | 8 | | Ace | | SV | | SVEEKKR | | 100% Ac | 100% | N.I. | N.D. | Q03435 | NHP10_YEAST | | control | **Non-histone protein 10** |  | 2 | 47 | 37 |
| 2 | 18 | | Ace,AcD3C13 | | SV | | SVEPVVVIDGKGHLVGR | | partial Ac- | 85% | 85% | -1% | P26784 | RL16A_YEAST | | hNaa60p,control | **60S ribosomal protein L16-A** |  | 13 | 99 | 35 |
| 2 | 15 | | Ace | | SV | | SVKPIDNYITNSVR | | 100% Ac | 100% | N.I. | N.D. | P32342 | SRP21_YEAST | | control | **Signal recognition particle subunit SRP21** |  | 1 | 49 | 38 |
| 2 | 14 | | Ace | | SV | | SVQIFGDQVTEER | | 100% Ac | 99% | 100% | 1% | P39076 | TCPB_YEAST | | hNaa60p,control | **T-complex protein 1 subunit beta** |  | 14 | 99 | 36 |
| 2 | 10 | | Ace | | SV | | SVSEQDPNR | | 100% Ac | 100% | N.I. | N.D. | Q07458 | RXT3_YEAST | | control | **Transcriptional regulatory protein RXT3** |  | 1 | 54 | 33 |
| 2 | 12 | | Ace | | SV | | SVSLEQTLGFR | | N.D. | N.I. | 100% | N.D. | P38828 | LSM12_YEAST | | hNaa60p | **Protein LSM12** |  | 1 | 69 | 37 |
| 2 | 8 | | Ace | | SV | | SVSTAKR | | N.D. | N.I. | N.D. | N.D. | P19880 | YAP1_YEAST | | hNaa60p | **AP-1-like transcription factor YAP1** |  | 1 | 37 | 35 |
| 2 | 13 | | Ace | | SV | | SVSTASTEMSVR | | 100% Ac | 100% | 100% | 0% | P53686 | HST2_YEAST | | hNaa60p,control | **NAD-dependent deacetylase HST2** |  | 6 | 76 | 34 |
| 2 | 17 | | Ace | | SY | | SYDKKADVTSLDEDLR | | 100% Ac | 100% | 100% | 0% | Q08873 | SCP1_YEAST | | hNaa60p,control | **Transgelin** |  | 4 | 88 | 37 |
| 2 | 19 | | Ace | | SY | | SYLPTYSNDLPAGPQGQR | | 100% Ac | 100% | N.I. | N.D. | P36125 | GMH1_YEAST | | control | **Protein GMH1** |  | 1 | 64 | 36 |
| 2 | 19 | | Ace | | SY | | SYNPYAYATSEQNGVNDR | | 100% Ac | 100% | N.D. | N.D. | P53845 | YIF1_YEAST | | control,hNaa60p | **Protein transport protein YIF1** |  | 6 | 114 | 28 |
| 2 | 10 | | Ace | | SY | | SYNYLKAAR | | N.D. | N.I. | N.D. | N.D. | P53889 | YNQ8_YEAST | | hNaa60p | **Uncharacterized hydrolase YNL168C** |  | 1 | 65 | 37 |
| 2 | 15 | | Ace | | SY | | SYSAADNLQDSFQR | | 100% Ac | 100% | 100% | 0% | P40510 | SER33_YEAST | | control,hNaa60p | **D-3-phosphoglycerate dehydrogenase 2** |  | 3 | 92 | 32 |
| 2 | 12 | | Ace | | SY | | SYTDNPPQTKR | | 100% Ac | 100% | 100% | 0% | O13297 | CET1_YEAST | | hNaa60p,control | **mRNA-capping enzyme subunit beta** |  | 3 | 77 | 36 |
| **22. Thr-** | | | | | | | | | | | | | | | | | | | |  |  |
| 2 | 9 | | AcD3C13 | | TA | | TAEDILLR | | N.D. | N.I. | 13% | N.D. | P47041 | BIT61_YEAST | | hNaa60p | **Target of rapamycin complex 2 subunit BIT61** |  | 1 | 42 | 37 |
| 2 | 16 | | AcD3C13 | | TA | | TASTPSNVMTLFLLR | | N.D. | N.I. | 27% | N.D. | Q12008 | PMG2_YEAST | | hNaa60p | **Phosphoglycerate mutase 2** |  | 1 | 53 | 37 |
| 2 | 20 | | Ace | | TA | | TASVSNTQNKLNELLDAIR | | partial Ac- | 78% | 78% | 1% | P16649 | TUP1_YEAST | | control,hNaa60p | **General transcriptional corepressor TUP1** |  | 4 | 65 | 37 |
| 2 | 14 | | AcD3C13 | | TA | | TATSDKVLKIQLR | | 100% free | 0% | 0% | 0% | P33317 | DUT_YEAST | | hNaa60p,control | **Deoxyuridine 5'-triphosphate nucleotidohydrolase** |  | 6 | 49 | 31 |
| 2 | 20 | | AcD3C13 | | TA | | TAVTDIIDELNDSSLSSTR | | 100% free | 0% | N.I. | N.D. | P47168 | TTI2_YEAST | | control | **TEL2-interacting protein 2** |  | 1 | 44 | 37 |
| 2 | 22 | | Ace | | TD | | TDELKSYEALKAELKKSLQDR | | N.D. | N.D. | N.D. | N.D. | P47128 | EAF6_YEAST | | control,hNaa60p | **Chromatin modification-related protein EAF6** |  | 4 | 100 | 36 |
| 2 | 19 | | Ace | | TD | | TDPHLNTPQVSTSPTFER | | partial Ac- | 75% | N.I. | N.D. | P43598 | YFI7_YEAST | | control | **Uncharacterized protein YFR017C** |  | 1 | 75 | 36 |
| 2 | 16 | | Ace | | TD | | TDYILPGPKALSQFR | | 100% Ac | 100% | N.I. | N.D. | P38972 | PUR4_YEAST | | control | **Phosphoribosylformylglycinamidine synthase** |  | 1 | 45 | 37 |
| 2 | 11 | | Ace,AcD3C13 | | TE | | TEDKSQVKIR | | N.D. | N.D. | N.D. | N.D. | Q12024 | YTM1_YEAST | | hNaa60p,control | **Microtubule-associated protein YTM1** |  | 5 | 52 | 36 |
| 2 | 18 | | Ace,AcD3C13 | | TE | | TEFELPPKYITAANDLR | | partial Ac- | 59% | N.D. | N.D. | P37303 | GLY1_YEAST | | control,hNaa60p | **Low specificity L-threonine aldolase** |  | 10 | 79 | 37 |
| 2 | 19 | | AcD3C13 | | TE | | TEFKAGSAKKGATLFKTR | | 100% free | 0% | 0% | 0% | P00044 | CYC1_YEAST | | control,hNaa60p | **Cytochrome c iso-1** |  | 3 | 60 | 33 |
| 2 | 19 | | AcD3C13 | | TE | | TELDYQGTAEAASTSYSR | | N.D. | N.I. | 10% | N.D. | P53224 | ORM1_YEAST | | hNaa60p | **Protein ORM1** |  | 1 | 60 | 33 |
| 2 | 20 | | Ace | | TE | | TEPALSSANNALMQKLTGR | | 100% Ac | 100% | 100% | 0% | P53759 | DUS1_YEAST | | control,hNaa60p | **tRNA-dihydrouridine synthase 1** |  | 5 | 135 | 37 |
| 2 | 13 | | AcD3C13 | | TF | | TFMQQLQEAGER | | 100% free | 0% | 0% | 0% | A6ZLA1 | MKAR_YEAST | | hNaa60p,control | **3-ketoacyl-CoA reductase** | B3LN00 (2-13)^AP38286 (2-13) | 9 | 73 | 35 |
| 2 | 19 | | AcD3C13 | | TG | | TGAATAAENSATQLEFYR | | 100% free | 0% | 0% | 0% | Q12517 | DCP1_YEAST | | hNaa60p,control | **mRNA-decapping enzyme subunit 1** |  | 4 | 112 | 36 |
| 2 | 19 | | AcD3C13 | | TG | | TGMESGENLENMEDILAR | | 100% free | 0% | 2% | 2% | P38747 | OTU2_YEAST | | control,hNaa60p | **OTU domain-containing protein 2** |  | 15 | 113 | 31 |
| 2 | 17 | | AcD3C13 | | TG | | TGMNDNNAAIPQQTPR | | 100% free | 0% | 0% | 0% | P25333 | HAL4_YEAST | | control | **Serine/threonine-protein kinase HAL4/SAT4** |  | 1 | 48 | 35 |
| 2 | 25 | | AcD3C13 | | TI | | TIDNYDNSKQDSSKYEVSGTGDGR | | 100% free | 0% | N.I. | N.D. | P38066 | RIB1_YEAST | | control | **GTP cyclohydrolase-2** |  | 1 | 39 | 34 |
| 2 | 11 | | AcD3C13 | | TI | | TISLSNIKKR | | 100% free | 0% | 0% | 0% | A6ZRW1 | NM111_YEAST | | control,hNaa60p | **Pro-apoptotic serine protease NMA111** | P53920 (2-11) | 3 | 57 | 29 |
| 2 | 10 | | AcD3C13 | | TI | | TISNLLKQR | | 100% free | 0% | 0% | 0% | P32316 | ACH1_YEAST | | control,hNaa60p | **Acetyl-CoA hydrolase** |  | 3 | 72 | 33 |
| 2 | 11 | | AcD3C13 | | TK | | TKEEIADKKR | | 100% free | 0% | 0% | 0% | A6ZUA1 | DBP3_YEAST | | control,hNaa60p | **ATP-dependent RNA helicase DBP3** | P20447 (2-11) | 4 | 54 | 34 |
| 2 | 13 | | AcD3C13 | | TK | | TKKKAATNYAER | | 100% free | 0% | N.I. | N.D. | P53280 | CF130_YEAST | | control | **Protein CAF130** |  | 1 | 64 | 35 |
| 2 | 11 | | AcD3C13 | | TK | | TKSEQQADSR | | 100% free | 0% | 0% | 0% | P32582 | CBS_YEAST | | hNaa60p,control | **Cystathionine beta-synthase** |  | 17 | 78 | 36 |
| 2 | 16 | | AcD3C13 | | TL | | TLDDDDYIKQMELQR | | partial Ac- | 8% | 7% | -1% | P40546 | FAF1_YEAST | | control,hNaa60p | **Protein FAF1** |  | 2 | 64 | 36 |
| 2 | 20 | | AcD3C13 | | TL | | TLPESKDFSYLFSDETNAR | | partial Ac- | 33% | N.I. | N.D. | P53090 | ARO8_YEAST | | control | **Aromatic amino acid aminotransferase 1** |  | 2 | 47 | 36 |
| 2 | 18 | | AcD3C13 | | TL | | TLSKYSKPTLNDPNLFR | | N.D. | N.I. | 8% | N.D. | P38067 | UGA2_YEAST | | hNaa60p | **Succinate-semialdehyde dehydrogenase [NADP+]** |  | 2 | 56 | 37 |
| 2 | 19 | | AcD3C13 | | TM | | TMDGKNKEEEQYLDLCKR | | N.D. | N.I. | 0% | N.D. | P06785 | TYSY_YEAST | | hNaa60p | **Thymidylate synthase** |  | 1 | 41 | 36 |
| 2 | 12 | | AcD3C13 | | TM | | TMEKNGGNSSR | | N.D. | N.I. | 0% | N.D. | P47044 | YJF5_YEAST | | hNaa60p | **UPF0717 protein YJL055W** |  | 1 | 43 | 35 |
| 2 | 11 | | AcD3C13 | | TN | | TNKSSLKNNR | | 100% free | 0% | N.I. | N.D. | Q08438 | VHS3_YEAST | | control | **Protein VHS3** |  | 1 | 49 | 36 |
| 2 | 8 | | AcD3C13 | | TP | | TPEAKKR | | 100% free | 0% | N.I. | N.D. | P48415 | SEC16_YEAST | | control | **COPII coat assembly protein SEC16** |  | 1 | 36 | 33 |
| 2 | 16 | | Ace,AcD3C13 | | TQ | | TQFTDIDKLAVSTIR | | partial Ac- | 9% | 9% | 0% | P23254 | TKT1_YEAST | | control,hNaa60p | **Transketolase 1** |  | 11 | 96 | 36 |
| 2 | 20 | | Ace | | TS | | TSASITNTGNETMNLPQMR | | N.D. | N.D. | 90% | N.D. | Q06648 | GIC2_YEAST | | control,hNaa60p | **GTPase-interacting component 2** |  | 5 | 84 | 32 |
| 2 | 11 | | Ace,AcD3C13 | | TS | | TSATDKSIDR | | partial Ac- | 37% | 43% | 6% | A6ZSP9 | LAG1_YEAST | | hNaa60p,control | **Sphingosine N-acyltransferase LAG1** | P38703 (2-11) | 3 | 77 | 37 |
| 2 | 12 | | Ace | | TS | | TSENPDVLLSR | | 100% Ac | 100% | N.I. | N.D. | Q12149 | RRP6_YEAST | | control | **Exosome complex exonuclease RRP6** |  | 1 | 42 | 37 |
| 2 | 20 | | Ace | | TS | | TSIGTGYDLSNSVFSPDGR | | 100% Ac | 100% | 100% | 0% | P21242 | PSA3_YEAST | | control,hNaa60p | **Proteasome component C1** |  | 3 | 39 | 34 |
| 2 | 17 | | Ace | | TS | | TSKVGEYEDVPEDESR | | N.D. | N.I. | 100% | N.D. | A6ZRY1 | OCA1_YEAST | | hNaa60p | **Putative tyrosine-protein phosphatase OCA1** | P50946 (2-17) | 1 | 63 | 33 |
| 2 | 13 | | Ace | | TS | | TSLYAPGAEDIR | | 100% Ac | 100% | 100% | 0% | Q08951 | AP3D_YEAST | | control,hNaa60p | **AP-3 complex subunit delta** |  | 2 | 62 | 36 |
| 2 | 20 | | Ace | | TS | | TSQLNELVEFLHSPQPAVR | | partial Ac- | 82% | N.I. | N.D. | P48362 | HGH1_YEAST | | control | **Protein HGH1** |  | 1 | 77 | 37 |
| 2 | 9 | | Ace,AcD3C13 | | TS | | TSVQNSPR | | N.D. | N.I. | 88% | N.D. | P18852 | GBG_YEAST | | hNaa60p | **Guanine nucleotide-binding protein subunit gamma** |  | 2 | 49 | 36 |
| 2 | 14 | | Ace | | TT | | TTASSSASQLQQR | | 100% Ac | 91% | 92% | 1% | Q03533 | KM8S_YEAST | | hNaa60p,control | **Probable serine/threonine-protein kinase YMR291W** |  | 3 | 96 | 37 |
| 2 | 24 | | AcD3C13 | | TT | | TTDNAKAQLTSSSGGNIIVVSNR | | partial Ac- | 22% | 21% | -1% | Q00764 | TPS1_YEAST | | control,hNaa60p | **Alpha,alpha-trehalose-phosphate synthase [UDP-forming] 56 kDa subunit** |  | 6 | 112 | 37 |
| 2 | 9 | | Ace | | TT | | TTNEEFIR | | 100% Ac | 100% | N.I. | N.D. | P32485 | HOG1_YEAST | | control | **Mitogen-activated protein kinase HOG1** |  | 1 | 53 | 36 |
| 2 | 13 | | Ace | | TT | | TTQESIKPLVDR | | partial Ac- | 79% | 77% | -1% | P53874 | UBP10_YEAST | | control,hNaa60p | **Ubiquitin carboxyl-terminal hydrolase 10** |  | 2 | 66 | 37 |
| 2 | 13 | | Ace,AcD3C13 | | TT | | TTTAQDNSPKKR | | partial Ac- | 65% | 62% | -3% | P31688 | TPS2_YEAST | | hNaa60p,control | **Trehalose-phosphatase** |  | 6 | 70 | 37 |
| 2 | 16 | | Ace | | TT | | TTTIGSPQMLANEER | | partial Ac- | 87% | N.I. | N.D. | P19263 | MED14_YEAST | | control | **Mediator of RNA polymerase II transcription subunit 14** |  | 1 | 87 | 35 |
| 2 | 12 | | Ace,AcD3C13 | | TT | | TTTSTTSVDGR | | partial Ac- | 52% | 48% | -4% | Q07362 | PBP4_YEAST | | control,hNaa60p | **Protein PBP4** |  | 9 | 93 | 35 |
| 2 | 8 | | AcD3C13 | | TV | | TVNDKKR | | N.D. | N.I. | 0% | N.D. | P38866 | FMO1_YEAST | | hNaa60p | **Thiol-specific monooxygenase** |  | 1 | 45 | 35 |
| 2 | 11 | | AcD3C13 | | TV | | TVSNIGGEER | | 100% free | 0% | 0% | 0% | Q05635 | RNH2B_YEAST | | control,hNaa60p | **Ribonuclease H2 subunit B** |  | 2 | 55 | 37 |
| **23. Val-** | | | | | | | | | | | | | | | | | | | |  |  |
| 2 | 29 | | AcD3C13 | | VA | | VAFLELTSDVSQPFVIPSLSPVSQPSSR | | N.D. | N.I. | 0% | N.D. | Q3E752 | YP036_YEAST | | hNaa60p | **Uncharacterized protein YPR036W-A** |  | 1 | 61 | 37 |
| 2 | 10 | | AcD3C13 | | VA | | VAFTVDQMR | | 100% free | 0% | 0% | 0% | P32324 | EF2_YEAST | | hNaa60p,control | **Elongation factor 2** |  | 291 | 68 | 36 |
| 2 | 23 | | AcD3C13 | | VA | | VAISEVKENPGVNSSNSGAVTR | | 100% free | 0% | N.D. | N.D. | Q03940 | RUVB1_YEAST | | control,hNaa60p | **RuvB-like protein 1** |  | 8 | 100 | 38 |
| 2 | 9 | | AcD3C13 | | VA | | VALISKKR | | 100% free | 0% | 0% | 0% | P05750 | RS3_YEAST | | hNaa60p,control | **40S ribosomal protein S3** |  | 184 | 61 | 26 |
| 2 | 24 | | AcD3C13 | | VA | | VAPTALKKATVTPVSGQDGGSSR | | 100% free | 0% | N.I. | N.D. | Q99216 | PNO1_YEAST | | control | **Pre-rRNA-processing protein PNO1** |  | 1 | 64 | 36 |
| 2 | 21 | | AcD3C13 | | VA | | VAQYTVPVGKAANEHETAPR | | 100% free | 0% | 0% | 0% | P30624 | LCF1_YEAST | | hNaa60p,control | **Long-chain-fatty-acid--CoA ligase 1** |  | 3 | 116 | 38 |
| 2 | 7 | | AcD3C13 | | VA | | VASTKR | | partial Ac- | 2% | N.I. | N.D. | Q05022 | RRP5_YEAST | | control | **rRNA biogenesis protein RRP5** |  | 1 | 41 | 34 |
| 2 | 7 | | AcD3C13 | | VA | | VATVKR | | 100% free | 0% | N.I. | N.D. | P35189 | TAF14_YEAST | | control | **Transcription initiation factor TFIID subunit 14** |  | 2 | 43 | 35 |
| 2 | 14 | | AcD3C13 | | VA | | VAYSPIIATYGNR | | 100% free | 0% | 0% | 0% | P38687 | SRP68_YEAST | | hNaa60p,control | **Signal recognition particle subunit SRP68** |  | 2 | 103 | 37 |
| 2 | 13 | | AcD3C13 | | VD | | VDLEQEFALGGR | | 100% free | 0% | N.I. | N.D. | Q12432 | EAF3_YEAST | | control | **Chromatin modification-related protein EAF3** |  | 1 | 64 | 37 |
| 2 | 20 | | AcD3C13 | | VD | | VDLMVPANDDPSNETDYSR | | N.D. | N.I. | 0% | N.D. | P40456 | YI151_YEAST | | hNaa60p | **Uncharacterized protein YIL151C** |  | 1 | 49 | 31 |
| 2 | 17 | | AcD3C13 | | VD | | VDLSTALIHGDDKDNR | | N.D. | N.D. | 0% | N.D. | P38716 | YHR2_YEAST | | hNaa60p,control | **Uncharacterized trans-sulfuration enzyme YHR112C** |  | 2 | 94 | 38 |
| 2 | 18 | | AcD3C13 | | VD | | VDTHKLADDVLQLLDNR | | partial Ac- | 2% | N.I. | N.D. | P43591 | YFH7_YEAST | | control | **Uncharacterized protein YFR007W** |  | 1 | 38 | 37 |
| 2 | 20 | | AcD3C13 | | VD | | VDVEEKSQEVEYVDPTVNR | | 100% free | 0% | N.I. | N.D. | Q06103 | RPN7_YEAST | | control | **26S proteasome regulatory subunit RPN7** |  | 2 | 63 | 37 |
| 2 | 10 | | AcD3C13 | | VE | | VEFSLKKAR | | 100% free | 0% | N.I. | N.D. | P38261 | EXO84_YEAST | | control | **Exocyst complex component EXO84** |  | 1 | 57 | 32 |
| 2 | 21 | | AcD3C13 | | VE | | VELTEIKDDVVQLDEPQFSR | | 100% free | 0% | N.I. | N.D. | P49334 | TOM22_YEAST | | control | **Mitochondrial import receptor subunit TOM22** |  | 3 | 104 | 37 |
| 2 | 7 | | AcD3C13 | | VF | | VFDLKR | | 100% free | 0% | N.I. | N.D. | P07172 | HIS8_YEAST | | control | **Histidinol-phosphate aminotransferase** | P35728 (27-32) | 1 | 43 | 33 |
| 2 | 13 | | AcD3C13 | | VG | | VGSKDIDLFNLR | | partial Ac- | 4% | N.I. | N.D. | P40483 | YIK8_YEAST | | control | **Putative zinc metalloproteinase YIL108W** |  | 1 | 66 | 37 |
| 2 | 13 | | AcD3C13 | | VI | | VIKEDCINNLAR | | N.D. | N.I. | 0% | N.D. | Q06651 | PIB1_YEAST | | hNaa60p | **E3 ubiquitin-protein ligase PIB1** |  | 1 | 63 | 37 |
| 2 | 21 | | AcD3C13 | | VK | | VKKVNNPLKIDYQNGIIENR | | N.D. | N.I. | 0% | N.D. | Q9URQ3 | TAD3_YEAST | | hNaa60p | **tRNA-specific adenosine deaminase subunit TAD3** |  | 1 | 59 | 34 |
| 2 | 9 | | AcD3C13 | | VK | | VKLAEFSR | | N.D. | N.D. | 0% | N.D. | P38968 | SEC31_YEAST | | control,hNaa60p | **Protein transport protein SEC31** |  | 3 | 66 | 36 |
| 2 | 7 | | AcD3C13 | | VK | | VKSYQR | | 100% free | 0% | N.I. | N.D. | Q12220 | UTP12_YEAST | | control | **U3 small nucleolar RNA-associated protein 12** |  | 1 | 41 | 35 |
| 2 | 16 | | AcD3C13 | | VK | | VKVYIHDNKVDSDYR | | 100% free | 0% | 0% | 0% | Q03677 | MTND_YEAST | | control,hNaa60p | **1,2-dihydroxy-3-keto-5-methylthiopentene dioxygenase** |  | 2 | 69 | 38 |
| 2 | 9 | | AcD3C13 | | VL | | VLADLGKR | | 100% free | 0% | 0% | 0% | P20424 | SRP54_YEAST | | hNaa60p,control | **Signal recognition particle subunit SRP54** |  | 3 | 76 | 33 |
| 2 | 8 | | AcD3C13 | | VL | | VLAMESR | | 100% free | 0% | 0% | 0% | Q01159 | MCE1_YEAST | | control,hNaa60p | **mRNA-capping enzyme subunit alpha** |  | 2 | 53 | 37 |
| 2 | 17 | | AcD3C13 | | VL | | VLEATVLVIDNSEYSR | | N.D. | N.I. | 0% | N.D. | P38886 | RPN10_YEAST | | hNaa60p | **26S proteasome regulatory subunit RPN10** |  | 4 | 110 | 37 |
| 2 | 12 | | AcD3C13 | | VL | | VLNPSKYQDTR | | N.D. | N.I. | 6% | N.D. | Q3E7B2 | YJ062_YEAST | | hNaa60p | **Mitochondrial membrane protein YJL062W-A** |  | 1 | 45 | 37 |
| 2 | 17 | | AcD3C13 | | VL | | VLVQDLLHPTAASEAR | | N.D. | N.D. | 0% | N.D. | P35997 | RS27A_YEAST | | control,hNaa60p | **40S ribosomal protein S27-A** | P38711 (2-17) | 185 | 119 | 36 |
| 2 | 9 | | AcD3C13 | | VM | | VMQEEKKR | | 100% free | 0% | 0% | 0% | A6ZZF6 | KKQ8_YEAST | | hNaa60p,control | **Probable serine/threonine-protein kinase KKQ8** | P36004 (2-9) | 2 | 56 | 37 |
| 2 | 14 | | AcD3C13 | | VN | | VNFDLGQVGEVFR | | N.D. | N.I. | 0% | N.D. | P42838 | LEM3_YEAST | | hNaa60p | **Alkylphosphocholine resistance protein LEM3** |  | 1 | 42 | 38 |
| 2 | 16 | | AcD3C13 | | VN | | VNISDFFGKNKKSVR | | 100% free | 0% | N.I. | N.D. | P38630 | RFC1_YEAST | | control | **Replication factor C subunit 1** |  | 2 | 56 | 35 |
| 2 | 8 | | AcD3C13 | | VN | | VNVPKTR | | 100% free | 0% | 0% | 0% | P02405 | RL44_YEAST | | control,hNaa60p | **60S ribosomal protein L42** |  | 7 | 39 | 31 |
| 2 | 19 | | AcD3C13 | | VP | | VPAESNAVQAKLAKTLQR | | 100% free | 0% | 0% | 0% | Q12125 | YO164_YEAST | | control,hNaa60p | **UPF0363 protein YOR164C** |  | 2 | 73 | 34 |
| 2 | 13 | | AcD3C13 | | VQ | | VQAIKLNDLKNR | | 100% free | 0% | N.I. | N.D. | Q06338 | BCP1_YEAST | | control | **Protein BCP1** |  | 1 | 72 | 32 |
| 2 | 44 | | AcD3C13 | | VQ | | VQAVAVLKGDAGVSGVVKFEQASESEPTTVSYEIAGNSPNAER | | 100% free | 0% | 0% | 0% | P00445 | SODC_YEAST | | hNaa60p,control | **Superoxide dismutase [Cu-Zn]** |  | 2 | 71 | 37 |
| 2 | 11 | | AcD3C13 | | VQ | | VQSAVLGFPR | | 100% free | 0% | 0% | 0% | P05694 | METE_YEAST | | hNaa60p,control | **5-methyltetrahydropteroyltriglutamate--homocysteine methyltransferase** |  | 79 | 83 | 35 |
| 2 | 10 | | AcD3C13 | | VS | | VSPTKMIIR | | N.D. | N.I. | 0% | N.D. | P32325 | DBF4_YEAST | | hNaa60p | **Protein DBF4** |  | 1 | 40 | 35 |
| 2 | 10 | | AcD3C13 | | VS | | VSQFAIEVR | | 100% free | 0% | N.I. | N.D. | P43569 | YFC8_YEAST | | control | **Uncharacterized ABC transporter ATP-binding protein YFL028C** |  | 1 | 39 | 37 |
| 2 | 15 | | AcD3C13 | | VT | | VTSNVVLVSGEGER | | 100% free | 0% | 0% | 0% | P52286 | SKP1_YEAST | | control,hNaa60p | **Suppressor of kinetochore protein 1** |  | 28 | 90 | 37 |
| 2 | 11 | | AcD3C13 | | VV | | VVLDKKLLER | | 100% free | 0% | 0% | 0% | P38276 | YBY7_YEAST | | control,hNaa60p | **UPF0303 protein YBR137W** |  | 7 | 74 | 27 |
| 2 | 10 | | AcD3C13 | | VV | | VVNTIYIAR | | 100% free | 0% | N.I. | N.D. | Q12415 | TFC7_YEAST | | control | **Transcription factor tau 55 kDa subunit** |  | 1 | 53 | 36 |
| 2 | 8 | | AcD3C13 | | VV | | VVQKKLR | | N.D. | N.I. | 0% | N.D. | Q12246 | LCB4_YEAST | | hNaa60p | **Sphingoid long chain base kinase 4** |  | 1 | 34 | 23 |
| 2 | 10 | | AcD3C13 | | VY | | VYTPSKGPR | | 100% free | 0% | N.D. | N.D. | P07264 | LEUC_YEAST | | hNaa60p,control | **3-isopropylmalate dehydratase** |  | 3 | 45 | 36 |
| **24. Ile** | | | | | | | | | | | | | | | | | | | |  |  |
| 1 | 21 | | AcD3C13 | | II | | IIEPSLKALASKYNCDKSVCR | | 100% free | 0% | 0% | 0% | P14796 | RL40_YEAST | | control,hNaa60p | **60S ribosomal protein L40** |  | 21 | 94 | 37 |
