# Supplementary material for: NatF Contributes to an Evolutionary Shift in Protein N-Terminal Acetylation and Is Important for Normal Chromosome Segregation
Source: PLoS Genet. 2011 Jul 7;7(7):e1002169. doi: 10.1371/journal.pgen.1002169 (PMC3131286; doi:10.1371/journal.pgen.1002169)
Supplement: Table S2 — List of 1,497 human N-terminal peptides (start position 1 or 2) identified in the hNaa60p overexpression or knockdown experiments in HeLa cells. (DOC) [file pgen.1002169.s004.doc]

| **Table S2. List of 1497 Human N-termini identified.** 1497 human N-terminal peptides (start position 1 or 2) identified in the hNaa60 overexpression or knockdown experiments are listed. Start and end positions, N-term modification status confirmed by MS/MS, corresponding peptide sequence identified, global N-term modification status in control samples for either hNaa60 overexpression or knockdown dataset (N-termini found to be less than 2% Ac and more than 98%, were considered as 100% free and 100% Ac respectively when considering their global Ac-status). N.D (not determined) indicates N-termini where acetylation status could not be calculated. %Ac of the N-terminus in the control, UniProt database primary accession number and UniProt name are indicated. The peptides are listed alphabetically according to their identified peptide sequence.  _____________________________________________________________________________________________________________________________________________________________________________________ | | | | | | | | | | | | |
| --- | --- | --- | --- | --- | --- | --- | --- | --- | --- | --- | --- | --- |
|
|  |  | |  |  | |  |  |  |  |  |  |  |
| **start** | **end** | | **N-term modification** | **AA**  **1-2** | | **sequence** | **N-term modification status** | **% Ac control** | **accession** | **Uniprot** | **Description** | **isoforms** |
| **1. Ala-** |  | |  |  | |  |  |  |  |  |  |  |
| 2 | 17 | | Ace | AA | | AAAAAAAAAAGAAGGR | 100% Ac- | 100.00 | Q86U42 | PABP2_HUMAN | **Polyadenylate-binding protein 2** |  |
| 2 | 15 | | Ace | AA | | AAAAAAGAGPEMVR | 100% Ac- | 100.00 | P28482 | MK01_HUMAN | **Mitogen-activated protein kinase 1** |  |
| 2 | 11 | | Ace | AA | | AAAAAAGEAR | 100% Ac- | 100.00 | P09417 | DHPR_HUMAN | **Dihydropteridine reductase** |  |
| 2 | 29 | | Ace | AA | | AAAAAAGPSPGSGPGDSPEGPEGEAPER | 100% Ac- | 100.00 | Q9UID3 | FFR_HUMAN | **Protein fat-free homolog** |  |
| 2 | 13 | | Ace | AA | | AAAAAAGSGTPR | 100% Ac- | 100.00 | Q9NR33 | DPOE4_HUMAN | **DNA polymerase epsilon subunit 4** |  |
| 2 | 16 | | Ace | AA | | AAAAAATAAAAASIR | Partial-Ac- | 62.03 | Q8WVM8 | SCFD1_HUMAN | **Sec1 family domain-containing protein 1** |  |
| 2 | 36 | | Ace | AA | | AAAAAAVGPGAGGAGSAVPGGAGPCATVSVFPGAR | N.D | N.D | Q86X55 | CARM1_HUMAN | **Histone-arginine methyltransferase CARM1** |  |
| 2 | 13 | | Ace | AA | | AAAAAEEGMEPR | 100% Ac- | 100.00 | P51788 | CLCN2_HUMAN | **Chloride channel protein 2** |  |
| 2 | 26 | | Ace | AA | | AAAAAEQQQFYLLLGNLLSPDNVVR | 100% Ac- | 100.00 | O00410 | IPO5_HUMAN | **Importin-5** |  |
| 2 | 13 | | Ace | AA | | AAAAAETPEVLR | 100% Ac- | 100.00 | Q9NXW9 | ALKB4_HUMAN | **Alkylated DA repair protein alkB homolog 4** |  |
| 2 | 25 | | Ace | AA | | AAAAAGLGGGGAGPGPEAGDFLAR | 100% Ac- | 100.00 | P23610 | F8I2_HUMAN | **Factor VIII intron 22 protein** |  |
| 2 | 13 | | Ace | AA | | AAAAAGTATSQR | 100% Ac- | 100.00 | Q9Y2Z0 | SUGT1_HUMAN | **Suppressor of G2 allele of SKP1 homolog** |  |
| 2 | 14 | | Ace | AA | | AAAAAMAEQESAR | 100% Ac- | 100.00 | Q7L5D6 | CG020_HUMAN | **UPF0363 protein C7orf20** |  |
| 2 | 15 | | Ace | AA | | AAAAAQGGGGGEPR | 100% Ac- | 100.00 | P27361 | MK03_HUMAN | **Mitogen-activated protein kinase 3** |  |
| 2 | 22 | | Ace | AA | | AAAAASAPQQLSDEELFSQLR | 100% Ac- | 100.00 | Q9Y2U8 | MAN1_HUMAN | **Inner nuclear membrane protein Man1** |  |
| 2 | 18 | | Ace | AA | | AAAAASASQDELNQLER | 100% Ac- | 100.00 | Q5VYK3 | ECM29_HUMAN | **Proteasome-associated protein ECM29 homolog** |  |
| 2 | 16 | | Ace | AA | | AAAADSFSGGPAGVR | 100% Ac- | 100.00 | Q96E14 | RMI2_HUMAN | **RecQ-mediated genome instability protein 2** |  |
| 2 | 26 | | Ace | AA | | AAAAECDVVMAATEPELLDDQEAKR | 100% Ac- | 100.00 | Q99615 | DNJC7_HUMAN | **DnaJ homolog subfamily C member 7** |  |
| 2 | 12 | | Ace | AA | | AAAAEGVLATR | 100% Ac- | 100.00 | Q6QNY1 | BL1S2_HUMAN | **Biogenesis of lysosome-related organelles complex 1 subunit 2** |  |
| 2 | 28 | | Ace | AA | | AAAAELSLLEKSLGLSKGNKYSAQGER | 100% Ac- | 100.00 | O43324 | MCA3_HUMAN | **Eukaryotic translation elongation factor 1 epsilon-1** |  |
| 2 | 13 | | Ace | AA | | AAAAGDADDEPR | 100% Ac- | 100.00 | Q6NXT1 | ANR54_HUMAN | **Ankyrin repeat domain-containing protein 54** |  |
| 2 | 17 | | Ace | AA | | AAAAGGGSCPGPGSAR | 100% Ac- | 100.00 | Q9UMN6 | MLL4_HUMAN | **Histone-lysine -methyltransferase MLL4** |  |
| 2 | 11 | | Ace | AA | | AAAAGGPCVR | N.D | N.D | Q5SRE5 | NU188_HUMAN | **Nucleoporin UP188 homolog** |  |
| 2 | 31 | | Ace | AA | | AAAAGAAAAAAAEGEAPAEMGALLLEKETR | 100% Ac- | 100.00 | Q96KQ7 | EHMT2_HUMAN | **Histone-lysine -methyltransferase, H3 lysine-9 specific 3** |  |
| 2 | 18 | | Ace | AA | | AAAAMAAAAGGGAGAAR | 100% Ac- | 100.00 | Q9NX46 | ARHL2_HUMAN | **Poly(ADP-ribose) glycohydrolase ARH3** |  |
| 2 | 14 | | Ace | AA | | AAAAPVAADDDER | 100% Ac- | 100.00 | Q9H5N1 | RABE2_HUMAN | **Rab GTPase-binding effector protein 2** |  |
| 2 | 32 | | Ace | AA | | AAAAPAAAAASSEAPAASATAEPEAGDQDSR | 100% Ac- | 100.00 | Q15283 | RASA2_HUMAN | **Ras GTPase-activating protein 2** |  |
| 2 | 24 | | Ace | AA | | AAAAQLSLTQLSSGNPVYEKYYR | 100% Ac- | 100.00 | P42566 | EPS15_HUMAN | **Epidermal growth factor receptor substrate 15** |  |
| 2 | 12 | | Ace | AA | | AAAATMAAAAR | 100% Ac- | 100.00 | Q8N1B4 | VPS52_HUMAN | **Vacuolar protein sorting-associated protein 52 homolog** |  |
| 2 | 14 | | Ace | AA | | AAAATAAEGVPSR | 100% Ac- | 100.00 | Q8TBC3 | SHKB1_HUMAN | **SH3KBP1-binding protein 1** |  |
| 2 | 10 | | Ace | AA | | AAAAVQGGR | 100% Ac- | 100.00 | Q15005 | SPCS2_HUMAN | **Signal peptidase complex subunit 2** |  |
| 2 | 18 | | Ace | AA | | AAAAVSESWPELELAER | 100% Ac- | 100.00 | Q8N1G4 | LRC47_HUMAN | **Leucine-rich repeat-containing protein 47** |  |
| 2 | 11 | | Ace | AA | | AAAAVSSAKR | 100% Ac- | 100.00 | P49914 | MTHFS_HUMAN | **5-formyltetrahydrofolate cyclo-ligase** |  |
| 2 | 11 | | Ace | AA | | AAAAVVEFQR | 100% Ac- | 100.00 | O00231 | PSD11_HUMAN | **26S proteasome non-ATPase regulatory subunit 11** |  |
| 2 | 26 | | Ace | AA | | AAAAAAAGDSDSWDADAFSVEDPVR | 100% Ac- | 100.00 | O75822 | EIF3J_HUMAN | **Eukaryotic translation initiation factor 3 subunit J** |  |
| 2 | 40 | | Ace | AA | | AAAAAAAGAAGSAAPAAAAGAPGSGGAPSGSQGVLIGDR | 100% Ac- | 100.00 | Q96S94 | CCNL2_HUMAN | **Cyclin-L2** |  |
| 2 | 18 | | Ace | AA | | AAAAAAALESWQAAAPR | 100% Ac- | 100.00 | Q9UH36 | SRR1L_HUMAN | **SRR1-like protein** |  |
| 2 | 25 | | Ace | AA | | AAADGALPEAAALEQPAELPASVR | 100% Ac- | 100.00 | P23025 | XPA_HUMAN | **DNA repair protein complementing XP-A cells** |  |
| 2 | 21 | | Ace | AA | | AAADGDDSLYPIAVLIDELR | 100% Ac- | 100.00 | P30153 | 2AAA_HUMAN | **Serine/threonine-protein phosphatase 2A 65 kDa regulatory subunit A alpha isoform** |  |
| 2 | 16 | | Ace | AA | | AAAEEEDGGPEGPNR | 100% Ac- | 100.00 | Q99942 | RNF5_HUMAN | **E3 ubiquitin-protein ligase RF5** |  |
| 2 | 22 | | Ace | AA | | AAAEEEPKPKKLKVEAPQALR | 100% Ac- | 100.00 | P55263 | ADK_HUMAN | **Adenosine kinase** |  |
| 2 | 16 | | Ace | AA | | AAAEEGCSVGAEADR | N.D | N.D | P40855 | PEX19_HUMAN | **Peroxisomal biogenesis factor 19** |  |
| 2 | 9 | | Ace | AA | | AAAEPSPR | 100% Ac- | 100.00 | Q53H96 | P5CR3_HUMAN | **Pyrroline-5-carboxylate reductase 3** |  |
| 2 | 10 | | Ace | AA | | AAAETQSLR | 100% Ac- | 100.00 | Q13523 | PRP4B_HUMAN | **Serine/threonine-protein kinase PRP4 homolog** |  |
| 2 | 9 | | Ace | AA | | AAAGALER | 100% Ac- | 100.00 | Q12769 | NU160_HUMAN | **Nuclear pore complex protein up160** |  |
| 2 | 16 | | Ace | AA | | AAAGGGGGGAAAAGR | 100% Ac- | 100.00 | Q9UL25 | RAB21_HUMAN | **Ras-related protein Rab-21** |  |
| 2 | 16 | | Ace | AA | | AAAITDMADLEELSR | N.D | N.D | Q6ZN18 | AEBP2_HUMAN | **Zinc finger protein AEBP2** |  |
| 2 | 28 | | Ace | AA | | AAALGASGGAGAGDDDFDQFDKPGAER | 100% Ac- | 100.00 | Q96EV2 | RBM33_HUMAN | **RA-binding protein 33** |  |
| 2 | 21 | | Ace | AA | | AAAMDVDTPSGTNSGAGKKR | 100% Ac- | 100.00 | P62877 | RBX1_HUMAN | **RING-box protein 1** |  |
| 2 | 9 | | Ace | AA | | AAAMVPGR | 100% Ac- | 100.00 | O43598 | RCL_HUMAN | **c-Myc-responsive protein Rcl** |  |
| 2 | 11 | | Ace | AA | | AAANKGNKPR | 100% Ac- | 100.00 | Q9GZN8 | CT027_HUMAN | **UPF0687 protein C20orf27** |  |
| 2 | 16 | | Ace | AA | | AAAPPSYCFVAFPPR | 100% Ac- | 100.00 | Q12765 | SCRN1_HUMAN | **Secernin-1** |  |
| 2 | 10 | | Ace | AA | | AAAPQAPGR | 100% Ac- | 100.00 | P78345 | RPP38_HUMAN | **Ribonuclease P protein subunit p38** |  |
| 2 | 14 | | Ace | AA | | AAAPVAAGSGAGR | N.D | N.D | Q8TEL6 | TP4AP_HUMAN | **Trpc4-associated protein** |  |
| 2 | 11 | | Ace | AA | | AAAQEADGAR | 100% Ac- | 100.00 | O96005 | CLPT1_HUMAN | **Cleft lip and palate transmembrane protein 1** |  |
| 2 | 11 | | Ace | AA | | AAASGSVLQR | 100% Ac- | 100.00 | Q5VWZ2 | LYPL1_HUMAN | **Lysophospholipase-like protein 1** |  |
| 2 | 11 | | Ace | AA | | AAASGYTDLR | 100% Ac- | 100.00 | Q6P6C2 | ALKB5_HUMAN | **Alkylated DA repair protein alkB homolog 5** |  |
| 2 | 37 | | Ace | AA | | AAATADPGAGNPQPGDSSGGGAGGGLPSPGEQELSR | 100% Ac- | 100.00 | Q6VN20 | RBP10_HUMAN | **Ran-binding protein 10** |  |
| 2 | 26 | | Ace | AA | | AAATGAVAASAASGQAEGKKITDLR | N.D | N.D | Q9NWH9 | SLTM_HUMAN | **SAFB-like transcription modulator** |  |
| 2 | 12 | | Ace | AA | | AAAVADEAVAR | 100% Ac- | 100.00 | Q9NVX2 | NLE1_HUMAN | **Notchless protein homolog 1** |  |
| 2 | 15 | | Ace | AA | | AAAVAMETDDAGNR | N.D | N.D | Q9Y3C7 | MED31_HUMAN | **Mediator of RNA polymerase II transcription subunit 31** |  |
| 2 | 12 | | Ace | AA | | AAAVAVAAASR | 100% Ac- | 100.00 | Q7Z5L9 | I2BP2_HUMAN | **Interferon regulatory factor 2-binding protein 2** |  |
| 2 | 9 | | Ace | AA | | AAAVLMDR | N.D | N.D | Q9HCX3 | ZN304_HUMAN | **Zinc finger protein 304** |  |
| 2 | 32 | | Ace | AA | | AAAVLSGPSAGSAAGVPGGTGGLSAVSSGPR | 100% Ac- | 100.00 | Q8IZL8 | PELP1_HUMAN | **Proline-, glutamic acid- and leucine-rich protein 1** |  |
| 2 | 9 | | Ace | AA | | AAAVLTDR | 100% Ac- | 100.00 | O43296 | ZN264_HUMAN | **Zinc finger protein 264** |  |
| 2 | 8 | | Ace | AA | | AAAVPQR | 100% Ac- | 100.00 | Q00688 | FKBP3_HUMAN | **FK506-binding protein 3** |  |
| 2 | 10 | | Ace | AA | | AAAVSSVVR | 100% Ac- | 100.00 | A6NDU8 | CE051_HUMAN | **UPF0600 protein C5orf51** |  |
| 2 | 11 | | Ace | AA | | AADEVAGGAR | 100% Ac- | 100.00 | Q6STE5 | SMRD3_HUMAN | **SWI/SF-related matrix-associated actin-dependent regulator of chromatin subfamily D member 3** |  |
| 2 | 18 | | Ace | AA | | AADISESSGADCKGDPR | 100% Ac- | 100.00 | O43583 | DENR_HUMAN | **Density-regulated protein** |  |
| 2 | 12 | | AcD3 | AA | | AADKGPAAGPR | Partial-Ac- | 28.86 | Q8TDD1 | DDX54_HUMAN | **ATP-dependent RA helicase DDX54** |  |
| 2 | 13 | | Ace | AA | | AADTQVSETLKR | Partial-Ac- | 80.93 | Q92616 | GCN1L_HUMAN | **Translational activator GC1** |  |
| 2 | 11 | | Ace | AA | | AADVFMCSPR | N.D | N.D | Q9HAP2 | MLXIP_HUMAN | **MLX-interacting protein** |  |
| 2 | 14 | | Ace | AA | | AAEALAAEAVASR | 100% Ac- | 100.00 | A2RTX5 | SYTC2_HUMAN | **Probable threonyl-tRA synthetase 2, cytoplasmic** |  |
| 2 | 11 | | Ace | AA | | AAEDELQLPR | 100% Ac- | 100.00 | P78318 | IGBP1_HUMAN | **Immunoglobulin-binding protein 1** |  |
| 2 | 16 | | Ace | AA | | AAEEEEVDSADTGER | 100% Ac- | 100.00 | Q8WTS1 | ABHD5_HUMAN | **Abhydrolase domain-containing protein 5** |  |
| 2 | 36 | | Ace | AA | | AAEEPQQQKQEPLGSDSEGVNCLAYDEAIMAQQDR | 100% Ac- | 100.00 | Q96FW1 | OTUB1_HUMAN | **Ubiquitin thioesterase OTUB1** |  |
| 2 | 9 | | Ace | AA | | AAEIDFLR | 100% Ac- | 100.00 | Q96NL6 | SCLT1_HUMAN | **Sodium channel and clathrin linker 1** |  |
| 2 | 31 | | AcD3 | AA | | AAEKQVPGGGGGGGSGGGGGSGGGGSGGGR | Partial-Ac- | 46.12 | Q9UHB9 | SRP68_HUMAN | **Signal recognition particle 68 kDa protein** |  |
| 2 | 16 | | Ace | AA | | AAELVEAKNMVMSFR | N.D | N.D | Q8N2W9 | PIAS4_HUMAN | **E3 SUMO-protein ligase PIAS4** |  |
| 2 | 16 | | Ace | AA | | AAEPNKTEIQTLFKR | 100% Ac- | 100.00 | Q8N6H7 | ARFG2_HUMAN | **ADP-ribosylation factor GTPase-activating protein 2** |  |
| 2 | 16 | | Ace | AA | | AAESALQVVEKLQAR | 100% Ac- | 100.00 | Q14241 | ELOA1_HUMAN | **Transcription elongation factor B polypeptide 3** |  |
| 2 | 12 | | Ace | AA | | AAESGSDFQQR | 100% Ac- | 100.00 | Q96BP3 | PPWD1_HUMAN | **Peptidylprolyl isomerase domain and WD repeat-containing protein 1** |  |
| 2 | 16 | | Ace | AA | | AAETQTLNFGPEWLR | 100% Ac- | 100.00 | Q6Y7W6 | PERQ2_HUMAN | **PERQ amino acid-rich with GYF domain-containing protein 2** |  |
| 2 | 19 | | Ace | AA | | AAEAADLGLGAAVPVELR | 100% Ac- | 100.00 | Q9Y5Q8 | TF3C5_HUMAN | **General transcription factor 3C polypeptide 5** |  |
| 2 | 11 | | Ace | AA | | AAEAAGGKYR | 100% Ac- | 100.00 | Q96A65 | EXOC4_HUMAN | **Exocyst complex component 4** |  |
| 2 | 10 | | Ace | AA | | AAGCSEAPR | 100% Ac- | 100.00 | Q3B726 | RPA43_HUMAN | **DNA-directed RA polymerase I subunit RPA43** |  |
| 2 | 15 | | Ace | AA | | AAGFKTVEPLEYYR | 100% Ac- | 100.00 | Q96B26 | EXOS8_HUMAN | **Exosome complex exonuclease RRP43** |  |
| 2 | 14 | | Ace | AA | | AAGGAVAAAPECR | 100% Ac- | 100.00 | Q9UL63 | MKLN1_HUMAN | **Muskelin** |  |
| 2 | 29 | | Ace | AA | | AAGGGGGSSKASSSSASSAGALESSLDR | Partial-Ac- | 65.69 | Q5VT52 | RPRD2_HUMAN | **Regulation of nuclear pre-mRA domain-containing protein 2** |  |
| 2 | 12 | | Ace | AA | | AAGGSGVGGKR | Partial-Ac- | 72.76 | Q9NZM5 | GSCR2_HUMAN | **Glioma tumor suppressor candidate region gene 2 protein** |  |
| 2 | 11 | | Ace | AA | | AAGKFASLPR | 100% Ac- | 100.00 | O75815 | BCAR3_HUMAN | **Breast cancer anti-estrogen resistance protein 3** |  |
| 2 | 21 | | Ace | AA | | AAGKSGGSAGEITFLEALAR | 100% Ac- | 100.00 | Q9GZR1 | SENP6_HUMAN | **Sentrin-specific protease 6** |  |
| 2 | 9 | | Ace | AA | | AAGPISER | 100% Ac- | 100.00 | Q15427 | SF3B4_HUMAN | **Splicing factor 3B subunit 4** |  |
| 2 | 14 | | Ace | AA | | AAGTLYTYPENWR | 100% Ac- | 100.00 | P26641 | EF1G_HUMAN | **Elongation factor 1-gamma** |  |
| 2 | 17 | | Ace | AA | | AAGTAAALAFLSQESR | 100% Ac- | 100.00 | O75607 | NPM3_HUMAN | **Nucleoplasmin-3** |  |
| 2 | 14 | | Ace | AA | | AAGVDCGDGVGAR | 100% Ac- | 100.00 | Q5TBB1 | RNH2B_HUMAN | **Ribonuclease H2 subunit B** |  |
| 2 | 27 | | Ace | AA | | AAGAAEAAVAAVEEVGSAGQFEELLR | 100% Ac- | 100.00 | O76003 | GLRX3_HUMAN | **Glutaredoxin-3** |  |
| 2 | 14 | | Ace | AA | | AAIYGGVEGGGTR | 100% Ac- | 100.00 | Q9UJ70 | NAGK_HUMAN | **N-acetyl-D-glucosamine kinase** |  |
| 2 | 21 | | Ace | AA | | AALDSLSLFTSLGLSEQKAR | 100% Ac- | 100.00 | P47897 | SYQ_HUMAN | **Glutaminyl-tRA synthetase** |  |
| 2 | 9 | | Ace | AA | | AALGEPVR | 100% Ac- | 100.00 | Q9NUP9 | LIN7C_HUMAN | **Lin-7 homolog C** |  |
| 2 | 10 | | Ace | AA | | AALGGDGLR | N.D | N.D | Q9Y256 | FACE2_HUMAN | **CAAX prenyl protease 2** |  |
| 2 | 17 | | Ace | AA | | AALGPSSQNVTEYVVR | N.D | N.D | P35269 | T2FA_HUMAN | **General transcription factor IIF subunit 1** |  |
| 2 | 29 | | Ace | AA | | AALGVAEAVAAPHPAEGAETAEAVELSR | 100% Ac- | 100.00 | Q86Y56 | HEAT2_HUMAN | **HEAT repeat-containing protein 2** |  |
| 2 | 8 | | Ace | AA | | AALKEDR | 100% Ac- | 100.00 | P55199 | ELL_HUMAN | **RNA polymerase II elongation factor ELL** |  |
| 2 | 41 | | Ace | AA | | AALMTPGTGAPPAPGDFSGEGSQGLPDPSPEPKQLPELIR | 100% Ac- | 100.00 | P19971 | TYPH_HUMAN | **Thymidine phosphorylase** |  |
| 2 | 17 | | Ace | AA | | AALTTLFKYIDENQDR | 100% Ac- | 100.00 | Q96KP4 | CNDP2_HUMAN | **Cytosolic non-specific dipeptidase** |  |
| 2 | 13 | | Ace | AA | | AALVLEDGSVLR | 100% Ac- | 100.00 | P27708 | PYR1_HUMAN | **CAD protein** |  |
| 2 | 13 | | Ace | AA | | AAMAVGGAGGSR | N.D | N.D | O14744 | ANM5_HUMAN | **Protein arginine -methyltransferase 5** |  |
| 2 | 25 | | Ace | AA | | AANATTNPSQLLPLELVDKCIGSR | 100% Ac- | 100.00 | Q9Y4Y9 | LSM5_HUMAN | **U6 snRA-associated Sm-like protein LSm5** |  |
| 2 | 15 | | Ace | AA | | AANSSGQGFQNKNR | 100% Ac- | 100.00 | Q9NRY2 | CI080_HUMAN | **Uncharacterized protein C9orf80** |  |
| 2 | 9 | | Ace | AA | | AANVFPFR | 100% Ac- | 100.00 | Q9H9F9 | ARP5_HUMAN | **Actin-related protein 5** |  |
| 2 | 9 | | Ace | AA | | AANVGDQR | 100% Ac- | 100.00 | Q96PV6 | LENG8_HUMAN | **Leukocyte receptor cluster member 8** |  |
| 2 | 20 | | Ace | AA | | AAPAGGGGSAVSVLAPNGR | 100% Ac- | 100.00 | Q9BZE9 | ASPC1_HUMAN | **Tether containing UBX domain for GLUT4** |  |
| 2 | 38 | | Ace | AA | | AAPAPGAGAASGGAGCSGGGAGAGAGSGSGAAGAGGR | 100% Ac- | 100.00 | P0C2W1 | FBSP1_HUMAN | **F-box/SPRY domain-containing protein 1** |  |
| 2 | 16 | | Ace | AA | | AAPAQQTTQPGGGKR | 100% Ac- | 100.00 | Q9NXG2 | THUM1_HUMAN | **THUMP domain-containing protein 1** |  |
| 2 | 14 | | Ace | AA | | AAPEGSGLGEDAR | 100% Ac- | 100.00 | Q96G03 | PGM2_HUMAN | **Phosphoglucomutase-2** |  |
| 2 | 21 | | Ace | AA | | AAPGPALCLFDVDGTLTAPR | 100% Ac- | 100.00 | O15305 | PMM2_HUMAN | **Phosphomannomutase 2** |  |
| 2 | 33 | | Ace | AA | | AAPLIPLSQQIPTGNSLYESYYKQVDPAYTGR | 100% Ac- | 100.00 | Q9UBC2 | EP15R_HUMAN | **Epidermal growth factor receptor substrate 15-like 1** |  |
| 2 | 18 | | Ace | AA | | AAPPGEYFSVGSQVSCR | 100% Ac- | 100.00 | Q3MHD2 | LSM12_HUMAN | **Protein LSM12 homolog** |  |
| 2 | 11 | | Ace | AA | | AAPSDGFKPR | 100% Ac- | 100.00 | Q92979 | NEP1_HUMAN | **Probable ribosome biogenesis protein EP1** |  |
| 2 | 15 | | Ace | AA | | AAPVVAPPGVVVSR | 100% Ac- | 100.00 | Q13144 | EI2BE_HUMAN | **Translation initiation factor eIF-2B subunit epsilon** |  |
| 2 | 22 | | Ace | AA | | AAQGVGPGPGSAAPPGLEAAR | 100% Ac- | 100.00 | Q6NZ67 | F128B_HUMAN | **Protein FAM128B** | Q6P582 (2-22) |
| 2 | 18 | | Ace | AA | | AAQIPIVATTSTPGIVR | 100% Ac- | 100.00 | Q6PI98 | IN80C_HUMAN | **IO80 complex subunit C** |  |
| 2 | 32 | | Ace | AA | | AAQKDQQKDAEAEGLSGTTLLPKLIPSGAGR | 100% Ac- | 100.00 | Q9UI14 | PRAF1_HUMAN | **Prenylated Rab acceptor protein 1** |  |
| 2 | 22 | | Ace | AA | | AAQSAPKVVLKSTTKMSLNER | 100% Ac- | 100.00 | Q9Y3Y2 | CA077_HUMAN | **Uncharacterized protein C1orf77** |  |
| 2 | 32 | | Ace | AA | | AAQVAPAAASSLGNPPPPPPSELKKAEQQQR | 100% Ac- | 100.00 | O14497 | ARI1A_HUMAN | **AT-rich interactive domain-containing protein 1A** |  |
| 2 | 9 | | Ace | AA | | AAQVGAVR | 100% Ac- | 100.00 | Q5VWQ0 | RSBN1_HUMAN | **Round spermatid basic protein 1** |  |
| 2 | 15 | | Ace | AA | | AASALYACTKCTQR | 100% Ac- | 100.00 | Q5HYJ3 | FA76B_HUMAN | **Protein FAM76B** |  |
| 2 | 12 | | Ace | AA | | AASELYTKFAR | 100% Ac- | 100.00 | Q9Y4I1 | MYO5A_HUMAN | **Myosin-Va** |  |
| 2 | 8 | | Ace | AA | | AASETVR | 100% Ac- | 100.00 | P38432 | P80C_HUMAN | **Coilin** |  |
| 2 | 33 | | Ace | AA | | AASGESGTSGGGGSTEEAFMTFYSEVKQIEKR | 100% Ac- | 100.00 | O75937 | DNJC8_HUMAN | **DnaJ homolog subfamily C member 8** |  |
| 2 | 11 | | Ace | AA | | AASGKLSTCR | 100% Ac- | 100.00 | Q8WVM0 | TFB1M_HUMAN | **Dimethyladenosine transferase 1, mitochondrial** |  |
| 2 | 21 | | Ace | AA | | AASGVEKSSKKKTEKKLAAR | N.D | N.D | Q8IXQ5 | KLHL7_HUMAN | **Kelch-like protein 7** |  |
| 2 | 11 | | Ace | AA | | AASQAVEEMR | 100% Ac- | 100.00 | O15160 | RPAC1_HUMAN | **DNA-directed RA polymerases I and III subunit RPAC1** |  |
| 2 | 59 | | AcD3 | AA | | AASQQQASAASSAAGVSGPSSAGGPGPQQQPQPPAQLVGPAQSGLLQQQQQDFDPVQR | 100% free | 0.00 | Q9NX70 | MED29_HUMAN | **Mediator of RA polymerase II transcription subunit 29** |  |
| 2 | 12 | | Ace | AA | | AASSLEQKLSR | 100% Ac- | 100.00 | O14733 | MP2K7_HUMAN | **Dual specificity mitogen-activated protein kinase kinase 7** |  |
| 2 | 13 | | Ace | AA | | AASSSGEKEKER | 100% Ac- | 100.00 | Q9NPJ6 | MED4_HUMAN | **Mediator of RA polymerase II transcription subunit 4** |  |
| 2 | 15 | | Ace | AA | | AASTDMAGLEESFR | 100% Ac- | 100.00 | Q9BW30 | TPPP3_HUMAN | **Tubulin polymerization-promoting protein family member 3** |  |
| 2 | 8 | | Ace | AA | | AASVEQR | 100% Ac- | 100.00 | Q96IU4 | ABHEB_HUMAN | **Abhydrolase domain-containing protein 14B** |  |
| 2 | 9 | | Ace | AA | | AASAAETR | 100% Ac- | 100.00 | Q7Z6J8 | UB2CB_HUMAN | **Ubiquitin-conjugating enzyme E2C-binding protein** |  |
| 2 | 17 | | Ace | AA | | AASAAAAELQASGGPR | 100% Ac- | 100.00 | Q9HCN4 | GPN1_HUMAN | **GPN-loop GTPase 1** |  |
| 2 | 32 | | Ace | AA | | AASAAAASAAAASAASGSPGPGEGSAGGEKR | 100% Ac- | 100.00 | Q13263 | TIF1B_HUMAN | **Transcription intermediary factor 1-beta** |  |
| 2 | 13 | | AcD3 | AA | | AATALLEAGLAR | Partial-Ac- | 48.88 | Q8WUK0 | PTPM1_HUMAN | **Protein-tyrosine phosphatase mitochondrial 1** |  |
| 2 | 17 | | Ace | AA | | AATASAGAGGIDGKPR | 100% Ac- | 100.00 | Q02978 | M2OM_HUMAN | **Mitochondrial 2-oxoglutarate/malate carrier protein** |  |
| 2 | 8 | | Ace | AA | | AATDIAR | 100% Ac- | 100.00 | Q14141 | SEPT6_HUMAN | **Septin-6** |  |
| 2 | 8 | | Ace | AA | | AATDLER | 100% Ac- | 100.00 | Q92599 | SEPT8_HUMAN | **Septin-8** |  |
| 2 | 15 | | Ace | AA | | AATFFGEVVKAPCR | 100% Ac- | 100.00 | O95456 | PSMG1_HUMAN | **Proteasome assembly chaperone 1** |  |
| 2 | 20 | | Ace | AA | | AATLDLKSKEEKDAELDKR | 100% Ac- | 100.00 | Q9Y3X0 | CCDC9_HUMAN | **Coiled-coil domain-containing protein 9** |  |
| 2 | 9 | | Ace | AA | | AATLLAAR | 100% Ac- | 100.00 | P28072 | PSB6_HUMAN | **Proteasome subunit beta type-6** |  |
| 2 | 28 | | Ace | AA | | AATMKKAAAEDVNVTFEDQQKINKFAR | 100% Ac- | 100.00 | Q9NQP4 | PFD4_HUMAN | **Prefoldin subunit 4** |  |
| 2 | 39 | | Ace | AA | | AATTANPEMTSDVPSLGPAIASGNSGPGIQGGGAIVQR | 100% Ac- | 100.00 | P27540 | ARNT_HUMAN | **Aryl hydrocarbon receptor nuclear translocator** |  |
| 2 | 17 | | Ace | AA | | AATAAAVVAEEDTELR | 100% Ac- | 100.00 | O95684 | FR1OP_HUMAN | **FGFR1 oncogene partner** |  |
| 2 | 17 | | Ace | AA | | AATAAEAVASGSGEPR | 100% Ac- | 100.00 | Q9NWT6 | HIF1N_HUMAN | **Hypoxia-inducible factor 1-alpha inhibitor** |  |
| 2 | 9 | | Ace | AA | | AAVAVAVR | 100% Ac- | 100.00 | Q9UI10 | EI2BD_HUMAN | **Translation initiation factor eIF-2B subunit delta** |  |
| 2 | 10 | | Ace | AA | | AAVDLEKLR | 100% Ac- | 100.00 | P17858 | K6PL_HUMAN | **6-phosphofructokinase, liver type** |  |
| 2 | 13 | | Ace | AA | | AAVDSDVESLPR | 100% Ac- | 100.00 | Q9H6E5 | TUT1_HUMAN | **U6 snRA-specific terminal uridylyltransferase 1** |  |
| 2 | 11 | | Ace | AA | | AAVGSGGYAR | 100% Ac- | 100.00 | O43379 | WDR62_HUMAN | **WD repeat-containing protein 62** |  |
| 2 | 19 | | Ace | AA | | AAVKDSCGKGEMATGNGR | 100% Ac- | 100.00 | P61758 | PFD3_HUMAN | **Prefoldin subunit 3** |  |
| 2 | 15 | | Ace | AA | | AAVKTLNPKAEVAR | 100% Ac- | 100.00 | P40227 | TCPZ_HUMAN | **T-complex protein 1 subunit zeta** |  |
| 2 | 10 | | Ace | AA | | AAVLESLLR | N.D | N.D | Q8IWY9 | CDAN1_HUMAN | **Codanin-1** |  |
| 2 | 13 | | Ace | AA | | AAVLKPVLLGLR | 100% Ac- | 100.00 | Q9BRP1 | PDD2L_HUMAN | **Programmed cell death protein 2-like** |  |
| 2 | 9 | | Ace | AA | | AAVLNAER | 100% Ac- | 100.00 | Q9H3P7 | GCP60_HUMAN | **Golgi resident protein GCP60** |  |
| 2 | 11 | | Ace | AA | | AAVLQQVLER | 100% Ac- | 100.00 | P12270 | TPR_HUMAN | **Nucleoprotein TPR** |  |
| 2 | 15 | | Ace | AA | | AAVPELLQQQEEDR | 100% Ac- | 100.00 | Q9Y5X3 | SNX5_HUMAN | **Sorting nexin-5** |  |
| 2 | 15 | | Ace | AA | | AAVPQNNLQEQLER | N.D | N.D | P54132 | BLM_HUMAN | **Bloom syndrome protein** |  |
| 2 | 13 | | Ace | AA | | AAVQMDPELAKR | 100% Ac- | 100.00 | Q9Y312 | CT004_HUMAN | **Uncharacterized protein C20orf4** |  |
| 2 | 25 | | Ace | AA | | AAVQAAEVKVDGSEPKLSKNELKR | 100% Ac- | 100.00 | Q15046 | SYK_HUMAN | **Lysyl-tRA synthetase** |  |
| 2 | 13 | | Ace | AA | | AAVVAATALKGR | 100% Ac- | 100.00 | Q8N490 | PNKD_HUMAN | **Probable hydrolase PKD** |  |
| 2 | 14 | | Ace | AA | | AAVAAGGLVGKGR | 100% Ac- | 100.00 | Q8IZD4 | DCP1B_HUMAN | **mRA-decapping enzyme 1B** |  |
| 2 | 19 | | AcD3 | AA | | AAVAATAAAKGNGGGGGR | N.D | N.D | Q6Y1H2 | PTPLB_HUMAN | **Protein-tyrosine phosphatase-like member B** |  |
| 2 | 10 | | Ace | AA | | AAYKLVLIR | 100% Ac- | 100.00 | P18669 | PGAM1_HUMAN | **Phosphoglycerate mutase 1** | Q8N0Y7 (2-10) |
| 2 | 19 | | Ace | AC | | ACGLVASNLNLKPGECLR | 100% Ac- | 100.00 | P09382 | LEG1_HUMAN | **Galectin-1** |  |
| 2 | 10 | | Ace | AC | | ACLLETPIR | 100% Ac- | 100.00 | Q8IUR7 | ARMC8_HUMAN | **Armadillo repeat-containing protein 8** |  |
| 2 | 22 | | Ace | AD | | ADDAGAAGGPGGPGGPGMGNR | 100% Ac- | 100.00 | P15880 | RS2_HUMAN | **40S ribosomal protein S2** |  |
| 2 | 31 | | Ace | AD | | ADDIDIEAMLEAPYKKDENKLSSANGHEER | 100% Ac- | 100.00 | Q14498 | RBM39_HUMAN | **RNA-binding protein 39** |  |
| 2 | 26 | | Ace | AD | | ADDLDFETGDAGASATFPMQCSALR | 100% Ac- | 100.00 | P63241 | IF5A1_HUMAN | **Eukaryotic translation initiation factor 5A-1** | Q6IS14 (2-26) |
| 2 | 28 | | Ace | AD | | ADDQGCIEEQGVEDSANEDSVDAKPDR | 100% Ac- | 100.00 | P55210 | CASP7_HUMAN | **Caspase-7** |  |
| 2 | 22 | | Ace | AD | | ADDVDQQQTTNTVEEPLDLIR | 100% Ac- | 100.00 | P62310 | LSM3_HUMAN | **U6 snRA-associated Sm-like protein LSm3** |  |
| 2 | 15 | | Ace | AD | | ADEALAGLDEGALR | 100% Ac- | 100.00 | P53814 | SMTN_HUMAN | **Smoothelin** |  |
| 2 | 9 | | Ace | AD | | ADEDLIFR | 100% Ac- | 100.00 | O00418 | EF2K_HUMAN | **Elongation factor 2 kinase** |  |
| 2 | 28 | | Ace | AD | | ADEEEDPTFEEENEEIGGGAEGGQGKR | 100% Ac- | 100.00 | Q15543 | TAF13_HUMAN | **Transcription initiation factor TFIID subunit 13** |  |
| 2 | 14 | | Ace | AD | | ADEEKLPPGWEKR | 100% Ac- | 100.00 | O15428 | PINL_HUMAN | **Putative PI1-like protein** | Q13526 (2-14) |
| 2 | 10 | | Ace | AD | | ADEELEALR | 100% Ac- | 100.00 | O14737 | PDCD5_HUMAN | **Programmed cell death protein 5** |  |
| 2 | 12 | | Ace | AD | | ADEIAKAQVAR | 100% Ac- | 100.00 | P49773 | HINT1_HUMAN | **Histidine triad nucleotide-binding protein 1** |  |
| 2 | 26 | | Ace | AD | | ADEIDFTTGDAGASSTYPMQCSALR | 100% Ac- | 100.00 | Q9GZV4 | IF5A2_HUMAN | **Eukaryotic translation initiation factor 5A-2** |  |
| 2 | 36 | | Ace | AD | | ADEKPKEGVKTENNDHINLKVAGQDGSVVQFKIKR | 100% Ac- | 100.00 | P61956 | SUMO2_HUMAN | **Small ubiquitin-related modifier 2** |  |
| 2 | 22 | | Ace | AD | | ADEAALALQPGGSPSAAGADR | N.D | N.D | Q96EB6 | SIRT1_HUMAN | **NAD-dependent deacetylase sirtuin-1** |  |
| 2 | 12 | | Ace | AD | | ADFAGPSSAGR | 100% Ac- | 100.00 | Q8IXW5 | RPAP2_HUMAN | **RNA polymerase II-associated protein 2** |  |
| 2 | 10 | | Ace | AD | | ADFDTYDDR | 100% Ac- | 100.00 | Q15056 | IF4H_HUMAN | **Eukaryotic translation initiation factor 4H** |  |
| 2 | 18 | | Ace | AD | | ADFLKGLPVYNKSNFSR | 100% Ac- | 100.00 | Q9BW61 | DDA1_HUMAN | **DET1- and DDB1-associated protein 1** |  |
| 2 | 11 | | Ace | AD | | ADGEEPEKKR | 100% Ac- | 100.00 | Q8TBC4 | UBA3_HUMAN | **EDD8-activating enzyme E1 catalytic subunit** |  |
| 2 | 14 | | Ace | AD | | ADGELNVDSLITR | 100% Ac- | 100.00 | P62140 | PP1B_HUMAN | **Serine/threonine-protein phosphatase PP1-beta catalytic subunit** |  |
| 2 | 16 | | Ace | AD | | ADGKAGDEKPEKSQR | 100% Ac- | 100.00 | Q8WW12 | PCNP_HUMAN | **PEST proteolytic signal-containing nuclear protein** |  |
| 2 | 12 | | Ace | AD | | ADGQVAELLLR | 100% Ac- | 100.00 | Q9Y285 | SYFA_HUMAN | **Phenylalanyl-tRA synthetase alpha chain** |  |
| 2 | 20 | | Ace | AD | | ADGSLTGGGLEAAAMAPER | 100% Ac- | 100.00 | Q8NEM2 | SHCBP_HUMAN | **SHC SH2 domain-binding protein 1** |  |
| 2 | 36 | | Ace | AD | | ADHSFSDGVPSDSVEAAKNASNTEKLTDQVMQNPR | N.D | N.D | Q99733 | NP1L4_HUMAN | **Nucleosome assembly protein 1-like 4** |  |
| 2 | 7 | | Ace | AD | | ADIIAR | 100% Ac- | 100.00 | O00170 | AIP_HUMAN | **AH receptor-interacting protein** | Q9P2P1 (1489-1494) |
| 2 | 8 | | Ace | AD | | ADIQTER | 100% Ac- | 100.00 | P62280 | RS11_HUMAN | **40S ribosomal protein S11** |  |
| 2 | 11 | | Ace | AD | | ADISLDELIR | 100% Ac- | 100.00 | Q9BY77 | PDIP3_HUMAN | **Polymerase delta-interacting protein 3** |  |
| 2 | 13 | | Ace | AD | | ADKEAGGSDGPR | 100% Ac- | 100.00 | O43301 | HS12A_HUMAN | **Heat shock 70 kDa protein 12A** |  |
| 2 | 24 | | Ace | AD | | ADKEKKKKESILDLSKYIDKTIR | 100% Ac- | 100.00 | Q9UK45 | LSM7_HUMAN | **U6 snRA-associated Sm-like protein LSm7** |  |
| 2 | 15 | | Ace | AD | | ADKEAAFDDAVEER | 100% Ac- | 100.00 | Q09028 | RBBP4_HUMAN | **Histone-binding protein RBBP4** |  |
| 2 | 17 | | Ace | AD | | ADKMDMSLDDIIKLNR | 100% Ac- | 100.00 | Q86V81 | THOC4_HUMAN | **THO complex subunit 4** |  |
| 2 | 40 | | Ace | AD | | ADKPDMGEIASFDKAKLKKTETQEKNTLPTKETIEQEKR | 100% Ac- | 100.00 | P63313 | TYB10_HUMAN | **Thymosin beta-10** |  |
| 2 | 17 | | Ace | AD | | ADKTPGGSQKASSKTR | 100% Ac- | 100.00 | O15042 | SR140_HUMAN | **U2-associated protein SR140** |  |
| 2 | 10 | | Ace | AD | | ADKVLKEKR | 100% Ac- | 100.00 | P29466 | CASP1_HUMAN | **Caspase-1** | Q5EG05 (2-10)^AQ5XLA6 (2-10) |
| 2 | 14 | | Ace | AD | | ADLAECNIKVMCR | 100% Ac- | 100.00 | P33176 | KINH_HUMAN | **Kinesin-1 heavy chain** |  |
| 2 | 15 | | Ace | AD | | ADLDKLNIDSIIQR | 100% Ac- | 100.00 | P36873 | PP1G_HUMAN | **Serine/threonine-protein phosphatase PP1-gamma catalytic subunit** |  |
| 2 | 15 | | Ace | AD | | ADLEEQLSDEEKVR | 100% Ac- | 100.00 | P47755 | CAZA2_HUMAN | **F-actin-capping protein subunit alpha-2** |  |
| 2 | 24 | | Ace | AD | | ADLLGSILSSMEKPPSLGDQETR | 100% Ac- | 100.00 | O75391 | SPAG7_HUMAN | **Sperm-associated antigen 7** |  |
| 2 | 23 | | Ace | AD | | ADLSLADALTEPSPDIEGEIKR | 100% Ac- | 100.00 | P27816 | MAP4_HUMAN | **Microtubule-associated protein 4** |  |
| 2 | 10 | | Ace | AD | | ADMQNLVER | 100% Ac- | 100.00 | Q01518 | CAP1_HUMAN | **Adenylyl cyclase-associated protein 1** |  |
| 2 | 11 | | Ace | AD | | ADNEKLDNQR | 100% Ac- | 100.00 | O00629 | IMA4_HUMAN | **Importin subunit alpha-4** |  |
| 2 | 16 | | Ace | AD | | ADPDVLTEVPAALKR | 100% Ac- | 100.00 | P29083 | T2EA_HUMAN | **General transcription factor IIE subunit 1** |  |
| 2 | 14 | | Ace | AD | | ADPKYADLPGIAR | 100% Ac- | 100.00 | Q13561 | DCTN2_HUMAN | **Dynactin subunit 2** |  |
| 2 | 17 | | Ace | AD | | ADPWQECMDYAVTLAR | 100% Ac- | 100.00 | P29218 | IMPA1_HUMAN | **Inositol monophosphatase** |  |
| 2 | 38 | | Ace | AD | | ADQLTEEQIAEFKEAFSLFDKDGDGTITTKELGTVMR | 100% Ac- | 100.00 | P62158 | CALM_HUMAN | **Calmodulin** |  |
| 2 | 15 | | Ace | AD | | ADSAELKQMVMSLR | 100% Ac- | 100.00 | O75925 | PIAS1_HUMAN | **E3 SUMO-protein ligase PIAS1** |  |
| 2 | 12 | | Ace | AD | | ADSELQLVEQR | 100% Ac- | 100.00 | P07741 | APT_HUMAN | **Adenine phosphoribosyltransferase** |  |
| 2 | 10 | | Ace | AD | | ADSGLLLKR | 100% Ac- | 100.00 | Q5SY16 | NOL9_HUMAN | **Nucleolar protein 9** |  |
| 2 | 27 | | Ace | AD | | ADSGTAGGAALAAPAPGPGSGGPGPR | 100% Ac- | 100.00 | Q96C90 | PP14B_HUMAN | **Protein phosphatase 1 regulatory subunit 14B** |  |
| 2 | 25 | | Ace | AD | | ADSKEGVLPLTAASTAPISFGFTR | 100% Ac- | 100.00 | Q92917 | GPKOW_HUMAN | **G patch domain and KOW motifs-containing protein** |  |
| 2 | 11 | | Ace | AD | | ADSSPALSLR | 100% Ac- | 100.00 | Q8IV50 | LYSM2_HUMAN | **LysM and putative peptidoglycan-binding domain-containing protein 2** |  |
| 2 | 14 | | Ace | AD | | ADTLESSLEDPLR | 100% Ac- | 100.00 | Q9H867 | CN138_HUMAN | **Uncharacterized protein C14orf138** |  |
| 2 | 20 | | Ace | AD | | ADTQYILPNDIGVSSLDCR | 100% Ac- | 100.00 | Q9NY33 | DPP3_HUMAN | **Dipeptidyl-peptidase 3** |  |
| 2 | 19 | | Ace | AD | | ADVFPGNDSTASQDVANR | 100% Ac- | 100.00 | P17252 | KPCA_HUMAN | **Protein kinase C alpha type** |  |
| 2 | 9 | | Ace | AD | | ADVLSVLR | 100% Ac- | 100.00 | Q6P1J9 | CDC73_HUMAN | **Parafibromin** |  |
| 2 | 15 | | Ace | AD | | ADVVVGKDKGGEQR | 100% Ac- | 100.00 | Q9NRG0 | CHRC1_HUMAN | **Chromatin accessibility complex protein 1** |  |
| 2 | 12 | | Ace | AD | | ADAAASPVGKR | 100% Ac- | 100.00 | Q7LBC6 | JHD2B_HUMAN | **JmjC domain-containing histone demethylation protein 2B** |  |
| 2 | 16 | | Ace | AD | | ADAAATAGAGGSGTR | N.D | N.D | Q7Z7C8 | TAF8_HUMAN | **Transcription initiation factor TFIID subunit 8** |  |
| 2 | 11 | | Ace | AD | | ADAAPQLGKR | 100% Ac- | 100.00 | Q96C86 | DCPS_HUMAN | **Scavenger mRA-decapping enzyme DcpS** |  |
| 2 | 17 | | Ace | AE | | AEAEESPGDPGTASPR | 100% Ac- | 100.00 | Q96EC8 | YIPF6_HUMAN | **Protein YIPF6** |  |
| 2 | 26 | | Ace | AE | | AEAGAGLSETVTETTVTVTTEPENR | 100% Ac- | 100.00 | O60927 | PP1RB_HUMAN | **Protein phosphatase 1 regulatory subunit 11** |  |
| 2 | 30 | | Ace | AE | | AEASATGACGEAMAAAEGSSGPAGLTLGR | 100% Ac- | 100.00 | Q99611 | SPS2_HUMAN | **Selenide, water dikinase 2** |  |
| 2 | 17 | | Ace | AE | | AEASSANLGSGCEEKR | 100% Ac- | 100.00 | Q6P1K2 | PMF1_HUMAN | **Polyamine-modulated factor 1** |  |
| 2 | 7 | | Ace | AE | | AEAVER | 100% Ac- | 100.00 | A4D1P6 | WDR91_HUMAN | **WD repeat-containing protein 91** |  |
| 2 | 9 | | Ace | AE | | AEAVKPQR | 100% Ac- | 100.00 | Q9HCE0 | K1632_HUMAN | **UPF0493 protein KIAA1632** |  |
| 2 | 16 | | Ace | AE | | AEDEPDAKSPKTGGR | 100% Ac- | 100.00 | P48382 | RFX5_HUMAN | **DNA-binding protein RFX5** |  |
| 2 | 20 | | Ace | AE | | AEDMETKIKNYKTAPFDSR | 100% Ac- | 100.00 | P14854 | CX6B1_HUMAN | **Cytochrome c oxidase subunit VIb isoform 1** |  |
| 2 | 33 | | Ace | AE | | AEEGIAAGGVMDVNTALQEVLKTALIHDGLAR | 100% Ac- | 100.00 | P25398 | RS12_HUMAN | **40S ribosomal protein S12** |  |
| 2 | 9 | | Ace | AE | | AEELVLER | 100% Ac- | 100.00 | P21980 | TGM2_HUMAN | **Protein-glutamine gamma-glutamyltransferase 2** |  |
| 2 | 29 | | Ace | AE | | AEEQPQVELFVKAGSDGAKIGNCPFSQR | 100% Ac- | 100.00 | O00299 | CLIC1_HUMAN | **Chloride intracellular channel protein 1** |  |
| 2 | 8 | | Ace | AE | | AEEQVNR | 100% Ac- | 100.00 | Q9NQE9 | HINT3_HUMAN | **Histidine triad nucleotide-binding protein 3** |  |
| 2 | 11 | | Ace | AE | | AEFLDDQETR | 100% Ac- | 100.00 | O14545 | TRAD1_HUMAN | **TRAF-type zinc finger domain-containing protein 1** |  |
| 2 | 14 | | Ace | AE | | AEFTSYKETASSR | 100% Ac- | 100.00 | Q86V48 | LUZP1_HUMAN | **Leucine zipper protein 1** |  |
| 2 | 14 | | Ace | AE | | AEGGGPEPGEQER | 100% Ac- | 100.00 | Q14CS0 | UBX2B_HUMAN | **UBX domain-containing protein 2B** |  |
| 2 | 13 | | Ace | AE | | AEGGAADLDTQR | 100% Ac- | 100.00 | Q9Y4E8 | UBP15_HUMAN | **Ubiquitin carboxyl-terminal hydrolase 15** |  |
| 2 | 25 | | Ace | AE | | AEGTAEAPLENGGGGDSGAGALER | 100% Ac- | 100.00 | Q96G46 | DUS3L_HUMAN | **tRA-dihydrouridine synthase 3-like** |  |
| 2 | 8 | | Ace | AE | | AEIIQER | 100% Ac- | 100.00 | Q9NYH9 | UTP6_HUMAN | **U3 small nucleolar RA-associated protein 6 homolog** |  |
| 2 | 9 | | Ace | AE | | AEISDLDR | 100% Ac- | 100.00 | P60510 | PP4C_HUMAN | **Serine/threonine-protein phosphatase 4 catalytic subunit** |  |
| 2 | 14 | | Ace | AE | | AELDLMAPGPLPR | N.D | N.D | O95361 | TRI16_HUMAN | **Tripartite motif-containing protein 16** |  |
| 2 | 14 | | Ace | AE | | AELGEADEAELQR | 100% Ac- | 100.00 | Q9Y5J9 | TIM8B_HUMAN | **Mitochondrial import inner membrane translocase subunit Tim8 B** |  |
| 2 | 30 | | Ace | AE | | AELIQKKLQGEVEKYQQLQKDLSKSMSGR | 100% Ac- | 100.00 | O15212 | PFD6_HUMAN | **Prefoldin subunit 6** |  |
| 2 | 17 | | Ace | AE | | AELQMLLEEEIPSGKR | 100% Ac- | 100.00 | Q8IZP0 | ABI1_HUMAN | **Abl interactor 1** |  |
| 2 | 17 | | Ace | AE | | AELSEEALLSVLPTIR | 100% Ac- | 100.00 | P45974 | UBP5_HUMAN | **Ubiquitin carboxyl-terminal hydrolase 5** |  |
| 2 | 17 | | Ace | AE | | AELTALESLIEMGFPR | 100% Ac- | 100.00 | Q04323 | UBXN1_HUMAN | **UBX domain-containing protein 1** |  |
| 2 | 9 | | Ace | AE | | AELTVEVR | 100% Ac- | 100.00 | P51114 | FXR1_HUMAN | **Fragile X mental retardation syndrome-related protein 1** |  |
| 2 | 18 | | Ace | AE | | AEMDPVAEFPQPPGAAR | 100% Ac- | 100.00 | Q9HAB8 | PPCS_HUMAN | **Phosphopantothenate--cysteine ligase** |  |
| 2 | 24 | | Ace | AE | | AEMGSKGVTAGKIASNVQKKLTR | 100% Ac- | 100.00 | O00499 | BIN1_HUMAN | **Myc box-dependent-interacting protein 1** |  |
| 2 | 14 | | Ace | AE | | AENLLDGPPNPKR | 100% Ac- | 100.00 | Q92793 | CBP_HUMAN | **CREB-binding protein** |  |
| 2 | 13 | | Ace | AE | | AENSVLTSTTGR | 100% Ac- | 100.00 | Q9H8V3 | ECT2_HUMAN | **Protein ECT2** |  |
| 2 | 16 | | Ace | AE | | AEPASVAAESLAGSR | 100% Ac- | 100.00 | Q9NQT5 | EXOS3_HUMAN | **Exosome complex exonuclease RRP40** |  |
| 2 | 38 | | Ace | AE | | AEPQPPSGGLTDEAALSCCSDADPSTKDFLLQQTMLR | 100% Ac- | 100.00 | Q04760 | LGUL_HUMAN | **Lactoylglutathione lyase** |  |
| 2 | 17 | | Ace | AE | | AEPSQAPTPAPAAQPR | 100% Ac- | 100.00 | Q92830 | GCNL2_HUMAN | **General control of amino acid synthesis protein 5-like 2** |  |
| 2 | 49 | | Ace | AE | | AEQEPTAEQLAQIAAENEEDEHSVNYKPPAQKSIQEIQELDKDDESLR | 100% Ac- | 100.00 | P52565 | GDIR1_HUMAN | **Rho GDP-dissociation inhibitor 1** |  |
| 2 | 9 | | Ace | AE | | AEQVALSR | 100% Ac- | 100.00 | P11413 | G6PD_HUMAN | **Glucose-6-phosphate 1-dehydrogenase** |  |
| 2 | 16 | | Ace | AE | | AEQVLPQALYLSNMR | 100% Ac- | 100.00 | P21580 | TNAP3_HUMAN | **Tumor necrosis factor, alpha-induced protein 3** |  |
| 2 | 13 | | Ace | AE | | AESDWDTVTVLR | 100% Ac- | 100.00 | O60869 | EDF1_HUMAN | **Endothelial differentiation-related factor 1** |  |
| 2 | 8 | | Ace | AE | | AESIIIR | 100% Ac- | 100.00 | Q8TAT6 | NPL4_HUMAN | **Nuclear protein localization protein 4 homolog** |  |
| 2 | 10 | | Ace | AE | | AESSDKLYR | 100% Ac- | 100.00 | P09874 | PARP1_HUMAN | **Poly [ADP-ribose] polymerase 1** |  |
| 2 | 17 | | Ace | AE | | AESSESFTMASSPAQR | Partial-Ac- | 88.61 | P49736 | MCM2_HUMAN | **DNA replication licensing factor MCM2** |  |
| 2 | 11 | | Ace | AE | | AETEALSKLR | 100% Ac- | 100.00 | Q9BUP3 | HTAI2_HUMAN | **Oxidoreductase HTATIP2** |  |
| 2 | 22 | | Ace | AE | | AETLEFNDVYQEVKGSMNDGR | 100% Ac- | 100.00 | Q08945 | SSRP1_HUMAN | **FACT complex subunit SSRP1** |  |
| 2 | 28 | | Ace | AE | | AETLPGSGDSGPGTASLGPGVAETGTR | 100% Ac- | 100.00 | Q14151 | SAFB2_HUMAN | **Scaffold attachment factor B2** |  |
| 2 | 14 | | Ace | AE | | AETSEEVAVLVQR | 100% Ac- | 100.00 | Q7Z6K3 | PTAR1_HUMAN | **Protein prenyltransferase alpha subunit repeat-containing protein 1** |  |
| 2 | 9 | | Ace | AE | | AETVADTR | 100% Ac- | 100.00 | O60493 | SNX3_HUMAN | **Sorting nexin-3** |  |
| 2 | 10 | | Ace | AE | | AEVEETLKR | 100% Ac- | 100.00 | Q8TF09 | DLRB2_HUMAN | **Dynein light chain roadblock-type 2** | Q9NP97 (2-10) |
| 2 | 10 | | Ace | AE | | AEVEQKKKR | 100% Ac- | 100.00 | P62841 | RS15_HUMAN | **40S ribosomal protein S15** |  |
| 2 | 11 | | Ace | AE | | AEVGEDSGAR | 100% Ac- | 100.00 | P85037 | FOXK1_HUMAN | **Forkhead box protein K1** |  |
| 2 | 12 | | Ace | AE | | AEVGEIIEGCR | N.D | N.D | Q92993 | TIP60_HUMAN | **Histone acetyltransferase HTATIP** |  |
| 2 | 21 | | Ace | AE | | AEVKVKVQPPDADPVEIENR | N.D | N.D | Q9H1D9 | RPC6_HUMAN | **DNA-directed RA polymerase III subunit RPC6** |  |
| 2 | 12 | | Ace | AE | | AEVQVLVLDGR | Partial-Ac- | 96.70 | P40429 | RL13A_HUMAN | **60S ribosomal protein L13a** |  |
| 2 | 19 | | Ace | AE | | AEVSIDQSKLPGVKEVCR | 100% Ac- | 100.00 | Q8IVM0 | CCD50_HUMAN | **Coiled-coil domain-containing protein 50** |  |
| 2 | 9 | | Ace | AE | | AEYDLTTR | 100% Ac- | 100.00 | P60228 | EIF3E_HUMAN | **Eukaryotic translation initiation factor 3 subunit E** |  |
| 2 | 10 | | Ace | AE | | AEAALEAVR | 100% Ac- | 100.00 | P0C870 | JMJD7_HUMAN | **JmjC domain-containing protein 7** |  |
| 2 | 16 | | Ace | AE | | AEAALLLLPEAAAER | 100% Ac- | 100.00 | Q96JB2 | COG3_HUMAN | **Conserved oligomeric Golgi complex subunit 3** |  |
| 2 | 7 | | Ace | AF | | AFANLR | 100% Ac- | 100.00 | O43175 | SERA_HUMAN | **D-3-phosphoglycerate dehydrogenase** |  |
| 2 | 8 | | Ace | AF | | AFSKGFR | 100% Ac- | 100.00 | O43426 | SYNJ1_HUMAN | **Synaptojanin-1** |  |
| 2 | 11 | | Ace | AF | | AFTNYSSLNR | 100% Ac- | 100.00 | Q9Y6X9 | MORC2_HUMAN | **MORC family CW-type zinc finger protein 2** |  |
| 2 | 33 | | Ace | AG | | AGAGPAPGLPGAGGPVVPGPGAGIPGKSGEER | 100% Ac- | 100.00 | Q9BTD8 | RBM42_HUMAN | **RA-binding protein 42** |  |
| 2 | 11 | | Ace | AG | | AGAGPTMLLR | 100% Ac- | 100.00 | Q9BRA0 | LSMD1_HUMAN | **LSM domain-containing protein 1** |  |
| 2 | 22 | | Ace | AG | | AGAGSAAVSGAGTPVAGPTGR | 100% Ac- | 100.00 | O95295 | SNAPN_HUMAN | **SARE-associated protein Snapin** |  |
| 2 | 13 | | Ace | AG | | AGAPTVSLPELR | 100% Ac- | 100.00 | Q8NFU3 | KAT_HUMAN | **Putative thiosulfate sulfurtransferase KAT** |  |
| 2 | 18 | | Ace | AG | | AGDLSAGFFMEELNTYR | 100% Ac- | 100.00 | P19525 | E2AK2_HUMAN | **Interferon-induced, double-stranded RA-activated protein kinase** |  |
| 2 | 22 | | Ace | AG | | AGGGAGDPGLGAAAAPAPETR | Partial-Ac- | 65.28 | Q13637 | RAB32_HUMAN | **Ras-related protein Rab-32** |  |
| 2 | 11 | | Ace | AG | | AGGGGDLSTR | 100% Ac- | 100.00 | P23497 | SP100_HUMAN | **Nuclear autoantigen Sp-100** |  |
| 2 | 20 | | AcD3 | AG | | AGGKAGKDSGKAKAKAVSR | Partial-Ac- | 7.20 | Q71UI9 | H2AV_HUMAN | **Histone H2A.V** |  |
| 2 | 20 | | AcD3 | AG | | AGGKAGKDSGKAKTKAVSR | 100% free | 0.00 | P0C0S5 | H2AZ_HUMAN | **Histone H2A.Z** |  |
| 2 | 24 | | Ace | AG | | AGILFEDIFDVKDIDPEGKKFDR | N.D | N.D | P52434 | RPAB3_HUMAN | **DNA-directed RA polymerases I, II, and III subunit RPABC3** |  |
| 2 | 12 | | Ace | AG | | AGIAAKLAKDR | 100% Ac- | 100.00 | P17655 | CAN2_HUMAN | **Calpain-2 catalytic subunit** |  |
| 2 | 18 | | Ace | AG | | AGKQAVSASGKWLDGIR | 100% Ac- | 100.00 | P14927 | QCR7_HUMAN | **Cytochrome b-c1 complex subunit 7** |  |
| 2 | 13 | | Ace | AG | | AGLELLSDQGYR | Partial-Ac- | 96.97 | Q9NPD3 | EXOS4_HUMAN | **Exosome complex exonuclease RRP41** |  |
| 2 | 18 | | Ace | AG | | AGLEVLFASAAPAITCR | 100% Ac- | 100.00 | Q92530 | PSMF1_HUMAN | **Proteasome inhibitor PI31 subunit** |  |
| 2 | 12 | | Ace | AG | | AGLNSLEAVKR | 100% Ac- | 100.00 | P67936 | TPM4_HUMAN | **Tropomyosin alpha-4 chain** |  |
| 2 | 10 | | Ace | AG | | AGLSDLELR | 100% Ac- | 100.00 | Q8NC56 | LEMD2_HUMAN | **LEM domain-containing protein 2** |  |
| 2 | 9 | | Ace | AG | | AGLTDLQR | 100% Ac- | 100.00 | O75935 | DCTN3_HUMAN | **Dynactin subunit 3** |  |
| 2 | 10 | | Ace | AG | | AGLTLFVGR | 100% Ac- | 100.00 | Q9NW13 | RBM28_HUMAN | **RNA-binding protein 28** |  |
| 2 | 10 | | Ace | AG | | AGNFDSEER | 100% Ac- | 100.00 | P46108 | CRK_HUMAN | **Proto-oncogene C-crk** |  |
| 2 | 10 | | Ace | AG | | AGPEGFQYR | N.D | N.D | O00459 | P85B_HUMAN | **Phosphatidylinositol 3-kinase regulatory subunit beta** |  |
| 2 | 21 | | Ace | AG | | AGPGSTGGQIGAAALAGGAR | 100% Ac- | 100.00 | Q8N653 | LZTR1_HUMAN | **Leucine-zipper-like transcriptional regulator 1** |  |
| 2 | 11 | | Ace | AG | | AGPLQGGGAR | Partial-Ac- | 91.33 | Q9P015 | RM15_HUMAN | **39S ribosomal protein L15, mitochondrial** |  |
| 2 | 8 | | Ace | AG | | AGPVKDR | 100% Ac- | 100.00 | Q9H633 | RPP21_HUMAN | **Ribonuclease P protein subunit p21** |  |
| 2 | 8 | | Ace | AG | | AGPVSLR | 100% Ac- | 100.00 | Q8ND04 | CQ071_HUMAN | **UPF0487 protein C17orf71** |  |
| 2 | 7 | | Ace | AG | | AGQAFR | 100% Ac- | 100.00 | P61604 | CH10_HUMAN | **10 kDa heat shock protein, mitochondrial** | Q86WI1 (2465-2470) |
| 2 | 10 | | Ace | AG | | AGQEDPVQR | 100% Ac- | 100.00 | Q8WUW1 | BRK1_HUMAN | **Probable protein BRICK1** |  |
| 2 | 17 | | Ace | AG | | AGSQDIFDAIVMADER | 100% Ac- | 100.00 | Q8WV07 | ORAV1_HUMAN | **Oral cancer overexpressed protein 1** |  |
| 2 | 13 | | Ace | AG | | AGSSEEAPDYGR | N.D | N.D | Q9NRG1 | PRDC1_HUMAN | **Phosphoribosyltransferase domain-containing protein 1** |  |
| 2 | 35 | | Ace | AG | | AGSVADSDAVVKLDDGHLNNSLSSPVQADVYFPR | 100% Ac- | 100.00 | Q3ZCW2 | LEGL_HUMAN | **Galectin-related protein** |  |
| 2 | 17 | | Ace | AG | | AGSYPEGAPAILADKR | 100% Ac- | 100.00 | Q6P1Q9 | MTL2B_HUMAN | **Methyltransferase-like protein 2B** |  |
| 2 | 17 | | Ace | AG | | AGSYPEGAPAVLADKR | 100% Ac- | 100.00 | Q96IZ6 | MTL2A_HUMAN | **Methyltransferase-like protein 2A** |  |
| 2 | 10 | | Ace | AG | | AGTGLLALR | 100% Ac- | 100.00 | Q9BT30 | ALKB7_HUMAN | **Alkylated DA repair protein alkB homolog 7** |  |
| 2 | 29 | | Ace | AG | | AGTGLVAGEVVVDALPYFDQGYEAPGVR | 100% Ac- | 100.00 | O75934 | SPF27_HUMAN | **Pre-mRA-splicing factor SPF27** |  |
| 2 | 13 | | Ace | AG | | AGTVVLDDVELR | 100% Ac- | 100.00 | P25205 | MCM3_HUMAN | **DNA replication licensing factor MCM3** |  |
| 2 | 10 | | Ace | AG | | AGAAAESGR | 100% Ac- | 100.00 | Q96EN8 | MOCOS_HUMAN | **Molybdenum cofactor sulfurase** |  |
| 2 | 9 | | Ace | AG | | AGAAMAER | 100% Ac- | 100.00 | Q86UD0 | CI140_HUMAN | **Protein C9orf140** |  |
| 2 | 11 | | AcD3 | AI | | AICQFFLQGR | 100% free | 0.00 | O15504 | NUPL2_HUMAN | **Nucleoporin-like 2** |  |
| 2 | 10 | | AcD3 | AK | | AKAKKVGAR | 100% free | 0.00 | P78316 | NOP14_HUMAN | **Nucleolar protein 14** |  |
| 2 | 13 | | Ace | AK | | AKATTIKEALAR | 100% Ac- | 100.00 | Q4LDG9 | DNAL1_HUMAN | **Dynein light chain 1, axonemal** |  |
| 2 | 26 | | Ace | AK | | AKFMTPVIQDNPSGWGPCAVPEQFR | N.D | N.D | O15371 | EIF3D_HUMAN | **Eukaryotic translation initiation factor 3 subunit D** |  |
| 2 | 24 | | AcD3 | AK | | AKGDPKKPKGKMSAYAFFVQTCR | Partial-Ac- | 18.80 | O15347 | HMGB3_HUMAN | **High mobility group protein B3** | P0C6E5 (2-24) |
| 2 | 7 | | AcD3 | AK | | AKIKAR | Partial-Ac- | 12.10 | P42766 | RL35_HUMAN | **60S ribosomal protein L35** |  |
| 2 | 12 | | Ace | AK | | AKISSPTETER | 100% Ac- | 100.00 | P31949 | S10AB_HUMAN | **Protein S100-A11** |  |
| 2 | 7 | | AcD3 | AK | | AKNKLR | 100% free | 0.00 | Q8N0T1 | CH059_HUMAN | **Uncharacterized protein C8orf59** |  |
| 2 | 11 | | Ace | AK | | AKPAQGAKYR | 100% Ac- | 100.00 | P08133 | ANXA6_HUMAN | **Annexin A6** |  |
| 2 | 8 | | Ace | AK | | AKPCGVR | 100% Ac- | 100.00 | Q9UL46 | PSME2_HUMAN | **Proteasome activator complex subunit 2** |  |
| 2 | 11 | | Ace | AK | | AKPLTDQEKR | 100% Ac- | 100.00 | P06737 | PYGL_HUMAN | **Glycogen phosphorylase, liver form** |  |
| 2 | 11 | | Ace | AK | | AKPLTDSEKR | 100% Ac- | 100.00 | P11216 | PYGB_HUMAN | **Glycogen phosphorylase, brain form** |  |
| 2 | 25 | | Ace | AK | | AKPAATAAAASEELSQVPDEELLR | N.D | N.D | A6NKD9 | CC85C_HUMAN | **Coiled-coil domain-containing protein 85C** |  |
| 2 | 8 | | Ace | AK | | AKQLQAR | 100% Ac- | 100.00 | Q16773 | KAT1_HUMAN | **Kynurenine--oxoglutarate transaminase 1** |  |
| 2 | 11 | | Ace | AK | | AKSNGENGPR | 100% Ac- | 100.00 | Q9BXI6 | TB10A_HUMAN | **TBC1 domain family member 10A** |  |
| 2 | 24 | | Ace | AK | | AKVAKDLNPGVKKMSLGQLQSAR | 100% Ac- | 100.00 | Q9NZU5 | LMCD1_HUMAN | **LIM and cysteine-rich domains protein 1** |  |
| 2 | 10 | | Ace | AK | | AKWGEGDPR | 100% Ac- | 100.00 | O95433 | AHSA1_HUMAN | **Activator of 90 kDa heat shock protein ATPase homolog 1** |  |
| 2 | 8 | | Ace | AL | | ALADSTR | 100% Ac- | 100.00 | Q96CX2 | KCD12_HUMAN | **BTB/POZ domain-containing protein KCTD12** |  |
| 2 | 21 | | Ace | AL | | ALDGPEQMELEEGKAGSGLR | 100% Ac- | 100.00 | P62195 | PRS8_HUMAN | **26S protease regulatory subunit 8** |  |
| 2 | 8 | | Ace | AL | | ALDVKSR | 100% Ac- | 100.00 | P50613 | CDK7_HUMAN | **Cell division protein kinase 7** |  |
| 2 | 9 | | Ace | AL | | ALEGMSKR | 100% Ac- | 100.00 | P42696 | RBM34_HUMAN | **RNA-binding protein 34** |  |
| 2 | 11 | | Ace | AL | | ALETVPKDLR | 100% Ac- | 100.00 | P63272 | SPT4H_HUMAN | **Transcription elongation factor SPT4** |  |
| 2 | 20 | | Ace | AL | | ALFPAFAGLSEAPDGGSSR | 100% Ac- | 100.00 | Q9H7Z3 | CN102_HUMAN | **UPF0614 protein C14orf102** |  |
| 2 | 8 | | Ace | AL | | ALFYVAR | N.D | N.D | Q8N8A6 | DDX51_HUMAN | **ATP-dependent RA helicase DDX51** |  |
| 2 | 12 | | Ace | AL | | ALKMVKGSIDR | 100% Ac- | 100.00 | O14617 | AP3D1_HUMAN | **AP-3 complex subunit delta-1** |  |
| 2 | 8 | | Ace | AL | | ALLCYNR | 100% Ac- | 100.00 | Q9UHD1 | CHRD1_HUMAN | **Cysteine and histidine-rich domain-containing protein 1** |  |
| 2 | 27 | | Ace | AL | | ALNGAEVDDFSWEPPTEAETKVLQAR | 100% Ac- | 100.00 | O60232 | SSA27_HUMAN | **Sjoegren syndrome/scleroderma autoantigen 1** |  |
| 2 | 9 | | Ace | AL | | ALSCTLNR | 100% Ac- | 100.00 | Q7Z4G4 | TRM11_HUMAN | **tRA guanosine-2'-O-methyltransferase TRM11 homolog** |  |
| 2 | 9 | | Ace | AL | | ALSGNCSR | 100% Ac- | 100.00 | Q68DU8 | KCD16_HUMAN | **BTB/POZ domain-containing protein KCTD16** |  |
| 2 | 8 | | Ace | AL | | ALSKGLR | 100% Ac- | 100.00 | O15056 | SYNJ2_HUMAN | **Synaptojanin-2** | Q8IX21 (644-650) |
| 2 | 40 | | AcD3 | AL | | ALSMPLNGLKEEDKEPLIELFVKAGSDGESIGNCPFSQR | 100% free | 0.00 | Q9Y696 | CLIC4_HUMAN | **Chloride intracellular channel protein 4** |  |
| 2 | 11 | | Ace | AL | | ALSQGLLTFR | 100% Ac- | 100.00 | A2RRD8 | ZN320_HUMAN | **Zinc finger protein 320** | Q96IR2 (2-11) |
| 2 | 10 | | AcD3 | AL | | ALSTIVSQR | 100% free | 0.00 | Q5EE01 | CUG2_HUMAN | **Cancer-up-regulated gene 2 protein** |  |
| 2 | 25 | | Ace | AL | | ALTSFLPAPTQLSQDQLEAEEKAR | 100% Ac- | 100.00 | Q13573 | SNW1_HUMAN | **SW domain-containing protein 1** |  |
| 2 | 8 | | Ace | AL | | ALVTLQR | 100% Ac- | 100.00 | Q8WYL5 | SSH1_HUMAN | **Protein phosphatase Slingshot homolog 1** |  |
| 2 | 28 | | Ace | AM | | AMDQVNALCEQLVKAVTVMMDPNSTQR | 100% Ac- | 100.00 | Q9HAV4 | XPO5_HUMAN | **Exportin-5** |  |
| 2 | 10 | | Ace | AM | | AMDSSLQAR | 100% Ac- | 100.00 | Q99661 | KIF2C_HUMAN | **Kinesin-like protein KIF2C** |  |
| 2 | 10 | | Ace | AM | | AMDSSLQAR | 100% Ac- | 100.00 | Q99661 | KIF2C_HUMAN | **Kinesin-like protein KIF2C** |  |
| 2 | 8 | | Ace | AM | | AMFEQMR | 100% Ac- | 100.00 | Q9UBQ5 | EIF3K_HUMAN | **Eukaryotic translation initiation factor 3 subunit K** |  |
| 2 | 8 | | Ace | AM | | AMMVFPR | 100% Ac- | 100.00 | Q9H0B6 | KLC2_HUMAN | **Kinesin light chain 2** |  |
| 2 | 9 | | Ace | AM | | AMQKIFAR | 100% Ac- | 100.00 | P13929 | ENOB_HUMAN | **Beta-enolase** |  |
| 2 | 27 | | Ace | AM | | AMQMQLEANADTSVEEESFGPQPISR | N.D | N.D | Q06609 | RAD51_HUMAN | **DNA repair protein RAD51 homolog 1** |  |
| 2 | 8 | | Ace | AM | | AMQAAKR | 100% Ac- | 100.00 | Q9Y3B4 | PM14_HUMAN | **Pre-mRA branch site protein p14** |  |
| 2 | 22 | | Ace | AM | | AMSSGGSGGGVPEQEDSVLFR | N.D | N.D | Q16637 | SMN_HUMAN | **Survival motor neuron protein** |  |
| 2 | 12 | | Ace | AN | | ANALASATCER | 100% Ac- | 100.00 | P48059 | LIMS1_HUMAN | **LIM and senescent cell antigen-like-containing domain protein 1** | Q9HB10 (64-74) |
| 2 | 16 | | Ace | AN | | ANDSGGPGGPSPSER | 100% Ac- | 100.00 | Q9GZT9 | EGLN1_HUMAN | **Egl nine homolog 1** |  |
| 2 | 19 | | Ace | AN | | ANDSPAKSLVDIDLSSLR | 100% Ac- | 100.00 | O95819 | M4K4_HUMAN | **Mitogen-activated protein kinase kinase kinase kinase 4** |  |
| 2 | 8 | | Ace | AN | | ANIAVQR | 100% Ac- | 100.00 | P61086 | UBE2K_HUMAN | **Ubiquitin-conjugating enzyme E2 K** |  |
| 2 | 12 | | Ace | AN | | ANKGPSYGMSR | 100% Ac- | 100.00 | Q01995 | TAGL_HUMAN | **Transgelin** |  |
| 2 | 10 | | Ace | AN | | ANLEESFPR | 100% Ac- | 100.00 | Q14690 | RRP5_HUMAN | **Protein RRP5 homolog** |  |
| 2 | 10 | | Ace | AN | | ANMQGLVER | 100% Ac- | 100.00 | P40123 | CAP2_HUMAN | **Adenylyl cyclase-associated protein 2** |  |
| 2 | 10 | | Ace | AN | | ANNDAVLKR | 100% Ac- | 100.00 | Q12904 | MCA1_HUMAN | **Multisynthetase complex auxiliary component p43** |  |
| 2 | 15 | | Ace | AN | | ANSANTNTVPKLYR | 100% Ac- | 100.00 | P52655 | TF2AA_HUMAN | **Transcription initiation factor IIA subunit 1** |  |
| 2 | 30 | | Ace | AN | | ANSGCKDVTGPDEESFLYFAYGSNLLTER | 100% Ac- | 100.00 | O75223 | GGCT_HUMAN | **Gamma-glutamylcyclotransferase** |  |
| 2 | 13 | | Ace | AN | | ANSTGKAPPDER | 100% Ac- | 100.00 | Q68CP9 | ARID2_HUMAN | **AT-rich interactive domain-containing protein 2** |  |
| 2 | 29 | | Ace | AN | | ANVADTKLYDILGVPPGASENELKKAYR | 100% Ac- | 100.00 | O60884 | DNJA2_HUMAN | **DnaJ homolog subfamily A member 2** |  |
| 2 | 17 | | AcD3 | AP | | APAEILNGKEISAQIR | 100% free | 0.00 | P11586 | C1TC_HUMAN | **C-1-tetrahydrofolate synthase, cytoplasmic** |  |
| 2 | 14 | | AcD3 | AP | | APAKKGGEKKKGR | 100% free | 0.00 | P62899 | RL31_HUMAN | **60S ribosomal protein L31** |  |
| 2 | 15 | | AcD3 | AP | | APAMQPAEIQFAQR | 100% free | 0.00 | Q14684 | RRP1B_HUMAN | **Ribosomal RA processing protein 1 homolog B** |  |
| 2 | 18 | | AcD3 | AP | | APDPVAAETAAQGPTPR | 100% free | 0.00 | O60427 | FADS1_HUMAN | **Fatty acid desaturase 1** |  |
| 2 | 11 | | AcD3 | AP | | APGEVTITVR | 100% free | 0.00 | Q3KRA6 | CB076_HUMAN | **UPF0538 protein C2orf76** |  |
| 2 | 22 | | AcD3 | AP | | APGQLALFSVSDKTGLVEFAR | 100% free | 0.00 | P31939 | PUR9_HUMAN | **Bifunctional purine biosynthesis protein PURH** |  |
| 2 | 16 | | AcD3 | AP | | APKQDPKPKFQEGER | 100% free | 0.00 | Q9UBU8 | MO4L1_HUMAN | **Mortality factor 4-like protein 1** |  |
| 2 | 14 | | AcD3 | AP | | APLDLDKYVEIAR | 100% free | 0.00 | O00743 | PPP6_HUMAN | **Serine/threonine-protein phosphatase 6 catalytic subunit** |  |
| 2 | 9 | | AcD3 | AP | | APLGGAPR | 100% free | 0.00 | Q15126 | PMVK_HUMAN | **Phosphomevalonate kinase** |  |
| 2 | 9 | | AcD3 | AP | | APLLEYER | 100% free | 0.00 | Q92889 | XPF_HUMAN | **DNA repair endonuclease XPF** |  |
| 2 | 20 | | AcD3 | AP | | APSGLKAVVGEKILSGVIR | 100% free | 0.00 | Q15833 | STXB2_HUMAN | **Syntaxin-binding protein 2** |  |
| 2 | 11 | | AcD3 | AP | | APSTPLLTVR | 100% free | 0.00 | Q9BY44 | EIF2A_HUMAN | **Eukaryotic translation initiation factor 2A** |  |
| 2 | 17 | | AcD3 | AP | | APSVPAAEPEYPKGIR | 100% free | 0.00 | P54819 | KAD2_HUMAN | **Adenylate kinase isoenzyme 2, mitochondrial** |  |
| 2 | 11 | | AcD3 | AP | | APTIQTQAQR | 100% free | 0.00 | Q8N7H5 | PAF1_HUMAN | **RNA polymerase II-associated factor 1 homolog** |  |
| 2 | 14 | | Ace | AQ | | AQAKINAKANEGR | 100% Ac- | 100.00 | O95816 | BAG2_HUMAN | **BAG family molecular chaperone regulator 2** |  |
| 2 | 12 | | Ace | AQ | | AQALSEEEFQR | 100% Ac- | 100.00 | Q4V328 | GRAP1_HUMAN | **GRIP1-associated protein 1** |  |
| 2 | 13 | | Ace | AQ | | AQDQGEKENPMR | 100% Ac- | 100.00 | P62913 | RL11_HUMAN | **60S ribosomal protein L11** |  |
| 2 | 25 | | Ace | AQ | | AQESPKNSAAEIPVTSNGEVDDSR | 100% Ac- | 100.00 | P53367 | ARFP1_HUMAN | **Arfaptin-1** |  |
| 2 | 25 | | Ace | AQ | | AQETNQTPGPMLCSTGCGFYGNPR | N.D | N.D | O76080 | ZFAN5_HUMAN | **A1-type zinc finger protein 5** |  |
| 2 | 14 | | Ace | AQ | | AQEVSEYLSQNPR | N.D | N.D | Q9NXV6 | CARF_HUMAN | **CDK2A-interacting protein** |  |
| 2 | 10 | | Ace | AQ | | AQGLIEVER | 100% Ac- | 100.00 | Q9BU02 | THTPA_HUMAN | **Thiamine-triphosphatase** |  |
| 2 | 8 | | Ace | AQ | | AQILPIR | 100% Ac- | 100.00 | Q00610 | CLH1_HUMAN | **Clathrin heavy chain 1** |  |
| 2 | 15 | | Ace | AQ | | AQLGKLLKEQKYDR | 100% Ac- | 100.00 | Q13564 | ULA1_HUMAN | **EDD8-activating enzyme E1 regulatory subunit** |  |
| 2 | 11 | | Ace | AQ | | AQNLKDLAGR | 100% Ac- | 100.00 | Q99623 | PHB2_HUMAN | **Prohibitin-2** |  |
| 2 | 17 | | Ace | AQ | | AQPGPASQPDVSLQQR | 100% Ac- | 100.00 | Q15276 | RABE1_HUMAN | **Rab GTPase-binding effector protein 1** |  |
| 2 | 17 | | Ace | AQ | | AQPGTLNLNNEVVKMR | 100% Ac- | 100.00 | Q8NEF9 | SRFB1_HUMAN | **Serum response factor-binding protein 1** |  |
| 2 | 29 | | Ace | AQ | | AQQQMTSSQKALMLELKSLQEEPVEGFR | 100% Ac- | 100.00 | Q712K3 | UB2R2_HUMAN | **Ubiquitin-conjugating enzyme E2 R2** |  |
| 2 | 24 | | Ace | AQ | | AQTLQMEIPNFGNSILECLNEQR | 100% Ac- | 100.00 | Q96RE7 | BTB14_HUMAN | **BTB/POZ domain-containing protein 14B** |  |
| 2 | 20 | | Ace | AQ | | AQVAMSTLPVEDEESSESR | N.D | N.D | P78347 | GTF2I_HUMAN | **General transcription factor II-I** |  |
| 2 | 13 | | Ace | AQ | | AQWNQLQQLDTR | 100% Ac- | 100.00 | P40763 | STAT3_HUMAN | **Signal transducer and activator of transcription 3** |  |
| 2 | 13 | | Ace | AQ | | AQYKGAASEAGR | 100% Ac- | 100.00 | Q14320 | FA50A_HUMAN | **Protein FAM50A** |  |
| 2 | 14 | | Ace | AQ | | AQAAGPAGGGEPR | 100% Ac- | 100.00 | Q08AE8 | SPIR1_HUMAN | **Protein spire homolog 1** |  |
| 2 | 21 | | Ace | AS | | ASAELQGKYQKLAQEYSKLR | 100% Ac- | 100.00 | Q6ZMI0 | CC128_HUMAN | **Coiled-coil domain-containing protein 128** |  |
| 2 | 12 | | Ace | AS | | ASAGSGMEEVR | N.D | N.D | Q9BQJ4 | TMM47_HUMAN | **Transmembrane protein 47** |  |
| 2 | 10 | | Ace | AS | | ASAGVAAGR | 100% Ac- | 100.00 | Q9NWA0 | MED9_HUMAN | **Mediator of RA polymerase II transcription subunit 9** |  |
| 2 | 13 | | Ace | AS | | ASALEQFVNSVR | 100% Ac- | 100.00 | Q9UNS2 | CSN3_HUMAN | **COP9 signalosome complex subunit 3** |  |
| 2 | 22 | | Ace | AS | | ASATAPAAAVPTLASPLEQLR | 100% Ac- | 100.00 | O95273 | CCDB1_HUMAN | **Cyclin-D1-binding protein 1** |  |
| 2 | 17 | | Ace | AS | | ASAVFEGTSLVNMFVR | N.D | N.D | Q5TGL8 | CF145_HUMAN | **PX domain-containing protein C6orf145** |  |
| 2 | 18 | | Ace | AS | | ASAVSPANLPAVLLQPR | 100% Ac- | 100.00 | P51610 | HCFC1_HUMAN | **Host cell factor** |  |
| 2 | 11 | | Ace | AS | | ASDAVQSEPR | 100% Ac- | 100.00 | Q16512 | PKN1_HUMAN | **Serine/threonine-protein kinase 1** |  |
| 2 | 9 | | Ace | AS | | ASEEASLR | 100% Ac- | 100.00 | Q96QU8 | XPO6_HUMAN | **Exportin-6** |  |
| 2 | 22 | | Ace | AS | | ASEELQKDLEEVKVLLEKATR | 100% Ac- | 100.00 | Q9HB71 | CYBP_HUMAN | **Calcyclin-binding protein** |  |
| 2 | 14 | | Ace | AS | | ASELEPEVQAIDR | 100% Ac- | 100.00 | Q8IWV8 | UBR2_HUMAN | **E3 ubiquitin-protein ligase UBR2** |  |
| 2 | 8 | | Ace | AS | | ASELGAR | 100% Ac- | 100.00 | Q9ULG1 | INO80_HUMAN | **Putative DA helicase IO80 complex homolog 1** |  |
| 2 | 13 | | Ace | AS | | ASESETLNPSAR | 100% Ac- | 100.00 | O75164 | JHD3A_HUMAN | **JmjC domain-containing histone demethylation protein 3A** |  |
| 2 | 17 | | Ace | AS | | ASGAGGVGGGGGGKIR | 100% Ac- | 100.00 | P49790 | NU153_HUMAN | **Nuclear pore complex protein up153** |  |
| 2 | 17 | | Ace | AS | | ASGAYNPYIEIIEQPR | 100% Ac- | 100.00 | Q04864 | REL_HUMAN | **C-Rel proto-oncogene protein** |  |
| 2 | 25 | | Ace | AS | | ASGCKIGPSILNSDLANLGAECLR | 100% Ac- | 100.00 | Q96AT9 | RPE_HUMAN | **Ribulose-phosphate 3-epimerase** |  |
| 2 | 20 | | Ace | AS | | ASGGSGGVSVPALWSEVNR | N.D | N.D | O76094 | SRP72_HUMAN | **Signal recognition particle 72 kDa protein** |  |
| 2 | 11 | | Ace | AS | | ASGQGPGPPR | N.D | N.D | Q13014 | BAK2_HUMAN | **Putative Bcl-2 homologous antagonist/killer 2** | Q16611 (2-11) |
| 2 | 11 | | Ace | AS | | ASGSGDSVTR | 100% Ac- | 100.00 | Q92797 | SYMPK_HUMAN | **Symplekin** |  |
| 2 | 13 | | Ace | AS | | ASGSGTKNLDFR | 100% Ac- | 100.00 | Q96NC0 | ZMAT2_HUMAN | **Zinc finger matrin-type protein 2** |  |
| 2 | 21 | | Ace | AS | | ASGVAVSDGVIKVFNDMKVR | 100% Ac- | 100.00 | P23528 | COF1_HUMAN | **Cofilin-1** |  |
| 2 | 13 | | Ace | AS | | ASGVQVADEVCR | 100% Ac- | 100.00 | P60981 | DEST_HUMAN | **Destrin** |  |
| 2 | 21 | | Ace | AS | | ASGVTVNDEVIKVFNDMKVR | 100% Ac- | 100.00 | Q9Y281 | COF2_HUMAN | **Cofilin-2** |  |
| 2 | 14 | | Ace | AS | | ASILDEYENSLSR | 100% Ac- | 100.00 | Q9P253 | VPS18_HUMAN | **Vacuolar protein sorting-associated protein 18 homolog** |  |
| 2 | 14 | | Ace | AS | | ASKEMFEDTVEER | 100% Ac- | 100.00 | Q16576 | RBBP7_HUMAN | **Histone-binding protein RBBP7** |  |
| 2 | 12 | | Ace | AS | | ASKGAGMSFSR | 100% Ac- | 100.00 | Q5XPI4 | RN123_HUMAN | **E3 ubiquitin-protein ligase RF123** |  |
| 2 | 11 | | Ace | AS | | ASLDDPGEVR | 100% Ac- | 100.00 | Q9H1K0 | RBNS5_HUMAN | **Rabenosyn-5** |  |
| 2 | 8 | | Ace | AS | | ASLEVSR | 100% Ac- | 100.00 | O60762 | DPM1_HUMAN | **Dolichol-phosphate mannosyltransferase** |  |
| 2 | 19 | | Ace | AS | | ASLFKKKTVDDVIKEQNR | 100% Ac- | 100.00 | Q9UQN3 | CHM2B_HUMAN | **Charged multivesicular body protein 2b** |  |
| 2 | 19 | | Ace | AS | | ASLLKVDQEVKLKVDSFR | 100% Ac- | 100.00 | P61289 | PSME3_HUMAN | **Proteasome activator complex subunit 3** |  |
| 2 | 9 | | Ace | AS | | ASLLQSDR | 100% Ac- | 100.00 | Q9UJW0 | DCTN4_HUMAN | **Dynactin subunit 4** |  |
| 2 | 20 | | Ace | AS | | ASLSLAPVNIFKAGADEER | 100% Ac- | 100.00 | P78371 | TCPB_HUMAN | **T-complex protein 1 subunit beta** |  |
| 2 | 18 | | Ace | AS | | ASLTVKAYLLGKEDAAR | 100% Ac- | 100.00 | Q13501 | SQSTM_HUMAN | **Sequestosome-1** |  |
| 2 | 14 | | Ace | AS | | ASMGTLAFDEYGR | N.D | N.D | P48643 | TCPE_HUMAN | **T-complex protein 1 subunit epsilon** |  |
| 2 | 12 | | Ace | AS | | ASMAAAIAASR | 100% Ac- | 100.00 | Q9H425 | CA198_HUMAN | **Uncharacterized protein C1orf198** |  |
| 2 | 16 | | Ace | AS | | ASNFKKANMASSSQR | 100% Ac- | 100.00 | P41208 | CETN2_HUMAN | **Centrin-2** |  |
| 2 | 38 | | Ace | AS | | ASNKTTLQKMGKKQNGKSKKVEEAEPEEFVVEKVLDR | 100% Ac- | 100.00 | Q13185 | CBX3_HUMAN | **Chromobox protein homolog 3** |  |
| 2 | 13 | | Ace | AS | | ASNNTASIAQAR | 100% Ac- | 100.00 | P59768 | GBG2_HUMAN | **Guanine nucleotide-binding protein G(I)/G(S)/G(O) subunit gamma-2** |  |
| 2 | 34 | | Ace | AS | | ASNSTKSFLADAGYGEQELDANSALMELDKGLR | 100% Ac- | 100.00 | Q9NVH2 | INT7_HUMAN | **Integrator complex subunit 7** |  |
| 2 | 12 | | Ace | AS | | ASNVTNKTDPR | 100% Ac- | 100.00 | P07910 | HNRPC_HUMAN | **Heterogeneous nuclear ribonucleoproteins C1/C2** |  |
| 2 | 33 | | Ace | AS | | ASPFSGALQLTDLDDFIGPSQECIKPVKVEKR | 100% Ac- | 100.00 | Q9H6Q4 | NARFL_HUMAN | **Nuclear prelamin A recognition factor-like protein** |  |
| 2 | 10 | | Ace | AS | | ASPGKDNYR | 100% Ac- | 100.00 | O15131 | IMA5_HUMAN | **Importin subunit alpha-6** | O60684 (5-13) |
| 2 | 24 | | Ace | AS | | ASQPNSSAKKKEEKGKNIQVVVR | 100% Ac- | 100.00 | P52732 | KIF11_HUMAN | **Kinesin-like protein KIF11** |  |
| 2 | 17 | | Ace | AS | | ASQSQGIQQLLQAEKR | 100% Ac- | 100.00 | O75348 | VATG1_HUMAN | **V-type proton ATPase subunit G 1** | O95670 (2-17) |
| 2 | 21 | | Ace | AS | | ASSAQSGGSSGGPAVPTVQR | 100% Ac- | 100.00 | Q7KZF4 | SND1_HUMAN | **Staphylococcal nuclease domain-containing protein 1** |  |
| 2 | 14 | | Ace | AS | | ASSDIQVKELEKR | 100% Ac- | 100.00 | P16949 | STMN1_HUMAN | **Stathmin** |  |
| 2 | 8 | | Ace | AS | | ASSEVAR | 100% Ac- | 100.00 | Q9P0V9 | SEP10_HUMAN | **Septin-10** |  |
| 2 | 13 | | Ace | AS | | ASSLNEDPEGSR | 100% Ac- | 100.00 | Q9Y530 | CF130_HUMAN | **Uncharacterized protein C6orf130** |  |
| 2 | 10 | | Ace | AS | | ASSNTVLMR | 100% Ac- | 100.00 | O95861 | BPNT1_HUMAN | **3'(2'),5'-bisphosphate nucleotidase 1** |  |
| 2 | 22 | | Ace | AS | | ASSSGAGAAAAAAAANLNAVR | Partial-Ac- | 87.90 | Q08AG7 | CM037_HUMAN | **UPF0582 protein C13orf37** |  |
| 2 | 17 | | Ace | AS | | ASSVGNVADSTEPTKR | 100% Ac- | 100.00 | O15294 | OGT1_HUMAN | **UDP--acetylglucosamine--peptide -acetylglucosaminyltransferase 110 kDa subunit** |  |
| 2 | 29 | | Ace | AS | | ASTGSQASDIDEIFGFFNDGEPPTKKPR | 100% Ac- | 100.00 | Q9BPY3 | F118B_HUMAN | **Protein FAM118B** |  |
| 2 | 12 | | Ace | AS | | ASTNAESQLQR | 100% Ac- | 100.00 | Q9BQS8 | FYCO1_HUMAN | **FYVE and coiled-coil domain-containing protein 1** |  |
| 2 | 15 | | Ace | AS | | ASVAVDPQPSVVTR | N.D | N.D | Q99541 | ADFP_HUMAN | **Adipophilin** |  |
| 2 | 15 | | Ace | AS | | ASVDFKTYVDQACR | 100% Ac- | 100.00 | Q9UKK6 | NXT1_HUMAN | **TF2-related export protein 1** |  |
| 2 | 12 | | Ace | AS | | ASYYEILDVPR | 100% Ac- | 100.00 | P25686 | DNJB2_HUMAN | **DnaJ homolog subfamily B member 2** |  |
| 2 | 14 | | Ace | AS | | ASAAAGEAEETTR | 100% Ac- | 100.00 | Q86VE0 | P42P0_HUMAN | **Myb-related protein p42POP** |  |
| 2 | 22 | | Ace | AT | | ATALSEEELDNEDYYSLLNVR | N.D | N.D | Q9NVH1 | DJC11_HUMAN | **DnaJ homolog subfamily C member 11** |  |
| 2 | 15 | | Ace | AT | | ATANGAVENGQPDR | 100% Ac- | 100.00 | P47895 | AL1A3_HUMAN | **Aldehyde dehydrogenase family 1 member A3** |  |
| 2 | 9 | | Ace | AT | | ATCAEILR | 100% Ac- | 100.00 | Q9NUQ8 | ABCF3_HUMAN | **ATP-binding cassette sub-family F member 3** |  |
| 2 | 19 | | Ace | AT | | ATDDKTSPTLDSANDLPR | 100% Ac- | 100.00 | Q9Y2I7 | FYV1_HUMAN | **FYVE finger-containing phosphoinositide kinase** |  |
| 2 | 12 | | Ace | AT | | ATDELATKLSR | 100% Ac- | 100.00 | Q96C19 | EFHD2_HUMAN | **EF-hand domain-containing protein D2** |  |
| 2 | 9 | | Ace | AT | | ATEEFIIR | 100% Ac- | 100.00 | Q14764 | MVP_HUMAN | **Major vault protein** |  |
| 2 | 14 | | Ace | AT | | ATEEKKPETEAAR | 100% Ac- | 100.00 | P61964 | WDR5_HUMAN | **WD repeat-containing protein 5** |  |
| 2 | 20 | | Ace | AT | | ATEGGGKEMNEIKTQFTTR | 100% Ac- | 100.00 | Q8TBZ3 | WDR20_HUMAN | **WD repeat-containing protein 20** |  |
| 2 | 21 | | Ace | AT | | ATELEYESVLCVKPDVSVYR | N.D | N.D | Q8NC96 | NECP1_HUMAN | **Adaptin ear-binding coat-associated protein 1** |  |
| 2 | 30 | | Ace | AT | | ATFSGPAGPILSLNPQEDVEFQKEVAQVR | Partial-Ac- | 58.61 | Q9BYG3 | MK67I_HUMAN | **MKI67 FHA domain-interacting nucleolar phosphoprotein** |  |
| 2 | 17 | | Ace | AT | | ATGANATPLDFPSKKR | 100% Ac- | 100.00 | Q15637 | SF01_HUMAN | **Splicing factor 1** |  |
| 2 | 9 | | Ace | AT | | ATGQKLMR | 100% Ac- | 100.00 | Q08257 | QOR_HUMAN | **Quinone oxidoreductase** |  |
| 2 | 11 | | Ace | AT | | ATGTPESQAR | 100% Ac- | 100.00 | Q96B45 | CJ032_HUMAN | **UPF0693 protein C10orf32** |  |
| 2 | 14 | | Ace | AT | | ATIPDWKLQLLAR | 100% Ac- | 100.00 | Q6NYC8 | PHTNS_HUMAN | **Phostensin** |  |
| 2 | 23 | | Ace | AT | | ATKCGNCGPGYSTPLEAMKGPR | Partial-Ac- | 69.46 | Q13228 | SBP1_HUMAN | **Selenium-binding protein 1** |  |
| 2 | 11 | | Ace | AT | | ATKIDKEACR | 100% Ac- | 100.00 | Q14019 | COTL1_HUMAN | **Coactosin-like protein** |  |
| 2 | 17 | | Ace | AT | | ATLIYVDKENGEPGTR | 100% Ac- | 100.00 | O95997 | PTTG1_HUMAN | **Securin** |  |
| 2 | 17 | | Ace | AT | | ATLVVNKLGAGVDSGR | 100% Ac- | 100.00 | P36406 | ARD1_HUMAN | **GTP-binding protein ARD-1** |  |
| 2 | 20 | | AcD3 | AT | | ATNWGSLLQDKQQLEELAR | 100% Ac- | 100.00 | P48637 | GSHB_HUMAN | **Glutathione synthetase** |  |
| 2 | 11 | | Ace | AT | | ATPASAPDTR | N.D | N.D | Q92843 | BCLW_HUMAN | **Apoptosis regulator Bcl-W** |  |
| 2 | 19 | | Ace | AT | | ATPDQKSPNVLLQNLCCR | 100% Ac- | 100.00 | Q96CW5 | GCP3_HUMAN | **Gamma-tubulin complex component 3** |  |
| 2 | 13 | | Ace | AT | | ATPLVAGPAALR | 100% Ac- | 100.00 | Q9BSI4 | TINF2_HUMAN | **TERF1-interacting nuclear factor 2** |  |
| 2 | 18 | | Ace | AT | | ATPSKKTSTPSPQPSKR | 100% Ac- | 100.00 | Q8IY18 | SMC5_HUMAN | **Structural maintenance of chromosomes protein 5** |  |
| 2 | 18 | | Ace | AT | | ATQADLMELDMAMEPDR | 100% Ac- | 100.00 | P35222 | CTNB1_HUMAN | **Catenin beta-1** |  |
| 2 | 20 | | Ace | AT | | ATSGANGPGSATASASNPR | 100% Ac- | 100.00 | Q53ET0 | CRTC2_HUMAN | **CREB-regulated transcription coactivator 2** |  |
| 2 | 16 | | Ace | AT | | ATSLDFKTYVDQACR | 100% Ac- | 100.00 | Q9NPJ8 | NXT2_HUMAN | **TF2-related export protein 2** |  |
| 2 | 12 | | Ace | AT | | ATSLGSNTYNR | 100% Ac- | 100.00 | Q9NW64 | RBM22_HUMAN | **Pre-mRA-splicing factor RBM22** |  |
| 2 | 23 | | Ace | AT | | ATSPQKSPSVPKSPTPKSPPSR | 100% Ac- | 100.00 | Q9NVD7 | PARVA_HUMAN | **Alpha-parvin** |  |
| 2 | 20 | | Ace | AT | | ATSSMSKGCFVFKPNSKKR | 100% Ac- | 100.00 | Q9UBD5 | ORC3_HUMAN | **Origin recognition complex subunit 3** |  |
| 2 | 10 | | Ace | AT | | ATSVLCCLR | 100% Ac- | 100.00 | Q5W111 | CLLD6_HUMAN | **Chronic lymphocytic leukemia deletion region gene 6 protein** |  |
| 2 | 20 | | Ace | AT | | ATTAELFEEPFVADEYIER | N.D | N.D | O00471 | EXOC5_HUMAN | **Exocyst complex component 5** |  |
| 2 | 14 | | Ace | AT | | ATTATMATSGSAR | 100% Ac- | 100.00 | P38919 | IF4A3_HUMAN | **Eukaryotic initiation factor 4A-III** |  |
| 2 | 12 | | Ace | AT | | ATVMAATAAER | 100% Ac- | 100.00 | Q9GZN7 | ROGDI_HUMAN | **Protein rogdi homolog** |  |
| 2 | 10 | | Ace | AT | | ATVQQLEGR | 100% Ac- | 100.00 | Q01469 | FABP5_HUMAN | **Fatty acid-binding protein, epidermal** |  |
| 2 | 14 | | Ace | AT | | ATVTATTKVPEIR | 100% Ac- | 100.00 | Q9Y230 | RUVB2_HUMAN | **RuvB-like 2** |  |
| 2 | 15 | | Ace | AT | | ATVVLGGDTMGPER | Partial-Ac- | 86.00 | Q16626 | MEA1_HUMAN | **Male-enhanced antigen 1** |  |
| 2 | 14 | | Ace | AT | | ATYLEFIQQNEER | 100% Ac- | 100.00 | Q15437 | SC23B_HUMAN | **Protein transport protein Sec23B** |  |
| 2 | 10 | | Ace | AT | | ATYSLANER | 100% Ac- | 100.00 | Q9P086 | MED11_HUMAN | **Mediator of RA polymerase II transcription subunit 11** |  |
| 2 | 10 | | Ace | AT | | ATYTCITCR | 100% Ac- | 100.00 | Q969S3 | ZN622_HUMAN | **Zinc finger protein 622** |  |
| 2 | 33 | | Ace | AT | | ATAAETSASEPEAESKAGPKADGEEDEVKAAR | 100% Ac- | 100.00 | Q15020 | SART3_HUMAN | **Squamous cell carcinoma antigen recognized by T-cells 3** |  |
| 2 | 12 | | Ace | AT | | ATAATSPALKR | 100% Ac- | 100.00 | Q8WTW3 | COG1_HUMAN | **Conserved oligomeric Golgi complex subunit 1** |  |
| 2 | 12 | | Ace | AV | | AVAELYTQYNR | N.D | N.D | Q9NQX4 | MYO5C_HUMAN | **Myosin-Vc** |  |
| 2 | 26 | | Ace | AV | | AVANSSPVNPVVFFDVSIGGQEVGR | 100% Ac- | 100.00 | O43447 | PPIH_HUMAN | **Peptidyl-prolyl cis-trans isomerase H** |  |
| 2 | 10 | | Ace | AV | | AVASDFYLR | 100% Ac- | 100.00 | Q96A72 | MGN2_HUMAN | **Protein mago nashi homolog 2** |  |
| 2 | 9 | | Ace | AV | | AVEDEGLR | 100% Ac- | 100.00 | Q14644 | RASA3_HUMAN | **Ras GTPase-activating protein 3** |  |
| 2 | 13 | | Ace | AV | | AVEDSTLQVVVR | N.D | N.D | Q86Y91 | KI18B_HUMAN | **Kinesin-like protein KIF18B** |  |
| 2 | 12 | | Ace | AV | | AVEELQSIIKR | 100% Ac- | 100.00 | Q92990 | GLMN_HUMAN | **Glomulin** |  |
| 2 | 11 | | Ace | AV | | AVEGSTITSR | N.D | N.D | O60336 | MABP1_HUMAN | **Mitogen-activated protein kinase-binding protein 1** |  |
| 2 | 10 | | Ace | AV | | AVFADLDLR | 100% Ac- | 100.00 | P78346 | RPP30_HUMAN | **Ribonuclease P protein subunit p30** |  |
| 2 | 8 | | AcD3 | AV | | AVGKNKR | 100% free | 0.00 | P61247 | RS3A_HUMAN | **40S ribosomal protein S3a** |  |
| 2 | 10 | | Ace | AV | | AVKVQTTKR | 100% Ac- | 100.00 | O75746 | CMC1_HUMAN | **Calcium-binding mitochondrial carrier protein Aralar1** |  |
| 2 | 25 | | AcD3 | AV | | AVLKLTDQPPLVQAIFSGDPEEIR | N.D | N.D | Q8N8A2 | ANR44_HUMAN | **Serine/threonine-protein phosphatase 6 regulatory ankyrin repeat subunit B** |  |
| 2 | 17 | | Ace | AV | | AVNVYSTSVTSDNLSR | 100% Ac- | 100.00 | Q15691 | MARE1_HUMAN | **Microtubule-associated protein RP/EB family member 1** |  |
| 2 | 12 | | Ace | AV | | AVPGCNKDSVR | 100% Ac- | 100.00 | Q8N9Q2 | S12IP_HUMAN | **Protein SFRS12IP1** |  |
| 2 | 15 | | Ace | AV | | AVPPTYADLGKSAR | 100% Ac- | 100.00 | P21796 | VDAC1_HUMAN | **Voltage-dependent anion-selective channel protein 1** |  |
| 2 | 33 | | Ace | AV | | AVPAAAMGPSALGQSGPGSMAPWCSVSSGPSR | 100% Ac- | 100.00 | Q96J01 | THOC3_HUMAN | **THO complex subunit 3** |  |
| 2 | 10 | | Ace | AV | | AVPAALILR | 100% Ac- | 100.00 | Q9Y6G5 | COMDA_HUMAN | **COMM domain-containing protein 10** |  |
| 2 | 9 | | Ace | AV | | AVQISKKR | 100% Ac- | 100.00 | P23396 | RS3_HUMAN | **40S ribosomal protein S3** |  |
| 2 | 21 | | Ace | AV | | AVQPKETLQLESAAEVGFVR | 100% Ac- | 100.00 | P43246 | MSH2_HUMAN | **DNA mismatch repair protein Msh2** |  |
| 2 | 21 | | Ace | AV | | AVSDALLPSFSTFASGPAGR | N.D | N.D | O43474 | KLF4_HUMAN | **Krueppel-like factor 4** |  |
| 2 | 18 | | Ace | AV | | AVSESQLKKMVSKYKYR | 100% Ac- | 100.00 | Q99816 | TS101_HUMAN | **Tumor susceptibility gene 101 protein** |  |
| 2 | 11 | | Ace | AV | | AVSTGVKVPR | 100% Ac- | 100.00 | Q15819 | UB2V2_HUMAN | **Ubiquitin-conjugating enzyme E2 variant 2** |  |
| 2 | 9 | | Ace | AV | | AVTAQAAR | 100% Ac- | 100.00 | Q92871 | PMM1_HUMAN | **Phosphomannomutase 1** |  |
| 2 | 10 | | Ace | AV | | AVTEASLLR | 100% Ac- | 100.00 | Q9NW38 | FANCL_HUMAN | **E3 ubiquitin-protein ligase FACL** |  |
| 2 | 11 | | Ace | AV | | AVTGWLESLR | 100% Ac- | 100.00 | Q9BVM2 | DPCD_HUMAN | **Protein DPCD** |  |
| 2 | 12 | | Ace | AV | | AVTLDKDAYYR | 100% Ac- | 100.00 | Q9Y5B9 | SP16H_HUMAN | **FACT complex subunit SPT16** |  |
| 2 | 24 | | Ace | AV | | AVVPASLSGQDVGSFAYLTIKDR | 100% Ac- | 100.00 | Q9H993 | CF211_HUMAN | **UPF0364 protein C6orf211** |  |
| 2 | 19 | | Ace | AV | | AVAAAAAAAGPAGAGGGR | 100% Ac- | 100.00 | Q13884 | SNTB1_HUMAN | **Beta-1-syntrophin** |  |
| **2. Cys-** |  | |  |  | |  |  |  |  |  |  |  |
| 2 | 21 | | Ace | CD | | CDFTEDQTAEFKEAFQLFDR | 100% Ac- | 100.00 | P60660 | MYL6_HUMAN | **Myosin light polypeptide 6** |  |
| 2 | 30 | | Ace | CD | | CDKEFMWALKNGDLDEVKDYVAKGEDVNR | 100% Ac- | 100.00 | P58546 | MTPN_HUMAN | **Myotrophin** |  |
| 2 | 13 | | AcD3 | CG | | CGDCVEKEYPNR | 100% free | 0.00 | Q8WUH1 | CHUR_HUMAN | **Protein Churchill** |  |
| 2 | 12 | | Ace | CS | | CSLASGATGGR | 100% Ac- | 100.00 | O60678 | ANM3_HUMAN | **Protein arginine -methyltransferase 3** |  |
| **3. Asp &Glu-** | | |  |  | |  |  |  |  |  |  |  |
| 2 | | 28 | Ace | DD | | DDDIAALVVDNGSGMCKAGFAGDDAPR | 100% Ac- | 100.00 | P60709 | ACTB_HUMAN | **Actin, cytoplasmic 1** |  |
| 2 | | 28 | Ace | EE | | EEEIAALVIDNGSGMCKAGFAGDDAPR | 100% Ac- | 100.00 | P63261 | ACTG_HUMAN | **Actin, cytoplasmic 2** |  |
| 2 | | 8 | AcD3 | EP | | EPLLLGR | N.D | N.D | O00533 | CHL1_HUMAN | **Neural cell adhesion molecule L1-like protein** |  |
| **4. Gly-** | |  |  |  | |  |  |  |  |  |  |  |
| 2 | | 13 | AcD3 | GK | | GKDYYQTLGLAR | 100% free | 0.00 | P25685 | DNJB1_HUMAN | **DnaJ homolog subfamily B member 1** |  |
| 2 | | 10 | AcD3 | GK | | GKGDPKKPR | 100% free | 0.00 | B2RPK0 | HMGL1_HUMAN | **Putative high mobility group protein 1-like 1** | P09429 (2-10) |
| 2 | | 10 | AcD3 | GK | | GKGDPNKPR | 100% free | 0.00 | P26583 | HMGB2_HUMAN | **High mobility group protein B2** |  |
| 2 | | 12 | AcD3 | GK | | GKKGKKEKKGR | 100% free | 0.00 | Q8TBB5 | KLDC4_HUMAN | **Kelch domain-containing protein 4** |  |
| 2 | | 11 | AcD3 | GK | | GKKGKVGKSR | 100% free | 0.00 | Q8IY81 | RRMJ3_HUMAN | **Putative rRA methyltransferase 3** |  |
| 2 | | 13 | AcD3 | GK | | GKVKVGVNGFGR | 100% free | 0.00 | P04406 | G3P_HUMAN | **Glyceraldehyde-3-phosphate dehydrogenase** |  |
| 2 | | 11 | AcD3 | GP | | GPAPAGEQLR | 100% free | 0.00 | Q9H6R4 | NOL6_HUMAN | **Nucleolar protein 6** |  |
| 2 | | 20 | AcD3 | GP | | GPPGPALPATMNNSSSETR | 100% free | 0.00 | Q15003 | CND2_HUMAN | **Condensin complex subunit 2** |  |
| 2 | | 19 | AcD3 | GQ | | GQNDLMGTAEDFADQFLR | 100% free | 0.00 | O15260 | SURF4_HUMAN | **Surfeit locus protein 4** |  |
| 2 | | 14 | AcD3 | GV | | GVQVETISPGDGR | 100% free | 0.00 | P62942 | FKB1A_HUMAN | **Peptidyl-prolyl cis-trans isomerase FKBP1A** |  |
| **5. Met-Ala-** | | |  |  | |  |  |  |  |  |  |  |
| 1 | | 26 | Ace | MA | | MADDLDFETGDAGASATFPMQCSALR | 100% Ac- | 100.00 | P63241 | IF5A1_HUMAN | **Eukaryotic translation initiation factor 5A-1** | Q6IS14 (1-26) |
| 1 | | 10 | Ace | MA | | MADEELEALR | 100% Ac- | 100.00 | O14737 | PDCD5_HUMAN | **Programmed cell death protein 5** |  |
| 1 | | 12 | AcD3 | MA | | MANALASATCER | 100% free | 0.00 | P48059 | LIMS1_HUMAN | **LIM and senescent cell antigen-like-containing domain protein 1** | Q9HB10 (63-74) |
| 1 | | 17 | Ace | MA | | MAPAEILNGKEISAQIR | Partial-Ac- | 80.91 | P11586 | C1TC_HUMAN | **C-1-tetrahydrofolate synthase, cytoplasmic** |  |
| 1 | | 14 | Ace | MA | | MAPAKKGGEKKKGR | 100% Ac- | 100.00 | P62899 | RL31_HUMAN | **60S ribosomal protein L31** |  |
| 1 | | 22 | Ace | MA | | MAPDSDPFPEGPLLKLLPLDAR | N.D | N.D | Q9BVQ7 | SPA5L_HUMAN | **Spermatogenesis-associated protein 5-like protein 1** |  |
| 1 | | 18 | Ace | MA | | MAPEENAGTELLLQSFER | Partial-Ac- | 81.00 | A6NL58 | F86A2_HUMAN | **Putative protein FAM86A-like 2** | Q96G04 (1-18) |
| 1 | | 11 | Ace | MA | | MAPGEVTITVR | 100% Ac- | 100.00 | Q3KRA6 | CB076_HUMAN | **UPF0538 protein C2orf76** |  |
| 1 | | 22 | AcD3 | MA | | MAPGQLALFSVSDKTGLVEFAR | Partial-Ac- | 48.17 | P31939 | PUR9_HUMAN | **Bifunctional purine biosynthesis protein PURH** |  |
| 1 | | 10 | Ace | MA | | MAPKGKVGTR | 100% Ac- | 100.00 | Q9BTX3 | TM208_HUMAN | **Transmembrane protein 208** |  |
| 1 | | 14 | Ace | MA | | MAPLDLDKYVEIAR | Partial-Ac- | 55.59 | O00743 | PPP6_HUMAN | **Serine/threonine-protein phosphatase 6 catalytic subunit** |  |
| 1 | | 9 | Ace | MA | | MAPLGGAPR | 100% Ac- | 100.00 | Q15126 | PMVK_HUMAN | **Phosphomevalonate kinase** |  |
| 1 | | 9 | Ace | MA | | MAPLLEYER | Partial-Ac- | 88.26 | Q92889 | XPF_HUMAN | **DNA repair endonuclease XPF** |  |
| 1 | | 11 | Ace | MA | | MAPSTPLLTVR | 100% Ac- | 100.00 | Q9BY44 | EIF2A_HUMAN | **Eukaryotic translation initiation factor 2A** |  |
| 1 | | 17 | Ace | MA | | MAPSVPAAEPEYPKGIR | 100% Ac- | 100.00 | P54819 | KAD2_HUMAN | **Adenylate kinase isoenzyme 2, mitochondrial** |  |
| 1 | | 13 | Ace | MA | | MAQDQGEKENPMR | 100% Ac- | 100.00 | P62913 | RL11_HUMAN | **60S ribosomal protein L11** |  |
| **6. Met-Cys-** | | |  |  | |  |  |  |  |  |  |  |
| 1 | | 29 | Ace | MC | | MCEEETTALVCDNGSGLCKAGFAGDDAPR | 100% Ac- | 100.00 | P63267 | ACTH_HUMAN | **Actin, gamma-enteric smooth muscle** |  |
| **7. Met-Asp & Met Glu-** | | | | | |  |  |  |  |  |  |  |
| **1** | | 9 | Ace | | MD | MDAELAEVR | N.D | N.D | Q6IPU0 | CENPP_HUMAN | **Centromere protein P** |  |
| **1** | | 12 | Ace | | MD | MDAFKGGMSLER | 100% Ac- | 100.00 | P78337 | PITX1_HUMAN | **Pituitary homeobox 1** |  |
| **1** | | 7 | Ace | | MD | MDAGFFR | 100% Ac- | 100.00 | Q8IYB3 | SRRM1_HUMAN | **Serine/arginine repetitive matrix protein 1** |  |
| **1** | | 17 | Ace | | MD | MDAGVTESGLNVTLTIR | Partial-Ac- | 88.54 | Q15365 | PCBP1_HUMAN | **Poly(rC)-binding protein 1** |  |
| **1** | | 21 | Ace | | MD | MDAIKKKMQMLKLDKENAIDR | Partial-Ac- | 94.30 | P07951 | TPM2_HUMAN | **Tropomyosin beta chain** |  |
| **1** | | 8 | Ace | | MD | MDAILNYR | 100% Ac- | 100.00 | Q9UKB3 | DJC12_HUMAN | **DnaJ homolog subfamily C member 12** |  |
| **1** | | 9 | Ace | | MD | MDALKSAGR | 100% Ac- | 100.00 | Q5SW96 | ARH_HUMAN | **Low density lipoprotein receptor adapter protein 1** |  |
| **1** | | 6 | Ace | | MD | MDALNR | 100% Ac- | 100.00 | Q86UU1 | PHLB1_HUMAN | **Pleckstrin homology-like domain family B member 1** |  |
| **1** | | 12 | Ace | | MD | MDAQCSAKVNAR | 100% Ac- | 100.00 | P49585 | PCY1A_HUMAN | **Choline-phosphate cytidylyltransferase A** |  |
| **1** | | 32 | Ace | | MD | MDASLEKIADPTLAEMGKNLKEAVKMLEDSQR | 100% Ac- | 100.00 | Q6P474 | PDXD2_HUMAN | **Pyridoxal-dependent decarboxylase domain-containing protein 2** | Q6P996 (1-32) |
| **1** | | 8 | Ace | | MD | MDATALER | 100% Ac- | 100.00 | Q9Y6W3 | CAN7_HUMAN | **Calpain-7** |  |
| **1** | | 23 | Ace | | MD | MDAVNAFNQELFSLMDMKPPISR | 100% Ac- | 100.00 | O95104 | SFR15_HUMAN | **Splicing factor, arginine/serine-rich 15** |  |
| **1** | | 12 | Ace | | MD | MDCEVNNGSSLR | N.D | N.D | P55957 | BID_HUMAN | **BH3-interacting domain death agonist** |  |
| **1** | | 28 | Ace | | MD | MDDDIAALVVDNGSGMCKAGFAGDDAPR | 100% Ac- | 100.00 | P60709 | ACTB_HUMAN | **Actin, cytoplasmic 1** |  |
| **1** | | 9 | Ace | | MD | MDDEEETYR | 100% Ac- | 100.00 | P19388 | RPAB1_HUMAN | **DNA-directed RA polymerases I, II, and III subunit RPABC1** |  |
| **1** | | 6 | Ace | | MD | MDDFER | 100% Ac- | 100.00 | Q05682 | CALD1_HUMAN | **Caldesmon** |  |
| **1** | | 9 | Ace | | MD | MDDIFTQCR | 100% Ac- | 100.00 | Q13418 | ILK_HUMAN | **Integrin-linked protein kinase** |  |
| **1** | | 28 | Ace | | MD | MDDKELIEYFKSQMKEDPDMASAVAAIR | 100% Ac- | 100.00 | Q14232 | EI2BA_HUMAN | **Translation initiation factor eIF-2B subunit alpha** |  |
| **1** | | 24 | Ace | | MD | MDDKGDPSNEEAPKAIKPTSKEFR | 100% Ac- | 100.00 | Q9BTC0 | DIDO1_HUMAN | **Death-inducer obliterator 1** |  |
| **1** | | 8 | Ace | | MD | MDDKKKKR | 100% Ac- | 100.00 | Q9BUN5 | CC28B_HUMAN | **Coiled-coil domain-containing protein 28B** |  |
| **1** | | 16 | Ace | | MD | MDDKPNPEALSDSSER | 100% Ac- | 100.00 | Q3LIE5 | CQ048_HUMAN | **Uncharacterized protein C17orf48** |  |
| **1** | | 28 | Ace | | MD | MDDSEVESTASILASVKEQEAQFEKLTR | 100% Ac- | 100.00 | O60716 | CTND1_HUMAN | **Catenin delta-1** |  |
| **1** | | 20 | Ace | | MD | MDDVPAPTPAPAPPAAAAPR | 100% Ac- | 100.00 | Q96SL8 | FIZ1_HUMAN | **Flt3-interacting zinc finger protein 1** |  |
| **1** | | 25 | Ace | | MD | MDEDGLPLMGSGIDLTKVPAIQQKR | 100% Ac- | 100.00 | Q9Y3C0 | CCD53_HUMAN | **Coiled-coil domain-containing protein 53** |  |
| **1** | | 14 | Ace | | MD | MDEESLESALQTYR | 100% Ac- | 100.00 | Q8N5A5 | ZGPAT_HUMAN | **Zinc finger CCCH-type with G patch domain-containing protein** |  |
| **1** | | 9 | Ace | | MD | MDEGPVDLR | 100% Ac- | 100.00 | P20749 | BCL3_HUMAN | **B-cell lymphoma 3-encoded protein** |  |
| **1** | | 19 | Ace | | MD | MDELAGGGGGGPGMAAPPR | 100% Ac- | 100.00 | Q13033 | STRN3_HUMAN | **Striatin-3** |  |
| **1** | | 23 | Ace | | MD | MDELQDVQLTEIKPLLNDKNGTR | Partial-Ac- | 85.43 | Q9UGV2 | NDRG3_HUMAN | **Protein DRG3** |  |
| **1** | | 18 | Ace | | MD | MDEQALLGLNPNADSDFR | 100% Ac- | 100.00 | O43592 | XPOT_HUMAN | **Exportin-T** |  |
| **1** | | 18 | Ace | | MD | MDESALLDLLECPVCLER | 100% Ac- | 100.00 | Q7Z6J0 | SH3R1_HUMAN | **Putative E3 ubiquitin-protein ligase SH3RF1** |  |
| **1** | | 12 | Ace | | MD | MDETSPLVSPER | 100% Ac- | 100.00 | Q9BTU6 | P4K2A_HUMAN | **Phosphatidylinositol 4-kinase type 2-alpha** |  |
| **1** | | 11 | Ace | | MD | MDETVAEFIKR | 100% Ac- | 100.00 | Q96H22 | CENPN_HUMAN | **Centromere protein** |  |
| **1** | | 16 | Ace | | MD | MDFLLGNPFSSPVGQR | 100% Ac- | 100.00 | O60784 | TOM1_HUMAN | **Target of Myb protein 1** |  |
| **1** | | 18 | Ace | | MD | MDFNMKKLASDAGIFFTR | 100% Ac- | 100.00 | Q9NR46 | SHLB2_HUMAN | **Endophilin-B2** |  |
| **1** | | 15 | Ace | | MD | MDGASAEQDGLQEDR | 100% Ac- | 100.00 | Q08378 | GOGA3_HUMAN | **Golgin subfamily A member 3** |  |
| **1** | | 16 | Ace | | MD | MDGAVMEGPLFLQSQR | 100% Ac- | 100.00 | Q99704 | DOK1_HUMAN | **Docking protein 1** |  |
| **1** | | 33 | AcD3 | | MD | MDGEEKTYGGCEGPDAMYVKLISSDGHEFIVKR | 100% free | 0.00 | Q15369 | ELOC_HUMAN | **Transcription elongation factor B polypeptide 1** |  |
| **1** | | 31 | Ace | | MD | MDGETAEEQGGPVPPPVAPGGPGLGGAPGGR | 100% Ac- | 100.00 | Q16644 | MAPK3_HUMAN | **MAP kinase-activated protein kinase 3** |  |
| **1** | | 23 | Ace | | MD | MDGFAGSLDDSISAASTSDVQDR | 100% Ac- | 100.00 | Q9HC35 | EMAL4_HUMAN | **Echinoderm microtubule-associated protein-like 4** |  |
| **1** | | 14 | Ace | | MD | MDGGDDGNLIIKKR | 100% Ac- | 100.00 | Q9GZU8 | NIP30_HUMAN | **EFA-interacting nuclear protein IP30** |  |
| **1** | | 14 | Ace | | MD | MDGIVPDIAVGTKR | 100% Ac- | 100.00 | P26599 | PTBP1_HUMAN | **Polypyrimidine tract-binding protein 1** |  |
| **1** | | 14 | Ace | | MD | MDGIVTEVAVGVKR | 100% Ac- | 100.00 | Q9UKA9 | PTBP2_HUMAN | **Polypyrimidine tract-binding protein 2** |  |
| **1** | | 17 | Ace | | MD | MDGKQGGMDGSKPAGPR | 100% Ac- | 100.00 | Q96DX5 | ASB9_HUMAN | **Ankyrin repeat and SOCS box protein 9** |  |
| **1** | | 8 | Ace | | MD | MDGLLNPR | 100% Ac- | 100.00 | Q5T6V5 | CI064_HUMAN | **UPF0553 protein C9orf64** |  |
| **1** | | 12 | Ace | | MD | MDGLQASAGPLR | 100% Ac- | 100.00 | Q9H6W3 | NO66_HUMAN | **Nucleolar protein 66** |  |
| **1** | | 16 | Ace | | MD | MDGTEGSAGQPGPAER | 100% Ac- | 100.00 | Q9BZ67 | FRMD8_HUMAN | **FERM domain-containing protein 8** |  |
| **1** | | 15 | Ace | | MD | MDGVVTDLITVGLKR | 100% Ac- | 100.00 | O95758 | ROD1_HUMAN | **Regulator of differentiation 1** |  |
| **1** | | 13 | Ace | | MD | MDGAAGPGDGPAR | 100% Ac- | 100.00 | Q32P44 | EMAL3_HUMAN | **Echinoderm microtubule-associated protein-like 3** |  |
| **1** | | 9 | Ace | | MD | MDIEAYLER | 100% Ac- | 100.00 | P18440 | ARY1_HUMAN | **Arylamine -acetyltransferase 1** |  |
| **1** | | 7 | Ace | | MD | MDIIFGR | 100% Ac- | 100.00 | Q9HAY6 | BCDO1_HUMAN | **Beta,beta-carotene 15,15'-monooxygenase** |  |
| **1** | | 10 | Ace | | MD | MDILKSEILR | 100% Ac- | 100.00 | Q99633 | PRP18_HUMAN | **Pre-mRA-splicing factor 18** |  |
| **1** | | 9 | Ace | | MD | MDKDCEMKR | 100% Ac- | 100.00 | P16455 | MGMT_HUMAN | **Methylated-DA--protein-cysteine methyltransferase** |  |
| **1** | | 21 | Ace | | MD | MDKDSQGLLDSSLMASGTASR | N.D | N.D | Q96EZ8 | MCRS1_HUMAN | **Microspherule protein 1** |  |
| **1** | | 18 | Ace | | MD | MDKNELVQKAKLAEQAER | 100% Ac- | 100.00 | P63104 | 1433Z_HUMAN | **14-3-3 protein zeta/delta** |  |
| **1** | | 17 | Ace | | MD | MDKNIGEQLNKAYEAFR | 100% Ac- | 100.00 | Q92844 | TANK_HUMAN | **TRAF family member-associated F-kappa-B activator** |  |
| **1** | | 12 | Ace | | MD | MDKVCAVFGGSR | 100% Ac- | 100.00 | Q8N4T8 | CBR4_HUMAN | **Carbonyl reductase 4** |  |
| **1** | | 11 | Ace | | MD | MDLDLLDLNPR | N.D | N.D | O43542 | XRCC3_HUMAN | **DNA repair protein XRCC3** |  |
| **1** | | 12 | Ace | | MD | MDLEEAEEFKER | 100% Ac- | 100.00 | Q53T94 | TAF1B_HUMAN | **TATA box-binding protein-associated factor RA polymerase I subunit B** |  |
| **1** | | 12 | Ace | | MD | MDLFGDLPEPER | 100% Ac- | 100.00 | Q9H0C8 | ILKAP_HUMAN | **Integrin-linked kinase-associated serine/threonine phosphatase 2C** |  |
| **1** | | 7 | Ace | | MD | MDLILNR | 100% Ac- | 100.00 | Q8IWZ6 | BBS7_HUMAN | **Bardet-Biedl syndrome 7 protein** |  |
| **1** | | 12 | Ace | | MD | MDLKTAVFNAAR | 100% Ac- | 100.00 | Q96JP0 | FEM1C_HUMAN | **Protein fem-1 homolog C** |  |
| **1** | | 9 | Ace | | MD | MDLPDSASR | 100% Ac- | 100.00 | Q9BQD3 | CS050_HUMAN | **UPF0459 protein C19orf50** |  |
| **1** | | 18 | Ace | | MD | MDLQAAGAQAQGAAEPSR | 100% Ac- | 100.00 | Q9NQ92 | CQ079_HUMAN | **Uncharacterized protein C17orf79** |  |
| **1** | | 8 | Ace | | MD | MDLSELER | 100% Ac- | 100.00 | O75792 | RNH2A_HUMAN | **Ribonuclease H2 subunit A** |  |
| **1** | | 24 | Ace | | MD | MDLSGVKKKSLLGVKENNKKSSTR | 100% Ac- | 100.00 | Q15287 | RNPS1_HUMAN | **RNA-binding protein with serine-rich domain 1** |  |
| **1** | | 10 | Ace | | MD | MDNFFTEGTR | 100% Ac- | 100.00 | Q9HD67 | MYO10_HUMAN | **Myosin-X** |  |
| **1** | | 12 | Ace | | MD | MDNLSSEEIQQR | 100% Ac- | 100.00 | O00161 | SNP23_HUMAN | **Synaptosomal-associated protein 23** |  |
| **1** | | 10 | Ace | | MD | MDNQCTVQVR | 100% Ac- | 100.00 | Q03111 | ENL_HUMAN | **Protein ENL** |  |
| **1** | | 19 | Ace | | MD | MDNSGKEAEAMALLAEAER | 100% Ac- | 100.00 | P54920 | SNAA_HUMAN | **Alpha-soluble SF attachment protein** |  |
| **1** | | 17 | Ace | | MD | MDNYADLSDTELTTLLR | 100% Ac- | 100.00 | P50402 | EMD_HUMAN | **Emerin** |  |
| **2** | | 12 | Ace | | MD | MDPCSVGVQLR | 100% Ac- | 100.00 | Q8N8R7 | CK046_HUMAN | **Uncharacterized protein C11orf46** |  |
| **1** | | 20 | Ace | | MD | MDPECAQLLPALCAVLVDPR | 100% Ac- | 100.00 | Q6PJG6 | CG027_HUMAN | **HEAT repeat-containing protein C7orf27** |  |
| **1** | | 20 | Ace | | MD | MDPEDEGVAGVMSVGPPAAR | N.D | N.D | P17098 | ZNF8_HUMAN | **Zinc finger protein 8** |  |
| **1** | | 11 | Ace | | MD | MDPFTEKLLER | 100% Ac- | 100.00 | Q9NQW6 | ANLN_HUMAN | **Actin-binding protein anillin** |  |
| **1** | | 19 | Ace | | MD | MDPGKDKEGVPQPSGPPAR | 100% Ac- | 100.00 | P07992 | ERCC1_HUMAN | **DNA excision repair protein ERCC-1** |  |
| **1** | | 9 | Ace | | MD | MDPGAALQR | N.D | N.D | Q6IQ22 | RAB12_HUMAN | **Putative Ras-related protein Rab-12** |  |
| **1** | | 11 | Ace | | MD | MDPNTIIEALR | 100% Ac- | 100.00 | O95373 | IPO7_HUMAN | **Importin-7** |  |
| **1** | | 18 | Ace | | MD | MDPSGVKVLETAEDIQER | 100% Ac- | 100.00 | Q13813 | SPTA2_HUMAN | **Spectrin alpha chain, brain** |  |
| **1** | | 7 | Ace | | MD | MDPSLLR | 100% Ac- | 100.00 | P29084 | T2EB_HUMAN | **Transcription initiation factor IIE subunit beta** |  |
| **1** | | 23 | Ace | | MD | MDPTAGSKKEPGGGAATEEGVNR | 100% Ac- | 100.00 | Q96GX5 | MASTL_HUMAN | **Microtubule-associated serine/threonine-protein kinase-like** |  |
| **1** | | 14 | Ace | | MD | MDPVVLSYMDSLLR | N.D | N.D | Q96LD8 | SENP8_HUMAN | **Sentrin-specific protease 8** |  |
| **1** | | 9 | Ace | | MD | MDQCVTVER | 100% Ac- | 100.00 | Q9H871 | RMD5A_HUMAN | **Protein RMD5 homolog A** |  |
| **1** | | 11 | Ace | | MD | MDQEPVGGVER | 100% Ac- | 100.00 | Q8N128 | F177A_HUMAN | **Protein FAM177A1** |  |
| **1** | | 12 | Ace | | MD | MDQVMQFVEPSR | 100% Ac- | 100.00 | P60059 | SC61G_HUMAN | **Protein transport protein Sec61 subunit gamma** |  |
| **1** | | 34 | Ace | | MD | MDSAGQDINLNSPNKGLLSDSMTDVPVDTGVAAR | 100% Ac- | 100.00 | O43399 | TPD54_HUMAN | **Tumor protein D54** |  |
| **1** | | 10 | Ace | | MD | MDSEAFQSAR | 100% Ac- | 100.00 | Q8WUF5 | IASPP_HUMAN | **RelA-associated inhibitor** |  |
| **1** | | 31 | Ace | | MD | MDSKQQCVKLNDGHFMPVLGFGTYAPPEVPR | 100% Ac- | 100.00 | P42330 | AK1C3_HUMAN | **Aldo-keto reductase family 1 member C3** |  |
| **1** | | 8 | Ace | | MD | MDSLAESR | 100% Ac- | 100.00 | Q9NS91 | RAD18_HUMAN | **E3 ubiquitin-protein ligase RAD18** |  |
| **1** | | 15 | Ace | | MD | MDSLEEPQKKVFKAR | 100% Ac- | 100.00 | Q6VMQ6 | MCAF1_HUMAN | **Activating transcription factor 7-interacting protein 1** |  |
| **1** | | 10 | Ace | | MD | MDSQKEALQR | N.D | N.D | Q9BXM9 | FSD1L_HUMAN | **FSD1-like protein** |  |
| **1** | | 16 | Ace | | MD | MDSSAVITQISKEEAR | N.D | N.D | Q9GZU7 | CTDS1_HUMAN | **Carboxy-terminal domain RA polymerase II polypeptide A small phosphatase 1** |  |
| **1** | | 11 | Ace | | MD | MDSTLTASEIR | 100% Ac- | 100.00 | P49588 | SYAC_HUMAN | **Alanyl-tRA synthetase, cytoplasmic** |  |
| **1** | | 16 | Ace | | MD | MDSVEKGAATSVSNPR | 100% Ac- | 100.00 | Q8NFW8 | NEUA_HUMAN | **N-acylneuraminate cytidylyltransferase** |  |
| **1** | | 10 | Ace | | MD | MDSVEKTTNR | 100% Ac- | 100.00 | Q9NWS6 | F118A_HUMAN | **Protein FAM118A** |  |
| **1** | | 10 | Ace | | MD | MDTAEEDICR | N.D | N.D | O60337 | MARH6_HUMAN | **E3 ubiquitin-protein ligase MARCH6** |  |
| **1** | | 10 | Ace | | MD | MDTKEEKKER | 100% Ac- | 100.00 | Q8NI08 | NCOA7_HUMAN | **Nuclear receptor coactivator 7** |  |
| **1** | | 9 | Ace | | MD | MDTMMLNVR | 100% Ac- | 100.00 | Q9H0H5 | RGAP1_HUMAN | **Rac GTPase-activating protein 1** |  |
| **1** | | 11 | Ace | | MD | MDTSDLFASCR | N.D | N.D | Q969K4 | ABTB1_HUMAN | **Ankyrin repeat and BTB/POZ domain-containing protein 1** |  |
| **1** | | 8 | Ace | | MD | MDVFLMIR | 100% Ac- | 100.00 | Q15370 | ELOB_HUMAN | **Transcription elongation factor B polypeptide 2** |  |
| **1** | | 13 | Ace | | MD | MDVGELLSYQPNR | 100% Ac- | 100.00 | Q8WYA6 | CTBL1_HUMAN | **Beta-catenin-like protein 1** |  |
| **1** | | 11 | Ace | | MD | MDVLVSECSAR | 100% Ac- | 100.00 | P54136 | SYRC_HUMAN | **Arginyl-tRA synthetase, cytoplasmic** |  |
| **1** | | 9 | Ace | | MD | MDVNIAPLR | 100% Ac- | 100.00 | O75915 | PRAF3_HUMAN | **PRA1 family protein 3** |  |
| **1** | | 13 | Ace | | MD | MDYDFKVKLSSER | N.D | N.D | P49336 | CDK8_HUMAN | **Cell division protein kinase 8** |  |
| **1** | | 12 | Ace | | MD | MDAATLTYDTLR | 100% Ac- | 100.00 | Q9Y4P1 | ATG4B_HUMAN | **Cysteine protease ATG4B** |  |
| **1** | | 10 | Ace | | ME | MEADIITNLR | 100% Ac- | 100.00 | Q96EA4 | CCD99_HUMAN | **Coiled-coil domain-containing protein 99** |  |
| **1** | | 8 | Ace | | ME | MEAFLGSR | 100% Ac- | 100.00 | P28070 | PSB4_HUMAN | **Proteasome subunit beta type-4** |  |
| **1** | | 8 | Ace | | ME | MEAFQELR | 100% Ac- | 100.00 | Q96JM3 | ZN828_HUMAN | **Zinc finger protein 828** |  |
| **1** | | 25 | Ace | | ME | MEAGPSGAAAGAYLPPLQQVFQAPR | 100% Ac- | 100.00 | Q9UL18 | I2C1_HUMAN | **Eukaryotic translation initiation factor 2C 1** |  |
| **1** | | 8 | Ace | | ME | MEAGSVVR | 100% Ac- | 100.00 | Q6XZF7 | DNMBP_HUMAN | **Dynamin-binding protein** |  |
| **1** | | 21 | Ace | | ME | MEAIAKYDFKATADDELSFKR | 100% Ac- | 100.00 | P62993 | GRB2_HUMAN | **Growth factor receptor-bound protein 2** |  |
| **1** | | 21 | Ace | | ME | MEAIKKKMQMLKLDKENALDR | 100% Ac- | 100.00 | P06753 | TPM3_HUMAN | **Tropomyosin alpha-3 chain** |  |
| **1** | | 12 | Ace | | ME | MEAKTLGTVTPR | 100% Ac- | 100.00 | Q9BW66 | CINP_HUMAN | **Cyclin-dependent kinase 2-interacting protein** |  |
| **1** | | 11 | Ace | | ME | MEALGDLEGPR | 100% Ac- | 100.00 | Q6NT16 | CF192_HUMAN | **Uncharacterized MFS-type transporter C6orf192** |  |
| **1** | | 21 | Ace | | ME | MEALGKLKQFDAYPKTLEDFR | N.D | N.D | Q9Y282 | ERGI3_HUMAN | **Endoplasmic reticulum-Golgi intermediate compartment protein 3** |  |
| **1** | | 6 | Ace | | ME | MEALSR | 100% Ac- | 100.00 | Q9NPI6 | DCP1A_HUMAN | **mRA-decapping enzyme 1A** |  |
| **1** | | 21 | Ace | | ME | MEANGLGPQGFPELKNDTFLR | 100% Ac- | 100.00 | P06132 | DCUP_HUMAN | **Uroporphyrinogen decarboxylase** |  |
| **1** | | 14 | Ace | | ME | MEAPEGGGGGPAAR | 100% Ac- | 100.00 | Q8N1B3 | FA58A_HUMAN | **Cyclin-related protein FAM58A** |  |
| **1** | | 17 | Ace | | ME | MEAPGLAQAAAAESDSR | N.D | N.D | P51817 | PRKX_HUMAN | **Serine/threonine-protein kinase PRKX** |  |
| **1** | | 11 | Ace | | ME | MEATGVLPFVR | 100% Ac- | 100.00 | Q13045 | FLII_HUMAN | **Protein flightless-1 homolog** |  |
| **1** | | 10 | Ace | | ME | MEATTAGVGR | 100% Ac- | 100.00 | Q8WUD4 | CCD12_HUMAN | **Coiled-coil domain-containing protein 12** |  |
| **1** | | 17 | Ace | | ME | MEAVLTEELDEEEQLLR | N.D | N.D | Q8N6M0 | OTU6B_HUMAN | **OTU domain-containing protein 6B** |  |
| **1** | | 43 | Ace | | ME | MEDAGAAGPGPEPEPEPEPEPEPAPEPEPEPKPGAGTSEAFSR | 100% Ac- | 100.00 | O94885 | SASH1_HUMAN | **SAM and SH3 domain-containing protein 1** |  |
| **1** | | 16 | Ace | | ME | MEDDAPVIYGLEFQAR | 100% Ac- | 100.00 | Q53HC9 | TSSC1_HUMAN | **Protein TSSC1** |  |
| **1** | | 16 | Ace | | ME | MEDEMPKTLYVGNLSR | 100% Ac- | 100.00 | P31483 | TIA1_HUMAN | **Nucleolysin TIA-1 isoform p40** |  |
| **1** | | 7 | Ace | | ME | MEDEVVR | 100% Ac- | 100.00 | P23193 | TCEA1_HUMAN | **Transcription elongation factor A protein 1** |  |
| **1** | | 14 | Ace | | ME | MEDGGLTAFEEDQR | 100% Ac- | 100.00 | Q96RG2 | PASK_HUMAN | **PAS domain-containing serine/threonine-protein kinase** |  |
| **1** | | 23 | Ace | | ME | MEDGVAGPQLGAAAEAAEAAEAR | 100% Ac- | 100.00 | P53805 | RCAN1_HUMAN | **Calcipressin-1** |  |
| **1** | | 14 | Ace | | ME | MEDGVLKEGFLVKR | 100% Ac- | 100.00 | Q9NYT0 | PLEK2_HUMAN | **Pleckstrin-2** |  |
| **1** | | 9 | Ace | | ME | MEDLEEDVR | N.D | N.D | Q6PGN9 | PSRC1_HUMAN | **Proline/serine-rich coiled-coil protein 1** |  |
| **1** | | 15 | Ace | | ME | MEDLGENTMVLSTLR | 100% Ac- | 100.00 | Q9Y6D9 | MD1L1_HUMAN | **Mitotic spindle assembly checkpoint protein MAD1** |  |
| **1** | | 12 | Ace | | ME | MEDLLDLDEELR | 100% Ac- | 100.00 | Q96FT9 | CN179_HUMAN | **Uncharacterized protein C14orf179** |  |
| **1** | | 28 | Ace | | ME | MEDSASASLSSAAATGTSTSTPAAPTAR | 100% Ac- | 100.00 | O76021 | RL1D1_HUMAN | **Ribosomal L1 domain-containing protein 1** |  |
| **1** | | 13 | Ace | | ME | MEDSMDMDMSPLR | N.D | N.D | P06748 | NPM_HUMAN | **Nucleophosmin** |  |
| **1** | | 10 | Ace | | ME | MEDSPLPDLR | 100% Ac- | 100.00 | Q8IV03 | CI150_HUMAN | **Uncharacterized protein C9orf150** |  |
| **1** | | 28 | Ace | | ME | MEDSQDLNEQSVKKTCTESDVSQSQNSR | N.D | N.D | O95677 | EYA4_HUMAN | **Eyes absent homolog 4** |  |
| **1** | | 15 | Ace | | ME | MEDVNSNVNADQEVR | 100% Ac- | 100.00 | Q9P270 | SLAI2_HUMAN | **SLAI motif-containing protein 2** |  |
| **1** | | 9 | Ace | | ME | MEDYLQGCR | 100% Ac- | 100.00 | Q86TP1 | PRUNE_HUMAN | **Protein prune homolog** |  |
| **1** | | 22 | Ace | | ME | MEDYTKIEKIGEGTYGVVYKGR | 100% Ac- | 100.00 | P06493 | CDC2_HUMAN | **Cell division control protein 2 homolog** |  |
| **1** | | 9 | Ace | | ME | MEDAAAPGR | 100% Ac- | 100.00 | Q9BV19 | CA050_HUMAN | **Uncharacterized protein C1orf50** |  |
| **1** | | 10 | Ace | | ME | MEEAELVKGR | 100% Ac- | 100.00 | Q9NP74 | PALMD_HUMAN | **Palmdelphin** |  |
| **1** | | 14 | Ace | | ME | MEEDEFIGEKTFQR | N.D | N.D | Q9H0Y0 | ATG10_HUMAN | **Autophagy-related protein 10** |  |
| **1** | | 8 | Ace | | ME | MEEDIDTR | N.D | N.D | Q00005 | 2ABB_HUMAN | **Serine/threonine-protein phosphatase 2A 55 kDa regulatory subunit B beta isoform** |  |
| **1** | | 9 | Ace | | ME | MEEDQELER | 100% Ac- | 100.00 | Q9Y2S0 | RPAC2_HUMAN | **DNA-directed RA polymerases I and III subunit RPAC2** |  |
| **1** | | 12 | Ace | | ME | MEEEAETEEQQR | 100% Ac- | 100.00 | Q8N2Z9 | CENPS_HUMAN | **Centromere protein S** |  |
| **1** | | 16 | Ace | | ME | MEEEGLECPNSSSEKR | 100% Ac- | 100.00 | Q7L7V1 | DHX32_HUMAN | **Putative pre-mRA-splicing factor ATP-dependent RA helicase DHX32** |  |
| **1** | | 14 | Ace | | ME | MEEEGVKEAGEKPR | N.D | N.D | Q8TD55 | PKHO2_HUMAN | **Pleckstrin homology domain-containing family O member 2** |  |
| **1** | | 28 | Ace | | ME | MEEEIAALVIDNGSGMCKAGFAGDDAPR | Partial-Ac- | 79.39 | P63261 | ACTG_HUMAN | **Actin, cytoplasmic 2** |  |
| **1** | | 22 | Ace | | ME | MEEIGILVEKAQDEIPALSVSR | N.D | N.D | P43686 | PRS6B_HUMAN | **26S protease regulatory subunit 6B** |  |
| **1** | | 14 | Ace | | ME | MEEIPAQEAAGSPR | 100% Ac- | 100.00 | Q8N720 | ZN655_HUMAN | **Zinc finger protein 655** |  |
| **1** | | 17 | Ace | | ME | MEEIYAKFVSQKISKTR | 100% Ac- | 100.00 | Q8NFH3 | NUP43_HUMAN | **Nucleoporin up43** |  |
| **1** | | 9 | Ace | | ME | MEELIVELR | 100% Ac- | 100.00 | Q8N556 | AFAP1_HUMAN | **Actin filament-associated protein 1** |  |
| **1** | | 10 | Ace | | ME | MEELSADEIR | 100% Ac- | 100.00 | O95155 | UBE4B_HUMAN | **Ubiquitin conjugation factor E4 B** |  |
| **1** | | 9 | Ace | | ME | MEELVVEVR | 100% Ac- | 100.00 | Q06787 | FMR1_HUMAN | **Fragile X mental retardation 1 protein** |  |
| **1** | | 11 | Ace | | ME | MEEPQAGDAAR | 100% Ac- | 100.00 | O15446 | RPA34_HUMAN | **DNA-directed RA polymerase I subunit RPA34** |  |
| **1** | | 21 | Ace | | ME | MEEQPQMQDADEPADSGGEGR | Partial-Ac- | 75.97 | Q9H3R5 | CENPH_HUMAN | **Centromere protein H** |  |
| **1** | | 12 | Ace | | ME | MEEQQPEPKSQR | 100% Ac- | 100.00 | Q05925 | HME1_HUMAN | **Homeobox protein engrailed-1** |  |
| **1** | | 18 | Ace | | ME | MEESDSEKTTEKENLGPR | N.D | N.D | Q5VZM2 | RRAGB_HUMAN | **Ras-related GTP-binding protein B** |  |
| **1** | | 11 | Ace | | ME | MEFAELIKTPR | 100% Ac- | 100.00 | Q96QG7 | MTMR9_HUMAN | **Myotubularin-related protein 9** |  |
| **1** | | 9 | Ace | | ME | MEFDCEGLR | 100% Ac- | 100.00 | Q8IX04 | UEVLD_HUMAN | **Ubiquitin-conjugating enzyme E2 variant 3** |  |
| **1** | | 15 | Ace | | ME | MEFQAVVMAVGGGSR | N.D | N.D | Q9NR50 | EI2BG_HUMAN | **Translation initiation factor eIF-2B subunit gamma** |  |
| **1** | | 11 | Ace | | ME | MEGAGAGSGFR | 100% Ac- | 100.00 | A8MT69 | STR13_HUMAN | **Stimulated by retinoic acid gene 13 protein homolog** |  |
| **1** | | 22 | Ace | | ME | MEGDAVEAIVEESETFIKGKER | 100% Ac- | 100.00 | P49711 | CTCF_HUMAN | **Transcriptional repressor CTCF** |  |
| **1** | | 11 | Ace | | ME | MEGEPPPVEER | 100% Ac- | 100.00 | Q6IEG0 | CF151_HUMAN | **U11/U12 small nuclear ribonucleoprotein 48 kDa protein** |  |
| **1** | | 13 | Ace | | ME | MEGGPAVCCQDPR | 100% Ac- | 100.00 | Q8N5S9 | KKCC1_HUMAN | **Calcium/calmodulin-dependent protein kinase kinase 1** |  |
| **2** | | 17 | Ace | | ME | MEGLDDGPDFLSEEDR | 100% Ac- | 100.00 | Q9UNH7 | SNX6_HUMAN | **Sorting nexin-6** |  |
| **1** | | 24 | Ace | | ME | MEGLEENGGVVQVGELLPCKICGR | 100% Ac- | 100.00 | Q96GY0 | F164A_HUMAN | **UPF0418 protein FAM164A** |  |
| **1** | | 11 | Ace | | ME | MEGPLSVFGDR | N.D | N.D | P17987 | TCPA_HUMAN | **T-complex protein 1 subunit alpha** |  |
| **1** | | 10 | Ace | | ME | MEGQDEVSAR | N.D | N.D | Q8WVP7 | LMBR1_HUMAN | **Limb region 1 protein homolog** |  |
| **1** | | 23 | Ace | | ME | MEGQSVEELLAKAEQDEAEKLQR | 100% Ac- | 100.00 | Q15050 | RRS1_HUMAN | **Ribosome biogenesis regulatory protein homolog** |  |
| **1** | | 20 | Ace | | ME | MEGSKTSNNSTMQVSFVCQR | 100% Ac- | 100.00 | Q14457 | BECN1_HUMAN | **Beclin-1** |  |
| **1** | | 7 | Ace | | ME | MEGSLER | 100% Ac- | 100.00 | P49366 | DHYS_HUMAN | **Deoxyhypusine synthase** | Q7Z4V0 (619-625) |
| **1** | | 22 | Ace | | ME | MEGVEEKKKEVPAVPETLKKKR | 100% Ac- | 100.00 | P18124 | RL7_HUMAN | **60S ribosomal protein L7** |  |
| **1** | | 19 | Ace | | ME | MEIGSAGPAGAQPLLMVPR | 100% Ac- | 100.00 | Q9H9G7 | I2C3_HUMAN | **Eukaryotic translation initiation factor 2C 3** |  |
| **1** | | 9 | Ace | | ME | MEIGTEISR | 100% Ac- | 100.00 | Q6PJT7 | ZC3HE_HUMAN | **Zinc finger CCCH domain-containing protein 14** |  |
| **1** | | 44 | Ace | | ME | MEKATVPVAAATAAEGEGSPPAVAAVAGPPAAAEVGGGVGGSSR | 100% Ac- | 100.00 | O75179 | ANR17_HUMAN | **Ankyrin repeat domain-containing protein 17** |  |
| **1** | | 8 | Ace | | ME | MEKDGLCR | 100% Ac- | 100.00 | Q00534 | CDK6_HUMAN | **Cell division protein kinase 6** |  |
| **1** | | 8 | Ace | | ME | MEKEETTR | 100% Ac- | 100.00 | Q09666 | AHNK_HUMAN | **Neuroblast differentiation-associated protein AHAK** |  |
| **1** | | 28 | Ace | | ME | MEKLYSENEGMASNQGKMENEEQPQDER | 100% Ac- | 100.00 | Q96EI5 | TCAL4_HUMAN | **Transcription elongation factor A protein-like 4** |  |
| **1** | | 18 | Ace | | ME | MEKTELIQKAKLAEQAER | 100% Ac- | 100.00 | P27348 | 1433T_HUMAN | **14-3-3 protein theta** |  |
| **1** | | 12 | Ace | | ME | MEKTLETVPLER | 100% Ac- | 100.00 | P22626 | ROA2_HUMAN | **Heterogeneous nuclear ribonucleoproteins A2/B1** |  |
| **1** | | 10 | Ace | | ME | MEKTQETVQR | 100% Ac- | 100.00 | O95749 | GGPPS_HUMAN | **Geranylgeranyl pyrophosphate synthetase** |  |
| **1** | | 8 | Ace | | ME | MELAQEAR | 100% Ac- | 100.00 | O94927 | K0841_HUMAN | **Uncharacterized protein KIAA0841** |  |
| **1** | | 8 | Ace | | ME | MELEAMSR | 100% Ac- | 100.00 | P61165 | CK010_HUMAN | **UPF0197 transmembrane protein C11orf10** |  |
| **1** | | 23 | Ace | | ME | MELEDGVVYQEEPGGSGAVMSER | 100% Ac- | 100.00 | O60271 | JIP4_HUMAN | **C-jun-amino-terminal kinase-interacting protein 4** |  |
| **1** | | 16 | Ace | | ME | MELENIVANTVLLKAR | 100% Ac- | 100.00 | P34947 | GRK5_HUMAN | **G protein-coupled receptor kinase 5** | P43250 (1-16) |
| **1** | | 6 | Ace | | ME | MELEVR | N.D | N.D | P51648 | AL3A2_HUMAN | **Fatty aldehyde dehydrogenase** |  |
| **1** | | 8 | Ace | | ME | MELFLAGR | 100% Ac- | 100.00 | Q7Z4W1 | DCXR_HUMAN | **L-xylulose reductase** |  |
| **1** | | 24 | Ace | | ME | MELGELLYNKSEYIETASGNKVSR | N.D | N.D | Q9BTE1 | DCTN5_HUMAN | **Dynactin subunit 5** |  |
| **1** | | 10 | Ace | | ME | MELGKGKLLR | 100% Ac- | 100.00 | Q9H6R7 | CB044_HUMAN | **WD repeat-containing protein C2orf44** |  |
| **1** | | 11 | Ace | | ME | MELGSCLEGGR | 100% Ac- | 100.00 | O95551 | TTRAP_HUMAN | **TRAF and TF receptor-associated protein** |  |
| **1** | | 15 | Ace | | ME | MELITILEKTVSPDR | 100% Ac- | 100.00 | Q14974 | IMB1_HUMAN | **Importin subunit beta-1** |  |
| **1** | | 10 | Ace | | ME | MELLCCEGTR | 100% Ac- | 100.00 | P30281 | CCND3_HUMAN | **G1/S-specific cyclin-D3** |  |
| **1** | | 18 | Ace | | ME | MELLGEYVGQEGKPQKLR | 100% Ac- | 100.00 | Q9BXV9 | CN142_HUMAN | **Uncharacterized protein C14orf142** |  |
| **1** | | 20 | Ace | | ME | MELQDPKMNGALPSDAVGYR | 100% Ac- | 100.00 | Q8WUX1 | S38A5_HUMAN | **Sodium-coupled neutral amino acid transporter 5** |  |
| **1** | | 35 | Ace | | ME | MELQKGKGAAAAAAASGAAGGGGGGAGAGAPGGGR | 100% Ac- | 100.00 | Q68E01 | INT3_HUMAN | **Integrator complex subunit 3** |  |
| **1** | | 9 | Ace | | ME | MELSAEYLR | 100% Ac- | 100.00 | Q9H3K6 | BOLA2_HUMAN | **BolA-like protein 2** |  |
| **1** | | 9 | Ace | | ME | MELSAVGER | 100% Ac- | 100.00 | O95503 | CBX6_HUMAN | **Chromobox protein homolog 6** | Q9HC52 (1-9) |
| **1** | | 26 | Ace | | ME | MELSDANLQTLTEYLKKTLDPDPAIR | 100% Ac- | 100.00 | P55060 | XPO2_HUMAN | **Exportin-2** |  |
| **1** | | 18 | Ace | | ME | MELSESVQKGFQMLADPR | 100% Ac- | 100.00 | Q9UBI1 | COMD3_HUMAN | **COMM domain-containing protein 3** |  |
| **1** | | 9 | Ace | | ME | MELVQVLKR | N.D | N.D | Q9UI09 | NDUAC_HUMAN | **ADH dehydrogenase [ubiquinone] 1 alpha subcomplex subunit 12** |  |
| **1** | | 12 | Ace | | ME | MEMKKKINLELR | 100% Ac- | 100.00 | Q9BTT0 | AN32E_HUMAN | **Acidic leucine-rich nuclear phosphoprotein 32 family member E** |  |
| **1** | | 13 | Ace | | ME | MEMPLPPDDQELR | 100% Ac- | 100.00 | Q8IWX8 | CHERP_HUMAN | **Calcium homeostasis endoplasmic reticulum protein** |  |
| **1** | | 15 | Ace | | ME | MEMSGLSFSEMEGCR | 100% Ac- | 100.00 | Q5JPI3 | CC038_HUMAN | **Uncharacterized protein C3orf38** |  |
| **1** | | 13 | Ace | | ME | MEMTEMTGVSLKR | 100% Ac- | 100.00 | Q96PZ0 | PUS7_HUMAN | **Pseudouridylate synthase 7 homolog** |  |
| **1** | | 19 | Ace | | ME | MENGAVYSPTTEEDPGPAR | N.D | N.D | Q9NX76 | CKLF6_HUMAN | **CKLF-like MARVEL transmembrane domain-containing protein 6** |  |
| **1** | | 29 | Ace | | ME | MENMAEEELLPLEKEEVEVAQVQVPTPAR | 100% Ac- | 100.00 | Q7Z2T5 | TRM1L_HUMAN | **TRM1-like protein** |  |
| **1** | | 12 | Ace | | ME | MENQLAKSTEER | 100% Ac- | 100.00 | Q9UKG9 | OCTC_HUMAN | **Peroxisomal carnitine O-octanoyltransferase** |  |
| **1** | | 19 | Ace | | ME | MENSDSNDKGSGDQSAAQR | 100% Ac- | 100.00 | Q9NVW2 | RNF12_HUMAN | **E3 ubiquitin-protein ligase RF12** |  |
| **1** | | 24 | Ace | | ME | MENSQLCKLFIGGLNVQTSESGLR | 100% Ac- | 100.00 | Q13151 | ROA0_HUMAN | **Heterogeneous nuclear ribonucleoprotein A0** |  |
| **1** | | 17 | Ace | | ME | MENSSAASASSEAGSSR | 100% Ac- | 100.00 | O43310 | K0427_HUMAN | **Uncharacterized protein KIAA0427** |  |
| **1** | | 10 | Ace | | ME | MENSTTTISR | 100% Ac- | 100.00 | Q14651 | PLSI_HUMAN | **Plastin-1** |  |
| **1** | | 16 | Ace | | ME | MEPAEQPSELVSAEGR | 100% Ac- | 100.00 | P47224 | MSS4_HUMAN | **Guanine nucleotide exchange factor MSS4** |  |
| **1** | | 19 | Ace | | ME | MEPAGPCGFCPAGEVQPAR | 100% Ac- | 100.00 | Q9UHR6 | ZNHI2_HUMAN | **Zinc finger HIT domain-containing protein 2** |  |
| **1** | | 13 | Ace | | ME | MEPAMEPETLEAR | Partial-Ac- | 84.51 | Q9UJY5 | GGA1_HUMAN | **ADP-ribosylation factor-binding protein GGA1** |  |
| **1** | | 9 | Ace | | ME | MEPAPSEVR | 100% Ac- | 100.00 | Q9Y4R8 | TELO2_HUMAN | **Telomere length regulation protein TEL2 homolog** |  |
| **1** | | 37 | Ace | | ME | MEPAVGGPGPLIVNNKQPQPPPPPPPAAAQPPPGAPR | 100% Ac- | 100.00 | Q5VSL9 | FA40A_HUMAN | **Protein FAM40A** |  |
| **1** | | 15 | Ace | | ME | MEPDGTYEPGFVGIR | 100% Ac- | 100.00 | P36954 | RPB9_HUMAN | **DNA-directed RA polymerase II subunit RPB9** |  |
| **1** | | 16 | Ace | | ME | MEPDPEPAAVEVPAGR | N.D | N.D | Q7Z6J9 | SEN54_HUMAN | **tRA-splicing endonuclease subunit Sen54** |  |
| **1** | | 7 | Ace | | ME | MEPEAPR | 100% Ac- | 100.00 | P48163 | MAOX_HUMAN | **ADP-dependent malic enzyme** |  |
| **1** | | 11 | Ace | | ME | MEPEEGTPLWR | 100% Ac- | 100.00 | Q9NX08 | COMD8_HUMAN | **COMM domain-containing protein 8** |  |
| **1** | | 12 | Ace | | ME | MEPELAAQKQPR | N.D | N.D | Q7Z628 | ARHG8_HUMAN | **Neuroepithelial cell-transforming gene 1 protein** |  |
| **1** | | 29 | Ace | | ME | MEPEQMLEGQTQVAENPHSEYGLTDNVER | 100% Ac- | 100.00 | Q9C005 | DPY30_HUMAN | **Protein dpy-30 homolog** |  |
| **1** | | 8 | Ace | | ME | MEPFTNDR | 100% Ac- | 100.00 | Q9UIQ6 | LCAP_HUMAN | **Leucyl-cystinyl aminopeptidase** |  |
| **1** | | 9 | Ace | | ME | MEPGEVKDR | 100% Ac- | 100.00 | Q8IWE5 | PKHM2_HUMAN | **Pleckstrin homology domain-containing family M member 2** |  |
| **1** | | 17 | Ace | | ME | MEPGPDGPAASGPAAIR | 100% Ac- | 100.00 | P19623 | SPEE_HUMAN | **Spermidine synthase** |  |
| **1** | | 9 | Ace | | ME | MEPGTNSFR | 100% Ac- | 100.00 | O43298 | ZBT43_HUMAN | **Zinc finger and BTB domain-containing protein 43** |  |
| **1** | | 16 | Ace | | ME | MEPKASCPAAAPLMER | 100% Ac- | 100.00 | Q96CD2 | COAC_HUMAN | **Phosphopantothenoylcysteine decarboxylase** |  |
| **1** | | 14 | Ace | | ME | MEPLKVEKFATAKR | 100% Ac- | 100.00 | Q9H7B4 | SMYD3_HUMAN | **SET and MYD domain-containing protein 3** |  |
| **1** | | 15 | Ace | | ME | MEPLAAYPLKCSGPR | 100% Ac- | 100.00 | Q8TED1 | GPX8_HUMAN | **Probable glutathione peroxidase 8** |  |
| **1** | | 29 | Ace | | ME | MEPNDSTSTAVEEPDSLEVLVKTLDSQTR | 100% Ac- | 100.00 | P46379 | BAT3_HUMAN | **Large proline-rich protein BAT3** |  |
| **1** | | 12 | Ace | | ME | MEPPGGSLGPGR | 100% Ac- | 100.00 | O60610 | DIAP1_HUMAN | **Protein diaphanous homolog 1** |  |
| **1** | | 35 | Ace | | ME | MEPPSCIQDEPFPHPLEPEPGVSAQPGPGKPSDKR | N.D | N.D | O60343 | TBCD4_HUMAN | **TBC1 domain family member 4** |  |
| **1** | | 11 | Ace | | ME | MEPSSKKLTGR | 100% Ac- | 100.00 | P11166 | GTR1_HUMAN | **Solute carrier family 2, facilitated glucose transporter member 1** |  |
| **1** | | 15 | Ace | | ME | MEPSSLELPADTVQR | 100% Ac- | 100.00 | Q16719 | KYNU_HUMAN | **Kynureninase** |  |
| **1** | | 25 | Ace | | ME | MEPTAPSLTEEDLTEVKKDALENLR | 100% Ac- | 100.00 | O95999 | BCL10_HUMAN | **B-cell lymphoma/leukemia 10** |  |
| **1** | | 11 | Ace | | ME | MEPVGCCGECR | 100% Ac- | 100.00 | Q8NEZ5 | FBX22_HUMAN | **F-box only protein 22** |  |
| **1** | | 22 | Ace | | ME | MEPAAGSSMEPSADWLATAAAR | 100% Ac- | 100.00 | P42771 | CD2A1_HUMAN | **Cyclin-dependent kinase inhibitor 2A, isoforms 1/2/3** |  |
| **1** | | 11 | Ace | | ME | MEPAAAATVQR | 100% Ac- | 100.00 | O60346 | PHLPP_HUMAN | **PH domain leucine-rich repeat-containing protein phosphatase** |  |
| **1** | | 9 | Ace | | ME | MEQCACVER | 100% Ac- | 100.00 | Q96G75 | RMD5B_HUMAN | **Protein RMD5 homolog B** |  |
| **1** | | 17 | Ace | | ME | MEQEPQNGEPAEIKIIR | 100% Ac- | 100.00 | Q8N0X7 | SPG20_HUMAN | **Spartin** |  |
| **1** | | 10 | Ace | | ME | MEQLSSANTR | 100% Ac- | 100.00 | P30740 | ILEU_HUMAN | **Leukocyte elastase inhibitor** |  |
| **1** | | 27 | Ace | | ME | MEQPGQDPTSDDVMDSFLEKFQSQPYR | 100% Ac- | 100.00 | O95801 | TTC4_HUMAN | **Tetratricopeptide repeat protein 4** |  |
| **1** | | 22 | Ace | | ME | MEQPGAAASGAGGGSEEPGGGR | 100% Ac- | 100.00 | O60879 | DIAP2_HUMAN | **Protein diaphanous homolog 2** |  |
| **1** | | 19 | Ace | | ME | MEQSPPPAPEPTQGPTPAR | 100% Ac- | 100.00 | Q96AY4 | TTC28_HUMAN | **Tetratricopeptide repeat protein 28** |  |
| **1** | | 9 | Ace | | ME | MEQVAEGAR | 100% Ac- | 100.00 | Q99549 | MPP8_HUMAN | **M-phase phosphoprotein 8** |  |
| **1** | | 8 | Ace | | ME | MEQVEILR | 100% Ac- | 100.00 | Q05209 | PTN12_HUMAN | **Tyrosine-protein phosphatase non-receptor type 12** |  |
| **1** | | 11 | Ace | | ME | MESAGLEQLLR | 100% Ac- | 100.00 | Q8TEX9 | IPO4_HUMAN | **Importin-4** |  |
| **1** | | 12 | Ace | | ME | MESAIAEGGASR | 100% Ac- | 100.00 | P78344 | IF4G2_HUMAN | **Eukaryotic translation initiation factor 4 gamma 2** |  |
| **1** | | 8 | Ace | | ME | MESALTAR | 100% Ac- | 100.00 | O00159 | MYO1C_HUMAN | **Myosin-Ic** |  |
| **1** | | 8 | Ace | | ME | MESDFYLR | 100% Ac- | 100.00 | P61326 | MGN_HUMAN | **Protein mago nashi homolog** |  |
| **1** | | 9 | Ace | | ME | MESEDLSGR | 100% Ac- | 100.00 | P33981 | TTK_HUMAN | **Dual specificity protein kinase TTK** |  |
| **1** | | 11 | Ace | | ME | MESGDEAAIER | 100% Ac- | 100.00 | Q8TDP1 | RNH2C_HUMAN | **Ribonuclease H2 subunit C** |  |
| **1** | | 14 | Ace | | ME | MESGKMAPPKNAPR | 100% Ac- | 100.00 | Q9HBM6 | TAF9B_HUMAN | **Transcription initiation factor TFIID subunit 9B** |  |
| **1** | | 13 | Ace | | ME | MESGSTAASEEAR | 100% Ac- | 100.00 | P10644 | KAP0_HUMAN | **cAMP-dependent protein kinase type I-alpha regulatory subunit** |  |
| **1** | | 15 | Ace | | ME | MESMFSSPAEAALQR | Partial-Ac- | 48.81 | Q9BQC3 | DPH2_HUMAN | **Diphthamide biosynthesis protein 2** |  |
| **1** | | 21 | Ace | | ME | MESPASSQPASMPQSKGKSKR | 100% Ac- | 100.00 | P46734 | MP2K3_HUMAN | **Dual specificity mitogen-activated protein kinase kinase 3** |  |
| **1** | | 12 | Ace | | ME | MESVSCSAAAVR | 100% Ac- | 100.00 | Q15814 | TBCC_HUMAN | **Tubulin-specific chaperone C** |  |
| **1** | | 21 | Ace | | ME | MESYHKPDQQKLQALKDTANR | 100% Ac- | 100.00 | P29401 | TKT_HUMAN | **Transketolase** |  |
| **1** | | 22 | Ace | | ME | METAPKPGKDVPPKKDKLQTKR | 100% Ac- | 100.00 | O15213 | WDR46_HUMAN | **WD repeat-containing protein 46** |  |
| **1** | | 34 | Ace | | ME | METDCNPMELSSMSGFEEGSELNGFEGTDMKDMR | 100% Ac- | 100.00 | Q9P0R6 | GSKIP_HUMAN | **GSK3-beta interaction protein** |  |
| **1** | | 10 | Ace | | ME | METDLNSQDR | 100% Ac- | 100.00 | O75143 | K0652_HUMAN | **UPF0630 protein KIAA0652** |  |
| **1** | | 22 | Ace | | ME | METEQPEETFPNTETNGEFGKR | 100% Ac- | 100.00 | P61978 | HNRPK_HUMAN | **Heterogeneous nuclear ribonucleoprotein K** |  |
| **1** | | 28 | Ace | | ME | METESGNQEKVMEEESTEKKKEVEKKKR | 100% Ac- | 100.00 | Q4G0J3 | LARP7_HUMAN | **La-related protein 7** |  |
| **1** | | 9 | Ace | | ME | METILEQQR | 100% Ac- | 100.00 | Q12874 | SF3A3_HUMAN | **Splicing factor 3A subunit 3** |  |
| **1** | | 11 | Ace | | ME | METLQSETKTR | 100% Ac- | 100.00 | Q9BWK5 | MRI_HUMAN | **Modulator of retrovirus infection homolog** |  |
| **1** | | 8 | Ace | | ME | METLSFPR | 100% Ac- | 100.00 | Q9BZD4 | NUF2_HUMAN | **Kinetochore protein uf2** |  |
| **1** | | 14 | Ace | | ME | METLSNASGTFAIR | 100% Ac- | 100.00 | P50453 | SPB9_HUMAN | **Serpin B9** |  |
| **1** | | 13 | Ace | | ME | METMASPGKDNYR | 100% Ac- | 100.00 | O60684 | IMA7_HUMAN | **Importin subunit alpha-7** |  |
| **1** | | 10 | Ace | | ME | METPLDVLSR | 100% Ac- | 100.00 | Q14135 | VGLL4_HUMAN | **Transcription cofactor vestigial-like protein 4** |  |
| **1** | | 21 | Ace | | ME | METQKDEAAQAKGAAASGSTR | N.D | N.D | Q5JTC6 | F123B_HUMAN | **Protein FAM123B** |  |
| **1** | | 18 | Ace | | ME | METSALKQQEQPAATKIR | 100% Ac- | 100.00 | P40937 | RFC5_HUMAN | **Replication factor C subunit 5** |  |
| **1** | | 7 | Ace | | ME | METVQLR | 100% Ac- | 100.00 | Q13043 | STK4_HUMAN | **Serine/threonine-protein kinase 4** |  |
| **1** | | 11 | Ace | | ME | MEVDAPGVDGR | 100% Ac- | 100.00 | Q8WVX3 | HCFT1_HUMAN | **Hepatitis C virus F protein-transactivated protein 1** |  |
| **1** | | 10 | Ace | | ME | MEVDINGESR | 100% Ac- | 100.00 | Q96GV9 | CE030_HUMAN | **UPF0684 protein C5orf30** |  |
| **1** | | 9 | Ace | | ME | MEVEAAEAR | 100% Ac- | 100.00 | Q96K58 | ZN668_HUMAN | **Zinc finger protein 668** |  |
| **1** | | 29 | Ace | | ME | MEVGGDTAAPAPGGAEDLEDTQFPSEEAR | 100% Ac- | 100.00 | Q7L1V2 | MON1B_HUMAN | **Vacuolar fusion protein MO1 homolog B** |  |
| **1** | | 22 | Ace | | ME | MEVKGKKQFTGKSTKTAQEKNR | 100% Ac- | 100.00 | Q15397 | K0020_HUMAN | **Pumilio domain-containing protein KIAA0020** |  |
| **1** | | 14 | Ace | | ME | MEVLAAETTSQQER | 100% Ac- | 100.00 | O75781 | PALM_HUMAN | **Paralemmin** |  |
| **1** | | 8 | Ace | | ME | MEVPAAGR | 100% Ac- | 100.00 | Q9Y5B0 | CTDP1_HUMAN | **RNA polymerase II subunit A C-terminal domain phosphatase** |  |
| **1** | | 9 | Ace | | ME | MEVQKEAQR | N.D | N.D | Q9BTL4 | IER2_HUMAN | **Immediate early response gene 2 protein** |  |
| **1** | | 27 | Ace | | ME | MEVSPLQPVNENMQVNKIKKNEDAKKR | 100% Ac- | 100.00 | P11388 | TOP2A_HUMAN | **DNA topoisomerase 2-alpha** |  |
| **1** | | 9 | Ace | | ME | MEVYIPSFR | 100% Ac- | 100.00 | Q9Y343 | SNX24_HUMAN | **Sorting nexin-24** |  |
| **1** | | 10 | Ace | | ME | MEVAANCSLR | 100% Ac- | 100.00 | Q96RS6 | NUDC1_HUMAN | **NudC domain-containing protein 1** |  |
| **1** | | 9 | Ace | | ME | MEYDEKLAR | 100% Ac- | 100.00 | Q6ZN54 | DEFI8_HUMAN | **Differentially expressed in FDCP 8 homolog** |  |
| **1** | | 19 | Ace | | ME | MEYLIGIQGPDYVLVASDR | 100% Ac- | 100.00 | P49721 | PSB2_HUMAN | **Proteasome subunit beta type-2** |  |
| **1** | | 10 | Ace | | ME | MEYMAESTDR | 100% Ac- | 100.00 | Q96D46 | NMD3_HUMAN | **60S ribosomal export protein MD3** |  |
| **1** | | 14 | Ace | | ME | MEAAAEPGNLAGVR | 100% Ac- | 100.00 | Q9Y5Y2 | NUBP2_HUMAN | **Nucleotide-binding protein 2** |  |
| **1** | | 10 | Ace | | ME | MEAAAVTVTR | 100% Ac- | 100.00 | Q8NFI3 | ENASE_HUMAN | **Cytosolic endo-beta--acetylglucosaminidase** |  |
| **1** | | 19 | Ace | | ME | MEAAVGVPDGGDQGGAGPR | 100% Ac- | 100.00 | Q01804 | OTUD4_HUMAN | **OTU domain-containing protein 4** |  |
| **8. Met-Phe-** | | |  | |  |  |  |  |  |  |  |  |
| 1 | | 7 | Ace | | MF | MFAKAFR | 100% Ac- | 100.00 | P41214 | LIGA_HUMAN | **Ligatin** |  |
| 1 | | 7 | Ace | | MF | MFAKATR | 100% Ac- | 100.00 | O60443 | DFNA5_HUMAN | **Non-syndromic hearing impairment protein 5** |  |
| 1 | | 17 | Ace | | MF | MFAKGKGSAVPSDGQAR | 100% Ac- | 100.00 | Q9BWW4 | SSBP3_HUMAN | **Single-stranded DA-binding protein 3** |  |
| 1 | | 11 | AcD3 | | MF | MFCEKAMELIR | Partial-Ac- | 23.81 | Q14691 | PSF1_HUMAN | **DNA replication complex GIS protein PSF1** |  |
| 1 | | 44 | Ace | | MF | MFEEKASSPSGKMGGEEKPIGAGEEKQKEGGKKKNKEGSGDGGR | Partial-Ac- | 52.68 | P26639 | SYTC_HUMAN | **Threonyl-tRA synthetase, cytoplasmic** |  |
| 3 | | 13 | Ace | | MF | MFESADSTATR | N.D | N.D | Q9BV29 | CO057_HUMAN | **Uncharacterized protein C15orf57** |  |
| 1 | | 20 | Ace | | MF | MFGAGDEDDTDFLSPSGGAR | 100% Ac- | 100.00 | Q5T1M5 | FKB15_HUMAN | **FK506-binding protein 15** |  |
| 1 | | 9 | AcD3 | | MF | MFGCLVAGR | Partial-Ac- | 11.52 | Q53FT3 | CK073_HUMAN | **Uncharacterized protein C11orf73** |  |
| 1 | | 10 | Ace | | MF | MFGIQESIQR | 100% Ac- | 100.00 | Q9UH73 | COE1_HUMAN | **Transcription factor COE1** |  |
| 1 | | 14 | Ace | | MF | MFGLDQFEPQVNSR | 100% Ac- | 100.00 | Q10571 | MN1_HUMAN | **Probable tumor suppressor protein M1** |  |
| 1 | | 13 | AcD3 | | MF | MFLQYYLNEQGDR | N.D | N.D | Q9NPE3 | NOP10_HUMAN | **H/ACA ribonucleoprotein complex subunit 3** |  |
| 1 | | 10 | AcD3 | | MF | MFLYNLTLQR | 100% free | 0.00 | Q15393 | SF3B3_HUMAN | **Splicing factor 3B subunit 3** |  |
| 1 | | 21 | Ace | | MF | MFPTGFSSPSPSAAAAAQEVR | 100% Ac- | 100.00 | Q13620 | CUL4B_HUMAN | **Cullin-4B** |  |
| 1 | | 9 | Ace | | MF | MFPAAPSPR | 100% Ac- | 100.00 | Q8WUM0 | NU133_HUMAN | **Nuclear pore complex protein up133** |  |
| 1 | | 20 | Ace | | MF | MFQIPEFEPSEQEDSSSAER | 100% Ac- | 100.00 | Q92934 | BAD_HUMAN | **Bcl2 antagonist of cell death** |  |
| 1 | | 13 | Ace | | MF | MFQLPVNNLGSLR | 100% Ac- | 100.00 | Q9H2P0 | ADNP_HUMAN | **Activity-dependent neuroprotector homeobox protein** |  |
| 1 | | 11 | Ace | | MF | MFQVPDSEGGR | 100% Ac- | 100.00 | Q9H910 | HN1L_HUMAN | **Hematological and neurological expressed 1-like protein** |  |
| 1 | | 17 | Ace | | MF | MFSALKKLVGSDQAPGR | 100% Ac- | 100.00 | Q3YEC7 | PARF_HUMAN | **Putative GTP-binding protein Parf** |  |
| 1 | | 9 | Ace | | MF | MFSEQAAQR | 100% Ac- | 100.00 | P49005 | DPOD2_HUMAN | **DNA polymerase delta subunit 2** |  |
| 1 | | 8 | Ace | | MF | MFSLDSFR | 100% Ac- | 100.00 | Q7L2E3 | DHX30_HUMAN | **Putative ATP-dependent RA helicase DHX30** |  |
| 1 | | 9 | Ace | | MF | MFSVESLER | 100% Ac- | 100.00 | Q86VS8 | HOOK3_HUMAN | **Protein Hook homolog 3** |  |
| 1 | | 10 | Ace | | MF | MFSWVSKDAR | 100% Ac- | 100.00 | Q9H4M9 | EHD1_HUMAN | **EH domain-containing protein 1** |  |
| 1 | | 15 | AcD3 | | MF | MFVELNNLLNTTPDR | 100% free | 0.00 | Q0PNE2 | TM103_HUMAN | **UPF0405 protein TMEM103** |  |
| 1 | | 15 | Ace | | MF | MFVQEEKIFAGKVLR | 100% Ac- | 100.00 | Q9BSL1 | UBAC1_HUMAN | **Ubiquitin-associated domain-containing protein 1** |  |
| **9. Met-Gly-** | | |  | |  |  |  |  |  |  |  |  |
| 1 | | 12 | Ace | | MG | MGDEMDAMIPER | 100% Ac- | 100.00 | P35658 | NU214_HUMAN | **Nuclear pore complex protein up214** |  |
| 1 | | 9 | Ace | | MG | MGEVEAPGR | N.D | N.D | Q96T60 | PNKP_HUMAN | **Bifunctional polynucleotide phosphatase/kinase** |  |
| 1 | | 19 | AcD3 | | MG | MGIQGLAKLIADVAPSAIR | Partial-Ac- | 8.34 | P39748 | FEN1_HUMAN | **Flap endonuclease 1** |  |
| 1 | | 10 | Ace | | MG | MGKGDPNKPR | 100% Ac- | 100.00 | P26583 | HMGB2_HUMAN | **High mobility group protein B2** |  |
| 1 | | 13 | AcD3 | | MG | MGKVKVGVNGFGR | Partial-Ac- | 14.46 | P04406 | G3P_HUMAN | **Glyceraldehyde-3-phosphate dehydrogenase** |  |
| 1 | | 20 | AcD3 | | MG | MGPPGPALPATMNNSSSETR | N.D | N.D | Q15003 | CND2_HUMAN | **Condensin complex subunit 2** |  |
| **10. Met-Ile-** | | |  | |  |  |  |  |  |  |  |  |
| 1 | | 8 | AcD3 | | MI | MIEDKGPR | Partial-Ac- | 15.12 | Q7Z401 | MYCPP_HUMAN | **C-myc promoter-binding protein** |  |
| 1 | | 9 | AcD3 | | MI | MIEESGNKR | N.D | N.D | O60941 | DTNB_HUMAN | **Dystrobrevin beta** |  |
| 1 | | 7 | Ace | | MI | MIEQQKR | Partial-Ac- | 56.97 | Q96DI7 | WDR57_HUMAN | **WD repeat-containing protein 57** |  |
| 1 | | 9 | AcD3 | | MI | MIEVVCNDR | 100% free | 0.00 | Q9BZL1 | UBL5_HUMAN | **Ubiquitin-like protein 5** |  |
| 1 | | 7 | AcD3 | | MI | MIIKEYR | 100% free | 0.00 | Q9BZ72 | PITM2_HUMAN | **Membrane-associated phosphatidylinositol transfer protein 2** |  |
| 1 | | 6 | AcD3 | | MI | MIIPVR | 100% free | 0.00 | P62875 | RPAB5_HUMAN | **DNA-directed RA polymerases I, II, and III subunit RPABC5** |  |
| 1 | | 33 | Ace | | MI | MIKLFSLKQQKKEEESAGGTKGSSKKASAAQLR | 100% Ac- | 100.00 | P61081 | UBC12_HUMAN | **EDD8-conjugating enzyme Ubc12** |  |
| 1 | | 9 | AcD3 | | MI | MILLEVNNR | 100% free | 0.00 | O15144 | ARPC2_HUMAN | **Actin-related protein 2/3 complex subunit 2** |  |
| 1 | | 14 | Ace | | MI | MISAAQLLDELMGR | 100% Ac- | 100.00 | O95232 | CROP_HUMAN | **Cisplatin resistance-associated overexpressed protein** |  |
| 1 | | 28 | AcD3 | | MI | MITKTHKVDLGLPEKKKKKKVVKEPETR | 100% free | 0.00 | Q1ED39 | CP088_HUMAN | **Protein C16orf88** |  |
| 1 | | 9 | Ace | | MI | MIVADSECR | 100% Ac- | 100.00 | P58004 | SESN2_HUMAN | **Sestrin-2** |  |
| **11. Met-Lys-** | |  |  | |  |  |  |  |  |  |  |  |
| 1 | | 13 | AcD3 | | MK | MKALILVGGYGTR | N.D | N.D | Q9Y5P6 | GMPPB_HUMAN | **Mannose-1-phosphate guanyltransferase beta** |  |
| 1 | | 8 | AcD3 | | MK | MKALSPVR | Partial-Ac- | 13.79 | Q02535 | ID3_HUMAN | **DNA-binding protein inhibitor ID-3** |  |
| 1 | | 22 | AcD3 | | MK | MKAQGETEESEKLSKMSSLLER | Partial-Ac- | 43.46 | Q15648 | MED1_HUMAN | **Mediator of RA polymerase II transcription subunit 1** |  |
| 1 | | 8 | AcD3 | | MK | MKASGTLR | Partial-Ac- | 16.19 | Q02543 | RL18A_HUMAN | **60S ribosomal protein L18a** |  |
| 1 | | 8 | AcD3 | | MK | MKAVKSER | Partial-Ac- | 49.03 | Q8TAD8 | SNIP1_HUMAN | **Smad nuclear-interacting protein 1** |  |
| 1 | | 11 | AcD3 | | MK | MKDKQKKKKER | 100% free | 0.00 | Q8IXJ9 | ASXL1_HUMAN | **Putative Polycomb group protein ASXL1** |  |
| 1 | | 10 | AcD3 | | MK | MKDSLVLLGR | 100% free | 0.00 | O76071 | CIAO1_HUMAN | **Protein CIAO1** |  |
| 1 | | 14 | AcD3 | | MK | MKEMSANTVLDSQR | Partial-Ac- | 16.95 | Q9BWT3 | PAPOG_HUMAN | **Poly(A) polymerase gamma** |  |
| 1 | | 11 | AcD3 | | MK | MKETPLSNCER | 100% free | 0.00 | Q06265 | EXOS9_HUMAN | **Exosome complex exonuclease RRP45** |  |
| 1 | | 11 | AcD3 | | MK | MKFNPFVTSDR | 100% free | 0.00 | P61254 | RL26_HUMAN | **60S ribosomal protein L26** | Q9UNX3 (1-11) |
| 1 | | 12 | AcD3 | | MK | MKGKEEKEGGAR | Partial-Ac- | 18.23 | Q9UNE7 | STUB1_HUMAN | **STIP1 homology and U box-containing protein 1** |  |
| 1 | | 14 | AcD3 | | MK | MKIEEVKSTTKTQR | 100% free | 0.00 | Q9Y265 | RUVB1_HUMAN | **RuvB-like 1** |  |
| 1 | | 7 | AcD3 | | MK | MKIFCSR | Partial-Ac- | 7.51 | Q9NVM4 | ANM7_HUMAN | **Protein arginine -methyltransferase 7** |  |
| 1 | | 8 | Ace | | MK | MKKAEMGR | 100% Ac- | 100.00 | Q96QD8 | S38A2_HUMAN | **Sodium-coupled neutral amino acid transporter 2** |  |
| 1 | | 8 | Ace | | MK | MKKFFDSR | 100% Ac- | 100.00 | Q2M2I8 | AAK1_HUMAN | **AP2-associated protein kinase 1** |  |
| 1 | | 10 | Ace | | MK | MKKISLKTLR | 100% Ac- | 100.00 | O14544 | SOCS6_HUMAN | **Suppressor of cytokine signaling 6** |  |
| 1 | | 7 | Ace | | MK | MKKQFNR | 100% Ac- | 100.00 | Q17R89 | RICH2_HUMAN | **Rho GTPase-activating protein RICH2** | Q68EM7 (1-7) |
| 1 | | 10 | Ace | | MK | MKKSYSGGTR | N.D | N.D | Q9UHD8 | SEPT9_HUMAN | **Septin-9** |  |
| 1 | | 9 | AcD3 | | MK | MKLKDTKSR | 100% free | 0.00 | Q9GZR7 | DDX24_HUMAN | **ATP-dependent RA helicase DDX24** |  |
| 1 | | 8 | AcD3 | | MK | MKLKEVDR | 100% free | 0.00 | O94979 | SC31A_HUMAN | **Protein transport protein Sec31A** |  |
| 2 | | 10 | AcD3 | | MK | MKLKSNQTR | Partial-Ac- | 16.72 | O95989 | NUDT3_HUMAN | **Diphosphoinositol polyphosphate phosphohydrolase 1** |  |
| 1 | | 22 | AcD3 | | MK | MKLNISFPATGCQKLIEVDDER | 100% free | 0.00 | P62753 | RS6_HUMAN | **40S ribosomal protein S6** |  |
| 1 | | 14 | AcD3 | | MK | MKLPIFIADAFTAR | 100% free | 0.00 | P30039 | PBLD_HUMAN | **Phenazine biosynthesis-like domain-containing protein** |  |
| 1 | | 13 | AcD3 | | MK | MKLSLTKVVNGCR | 100% Ac- | 100.00 | Q9H974 | QTRD1_HUMAN | **Queuine tRA-ribosyltransferase domain-containing protein 1** |  |
| 1 | | 9 | AcD3 | | MK | MKLTDSVLR | Partial-Ac- | 4.86 | Q6UXN9 | WDR82_HUMAN | **WD repeat-containing protein 82** |  |
| 1 | | 10 | AcD3 | | MK | MKMADAKQKR | 100% free | 0.00 | O14974 | MYPT1_HUMAN | **Protein phosphatase 1 regulatory subunit 12A** |  |
| 1 | | 13 | AcD3 | | MK | MKMFESADSTATR | Partial-Ac- | 13.17 | Q9BV29 | CO057_HUMAN | **Uncharacterized protein C15orf57** |  |
| 1 | | 16 | AcD3 | | MK | MKMTVDFEECLKDSPR | 100% free | 0.00 | Q15057 | ACAP2_HUMAN | **ARFGAP with coiled-coil, AK repeat and PH domain-containing protein 2** |  |
| 1 | | 22 | AcD3 | | MK | MKPLVVFVLGGPGAGKGTQCAR | 100% free | 0.00 | P30085 | KCY_HUMAN | **UMP-CMP kinase** |  |
| 1 | | 25 | AcD3 | | MK | MKPPAACAGDMADAASPCSVVNDLR | Partial-Ac- | 7.02 | P52888 | THOP1_HUMAN | **Thimet oligopeptidase** |  |
| 1 | | 9 | AcD3 | | MK | MKPTGTDPR | 100% free | 0.00 | Q15051 | IQCB1_HUMAN | **IQ calmodulin-binding motif-containing protein 1** |  |
| 1 | | 25 | AcD3 | | MK | MKQESAAPNTPPTSQSPTPSAQFPR | Partial-Ac- | 21.13 | Q9BUX1 | CHAC1_HUMAN | **Cation transport regulator-like protein 1** |  |
| 1 | | 14 | AcD3 | | MK | MKQLPVLEPGDKPR | Partial-Ac- | 28.15 | Q7Z6M1 | RABEK_HUMAN | **Rab9 effector protein with Kelch motifs** |  |
| 1 | | 10 | AcD3 | | MK | MKQLPAATVR | 100% free | 0.00 | P54277 | PMS1_HUMAN | **PMS1 protein homolog 1** |  |
| 1 | | 14 | Ace | | MK | MKSDCMQTTICQER | Partial-Ac- | 73.88 | O95620 | DUS4L_HUMAN | **tRA-dihydrouridine synthase 4-like** |  |
| 1 | | 9 | Ace | | MK | MKSLKAKFR | 100% Ac- | 100.00 | Q9P0K7 | RAI14_HUMAN | **Ankycorbin** |  |
| 1 | | 9 | Ace | | MK | MKSPDEVLR | Partial-Ac- | 75.80 | Q53GA4 | PHLA2_HUMAN | **Pleckstrin homology-like domain family A member 2** |  |
| 1 | | 8 | Ace | | MK | MKTAENIR | Partial-Ac- | 84.03 | Q8IV42 | PSTK_HUMAN | **L-seryl-tRA(Sec) kinase** |  |
| 1 | | 23 | AcD3 | | MK | MKTILSNQTVDIPENVDITLKGR | 100% free | 0.00 | P32969 | RL9_HUMAN | **60S ribosomal protein L9** |  |
| 1 | | 12 | AcD3 | | MK | MKTPFGKTPGQR | N.D | N.D | Q9Y657 | SPIN1_HUMAN | **Spindlin-1** |  |
| 1 | | 13 | AcD3 | | MK | MKTPFGKAAAGQR | 100% free | 0.00 | Q5JUX0 | SPIN3_HUMAN | **Spindlin-3** |  |
| 1 | | 15 | AcD3 | | MK | MKTPNAQEAEGQQTR | Partial-Ac- | 12.66 | Q99865 | SPI2A_HUMAN | **Spindlin-2A** | Q9BPZ2 (1-15) |
| 1 | | 8 | AcD3 | | MK | MKVKMLSR | 100% free | 0.00 | Q9NV06 | SOF1_HUMAN | **WD repeat and SOF domain-containing protein 1** |  |
| 1 | | 21 | AcD3 | | MK | MKVLLLKDAKEDDCGQDPYIR | N.D | N.D | P10746 | HEM4_HUMAN | **Uroporphyrinogen-III synthase** |  |
| 1 | | 7 | Ace | | MK | MKVSTLR | Partial-Ac- | 71.85 | Q9C0C2 | TB182_HUMAN | **182 kDa tankyrase 1-binding protein** |  |
| 1 | | 11 | AcD3 | | MK | MKVVPEKNAVR | 100% free | 0.00 | Q9C0C7 | AMRA1_HUMAN | **Activating molecule in BEC1-regulated autophagy protein 1** |  |
| 1 | | 9 | AcD3 | | MK | MKYKNLMAR | 100% free | 0.00 | Q14511 | CASL_HUMAN | **Enhancer of filamentation 1** |  |
| **12. Met-Leu-** | | | | |  |  |  |  |  |  |  |  |
| 1 | | 7 | Ace | | ML | MLALISR | 100% Ac- | 100.00 | Q9NVJ2 | ARL8B_HUMAN | **ADP-ribosylation factor-like protein 8B** |  |
| 1 | | 10 | AcD3 | | ML | MLDLEVVPER | 100% free | 0.00 | Q9BSU1 | CP070_HUMAN | **UPF0183 protein C16orf70** |  |
| 1 | | 9 | AcD3 | | ML | MLEAIDKNR | N.D | N.D | O75970 | MPDZ_HUMAN | **Multiple PDZ domain protein** |  |
| 1 | | 20 | Ace | | ML | MLEAPGPSDGCELSNPSASR | 100% Ac- | 100.00 | Q9UNI6 | DUS12_HUMAN | **Dual specificity protein phosphatase 12** |  |
| 1 | | 12 | AcD3 | | ML | MLEGDLVSKMLR | 100% free | 0.00 | Q8NHU6 | TDRD7_HUMAN | **Tudor domain-containing protein 7** |  |
| 1 | | 18 | AcD3 | | ML | MLESSGCKALKEGVLEKR | N.D | N.D | Q8WV24 | PHLA1_HUMAN | **Pleckstrin homology-like domain family A member 1** |  |
| 1 | | 16 | AcD3 | | ML | MLESYVTPILMSYVNR | N.D | N.D | Q7Z7G8 | VP13B_HUMAN | **Vacuolar protein sorting-associated protein 13B** |  |
| 1 | | 8 | AcD3 | | ML | MLFNSVLR | Partial-Ac- | 23.70 | Q9NZJ0 | DTL_HUMAN | **Denticleless protein homolog** |  |
| 1 | | 11 | Ace | | ML | MLGAPDESSVR | 100% Ac- | 100.00 | Q7Z4S6 | KI21A_HUMAN | **Kinesin-like protein KIF21A** |  |
| **1** | | 9 | Ace | | ML | MLGAVEGPR | 100% Ac- | 100.00 | Q14012 | KCC1A_HUMAN | **Calcium/calmodulin-dependent protein kinase type 1** |  |
| 1 | | 8 | Ace | | ML | MLGFLSAR | 100% Ac- | 100.00 | Q13216 | ERCC8_HUMAN | **DA excision repair protein ERCC-8** |  |
| 1 | | 9 | Ace | | ML | MLGGSLGSR | 100% Ac- | 100.00 | Q9Y3B8 | ORN_HUMAN | **Oligoribonuclease, mitochondrial** |  |
| 1 | | 12 | Ace | | ML | MLGNSAPGPATR | Partial-Ac- | 78.90 | P20248 | CCNA2_HUMAN | **Cyclin-A2** |  |
| 2 | | 16 | Ace | | ML | MLGPEGGEGFVVKLR | 100% Ac- | 100.00 | P52597 | HNRPF_HUMAN | **Heterogeneous nuclear ribonucleoprotein F** |  |
| 1 | | 10 | Ace | | ML | MLGSGFKAER | N.D | N.D | P53990 | K0174_HUMAN | **Uncharacterized protein KIAA0174** |  |
| 1 | | 28 | Ace | | ML | MLGSSVKSVQPEVELSSGGGDEGADEPR | 100% Ac- | 100.00 | Q8NEY1 | NAV1_HUMAN | **Neuron navigator 1** |  |
| 2 | | 16 | Ace | | ML | MLGTEGGEGFVVKVR | 100% Ac- | 100.00 | P31943 | HNRH1_HUMAN | **Heterogeneous nuclear ribonucleoprotein H** |  |
| 1 | | 9 | AcD3 | | ML | MLITVYCVR | 100% free | 0.00 | Q8WTU0 | DDI1_HUMAN | **Protein DDI1 homolog 1** |  |
| 1 | | 10 | Ace | | ML | MLKAVLKKSR | 100% Ac- | 100.00 | Q9C0D5 | TANC1_HUMAN | **Protein TAC1** |  |
| 1 | | 25 | Ace | | ML | MLKPQPLQQPSQPQQPPPTQQAVAR | 100% Ac- | 100.00 | Q8WWM7 | ATX2L_HUMAN | **Ataxin-2-like protein** |  |
| 1 | | 12 | Ace | | ML | MLKSKTFLKKTR | 100% Ac- | 100.00 | Q9Y2L1 | RRP44_HUMAN | **Exosome complex exonuclease RRP44** |  |
| 1 | | 19 | AcD3 | | ML | MLLFCPGCGNGLIVEEGQR | N.D | N.D | Q9Y2Y1 | RPC10_HUMAN | **DNA-directed RA polymerase III subunit RPC10** |  |
| 1 | | 11 | Ace | | ML | MLLLPSAADGR | Partial-Ac- | 71.35 | Q99543 | DNJC2_HUMAN | **DnaJ homolog subfamily C member 2** |  |
| 1 | | 8 | Ace | | ML | MLLSVTSR | 100% Ac- | 100.00 | Q15434 | RBMS2_HUMAN | **RNA-binding motif, single-stranded-interacting protein 2** |  |
| 1 | | 8 | AcD3 | | ML | MLMPKKNR | 100% free | 0.00 | P46783 | RS10_HUMAN | **40S ribosomal protein S10** | Q9NQ39 (1-8) |
| 1 | | 15 | AcD3 | | ML | MLPGVGVFGTSLTAR | 100% free | 0.00 | Q9NXC2 | GFOD1_HUMAN | **Glucose-fructose oxidoreductase domain-containing protein 1** |  |
| 1 | | 11 | AcD3 | | ML | MLQKPKSVKLR | Partial-Ac- | 22.11 | O76075 | DFFB_HUMAN | **DNA fragmentation factor subunit beta** |  |
| 1 | | 58 | AcD3 | | ML | MLSGKKAAAAAAAAAAAATGTEAGPGTAGGSENGSEVAAQPAGLSGPAEVGPGAVGER | 100% free | 0.00 | O60341 | LSD1_HUMAN | **Lysine-specific histone demethylase 1** |  |
| 1 | | 11 | AcD3 | | ML | MLSLDFLDDVR | N.D | N.D | P67812 | SC11A_HUMAN | **Signal peptidase complex catalytic subunit SEC11A** |  |
| 1 | | 11 | Ace | | ML | MLSLQYPDVYR | 100% Ac- | 100.00 | P48147 | PPCE_HUMAN | **Prolyl endopeptidase** |  |
| 1 | | 8 | Ace | | ML | MLSPEAER | 100% Ac- | 100.00 | Q9NUG6 | PDRG1_HUMAN | **p53 and DA damage-regulated protein 1** |  |
| 1 | | 13 | Ace | | ML | MLSSTAMYSAPGR | 100% Ac- | 100.00 | P20618 | PSB1_HUMAN | **Proteasome subunit beta type-1** |  |
| 1 | | 10 | Ace | | ML | MLTCNKAGSR | 100% Ac- | 100.00 | Q13362 | 2A5G_HUMAN | **Serine/threonine-protein phosphatase 2A 56 kDa regulatory subunit gamma isoform** |  |
| 1 | | 22 | Ace | | ML | MLTDSGGGGTSFEEDLDSVAPR | 100% Ac- | 100.00 | Q8IWZ3 | ANKH1_HUMAN | **Ankyrin repeat and KH domain-containing protein 1** |  |
| 1 | | 11 | AcD3 | | ML | MLTKFETKSAR | 100% free | 0.00 | P53621 | COPA_HUMAN | **Coatomer subunit alpha** |  |
| 1 | | 10 | Ace | | ML | MLTLASKLKR | 100% Ac- | 100.00 | Q969M7 | UBE2F_HUMAN | **EDD8-conjugating enzyme UBE2F** |  |
| 1 | | 10 | AcD3 | | ML | MLVVEVANGR | 100% free | 0.00 | Q9BSV6 | SEN34_HUMAN | **tRA-splicing endonuclease subunit Sen34** |  |
| **1** | | 9 | Ace | | ML | MLVAAAAER | Partial-Ac- | 74.17 | Q96S19 | CP013_HUMAN | **UPF0585 protein C16orf13** |  |
| **13. Met-Met-** | | | | |  |  |  |  |  |  |  |  |
| 1 | | 17 | Ace | | MM | MMADGAAAGAGGSPSLR | 100% Ac- | 100.00 | Q8IWC1 | MA7D3_HUMAN | **MAP7 domain-containing protein 3** |  |
| 1 | | 24 | Ace | | MM | MMCGAPSATQPATAETQHIADQVR | N.D | N.D | P04080 | CYTB_HUMAN | **Cystatin-B** |  |
| 1 | | 12 | Ace | | MM | MMDPCSVGVQLR | 100% Ac- | 100.00 | Q8N8R7 | CK046_HUMAN | **Uncharacterized protein C11orf46** |  |
| 1 | | 17 | Ace | | MM | MMEGLDDGPDFLSEEDR | 100% Ac- | 100.00 | Q9UNH7 | SNX6_HUMAN | **Sorting nexin-6** |  |
| 1 | | 10 | AcD3 | | MM | MMGKEEEIAR | Partial-Ac- | 28.57 | Q15560 | TCEA2_HUMAN | **Transcription elongation factor A protein 2** |  |
| 1 | | 11 | Ace | | MM | MMGSKMASASR | 100% Ac- | 100.00 | P82909 | RT36_HUMAN | **28S ribosomal protein S36, mitochondrial** |  |
| 1 | | 10 | Ace | | MM | MMKFKPNQTR | Partial-Ac- | 79.86 | Q9NZJ9 | NUDT4_HUMAN | **Diphosphoinositol polyphosphate phosphohydrolase 2** |  |
| 1 | | 10 | Ace | | MM | MMKLKSNQTR | 100% Ac- | 100.00 | O95989 | NUDT3_HUMAN | **Diphosphoinositol polyphosphate phosphohydrolase 1** |  |
| 1 | | 16 | Ace | | MM | MMLGPEGGEGFVVKLR | 100% Ac- | 100.00 | P52597 | HNRPF_HUMAN | **Heterogeneous nuclear ribonucleoprotein F** |  |
| 1 | | 16 | Ace | | MM | MMLGTEGGEGFVVKVR | 100% Ac- | 100.00 | P31943 | HNRH1_HUMAN | **Heterogeneous nuclear ribonucleoprotein H** |  |
| 1 | | 8 | Ace | | MM | MMLSTEGR | 100% Ac- | 100.00 | P55795 | HNRH2_HUMAN | **Heterogeneous nuclear ribonucleoprotein H2** |  |
| 1 | | 9 | Ace | | MM | MMTSVGTNR | 100% Ac- | 100.00 | Q14157 | UBP2L_HUMAN | **Ubiquitin-associated protein 2-like** |  |
| 1 | | 11 | Ace | | MM | MMVESASETIR | 100% Ac- | 100.00 | Q8IVH2 | FOXP4_HUMAN | **Forkhead box protein P4** |  |
| 1 | | 35 | Ace | | MM | MMAAEAGSEEGGPVTAGAGGGGAAAGSSAYPAVCR | 100% Ac- | 100.00 | P20936 | RASA1_HUMAN | **Ras GTPase-activating protein 1** |  |
| **14. Met-Asn** | | |  | |  |  |  |  |  |  |  |  |
| 1 | | 15 | Ace | | MN | MNAGSDPVVIVSAAR | 100% Ac- | 100.00 | Q9BWD1 | THIC_HUMAN | **Acetyl-CoA acetyltransferase, cytosolic** |  |
| 1 | | 13 | Ace | | MN | MNALLEQKEQQER | 100% Ac- | 100.00 | Q6AI12 | ANR40_HUMAN | **Ankyrin repeat domain-containing protein 40** |  |
| 1 | | 20 | Ace | | MN | MNDFGIKNMDQVAPVANSYR | 100% Ac- | 100.00 | P15036 | ETS2_HUMAN | **Protein C-ets-2** |  |
| 1 | | 8 | Ace | | MN | MNDTVTIR | 100% Ac- | 100.00 | P62847 | RS24_HUMAN | **40S ribosomal protein S24** |  |
| 1 | | 10 | Ace | | MN | MNFLSTAESR | N.D | N.D | Q2KHR3 | QSER1_HUMAN | **Glutamine and serine-rich protein 1** |  |
| 1 | | 8 | Ace | | MN | MNFSGGGR | 100% Ac- | 100.00 | Q86TN4 | TRPT1_HUMAN | **tRA 2'-phosphotransferase 1** |  |
| 1 | | 11 | Ace | | MN | MNGFTPDEMSR | 100% Ac- | 100.00 | O75446 | SAP30_HUMAN | **Histone deacetylase complex subunit SAP30** |  |
| 1 | | 16 | Ace | | MN | MNGPADGEVDYKKKYR | 100% Ac- | 100.00 | Q8NBZ0 | IN80E_HUMAN | **INO80 complex subunit E** |  |
| 1 | | 8 | Ace | | MN | MNILAPVR | 100% Ac- | 100.00 | Q9BZG1 | RAB34_HUMAN | **Ras-related protein Rab-34** |  |
| 1 | | 21 | Ace | | MN | MNIMDFNVKKLAADAGTFLSR | 100% Ac- | 100.00 | Q9Y371 | SHLB1_HUMAN | **Endophilin-B1** |  |
| 1 | | 13 | Ace | | MN | MNKLKSSQKDKVR | 100% Ac- | 100.00 | Q96GG9 | DCNL1_HUMAN | **DC1-like protein 1** |  |
| 1 | | 14 | Ace | | MN | MNLAEICDNAKKGR | 100% Ac- | 100.00 | Q9BW62 | KATL1_HUMAN | **Katanin p60 ATPase-containing subunit A-like 1** |  |
| 1 | | 13 | Ace | | MN | MNLDGSAQDPEKR | 100% Ac- | 100.00 | Q8TAC1 | RFESD_HUMAN | **Rieske domain-containing protein** |  |
| 1 | | 8 | Ace | | MN | MNLFNLDR | 100% Ac- | 100.00 | Q9H4L7 | SMRCD_HUMAN | **SWI/SF-related matrix-associated actin-dependent regulator of chromatin subfamily A containing DEAD/H box 1** |  |
| 1 | | 9 | Ace | | MN | MNLLPKSSR | 100% Ac- | 100.00 | Q8N6R0 | K0859_HUMAN | **Putative methyltransferase KIAA0859** |  |
| 1 | | 13 | Ace | | MN | MNLLPNIESPVTR | 100% Ac- | 100.00 | P17980 | PRS6A_HUMAN | **26S protease regulatory subunit 6A** |  |
| 1 | | 13 | Ace | | MN | MNLQAQPKAQNKR | 100% Ac- | 100.00 | Q6PJG2 | CN043_HUMAN | **Uncharacterized protein C14orf43** |  |
| 1 | | 24 | Ace | | MN | MNNGGKAEKENTPSEANLQEEEVR | 100% Ac- | 100.00 | Q93062 | RBPMS_HUMAN | **RNA-binding protein with multiple splicing** |  |
| 1 | | 15 | Ace | | MN | MNNQKQQKPTLSGQR | 100% Ac- | 100.00 | Q7L1Q6 | BZW1_HUMAN | **Basic leucine zipper and W2 domain-containing protein 1** |  |
| 1 | | 27 | Ace | | MN | MNNSGADEIGKLFVGGLDWSTTQETLR | 100% Ac- | 100.00 | Q96EP5 | DAZP1_HUMAN | **DAZ-associated protein 1** |  |
| 1 | | 16 | Ace | | MN | MNNSLENTISFEEYIR | 100% Ac- | 100.00 | Q2TAL8 | QRIC1_HUMAN | **Glutamine-rich protein 1** |  |
| 1 | | 9 | Ace | | MN | MNPGFDLSR | 100% Ac- | 100.00 | Q8IVH8 | M4K3_HUMAN | **Mitogen-activated protein kinase kinase kinase kinase 3** |  |
| 1 | | 16 | Ace | | MN | MNPLTKVKLINELNER | 100% Ac- | 100.00 | Q9Y388 | RBMX2_HUMAN | **RNA-binding motif protein, X-linked 2** |  |
| 1 | | 14 | Ace | | MN | MNQEKLAKLQAQVR | 100% Ac- | 100.00 | Q96K17 | BT3L4_HUMAN | **Transcription factor BTF3 homolog 4** | P20290 (50-63) |
| 1 | | 15 | Ace | | MN | MNSDQDVALKLAQER | N.D | N.D | Q92738 | US6NL_HUMAN | **USP6 -terminal-like protein** |  |
| 1 | | 10 | Ace | | MN | MNSIKNVPAR | N.D | N.D | O75146 | HIP1R_HUMAN | **Huntingtin-interacting protein 1-related protein** |  |
| 1 | | 20 | Ace | | MN | MNSMKTEENKSFSAMEDDQR | 100% Ac- | 100.00 | Q6SJ93 | F111B_HUMAN | **Protein FAM111B** |  |
| 1 | | 10 | Ace | | MN | MNSPVDPGAR | 100% Ac- | 100.00 | Q8TEU7 | RPGF6_HUMAN | **Rap guanine nucleotide exchange factor 6** |  |
| 1 | | 13 | Ace | | MN | MNSSSANITYASR | N.D | N.D | P35869 | AHR_HUMAN | **Aryl hydrocarbon receptor** |  |
| 1 | | 22 | Ace | | MN | MNSSTSTMSEEPDALSVVNQLR | 100% Ac- | 100.00 | Q9NVT9 | ARMC1_HUMAN | **Armadillo repeat-containing protein 1** |  |
| 1 | | 13 | Ace | | MN | MNSVGEACTDMKR | 100% Ac- | 100.00 | O43715 | TRIA1_HUMAN | **TP53-regulated inhibitor of apoptosis 1** |  |
| 1 | | 15 | Ace | | MN | MNTSIPYQQNPYNPR | 100% Ac- | 100.00 | O94929 | ABLM3_HUMAN | **Actin-binding LIM protein 3** |  |
| 1 | | 10 | Ace | | MN | MNVTPEVKSR | 100% Ac- | 100.00 | Q5T3I0 | GPTC4_HUMAN | **G patch domain-containing protein 4** |  |
| 1 | | 9 | Ace | | MN | MNVTSIALR | 100% Ac- | 100.00 | Q9H9A7 | RMI1_HUMAN | **RecQ-mediated genome instability protein 1** |  |
| 1 | | 12 | Ace | | MN | MNWNKGGPGTKR | N.D | N.D | Q86XP3 | DDX42_HUMAN | **ATP-dependent RA helicase DDX42** |  |
| 1 | | 14 | Ace | | MN | MNYQQQLANSAAIR | 100% Ac- | 100.00 | Q9UPQ3 | AGAP1_HUMAN | **Arf-GAP, GTPase, AK repeat and PH domain-containing protein 1** |  |
| **15. Met-Pro-** | | |  | |  |  |  |  |  |  |  |  |
| 1 | | 14 | AcD3 | | MP | MPFLELDTNLPANR | 100% free | 0.00 | A6NHG4 | DDTL_HUMAN | **D-dopachrome decarboxylase-like protein** | P30046 (1-14) |
| 1 | | 22 | AcD3 | | MP | MPGGLLLGDVAPNFEANTTVGR | 100% free | 0.00 | P30041 | PRDX6_HUMAN | **Peroxiredoxin-6** |  |
| 1 | | 16 | AcD3 | | MP | MPKCPKCNKEVYFAER | 100% free | 0.00 | P50238 | CRIP1_HUMAN | **Cysteine-rich protein 1** |  |
| 1 | | 8 | AcD3 | | MP | MPKTISVR | 100% free | 0.00 | P26038 | MOES_HUMAN | **Moesin** |  |
| 1 | | 12 | AcD3 | | MP | MPMFIVNTNVPR | 100% free | 0.00 | P14174 | MIF_HUMAN | **Macrophage migration inhibitory factor** |  |
| 1 | | 12 | AcD3 | | MP | MPPYTVVYFPVR | 100% free | 0.00 | P09211 | GSTP1_HUMAN | **Glutathione S-transferase P** |  |
| **16. Met-Gln-** | | |  | |  |  |  |  |  |  |  |  |
| 1 | | 20 | Ace | | MQ | MQAFLKGTSISTKPPLTKDR | 100% Ac- | 100.00 | P35249 | RFC4_HUMAN | **Replication factor C subunit 4** |  |
| 1 | | 13 | Ace | | MQ | MQAGKPILYSYFR | 100% Ac- | 100.00 | O43708 | MAAI_HUMAN | **Maleylacetoacetate isomerase** |  |
| 1 | | 16 | Ace | | MQ | MQDAENVAVPEAAEER | 100% Ac- | 100.00 | P55884 | EIF3B_HUMAN | **Eukaryotic translation initiation factor 3 subunit B** |  |
| 1 | | 17 | Ace | | MQ | MQDDSIEASTSISQLLR | N.D | N.D | Q86TC9 | MYPN_HUMAN | **Myopalladin** |  |
| 1 | | 15 | Ace | | MQ | MQDPNADTEWNDILR | 100% Ac- | 100.00 | Q9H2J4 | PDCL3_HUMAN | **Phosducin-like protein 3** |  |
| 1 | | 13 | Ace | | MQ | MQESQTKSMFVSR | 100% Ac- | 100.00 | Q9Y6D5 | BIG2_HUMAN | **Brefeldin A-inhibited guanine nucleotide-exchange protein 2** |  |
| 1 | | 10 | Ace | | MQ | MQKIKSLMTR | 100% Ac- | 100.00 | Q96JG6 | CC132_HUMAN | **Coiled-coil domain-containing protein 132** |  |
| 1 | | 15 | Ace | | MQ | MQKSEGSGGTQLKNR | 100% Ac- | 100.00 | Q8N7B6 | CD028_HUMAN | **Uncharacterized protein C4orf28** |  |
| 1 | | 24 | Ace | | MQ | MQKYEKLEKIGEGTYGTVFKAKNR | N.D | N.D | Q00535 | CDK5_HUMAN | **Cell division protein kinase 5** |  |
| 1 | | 11 | AcD3 | | MQ | MQLTVKALQGR | 100% free | 0.00 | P11441 | UBL4A_HUMAN | **Ubiquitin-like protein 4A** |  |
| 1 | | 15 | Ace | | MQ | MQNDAGEFVDLYVPR | 100% Ac- | 100.00 | P63220 | RS21_HUMAN | **40S ribosomal protein S21** |  |
| 1 | | 11 | Ace | | MQ | MQPASAKWYDR | Partial-Ac- | 79.97 | Q15185 | TEBP_HUMAN | **Prostaglandin E synthase 3** |  |
| 1 | | 42 | AcD3 | | MQ | MQQPQPQGQQQPGPGQQLGGQGAAPGAGGGPGGGPGPGPCLR | Partial-Ac- | 32.75 | Q7Z7E8 | UB2Q1_HUMAN | **Ubiquitin-conjugating enzyme E2 Q1** |  |
| 1 | | 11 | Ace | | MQ | MQSPAVLVTSR | 100% Ac- | 100.00 | Q53T59 | H1BP3_HUMAN | **HCLS1-binding protein 3** |  |
| 1 | | 9 | AcD3 | | MQ | MQTFLKGKR | Partial-Ac- | 20.67 | Q13572 | ITPK1_HUMAN | **Inositol-tetrakisphosphate 1-kinase** |  |
| 1 | | 13 | Ace | | MQ | MQTPVNIPVPVLR | 100% Ac- | 100.00 | Q9H840 | GEMI7_HUMAN | **Gem-associated protein 7** |  |
| 1 | | 10 | Ace | | MQ | MQVAMNGKAR | 100% Ac- | 100.00 | Q9ULA0 | DNPEP_HUMAN | **Aspartyl aminopeptidase** |  |
| 1 | | 10 | Ace | | MQ | MQAALEVTAR | 100% Ac- | 100.00 | Q9BSY4 | CHCH5_HUMAN | **Coiled-coil-helix-coiled-coil-helix domain-containing protein 5** |  |
| **17. Met-Ser-** | | |  | |  |  |  |  |  |  |  |  |
| 1 | | 9 | Ace | | MS | MSELPGDVR | 100% Ac- | 100.00 | Q15527 | SURF2_HUMAN | **Surfeit locus protein 2** |  |
| 1 | | 12 | Ace | | MS | MSIEIESSDVIR | 100% Ac- | 100.00 | Q2TAY7 | SMU1_HUMAN | **WD40 repeat-containing protein SMU1** |  |
| 1 | | 10 | Ace | | MS | MSKNTVSSAR | 100% Ac- | 100.00 | O15511 | ARPC5_HUMAN | **Actin-related protein 2/3 complex subunit 5** |  |
| 1 | | 14 | Ace | | MS | MSKSESPKEPEQLR | 100% Ac- | 100.00 | P09651 | ROA1_HUMAN | **Heterogeneous nuclear ribonucleoprotein A1** | P0C7M2 (1-14) |
| 1 | | 9 | Ace | | MS | MSPEVALNR | Partial-Ac- | 93.97 | Q9NXR7 | BRE_HUMAN | **Protein BRE** |  |
| 1 | | 14 | Ace | | MS | MSPTPPLFSLPEAR | 100% Ac- | 100.00 | Q96FV9 | THOC1_HUMAN | **THO complex subunit 1** |  |
| 1 | | 15 | AcD3 | | MS | MSTGTFVVSQPLNYR | 100% free | 0.00 | P49189 | AL9A1_HUMAN | **4-trimethylaminobutyraldehyde dehydrogenase** |  |
| 1 | | 13 | Ace | | MS | MSTNENANTPAAR | 100% Ac- | 100.00 | P52292 | IMA2_HUMAN | **Importin subunit alpha-2** |  |
| 1 | | 19 | Ace | | MS | MSVPAFIDISEEDQAAELR | 100% Ac- | 100.00 | Q7L2H7 | EIF3M_HUMAN | **Eukaryotic translation initiation factor 3 subunit M** |  |
| **18. Met-Thr-** | | |  | |  |  |  |  |  |  |  |  |
| 1 | | 39 | Ace | | MT | MTAEEMKATESGAQSAPLPMEGVDISPKQDEGVLKVIKR | 100% Ac- | 100.00 | Q02790 | FKBP4_HUMAN | **FK506-binding protein 4** |  |
| 1 | | 9 | Ace | | MT | MTDDKDVLR | 100% Ac- | 100.00 | Q9H1Y0 | ATG5_HUMAN | **Autophagy protein 5** |  |
| 1 | | 9 | Ace | | MT | MTDYGEEQR | 100% Ac- | 100.00 | Q9H446 | RWDD1_HUMAN | **RWD domain-containing protein 1** |  |
| 1 | | 10 | Ace | | MT | MTEESSDVPR | 100% Ac- | 100.00 | Q5VZK9 | LR16A_HUMAN | **Leucine-rich repeat-containing protein 16A** |  |
| 1 | | 11 | Ace | | MT | MTELQSALLLR | 100% Ac- | 100.00 | P62253 | UB2G1_HUMAN | **Ubiquitin-conjugating enzyme E2 G1** |  |
| 1 | | 15 | Ace | | MT | MTENSTSAPAAKPKR | 100% Ac- | 100.00 | P07305 | H10_HUMAN | **Histone H1.0** |  |
| 1 | | 8 | Ace | | MT | MTEQMTLR | 100% Ac- | 100.00 | P63244 | GBLP_HUMAN | **Guanine nucleotide-binding protein subunit beta-2-like 1** |  |
| 1 | | 16 | Ace | | MT | MTEVVPSSALSEVSLR | N.D | N.D | Q9H7X0 | NAT15_HUMAN | **N-acetyltransferase 15** |  |
| 1 | | 11 | Ace | | MT | MTGKSVKDVDR | 100% Ac- | 100.00 | Q8WU79 | SMAP2_HUMAN | **Stromal membrane-associated protein 2** |  |
| 1 | | 11 | AcD3 | | MT | MTKGTSSFGKR | Partial-Ac- | 34.09 | P61927 | RL37_HUMAN | **60S ribosomal protein L37** |  |
| 1 | | 15 | Ace | | MT | MTLEEFSAGEQKTER | N.D | N.D | P24522 | GA45A_HUMAN | **Growth arrest and DA-damage-inducible protein GADD45 alpha** |  |
| 1 | | 20 | Ace | | MT | MTMDKSELVQKAKLAEQAER | 100% Ac- | 100.00 | P31946 | 1433B_HUMAN | **14-3-3 protein beta/alpha** |  |
| 1 | | 12 | Ace | | MT | MTNEEPLPKKVR | 100% Ac- | 100.00 | Q15007 | FL2D_HUMAN | **Pre-mRA-splicing regulator WTAP** |  |
| 1 | | 8 | AcD3 | | MT | MTNTKGKR | 100% free | 0.00 | P46778 | RL21_HUMAN | **60S ribosomal protein L21** |  |
| 1 | | 24 | Ace | | MT | MTQAEKGDTENGKEKGGEKEKEQR | 100% Ac- | 100.00 | Q9H981 | ARP8_HUMAN | **Actin-related protein 8** |  |
| 1 | | 11 | AcD3 | | MT | MTSALENYINR | Partial-Ac- | 43.77 | O95777 | LSM8_HUMAN | **U6 snRA-associated Sm-like protein LSm8** |  |
| 1 | | 17 | Ace | | MT | MTSDQDAKVVAEPQTQR | 100% Ac- | 100.00 | Q92615 | LARP5_HUMAN | **La-related protein 5** |  |
| 1 | | 10 | Ace | | MT | MTSLAQQLQR | Partial-Ac- | 86.57 | Q9H583 | HEAT1_HUMAN | **HEAT repeat-containing protein 1** |  |
| 1 | | 9 | Ace | | MT | MTSMTQSLR | 100% Ac- | 100.00 | Q9NPJ3 | THEM2_HUMAN | **Thioesterase superfamily member 2** |  |
| 1 | | 10 | Ace | | MT | MTTASTSQVR | 100% Ac- | 100.00 | P02794 | FRIH_HUMAN | **Ferritin heavy chain** |  |
| 1 | | 31 | Ace | | MT | MTTDEGAKNNEESPTATVAEQGEDITSKKDR | 100% Ac- | 100.00 | Q13451 | FKBP5_HUMAN | **FK506-binding protein 5** |  |
| **19. Met- Val-** | | | | |  |  |  |  |  |  |  |  |
| **1** | | 12 | AcD3 | | MV | MVDYYEVLGVQR | 100% free | 0.00 | O75190 | DNJB6_HUMAN | **DnaJ homolog subfamily B member 6** |  |
| **1** | | 8 | Ace | | MV | MVEEENIR | 100% Ac- | 100.00 | Q8IWA0 | WDR75_HUMAN | **WD repeat-containing protein 75** |  |
| **1** | | 21 | Ace | | MV | MVEKEEAGGGISEEEAAQYDR | 100% Ac- | 100.00 | Q9UBE0 | SAE1_HUMAN | **SUMO-activating enzyme subunit 1** |  |
| **2** | | 11 | Ace | | MV | MVESASETIR | 100% Ac- | 100.00 | Q8IVH2 | FOXP4_HUMAN | **Forkhead box protein P4** |  |
| **1** | | 10 | Ace | | MV | MVGEEKMSLR | 100% Ac- | 100.00 | P35610 | SOAT1_HUMAN | **Sterol O-acyltransferase 1** |  |
| **1** | | 18 | Ace | | MV | MVGGEAAAAVEELVSGVR | 100% Ac- | 100.00 | Q96HQ2 | C2AIL_HUMAN | **CDK2AIP -terminal-like protein** |  |
| **1** | | 22 | Ace | | MV | MVGGGGVGGGLLENANPLIYQR | Partial-Ac- | 71.23 | Q9Y3D0 | FA96B_HUMAN | **UPF0195 protein FAM96B** |  |
| **1** | | 23 | AcD3 | | MV | MVGVKPVGSDPDFQPELSGAGSR | Partial-Ac- | 23.79 | O43396 | TXNL1_HUMAN | **Thioredoxin-like protein 1** |  |
| **1** | | 8 | AcD3 | | MV | MVLADLGR | Partial-Ac- | 37.12 | P61011 | SRP54_HUMAN | **Signal recognition particle 54 kDa protein** |  |
| **1** | | 9 | Ace | | MV | MVLDLDLFR | Partial-Ac- | 65.69 | P49591 | SYSC_HUMAN | **Seryl-tRA synthetase, cytoplasmic** |  |
| **1** | | 13 | AcD3 | | MV | MVLESVVADLLNR | 100% free | 0.00 | Q709C8 | VP13C_HUMAN | **Vacuolar protein sorting-associated protein 13C** |  |
| **1** | | 8 | AcD3 | | MV | MVLIKEFR | Partial-Ac- | 36.30 | P48739 | PIPNB_HUMAN | **Phosphatidylinositol transfer protein beta isoform** |  |
| **1** | | 15 | AcD3 | | MV | MVLLESEQFLTELTR | 100% free | 0.00 | P37108 | SRP14_HUMAN | **Signal recognition particle 14 kDa protein** |  |
| **1** | | 8 | Ace | | MV | MVLLKEYR | Partial-Ac- | 55.79 | Q00169 | PIPNA_HUMAN | **Phosphatidylinositol transfer protein alpha isoform** |  |
| **1** | | 9 | AcD3 | | MV | MVLSELAAR | Partial-Ac- | 30.09 | Q8TB03 | CX038_HUMAN | **Uncharacterized protein CXorf38** |  |
| **1** | | 13 | Ace | | MV | MVMEKPSPLLVGR | 100% Ac- | 100.00 | Q13283 | G3BP1_HUMAN | **Ras GTPase-activating protein-binding protein 1** | Q9UN86 (1-13) |
| **1** | | 10 | AcD3 | | MV | MVNFTVDQIR | Partial-Ac- | 32.20 | P13639 | EF2_HUMAN | **Elongation factor 2** |  |
| **1** | | 19 | Ace | | MV | MVNPTVFFDIAVDGEPLGR | Partial-Ac- | 77.63 | P62937 | PPIA_HUMAN | **Peptidyl-prolyl cis-trans isomerase A** |  |
| **1** | | 8 | AcD3 | | MV | MVNVPKTR | 100% free | 0.00 | P83881 | RL36A_HUMAN | **60S ribosomal protein L36a** | Q969Q0 (1-8) |
| **1** | | 10 | AcD3 | | MV | MVPGSEGPAR | Partial-Ac- | 26.14 | Q71SY5 | MED25_HUMAN | **Mediator of RA polymerase II transcription subunit 25** |  |
| **1** | | 24 | Ace | | MV | MVTEQEVDAIGQTLVDPKQPLQAR | 100% Ac- | 100.00 | Q9BU89 | DOHH_HUMAN | **Deoxyhypusine hydroxylase** |  |
| **1** | | 13 | Ace | | MV | MVVSKMNKDAQMR | 100% Ac- | 100.00 | Q9NPA8 | ENY2_HUMAN | **Enhancer of yellow 2 transcription factor homolog** |  |
| **1** | | 17 | Ace | | MV | MVAAKKTKKSLESINSR | 100% Ac- | 100.00 | P62888 | RL30_HUMAN | **60S ribosomal protein L30** |  |
| **20. Met-Tyr** | | |  | |  |  |  |  |  |  |  |  |
| 1 | | 18 | Ace | | MY | MYAKGGKGSAVPSDSQAR | 100% Ac- | 100.00 | Q9BWG4 | SSBP4_HUMAN | **Single-stranded DA-binding protein 4** |  |
| 1 | | 14 | Ace | | MY | MYEGKKTKNMFLTR | N.D | N.D | Q9Y6D6 | BIG1_HUMAN | **Brefeldin A-inhibited guanine nucleotide-exchange protein 1** |  |
| 1 | | 11 | Ace | | MY | MYNGIGLPTPR | 100% Ac- | 100.00 | Q9UQ35 | SRRM2_HUMAN | **Serine/arginine repetitive matrix protein 2** |  |
| 1 | | 12 | AcD3 | | MY | MYPESTTGSPAR | Partial-Ac- | 40.79 | Q13303 | KCAB2_HUMAN | **Voltage-gated potassium channel subunit beta-2** |  |
| 1 | | 14 | Ace | | MY | MYQDYPGNFDTSSR | N.D | N.D | P15408 | FOSL2_HUMAN | **Fos-related antigen 2** |  |
| 1 | | 13 | Ace | | MY | MYSLNQEIKAFSR | N.D | N.D | Q8WTR2 | DUS19_HUMAN | **Dual specificity protein phosphatase 19** |  |
| 1 | | 15 | Ace | | MY | MYTITKGPSKLVAQR | 100% Ac- | 100.00 | Q9BUT9 | CP014_HUMAN | **Uncharacterized protein C16orf14** |  |
| **21. Met-Tyr** | | |  | |  |  |  |  |  |  |  |  |
| 2 | | 18 | AcD3 | | PA | PAPEQASLVEEGQPQTR | 100% free | 0.00 | Q99569 | PKP4_HUMAN | **Plakophilin-4** |  |
| 2 | | 10 | AcD3 | | PA | PAPAATYER | 100% free | 0.00 | O95059 | RPP14_HUMAN | **Ribonuclease P protein subunit p14** |  |
| 2 | | 20 | AcD3 | | PA | PAVDKLLLEEALQDSPQTR | 100% free | 0.00 | Q8NEU8 | DP13B_HUMAN | **DCC-interacting protein 13-beta** |  |
| 2 | | 20 | AcD3 | | PA | PAVLGFEGSANKIGVGVVR | 100% free | 0.00 | Q9NPF4 | OSGEP_HUMAN | **Probable O-sialoglycoprotein endopeptidase** |  |
| 2 | | 11 | AcD3 | | PA | PAVSKGDGMR | 100% free | 0.00 | O94973 | AP2A2_HUMAN | **AP-2 complex subunit alpha-2** | O95782 (2-11) |
| 2 | | 16 | AcD3 | | PA | PAVSLPPKENALFKR | 100% free | 0.00 | Q9BXJ9 | NARG1_HUMAN | **MDA receptor-regulated protein 1** |  |
| 2 | | 25 | AcD3 | | PA | PAYHSSLMDPDTKLIGNMALLPIR | 100% free | 0.00 | O15145 | ARPC3_HUMAN | **Actin-related protein 2/3 complex subunit 3** |  |
| 2 | | 30 | AcD3 | | PD | PDPAKSAPAPKKGSKKAVTKAQKKDGKKR | 100% free | 0.00 | P23527 | H2B1O_HUMAN | **Histone H2B type 1-O** | Q93079 (2-30) |
| 2 | | 30 | AcD3 | | PD | PDPAKSAPAPKKGSKKAVTKVQKKDGKKR | N.D | N.D | Q5QNW6 | H2B2F_HUMAN | **Histone H2B type 2-F** |  |
| 2 | | 30 | AcD3 | | PD | PDPSKSAPAPKKGSKKAVTKAQKKDGKKR | 100% free | 0.00 | Q8N257 | H2B3B_HUMAN | **Histone H2B type 3-B** |  |
| 2 | | 9 | AcD3 | | PD | PDSNFAER | 100% free | 0.00 | Q9UJ83 | HACL1_HUMAN | **2-hydroxyacyl-CoA lyase 1** |  |
| 2 | | 10 | AcD3 | | PD | PDYLGADQR | 100% free | 0.00 | P35998 | PRS7_HUMAN | **26S protease regulatory subunit 7** |  |
| 2 | | 18 | AcD3 | | PE | PEIVDTCSLASPASVCR | 100% free | 0.00 | P09960 | LKHA4_HUMAN | **Leukotriene A-4 hydrolase** |  |
| 2 | | 30 | AcD3 | | PE | PELAKSAPAPKKGSKKAVTKAQKKDGKKR | 100% free | 0.00 | Q99880 | H2B1L_HUMAN | **Histone H2B type 1-L** |  |
| 2 | | 18 | AcD3 | | PE | PENPATDKLQVLQVLDR | 100% free | 0.00 | Q8NI35 | INADL_HUMAN | **InaD-like protein** |  |
| 2 | | 8 | AcD3 | | PE | PENVAPR | 100% free | 0.00 | P50991 | TCPD_HUMAN | **T-complex protein 1 subunit delta** |  |
| 2 | | 30 | AcD3 | | PE | PEPAKSAPAPKKGSKKAVTKAQKKDGKKR | 100% free | 0.00 | O60814 | H2B1K_HUMAN | **Histone H2B type 1-K** | P06899 (2-30)^AP62807 (2-30)^AQ16778 (2-30) |
| 2 | | 30 | AcD3 | | PE | PEPSKSAPAPKKGSKKAVTKAQKKDGKKR | 100% free | 0.00 | Q99877 | H2B1N_HUMAN | **Histone H2B type 1-N** |  |
| 2 | | 30 | AcD3 | | PE | PEPVKSAPVPKKGSKKAINKAQKKDGKKR | 100% free | 0.00 | Q99879 | H2B1M_HUMAN | **Histone H2B type 1-M** |  |
| 2 | | 14 | AcD3 | | PF | PFAEDKTYKYICR | 100% free | 0.00 | Q7Z434 | MAVS_HUMAN | **Mitochondrial antiviral-signaling protein** |  |
| 2 | | 9 | AcD3 | | PF | PFLDIQKR | 100% free | 0.00 | O43920 | NDUS5_HUMAN | **NADH dehydrogenase [ubiquinone] iron-sulfur protein 5** |  |
| 2 | | 14 | AcD3 | | PF | PFLELDTNLPANR | 100% free | 0.00 | A6NHG4 | DDTL_HUMAN | **D-dopachrome decarboxylase-like protein** | P30046 (2-14) |
| 2 | | 22 | AcD3 | | PG | PGGLLLGDVAPNFEANTTVGR | 100% free | 0.00 | P30041 | PRDX6_HUMAN | **Peroxiredoxin-6** |  |
| 2 | | 20 | AcD3 | | PG | PGIDKLPIEETLEDSPQTR | 100% free | 0.00 | Q9UKG1 | DP13A_HUMAN | **DCC-interacting protein 13-alpha** |  |
| 2 | | 7 | AcD3 | | PG | PGLSCR | 100% free | 0.00 | P05198 | IF2A_HUMAN | **Eukaryotic translation initiation factor 2 subunit 1** |  |
| 2 | | 16 | AcD3 | | PG | PGPTPSGTNVGSSGR | 100% free | 0.00 | P60468 | SC61B_HUMAN | **Protein transport protein Sec61 subunit beta** |  |
| 2 | | 30 | AcD3 | | PG | PGPTQTLSPNGENNNDIIQDNNGTIIPFR | 100% free | 0.00 | Q15555 | MARE2_HUMAN | **Microtubule-associated protein RP/EB family member 2** |  |
| 2 | | 14 | AcD3 | | PG | PGSAAKGSELSER | 100% free | 0.00 | P49770 | EI2BB_HUMAN | **Translation initiation factor eIF-2B subunit beta** |  |
| 2 | | 16 | AcD3 | | PG | PGVTVKDVNQQEFVR | 100% free | 0.00 | P39019 | RS19_HUMAN | **40S ribosomal protein S19** |  |
| 2 | | 20 | AcD3 | | PI | PINKSEKPESCDNVKVVVR | N.D | N.D | Q9Y496 | KIF3A_HUMAN | **Kinesin-like protein KIF3A** |  |
| 2 | | 9 | AcD3 | | PK | PKAKGKTR | 100% free | 0.00 | Q9Y3C1 | NOP16_HUMAN | **Nucleolar protein 16** |  |
| 2 | | 12 | AcD3 | | PK | PKAPKGKSAGR | 100% free | 0.00 | Q96EY4 | CD043_HUMAN | **UPF0534 protein C4orf43** |  |
| 2 | | 16 | AcD3 | | PK | PKCPKCNKEVYFAER | 100% free | 0.00 | P50238 | CRIP1_HUMAN | **Cysteine-rich protein 1** |  |
| 2 | | 8 | AcD3 | | PK | PKFKAAR | 100% free | 0.00 | Q13895 | BYST_HUMAN | **Bystin** |  |
| 2 | | 35 | AcD3 | | PK | PKGKKAKGKKVAPAPAVVKKQEAKKVVNPLFEKR | 100% free | 0.00 | P62424 | RL7A_HUMAN | **60S ribosomal protein L7a** |  |
| 2 | | 16 | AcD3 | | PK | PKKFQGENTKSAAAR | 100% free | 0.00 | Q96CT7 | CC124_HUMAN | **Coiled-coil domain-containing protein 124** |  |
| 2 | | 47 | AcD3 | | PK | PKKKPTPIQLNPAPDGSAVNGTSSAETNLEALQKKLEELELDEQQR | 100% free | 0.00 | Q02750 | MP2K1_HUMAN | **Dual specificity mitogen-activated protein kinase kinase 1** |  |
| 2 | | 9 | AcD3 | | PK | PKKKTGAR | 100% free | 0.00 | Q9Y3S2 | ZN330_HUMAN | **Zinc finger protein 330** |  |
| 2 | | 12 | AcD3 | | PK | PKNKGKGGKNR | 100% free | 0.00 | O14602 | IF1AY_HUMAN | **Eukaryotic translation initiation factor 1A, Y-chromosomal** | P47813 (2-12) |
| 2 | | 7 | AcD3 | | PK | PKNKKR | 100% free | 0.00 | O00458 | IFRD1_HUMAN | **Interferon-related developmental regulator 1** | **Q9BX63 (574-579)** |
| 2 | | 10 | AcD3 | | PK | PKNSKVVKR | N.D | N.D | Q9H2J7 | S6A15_HUMAN | **Orphan sodium- and chloride-dependent neurotransmitter transporter TT73** |  |
| 2 | | 8 | AcD3 | | PK | PKPINVR | 100% free | 0.00 | P15311 | EZRI_HUMAN | **Ezrin** | P35241 (2-8) |
| 2 | | 27 | AcD3 | | PK | PKSKELVSSSSSGSDSDSEVDKKLKR | 100% free | 0.00 | P53999 | TCP4_HUMAN | **Activated RA polymerase II transcriptional coactivator p15** |  |
| 2 | | 8 | AcD3 | | PK | PKTISVR | 100% free | 0.00 | P26038 | MOES_HUMAN | **Moesin** |  |
| 2 | | 11 | AcD3 | | PK | PKVKSGAIGR | 100% free | 0.00 | Q9UNQ2 | DIMT1_HUMAN | **Probable dimethyladenosine transferase** |  |
| 2 | | 11 | AcD3 | | PL | PLELELCPGR | 100% free | 0.00 | Q9NR45 | SIAS_HUMAN | **Sialic acid synthase** |  |
| 2 | | 18 | AcD3 | | PL | PLENLEEEGLPKNPDLR | 100% free | 0.00 | Q15008 | PSMD6_HUMAN | **26S proteasome non-ATPase regulatory subunit 6** |  |
| 2 | | 25 | AcD3 | | PL | PLLTQQIQDEDDQYSLVASLDNVR | 100% free | 0.00 | O60671 | RAD1_HUMAN | **Cell cycle checkpoint protein RAD1** |  |
| 2 | | 8 | AcD3 | | PL | PLLVEGR | 100% free | 0.00 | Q96AB6 | NTAN1_HUMAN | **Protein -terminal asparagine amidohydrolase** | Q9Y493 (2370-2376) |
| 2 | | 10 | AcD3 | | PL | PLNVSFTNR | 100% free | 0.00 | P01106 | MYC_HUMAN | **Myc proto-oncogene protein** |  |
| 2 | | 10 | AcD3 | | PL | PLPVALQTR | 100% free | 0.00 | O60828 | PQBP1_HUMAN | **Polyglutamine-binding protein 1** |  |
| 2 | | 10 | AcD3 | | PL | PLTGVEPAR | 100% free | 0.00 | Q6P1X5 | TAF2_HUMAN | **Transcription initiation factor TFIID subunit 2** |  |
| 2 | | 16 | AcD3 | | PL | PLVVFCGLPYSGKSR | 100% free | 0.00 | Q96EK9 | KTI12_HUMAN | **Protein KTI12 homolog** |  |
| 2 | | 12 | AcD3 | | PM | PMFIVNTNVPR | 100% free | 0.00 | P14174 | MIF_HUMAN | **Macrophage migration inhibitory factor** |  |
| 2 | | 12 | AcD3 | | PN | PNFCAAPNCTR | 100% free | 0.00 | O43422 | P52K_HUMAN | **52 kDa repressor of the inhibitor of the protein kinase** |  |
| 2 | | 12 | AcD3 | | PN | PNFSGNWKIIR | N.D | N.D | P29373 | RABP2_HUMAN | **Cellular retinoic acid-binding protein 2** |  |
| 2 | | 11 | AcD3 | | PN | PNPKNSKGGR | 100% free | 0.00 | Q9UBI9 | HDC_HUMAN | **Headcase protein homolog** |  |
| 2 | | 24 | AcD3 | | PN | PNSEPASLLELFNSIATQGELVR | 100% free | 0.00 | P23381 | SYWC_HUMAN | **Tryptophanyl-tRA synthetase, cytoplasmic** |  |
| 2 | | 16 | AcD3 | | PN | PNVLLPPKESNLFKR | N.D | N.D | Q6N069 | NARGL_HUMAN | **MDA receptor-regulated 1-like protein** |  |
| 2 | | 11 | AcD3 | | PP | PPKVTSELLR | 100% free | 0.00 | Q9NQW7 | XPP1_HUMAN | **Xaa-Pro aminopeptidase 1** |  |
| 2 | | 12 | AcD3 | | PP | PPYTVVYFPVR | 100% free | 0.00 | P09211 | GSTP1_HUMAN | **Glutathione S-transferase P** |  |
| 2 | | 12 | AcD3 | | PQ | PQYQTWEEFSR | 100% free | 0.00 | P49458 | SRP09_HUMAN | **Signal recognition particle 9 kDa protein** |  |
| 2 | | 20 | AcD3 | | PS | PSDLAKKKAAKKKEAAKAR | 100% free | 0.00 | Q9UG63 | ABCF2_HUMAN | **ATP-binding cassette sub-family F member 2** |  |
| 2 | | 10 | AcD3 | | PS | PSEKTFKQR | 100% free | 0.00 | A6NCE7 | MP3B2_HUMAN | **Microtubule-associated proteins 1A/1B light chain 3 beta 2** | Q9GZQ8 (2-10) |
| 2 | | 15 | AcD3 | | PS | PSKGPLQSVQVFGR | 100% free | 0.00 | P62249 | RS16_HUMAN | **40S ribosomal protein S16** |  |
| 2 | | 11 | AcD3 | | PS | PSKKKKYNAR | 100% free | 0.00 | Q14919 | NC2A_HUMAN | **Dr1-associated corepressor** |  |
| 2 | | 23 | AcD3 | | PS | PSPQLLVLFGSQTGTAQDVSER | N.D | N.D | Q9UHB4 | NDOR1_HUMAN | **ADPH-dependent diflavin oxidoreductase 1** |  |
| 2 | | 11 | AcD3 | | PS | PSSLFADLER | N.D | N.D | Q6ZN04 | MEX3B_HUMAN | **RNA-binding protein MEX3B** |  |
| 2 | | 28 | AcD3 | | PS | PSSLLGAAMPASTSAAALQEALENAGR | 100% free | 0.00 | O75694 | NU155_HUMAN | **Nuclear pore complex protein up155** |  |
| 2 | | 19 | AcD3 | | PS | PSSTSPDQGDDLENCILR | N.D | N.D | Q5T7W7 | CI097_HUMAN | **Uncharacterized protein C9orf97** |  |
| 2 | | 15 | AcD3 | | PT | PTAESEAKVKTKVR | N.D | N.D | Q8N157 | AHI1_HUMAN | **Jouberin** |  |
| 2 | | 32 | AcD3 | | PT | PTTQQSPQDEQEKLLDEAIQAVKVQSFQMKR | 100% free | 0.00 | Q96QK1 | VPS35_HUMAN | **Vacuolar protein sorting-associated protein 35** |  |
| 2 | | 9 | AcD3 | | PT | PTVEELYR | 100% free | 0.00 | Q9BZZ5 | API5_HUMAN | **Apoptosis inhibitor 5** |  |
| 2 | | 8 | AcD3 | | PT | PTVSVKR | 100% free | 0.00 | Q9NSD9 | SYFB_HUMAN | **Phenylalanyl-tRA synthetase beta chain** |  |
| 2 | | 10 | AcD3 | | PV | PVAGSELPR | 100% free | 0.00 | P04818 | TYSY_HUMAN | **Thymidylate synthase** |  |
| 2 | | 24 | AcD3 | | PV | PVTEKDLAEDAPWKKIQQNTFTR | 100% free | 0.00 | O75369 | FLNB_HUMAN | **Filamin-B** |  |
| 2 | | 15 | AcD3 | | PV | PVWGGGNKCGACGR | N.D | N.D | Q16527 | CSRP2_HUMAN | **Cysteine and glycine-rich protein 2** |  |
| 2 | | 10 | AcD3 | | PY | PYANQPTVR | 100% free | 0.00 | P19387 | RPB3_HUMAN | **DNA-directed RA polymerase II subunit RPB3** |  |
| 2 | | 17 | AcD3 | | PY | PYEIKKVFASLPQVER | 100% free | 0.00 | O75083 | WDR1_HUMAN | **WD repeat-containing protein 1** |  |
| 2 | | 22 | AcD3 | | PY | PYQYPALTPEQKKELSDIAHR | 100% free | 0.00 | P04075 | ALDOA_HUMAN | **Fructose-bisphosphate aldolase A** |  |
| **22. Ser-** | | |  | |  |  |  |  |  |  |  |  |
| 2 | | 28 | Ace | | SA | SADGAEADGSTQVTVEEPVQQPSVVDR | 100% Ac- | 100.00 | O60664 | M6PBP_HUMAN | **Mannose-6-phosphate receptor-binding protein 1** |  |
| 2 | | 13 | Ace | | SA | SADAAAGAPLPR | 100% Ac- | 100.00 | O14745 | NHERF_HUMAN | **Ezrin-radixin-moesin-binding phosphoprotein 50** |  |
| 2 | | 10 | Ace | | SA | SAEEMVQIR | 100% Ac- | 100.00 | Q9P2K6 | KLDC5_HUMAN | **Kelch domain-containing protein 5** |  |
| 2 | | 9 | Ace | | SA | SAEGYQYR | 100% Ac- | 100.00 | P27986 | P85A_HUMAN | **Phosphatidylinositol 3-kinase regulatory subunit alpha** |  |
| 2 | | 29 | Ace | | SA | SAEVETSEGVDESEKKNSGALEKENQMR | 100% Ac- | 100.00 | P30519 | HMOX2_HUMAN | **Heme oxygenase 2** |  |
| 2 | | 35 | AcD3 | | SA | SAEVPEAASAEEQKEMEDKVTSPEKAEEAKLKAR | 100% free | 0.00 | P56211 | ARP19_HUMAN | **cAMP-regulated phosphoprotein 19** |  |
| 2 | | 32 | Ace | | SA | SAGGPCPAAAGGGPGGASCSVGAPGGVSMFR | 100% Ac- | 100.00 | Q9HD26 | GOPC_HUMAN | **Golgi-associated PDZ and coiled-coil motif-containing protein** |  |
| 2 | | 15 | Ace | | SA | SAIPAEESDQLLIR | 100% Ac- | 100.00 | Q9UKF6 | CPSF3_HUMAN | **Cleavage and polyadenylation specificity factor subunit 3** |  |
| 2 | | 28 | Ace | | SA | SAKAISEQTGKELLYKFICTTSAIQNR | 100% Ac- | 100.00 | P53396 | ACLY_HUMAN | **ATP-citrate synthase** |  |
| 2 | | 15 | Ace | | SA | SANEDQEMELEALR | 100% Ac- | 100.00 | Q6NW29 | RWDD4_HUMAN | **RWD domain-containing protein 4A** |  |
| 2 | | 8 | Ace | | SA | SAPFEER | 100% Ac- | 100.00 | Q8WVJ2 | NUDC2_HUMAN | **NudC domain-containing protein 2** |  |
| 2 | | 12 | Ace | | SA | SAPSEEEEYAR | N.D | N.D | Q8TD16 | BICD2_HUMAN | **Protein bicaudal D homolog 2** |  |
| 2 | | 13 | Ace | | SA | SAQGDCEFLVQR | 100% Ac- | 100.00 | Q9NVR2 | INT10_HUMAN | **Integrator complex subunit 10** |  |
| 2 | | 25 | Ace | | SA | SAQSVEEDSILIIPTPDEEEKILR | 100% Ac- | 100.00 | P17028 | ZNF24_HUMAN | **Zinc finger protein 24** |  |
| 2 | | 29 | Ace | | SA | SASAPAAEGEGTPTQPASEKEPEMPGPR | 100% Ac- | 100.00 | P35659 | DEK_HUMAN | **Protein DEK** |  |
| 2 | | 10 | Ace | | SA | SASSLLEQR | 100% Ac- | 100.00 | Q9Y5A9 | YTHD2_HUMAN | **YTH domain family protein 2** |  |
| 2 | | 11 | Ace | | SA | SASVVSVISR | Partial-Ac- | 80.83 | P61803 | DAD1_HUMAN | **Dolichyl-diphosphooligosaccharide--protein glycosyltransferase subunit DAD1** |  |
| 2 | | 9 | Ace | | SA | SATSVDQR | 100% Ac- | 100.00 | Q7Z739 | YTHD3_HUMAN | **YTH domain family protein 3** |  |
| 2 | | 10 | Ace | | SA | SATSVDTQR | 100% Ac- | 100.00 | Q9BYJ9 | YTHD1_HUMAN | **YTH domain family protein 1** |  |
| 2 | | 30 | Ace | | SA | SATVVDAVNAAPLSGSKEMSLEEPKKMTR | 100% Ac- | 100.00 | O95391 | SLU7_HUMAN | **Pre-mRA-splicing factor SLU7** |  |
| 2 | | 18 | Ace | | SC | SCINLPTVLPGSPSKTR | 100% Ac- | 100.00 | P04183 | KITH_HUMAN | **Thymidine kinase, cytosolic** |  |
| 2 | | 18 | Ace | | SC | SCTIEKALADAKALVER | 100% Ac- | 100.00 | Q9NVK5 | FGOP2_HUMAN | **FGFR1 oncogene partner 2** |  |
| 3 | | 19 | Ace | | SD | SDEKNLGVSQKLVSPSR | 100% Ac- | 100.00 | Q9H4L5 | OSBL3_HUMAN | **Oxysterol-binding protein-related protein 3** |  |
| 2 | | 9 | Ace | | SD | SDFDEFER | 100% Ac- | 100.00 | P26368 | U2AF2_HUMAN | **Splicing factor U2AF 65 kDa subunit** |  |
| 2 | | 28 | Ace | | SD | SDFDSNPFADPDLNNPFKDPSVTQVTR | 100% Ac- | 100.00 | O15126 | SCAM1_HUMAN | **Secretory carrier-associated membrane protein 1** |  |
| 2 | | 7 | Ace | | SD | SDGFDR | 100% Ac- | 100.00 | Q9H074 | PAIP1_HUMAN | **Polyadenylate-binding protein-interacting protein 1** |  |
| 2 | | 50 | AcD3 | | SD | SDKDDIETPLLTEAAPILEDGNCEPAKNSESVDQGAKPESKSEPVVSTR |  | 43.98 | Q9NRY5 | F1142_HUMAN | **Protein FAM114A2** |  |
| 2 | | 19 | Ace | | SD | SDKLPYKVADIGLAAWGR | 100% Ac- | 100.00 | P23526 | SAHH_HUMAN | **Adenosylhomocysteinase** |  |
| 2 | | 15 | Ace | | SD | SDKPDLSEVEKFDR | 100% Ac- | 100.00 | Q99406 | TYBN_HUMAN | **B thymosin beta** |  |
| 2 | | 13 | Ace | | SD | SDKSELKAELER | 100% Ac- | 100.00 | Q13409 | DC1I2_HUMAN | **Cytoplasmic dynein 1 intermediate chain 2** |  |
| 2 | | 17 | Ace | | SD | SDNGELEDKPPAPPVR | Partial-Ac- | 86.94 | Q13177 | PAK2_HUMAN | **Serine/threonine-protein kinase PAK 2** |  |
| 2 | | 14 | Ace | | SD | SDQQLDCALDLMR | 100% Ac- | 100.00 | P47756 | CAPZB_HUMAN | **F-actin-capping protein subunit beta** |  |
| 2 | | 15 | Ace | | SD | SDSEKLNLDSIIGR | Partial-Ac- | 94.95 | P62136 | PP1A_HUMAN | **Serine/threonine-protein phosphatase PP1-alpha catalytic subunit** |  |
| 2 | | 12 | Ace | | SD | SDSGEQNYGER | 100% Ac- | 100.00 | P62995 | TRA2B_HUMAN | **Splicing factor, arginine/serine-rich 10** |  |
| 2 | | 10 | Ace | | SD | SDTAVADTR | 100% Ac- | 100.00 | Q9UMY4 | SNX12_HUMAN | **Sorting nexin-12** |  |
| 2 | | 12 | Ace | | SD | SDTLTADVIGR | 100% Ac- | 100.00 | Q15813 | TBCE_HUMAN | **Tubulin-specific chaperone E** |  |
| 2 | | 13 | Ace | | SD | SDTSESGAGLTR | 100% Ac- | 100.00 | Q9UNF1 | MAGD2_HUMAN | **Melanoma-associated antigen D2** |  |
| 2 | | 12 | Ace | | SD | SDVEENNFEGR | 100% Ac- | 100.00 | Q13595 | TRA2A_HUMAN | **Transformer-2 protein homolog** |  |
| 2 | | 40 | Ace | | SD | SDYSTGGPPPGPPPPAGGGGGAGGAGGGPPPGPPGAGDR | 100% Ac- | 100.00 | Q92945 | FUBP2_HUMAN | **Far upstream element-binding protein 2** |  |
| 2 | | 31 | Ace | | SD | SDAAVDTSSEITTKDLKEKKEVVEEAENGR | 100% Ac- | 100.00 | P06454 | PTMA_HUMAN | **Prothymosin alpha** |  |
| 2 | | 8 | Ace | | SE | SEADGLR | 100% Ac- | 100.00 | Q9HDC9 | APMAP_HUMAN | **Adipocyte plasma membrane-associated protein** |  |
| 2 | | 7 | Ace | | SE | SEAYFR | 100% Ac- | 100.00 | Q9BRX5 | PSF3_HUMAN | **DNA replication complex GIS protein PSF3** | Q9Y6N5 (212-217) |
| 2 | | 11 | Ace | | SE | SEDEEKVKLR | 100% Ac- | 100.00 | P56962 | STX17_HUMAN | **Syntaxin-17** |  |
| 2 | | 22 | Ace | | SE | SEEIITPVYCTGVSAQVQKQR | 100% Ac- | 100.00 | P07384 | CAN1_HUMAN | **Calpain-1 catalytic subunit** |  |
| 2 | | 9 | Ace | | SE | SEESDSLR | 100% Ac- | 100.00 | Q9BTX7 | TTPAL_HUMAN | **Alpha-tocopherol transfer protein-like** |  |
| 2 | | 16 | Ace | | SE | SEETATSDNDNSYAR | 100% Ac- | 100.00 | Q7Z699 | SPRE1_HUMAN | **Sprouty-related, EVH1 domain-containing protein 1** |  |
| 2 | | 13 | Ace | | SE | SEKKLETTAQQR | 100% Ac- | 100.00 | Q14191 | WRN_HUMAN | **Werner syndrome ATP-dependent helicase** |  |
| 2 | | 30 | Ace | | SE | SEKSVEAAAELSAKDLKEKKEKVEEKASR | N.D | N.D | P20962 | PTMS_HUMAN | **Parathymosin** |  |
| 2 | | 9 | Ace | | SE | SELPGDVR | 100% Ac- | 100.00 | Q15527 | SURF2_HUMAN | **Surfeit locus protein 2** |  |
| 2 | | 14 | Ace | | SE | SENNKNSLESSLR | 100% Ac- | 100.00 | P09913 | IFIT2_HUMAN | **Interferon-induced protein with tetratricopeptide repeats 2** |  |
| 2 | | 9 | Ace | | SE | SEPAGDVR | 100% Ac- | 100.00 | P38936 | CDN1A_HUMAN | **Cyclin-dependent kinase inhibitor 1** |  |
| 2 | | 16 | Ace | | SE | SEPKAIDPKLSTTDR | Partial-Ac- | 85.51 | Q08209 | PP2BA_HUMAN | **Serine/threonine-protein phosphatase 2B catalytic subunit alpha isoform** |  |
| 2 | | 10 | Ace | | SE | SEQSICQAR | 100% Ac- | 100.00 | Q8N8S7 | ENAH_HUMAN | **Protein enabled homolog** | Q9UI08 (2-10) |
| 2 | | 15 | Ace | | SE | SEQTPAEAGAAGAR | 100% Ac- | 100.00 | O94913 | PCF11_HUMAN | **Pre-mRA cleavage complex 2 protein Pcf11** |  |
| 2 | | 17 | Ace | | SE | SESFDCAKCNESLYGR | 100% Ac- | 100.00 | Q13643 | FHL3_HUMAN | **Four and a half LIM domains protein 3** |  |
| 2 | | 19 | Ace | | SE | SESLVVCDVAEDLVEKLR | 100% Ac- | 100.00 | P60983 | GMFB_HUMAN | **Glia maturation factor beta** |  |
| 2 | | 24 | Ace | | SE | SESSSKSSQPLASKQEKDGTEKR | 100% Ac- | 100.00 | P17096 | HMGA1_HUMAN | **High mobility group protein HMG-I/HMG-Y** |  |
| 2 | | 33 | Ace | | SE | SETAPAAPAAAPPAEKAPVKKKAAKKAGGTPR | 100% Ac- | 100.00 | P16403 | H12_HUMAN | **Histone H1.2** |  |
| 2 | | 25 | Ace | | SE | SETAPAAPAAPAPAEKTPVKKKAR | Partial-Ac- | 72.25 | P10412 | H14_HUMAN | **Histone H1.4** |  |
| 2 | | 14 | Ace | | SE | SETPAQCSIKQER | 100% Ac- | 100.00 | P41212 | ETV6_HUMAN | **Transcription factor ETV6** |  |
| 2 | | 10 | Ace | | SE | SETVICSSR | 100% Ac- | 100.00 | P50552 | VASP_HUMAN | **Vasodilator-stimulated phosphoprotein** |  |
| 2 | | 9 | Ace | | SE | SEVSCKKR | 100% Ac- | 100.00 | P32321 | DCTD_HUMAN | **Deoxycytidylate deaminase** |  |
| 2 | | 8 | Ace | | SF | SFLFGSR | 100% Ac- | 100.00 | Q7L9L4 | MOL1A_HUMAN | **Mps one binder kinase activator-like 1A** |  |
| 2 | | 8 | Ace | | SF | SFLFSSR | 100% Ac- | 100.00 | Q9H8S9 | MOL1B_HUMAN | **Mps one binder kinase activator-like 1B** |  |
| 2 | | 18 | Ace | | SF | SFLKSFPPPGPAEGLLR | Partial-Ac- | 84.03 | P54105 | ICLN_HUMAN | **Methylosome subunit pICln** |  |
| 2 | | 10 | Ace | | SF | SFPKAPLKR | 100% Ac- | 100.00 | O75330 | HMMR_HUMAN | **Hyaluronan mediated motility receptor** |  |
| 2 | | 22 | Ace | | SG | SGASSSEQNNNSYETKTPNLR | 100% Ac- | 100.00 | O60825 | F262_HUMAN | **6-phosphofructo-2-kinase/fructose-2,6-biphosphatase 2** |  |
| 2 | | 11 | Ace | | SG | SGASVKVAVR | 100% Ac- | 100.00 | O60333 | KIF1B_HUMAN | **Kinesin-like protein KIF1B** |  |
| 2 | | 30 | Ace | | SG | SGEDEQQEQTIAEDLVVTKYKMGGDIANR | 100% Ac- | 100.00 | Q9UQ80 | PA2G4_HUMAN | **Proliferation-associated protein 2G4** |  |
| 2 | | 19 | Ace | | SG | SGEDGPAAGPGAAAAAAR | 100% Ac- | 100.00 | Q9BZL4 | PP12C_HUMAN | **Protein phosphatase 1 regulatory subunit 12C** |  |
| 2 | | 22 | Ace | | SG | SGEENPASKPTPVQDVQGDGR | 100% Ac- | 100.00 | Q15102 | PA1B3_HUMAN | **Platelet-activating factor acetylhydrolase IB subunit gamma** |  |
| 2 | | 8 | Ace | | SG | SGELSNR | 100% Ac- | 100.00 | Q9BQI0 | IBA2_HUMAN | **Ionized calcium-binding adapter molecule 2** |  |
| 2 | | 24 | Ace | | SG | SGEPGQTSVAPPPEEVEPGSGVR | 100% Ac- | 100.00 | Q9BRT3 | CQ037_HUMAN | **Uncharacterized protein C17orf37** |  |
| 2 | | 27 | Ace | | SG | SGFDDPGIFYSDSFGGDAQADEGQAR | 100% Ac- | 100.00 | P33992 | MCM5_HUMAN | **DNA replication licensing factor MCM5** |  |
| 2 | | 9 | Ace | | SG | SGFLEGLR | 100% Ac- | 100.00 | O95807 | TM50A_HUMAN | **Transmembrane protein 50A** |  |
| 2 | | 9 | Ace | | SG | SGFSTEER | 100% Ac- | 100.00 | Q15181 | IPYR_HUMAN | **Inorganic pyrophosphatase** |  |
| 2 | | 23 | Ace | | SG | SGGGTETPVGCEAAPGGGSKKR | 100% Ac- | 100.00 | Q8TDH9 | MUTED_HUMAN | **Protein Muted homolog** |  |
| 2 | | 10 | Ace | | SG | SGGLLKALR | 100% Ac- | 100.00 | P52298 | NCBP2_HUMAN | **Nuclear cap-binding protein subunit 2** |  |
| 2 | | 10 | Ace | | SG | SGGSADYNR | N.D | N.D | Q14240 | IF4A2_HUMAN | **Eukaryotic initiation factor 4A-II** |  |
| 2 | | 8 | Ace | | SG | SGIALSR | 100% Ac- | 100.00 | P63279 | UBC9_HUMAN | **SUMO-conjugating enzyme UBC9** |  |
| 2 | | 17 | Ace | | SG | SGKANASKKNAQQLKR | 100% Ac- | 100.00 | Q7L2Z9 | CENPQ_HUMAN | **Centromere protein Q** |  |
| 2 | | 9 | Ace | | SG | SGLDGVKR | 100% Ac- | 100.00 | Q9H0U9 | TSYL1_HUMAN | **Testis-specific Y-encoded-like protein 1** |  |
| 2 | | 16 | Ace | | SG | SGLGENLDPLASDSR | 100% Ac- | 100.00 | Q9Y6Q9 | NCOA3_HUMAN | **Nuclear receptor coactivator 3** |  |
| 2 | | 9 | Ace | | SG | SGLVLGQR | 100% Ac- | 100.00 | Q9NSK0 | KLC4_HUMAN | **Kinesin light chain 4** |  |
| 2 | | 19 | Ace | | SG | SGNGNAAATAEENSPKMR | 100% Ac- | 100.00 | P08397 | HEM3_HUMAN | **Porphobilinogen deaminase** |  |
| 2 | | 9 | Ace | | SG | SGQSLTDR | 100% Ac- | 100.00 | Q13492 | PICAL_HUMAN | **Phosphatidylinositol-binding clathrin assembly protein** |  |
| 2 | | 20 | Ace | | SG | SGSCAAPGPGSGSSPAACR | 100% Ac- | 100.00 | Q6ZUT9 | DEN5B_HUMAN | **DEN domain-containing protein 5B** |  |
| 2 | | 37 | Ace | | SG | SGSNPKAAAAASAAGPGGLVAGKEEKKKAGGGVLNR | 100% Ac- | 100.00 | A6NIH7 | U119B_HUMAN | **Protein unc-119 homolog B** |  |
| 2 | | 18 | Ace | | SG | SGSSSVAAMKKVVQQLR | 100% Ac- | 100.00 | P63218 | GBG5_HUMAN | **Guanine nucleotide-binding protein G(I)/G(S)/G(O) subunit gamma-5** |  |
| 2 | | 17 | Ace | | SG | SGTNLDGNDEFDEQLR | 100% Ac- | 100.00 | O43719 | HTSF1_HUMAN | **HIV Tat-specific factor 1** |  |
| 2 | | 22 | Ace | | SG | SGTSSPEAVKKLLENMQSDLR | 100% Ac- | 100.00 | Q7Z3U7 | MON2_HUMAN | **Protein MO2 homolog** |  |
| 2 | | 12 | Ace | | SI | SIAGVAAQEIR | 100% Ac- | 100.00 | Q9UJM3 | ERRFI_HUMAN | **ERBB receptor feedback inhibitor 1** |  |
| 2 | | 12 | Ace | | SI | SIEIESSDVIR | 100% Ac- | 100.00 | Q2TAY7 | SMU1_HUMAN | **WD40 repeat-containing protein SMU1** |  |
| 2 | | 11 | Ace | | SI | SIETLLEAAR | 100% Ac- | 100.00 | Q99583 | MNT_HUMAN | **Max-binding protein MT** |  |
| 2 | | 11 | Ace | | SI | SIFTPTNQIR | 100% Ac- | 100.00 | Q9Y3A5 | SBDS_HUMAN | **Ribosome maturation protein SBDS** |  |
| 2 | | 26 | Ace | | SI | SIMSYNGGAVMAMKGKNCVAIAADR | 100% Ac- | 100.00 | P49720 | PSB3_HUMAN | **Proteasome subunit beta type-3** |  |
| 2 | | 14 | Ace | | SI | SISSDEVNFLVYR | 100% Ac- | 100.00 | Q9BZK7 | TBL1R_HUMAN | **F-box-like/WD repeat-containing protein TBL1XR1** |  |
| 2 | | 14 | Ace | | SI | SITSDEVNFLVYR | 100% Ac- | 100.00 | O60907 | TBL1X_HUMAN | **F-box-like/WD repeat-containing protein TBL1X** | Q9BQ87 (2-14) |
| 2 | | 10 | Ace | | SK | SKISEAVKR | 100% Ac- | 100.00 | P30838 | AL3A1_HUMAN | **Aldehyde dehydrogenase, dimeric ADP-preferring** |  |
| 2 | | 20 | Ace | | SK | SKKISGGSVVEMQGDEMTR | 100% Ac- | 100.00 | O75874 | IDHC_HUMAN | **Isocitrate dehydrogenase [ADP] cytoplasmic** |  |
| 2 | | 13 | Ace | | SK | SKKKGLSAEEKR | 100% Ac- | 100.00 | Q9BWT6 | MND1_HUMAN | **Meiotic nuclear division protein 1 homolog** |  |
| 2 | | 17 | Ace | | SK | SKLGKFFKGGGSSKSR | 100% Ac- | 100.00 | Q96CF2 | CHM4C_HUMAN | **Charged multivesicular body protein 4c** |  |
| 2 | | 11 | Ace | | SK | SKLKSSESVR | 100% Ac- | 100.00 | O15066 | KIF3B_HUMAN | **Kinesin-like protein KIF3B** |  |
| 2 | | 17 | Ace | | SK | SKLKVIPEKSLTNNSR | 100% Ac- | 100.00 | Q9UPW5 | CBPC1_HUMAN | **Cytosolic carboxypeptidase 1** |  |
| 2 | | 10 | Ace | | SK | SKNTVSSAR | 100% Ac- | 100.00 | O15511 | ARPC5_HUMAN | **Actin-related protein 2/3 complex subunit 5** |  |
| 2 | | 18 | Ace | | SK | SKPPPKPVKPGQVKVFR | 100% Ac- | 100.00 | Q92882 | OSTF1_HUMAN | **Osteoclast-stimulating factor 1** |  |
| 2 | | 14 | Ace | | SK | SKSESPKEPEQLR | 100% Ac- | 100.00 | P09651 | ROA1_HUMAN | **Heterogeneous nuclear ribonucleoprotein A1** | P0C7M2 (2-14) |
| 2 | | 12 | Ace | | SK | SKSFQQSSLSR | 100% Ac- | 100.00 | P43243 | MATR3_HUMAN | **Matrin-3** |  |
| 2 | | 13 | Ace | | SK | SKSLKKLVEESR | 100% Ac- | 100.00 | Q15404 | RSU1_HUMAN | **Ras suppressor protein 1** |  |
| 2 | | 12 | Ace | | SK | SKTNKSKSGSR | 100% Ac- | 100.00 | Q9Y2W1 | TR150_HUMAN | **Thyroid hormone receptor-associated protein 3** |  |
| 2 | | 19 | Ace | | SL | SLDIQSLDIQCEELSDAR | 100% Ac- | 100.00 | P13489 | RINI_HUMAN | **Ribonuclease inhibitor** |  |
| 2 | | 11 | Ace | | SL | SLEDPFFVVR | 100% Ac- | 100.00 | O60499 | STX10_HUMAN | **Syntaxin-10** |  |
| 2 | | 22 | Ace | | SL | SLKLQASNVTNKNDPKSINSR | 100% Ac- | 100.00 | Q9UKM9 | RALY_HUMAN | **RA-binding protein Raly** |  |
| 2 | | 12 | Ace | | SL | SLLDGLASSPR | N.D | N.D | Q3YBR2 | TBRG1_HUMAN | **Transforming growth factor beta regulator 1** |  |
| 2 | | 19 | Ace | | SL | SLLNKPKSEMTPEELQKR | 100% Ac- | 100.00 | P62316 | SMD2_HUMAN | **Small nuclear ribonucleoprotein Sm D2** |  |
| 2 | | 11 | Ace | | SL | SLNPPIFLKR | 100% Ac- | 100.00 | Q8N5C7 | DTWD1_HUMAN | **DTW domain-containing protein 1** |  |
| 2 | | 10 | Ace | | SL | SLPLTEEQR | 100% Ac- | 100.00 | Q9NZC9 | SMAL1_HUMAN | **SWI/SF-related matrix-associated actin-dependent regulator of chromatin subfamily A-like protein 1** |  |
| 2 | | 10 | Ace | | SL | SLQSAQYLR | 100% Ac- | 100.00 | Q92540 | SMG7_HUMAN | **Protein SMG7** |  |
| 2 | | 32 | Ace | | SL | SLQVLNDKNVSNEKNTENCDFLFSPPEVTGR | 100% Ac- | 100.00 | Q9Y6A5 | TACC3_HUMAN | **Transforming acidic coiled-coil-containing protein 3** |  |
| 2 | | 18 | Ace | | SL | SLSNKLTLDKLDVKGKR | 100% Ac- | 100.00 | P00558 | PGK1_HUMAN | **Phosphoglycerate kinase 1** |  |
| 2 | | 15 | Ace | | SL | SLVDLGKKLLEAAR | 100% Ac- | 100.00 | Q06547 | GABP1_HUMAN | **GA-binding protein subunit beta-1** |  |
| 2 | | 9 | Ace | | SL | SLVDLGKR | N.D | N.D | Q8TAK5 | GABP2_HUMAN | **GA-binding protein subunit beta-2** |  |
| 2 | | 13 | Ace | | SL | SLVLNDLLICCR | N.D | N.D | Q13315 | ATM_HUMAN | **Serine-protein kinase ATM** |  |
| 2 | | 11 | Ace | | SM | SMILSASVIR | 100% Ac- | 100.00 | Q96IW7 | SC22A_HUMAN | **Vesicle-trafficking protein SEC22a** |  |
| 2 | | 11 | Ace | | SM | SMTLGYWDIR | 100% Ac- | 100.00 | Q03013 | GSTM4_HUMAN | **Glutathione S-transferase Mu 4** |  |
| 2 | | 42 | Ace | | SN | SNEVETSATNGQPDQQAAPKAPSKKEKKKGPEKTDEYLLAR | 100% Ac- | 100.00 | P98082 | DAB2_HUMAN | **Disabled homolog 2** |  |
| 2 | | 11 | Ace | | SN | SNKEGSGGFR | 100% Ac- | 100.00 | Q99598 | TSNAX_HUMAN | **Translin-associated protein X** |  |
| 2 | | 11 | Ace | | SN | SNLSKGTGSR | 100% Ac- | 100.00 | Q13618 | CUL3_HUMAN | **Cullin-3** |  |
| 2 | | 18 | Ace | | SN | SNNGLDIQDKPPAPPMR | 100% Ac- | 100.00 | Q13153 | PAK1_HUMAN | **Serine/threonine-protein kinase PAK 1** |  |
| 2 | | 14 | AcD3 | | SP | SPTPPLFSLPEAR | 100% free | 0.00 | Q96FV9 | THOC1_HUMAN | **THO complex subunit 1** |  |
| 2 | | 14 | Ace | | SQ | SQAEFEKAAEEVR | 100% Ac- | 100.00 | P07108 | ACBP_HUMAN | **Acyl-CoA-binding protein** |  |
| 2 | | 9 | Ace | | SQ | SQEGDYGR | N.D | N.D | Q9NUW8 | TYDP1_HUMAN | **Tyrosyl-DA phosphodiesterase 1** |  |
| 2 | | 13 | Ace | | SQ | SQEGVELEKSVR | 100% Ac- | 100.00 | Q9NUL5 | CS066_HUMAN | **UPF0515 protein C19orf66** |  |
| 2 | | 31 | Ace | | SQ | SQKQEEENPAEETGEEKQDTQEKEGILPER | 100% Ac- | 100.00 | O43768 | ENSA_HUMAN | **Alpha-endosulfine** |  |
| 2 | | 14 | Ace | | SQ | SQPPLLPASAETR | 100% Ac- | 100.00 | Q9BZ29 | DOCK9_HUMAN | **Dedicator of cytokinesis protein 9** |  |
| 2 | | 20 | Ace | | SQ | SQSGAVSCCPGATNGSLGR | 100% Ac- | 100.00 | Q9H6S3 | ES8L2_HUMAN | **Epidermal growth factor receptor kinase substrate 8-like protein 2** |  |
| 2 | | 9 | Ace | | SQ | SQSKGKKR | 100% Ac- | 100.00 | P52564 | MP2K6_HUMAN | **Dual specificity mitogen-activated protein kinase kinase 6** |  |
| 2 | | 10 | Ace | | SQ | SQTQDYECR | 100% Ac- | 100.00 | Q9C0H5 | K1688_HUMAN | **Uncharacterized protein KIAA1688** |  |
| 2 | | 19 | Ace | | SS | SSAPTTPPSVDKVDGFSR | 100% Ac- | 100.00 | Q16537 | 2A5E_HUMAN | **Serine/threonine-protein phosphatase 2A 56 kDa regulatory subunit epsilon isoform** |  |
| 2 | | 11 | Ace | | SS | SSESEKDKER | N.D | N.D | Q5U649 | CL060_HUMAN | **Uncharacterized protein C12orf60** |  |
| 2 | | 9 | Ace | | SS | SSESSKKR | 100% Ac- | 100.00 | Q13769 | THOC5_HUMAN | **THO complex subunit 5 homolog** |  |
| 2 | | 16 | Ace | | SS | SSGADGGGGAAVAAR | 100% Ac- | 100.00 | Q5JPI9 | METLA_HUMAN | **Methyltransferase-like protein 10** |  |
| 2 | | 12 | Ace | | SS | SSGASASALQR | 100% Ac- | 100.00 | P50151 | GBG10_HUMAN | **Guanine nucleotide-binding protein G(I)/G(S)/G(O) subunit gamma-10** |  |
| 2 | | 20 | Ace | | SS | SSIGTGYDLSASTFSPDGR | 100% Ac- | 100.00 | P25788 | PSA3_HUMAN | **Proteasome subunit alpha type-3** |  |
| 2 | | 14 | Ace | | SS | SSILPFTPPIVKR | 100% Ac- | 100.00 | P84022 | SMAD3_HUMAN | **Mothers against decapentaplegic homolog 3** |  |
| 2 | | 14 | Ace | | SS | SSILPFTPPVVKR | 100% Ac- | 100.00 | Q15796 | SMAD2_HUMAN | **Mothers against decapentaplegic homolog 2** |  |
| 2 | | 14 | Ace | | SS | SSKEVKTALKSAR | 100% Ac- | 100.00 | Q6PGP7 | TTC37_HUMAN | **Tetratricopeptide repeat protein 37** |  |
| 2 | | 14 | Ace | | SS | SSKKAKTKTTKKR | 100% Ac- | 100.00 | O14950 | MRLC2_HUMAN | **Myosin regulatory light chain MRLC2** |  |
| 2 | | 12 | Ace | | SS | SSKQEIMSDQR | 100% Ac- | 100.00 | Q9H501 | ESF1_HUMAN | **ESF1 homolog** |  |
| 2 | | 15 | Ace | | SS | SSKTASTNNIAQAR | 100% Ac- | 100.00 | Q9UBI6 | GBG12_HUMAN | **Guanine nucleotide-binding protein G(I)/G(S)/G(O) subunit gamma-12** |  |
| 2 | | 7 | Ace | | SS | SSKVSR | 100% Ac- | 100.00 | P62906 | RL10A_HUMAN | **60S ribosomal protein L10a** |  |
| 2 | | 10 | Ace | | SS | SSLSEYAFR | 100% Ac- | 100.00 | Q9Y291 | RT33_HUMAN | **28S ribosomal protein S33, mitochondrial** |  |
| 2 | | 11 | Ace | | SS | SSNECFKCGR | 100% Ac- | 100.00 | P62633 | CNBP_HUMAN | **Cellular nucleic acid-binding protein** |  |
| 2 | | 13 | Ace | | SS | SSPMPDCTSKCR | 100% Ac- | 100.00 | Q9P2D0 | IBTK_HUMAN | **Inhibitor of Bruton tyrosine kinase** |  |
| 2 | | 25 | Ace | | SS | SSPPEGKLETKAGHPPAVKAGGMR | 100% Ac- | 100.00 | P51397 | DAP1_HUMAN | **Death-associated protein 1** |  |
| 2 | | 10 | Ace | | SS | SSQKGNVAR | 100% Ac- | 100.00 | Q96MD7 | CI085_HUMAN | **Uncharacterized protein C9orf85** |  |
| 2 | | 24 | Ace | | SS | SSSLGKEKDSKEKDPKVPSAKER | 100% Ac- | 100.00 | Q9UQ13 | SHOC2_HUMAN | **Leucine-rich repeat protein SHOC-2** |  |
| 2 | | 10 | Ace | | SS | SSSPLSKKR | 100% Ac- | 100.00 | P22314 | UBA1_HUMAN | **Ubiquitin-like modifier-activating enzyme 1** |  |
| 2 | | 23 | Ace | | SS | SSSPVNVKKLKVSELKEELKKR | 100% Ac- | 100.00 | Q00839 | HNRPU_HUMAN | **Heterogeneous nuclear ribonucleoprotein U** |  |
| 2 | | 13 | Ace | | SS | SSSVEQKKGPTR | 100% Ac- | 100.00 | Q8IWS0 | PHF6_HUMAN | **PHD finger protein 6** |  |
| 2 | | 17 | Ace | | SS | SSTLAKIAEIEAEMAR | 100% Ac- | 100.00 | Q9Y295 | DRG1_HUMAN | **Developmentally-regulated GTP-binding protein 1** |  |
| 2 | | 21 | Ace | | SS | SSTQFNKGPSYGLSAEVKNR | 100% Ac- | 100.00 | Q99439 | CNN2_HUMAN | **Calponin-2** |  |
| 2 | | 12 | Ace | | SS | SSVQQQPPPPR | 100% Ac- | 100.00 | O00401 | WASL_HUMAN | **Neural Wiskott-Aldrich syndrome protein** |  |
| 2 | | 17 | Ace | | ST | STAQSLKSVDYEVFGR | N.D | N.D | P14621 | ACYP2_HUMAN | **Acylphosphatase-2** |  |
| 2 | | 10 | Ace | | ST | STELFSSTR | 100% Ac- | 100.00 | P30307 | MPIP3_HUMAN | **M-phase inducer phosphatase 3** |  |
| 2 | | 10 | Ace | | ST | STGDSFETR | 100% Ac- | 100.00 | Q13464 | ROCK1_HUMAN | **Rho-associated protein kinase 1** |  |
| 2 | | 12 | Ace | | ST | STGGDFGNPLR | 100% Ac- | 100.00 | P20340 | RAB6A_HUMAN | **Ras-related protein Rab-6A** |  |
| 2 | | 13 | Ace | | ST | STGPTAATGSNR | 100% Ac- | 100.00 | Q15836 | VAMP3_HUMAN | **Vesicle-associated membrane protein 3** |  |
| 2 | | 15 | Ace | | ST | STGTFVVSQPLNYR | 100% Ac- | 100.00 | P49189 | AL9A1_HUMAN | **4-trimethylaminobutyraldehyde dehydrogenase** |  |
| 2 | | 11 | Ace | | ST | STKVPIYLKR | 100% Ac- | 100.00 | O14613 | BORG1_HUMAN | **Cdc42 effector protein 2** |  |
| 2 | | 11 | Ace | | ST | STLFPSLFPR | 100% Ac- | 100.00 | P60006 | CK051_HUMAN | **Uncharacterized protein C11orf51** |  |
| 7 | | 20 | Ace | | ST | STLPVEDEESSESR | 100% Ac- | 100.00 | P78347 | GTF2I_HUMAN | **General transcription factor II-I** |  |
| 2 | | 13 | Ace | | ST | STNENANTPAAR | 100% Ac- | 100.00 | P52292 | IMA2_HUMAN | **Importin subunit alpha-2** |  |
| 7 | | 18 | Ace | | ST | STPLPAIVPAAR | 100% Ac- | 100.00 | O75608 | LYPA1_HUMAN | **Acyl-protein thioesterase 1** |  |
| 2 | | 26 | Ace | | ST | STPPLAASGMAPGPFAGPQAQQAAR | 100% Ac- | 100.00 | Q96HR3 | MED30_HUMAN | **Mediator of RA polymerase II transcription subunit 30** |  |
| 2 | | 14 | Ace | | ST | STAAVPELKQISR | 100% Ac- | 100.00 | Q9BRJ7 | SDOS_HUMAN | **Protein syndesmos** |  |
| 2 | | 9 | Ace | | SV | SVACVLKR | 100% Ac- | 100.00 | Q14671 | PUM1_HUMAN | **Pumilio homolog 1** |  |
| 2 | | 9 | Ace | | SV | SVAFAAPR | 100% Ac- | 100.00 | Q15532 | SSXT_HUMAN | **Protein SSXT** |  |
| 2 | | 9 | Ace | | SV | SVAGGEIR | 100% Ac- | 100.00 | Q9H773 | XTP3A_HUMAN | **XTP3-transactivated gene A protein** |  |
| 2 | | 28 | Ace | | SV | SVFGKLFGAGGGKAGKGGPTPQEAIQR | 100% Ac- | 100.00 | Q9H444 | CHM4B_HUMAN | **Charged multivesicular body protein 4b** |  |
| 2 | | 11 | Ace | | SV | SVKEAGSSGR | 100% Ac- | 100.00 | Q13459 | MYO9B_HUMAN | **Myosin-IXb** |  |
| 2 | | 9 | Ace | | SV | SVKEGAQR | 100% Ac- | 100.00 | Q27J81 | INF2_HUMAN | **Inverted formin-2** |  |
| 2 | | 9 | Ace | | SV | SVLGEYER | 100% Ac- | 100.00 | Q8TD19 | NEK9_HUMAN | **Serine/threonine-protein kinase ek9** |  |
| 2 | | 9 | Ace | | SV | SVNMDELR | N.D | N.D | Q8IYN6 | F100B_HUMAN | **Protein FAM100B** |  |
| 2 | | 7 | Ace | | SV | SVNVNR | 100% Ac- | 100.00 | P55010 | IF5_HUMAN | **Eukaryotic translation initiation factor 5** |  |
| 2 | | 19 | Ace | | SV | SVPAFIDISEEDQAAELR | 100% Ac- | 100.00 | Q7L2H7 | EIF3M_HUMAN | **Eukaryotic translation initiation factor 3 subunit M** |  |
| 2 | | 23 | Ace | | SV | SVPSALMKQPPIQSTAGAVPVR | 100% Ac- | 100.00 | P55081 | MFAP1_HUMAN | **Microfibrillar-associated protein 1** |  |
| 2 | | 17 | Ace | | SV | SVQVAAPGSAGLGPER | N.D | N.D | Q6P597 | KLC3_HUMAN | **Kinesin light chain 3** |  |
| 2 | | 9 | Ace | | SV | SVSLVVIR | 100% Ac- | 100.00 | Q7Z569 | BRAP_HUMAN | **BRCA1-associated protein** |  |
| 2 | | 19 | Ace | | SV | SVVGLDVGSQSCYIAVAR | N.D | N.D | Q92598 | HS105_HUMAN | **Heat shock protein 105 kDa** |  |
| 2 | | 10 | Ace | | SY | SYGPLDMYR | N.D | N.D | Q86Y82 | STX12_HUMAN | **Syntaxin-12** |  |
| 2 | | 15 | Ace | | SY | SYIPGQPVTAVVQR | 100% Ac- | 100.00 | O14907 | TX1B3_HUMAN | **Tax1-binding protein 3** |  |
| 2 | | 38 | Ace | | SY | SYPADDYESEAAYDPYAYPSDYDMHTGDPKQDLAYER | 100% Ac- | 100.00 | Q9Y262 | IF3EI_HUMAN | **Eukaryotic translation initiation factor 3 subunit E-interacting protein** |  |
| 2 | | 11 | Ace | | SY | SYQGKKNIPR | 100% Ac- | 100.00 | Q16555 | DPYL2_HUMAN | **Dihydropyrimidinase-related protein 2** | Q14195 (2-11) |
| 2 | | 11 | Ace | | SY | SYQGKKNIPR | 100% Ac- | 100.00 | Q14195 | DPYL3_HUMAN | **Dihydropyrimidinase-related protein 3** | **Q16555 (2-11)** |
| 2 | | 17 | Ace | | SY | SYTPGVGGDPAQLAQR | 100% Ac- | 100.00 | O15400 | STX7_HUMAN | **Syntaxin-7** |  |
| 2 | | 14 | Ace | | SA | SAADEVDGLGVAR | 100% Ac- | 100.00 | P46940 | IQGA1_HUMAN | **Ras GTPase-activating-like protein IQGAP1** |  |
| 2 | | 10 | Ace | | SA | SAAKENPCR | 100% Ac- | 100.00 | Q6WCQ1 | MPRIP_HUMAN | **Myosin phosphatase Rho-interacting protein** |  |
| 2 | | 13 | Ace | | SA | SAALFSLDGPAR | 100% Ac- | 100.00 | P49716 | CEBPD_HUMAN | **CCAAT/enhancer-binding protein delta** |  |
| 2 | | 10 | Ace | | SA | SAAQVSSSR | 100% Ac- | 100.00 | Q9H1B7 | EAP1_HUMAN | **Enhanced at puberty protein 1** |  |
| 2 | | 12 | Ace | | SA | SAAVTAGKLAR | 100% Ac- | 100.00 | P53350 | PLK1_HUMAN | **Serine/threonine-protein kinase PLK1** |  |
| **23. Thr** | | |  | |  |  |  |  |  |  |  |  |
| 2 | | 41 | AcD3 | | TA | TAEDSTAAMSSDSAAGSSAKVPEGVAGAPNEAALLALMER | 100% free | 0.00 | A0AV96 | RBM47_HUMAN | **RNA-binding protein 47** |  |
| 2 | | 39 | AcD3 | | TA | TAEEMKATESGAQSAPLPMEGVDISPKQDEGVLKVIKR | 100% free | 0.00 | Q02790 | FKBP4_HUMAN | **FK506-binding protein 4** |  |
| 2 | | 11 | Ace | | TA | TAIIKEIVSR | 100% Ac- | 100.00 | P60484 | PTEN_HUMAN | **Phosphatidylinositol-3,4,5-trisphosphate 3-phosphatase and dual-specificity protein phosphatase PTE** |  |
| 2 | | 17 | Ace | | TA | TAPCPPPPPDPQFVLR | 100% Ac- | 100.00 | Q9BYB4 | GNB1L_HUMAN | **Guanine nucleotide-binding protein subunit beta-like protein 1** |  |
| 2 | | 11 | Ace | | TA | TAQGGLVANR | 100% Ac- | 100.00 | Q5J8M3 | TMM85_HUMAN | **Transmembrane protein 85** |  |
| 2 | | 10 | Ace | | TA | TAVNVALIR | N.D | N.D | Q9NUK0 | MBNL3_HUMAN | **Muscleblind-like protein 3** |  |
| 2 | | 9 | Ace | | TD | TDDKDVLR | Partial-Ac- | 86.83 | Q9H1Y0 | ATG5_HUMAN | **Autophagy protein 5** |  |
| 2 | | 10 | Ace | | TD | TDFKLGIVR | 100% Ac- | 100.00 | Q96E35 | ZMY19_HUMAN | **Zinc finger MYD domain-containing protein 19** |  |
| 2 | | 10 | Ace | | TD | TDPFCVGGR | 100% Ac- | 100.00 | Q96KM6 | Z512B_HUMAN | **Zinc finger protein 512B** |  |
| 2 | | 30 | Ace | | TD | TDSKYFTTNKKGEIFELKAELNNEKKEKR | 100% Ac- | 100.00 | P63010 | AP2B1_HUMAN | **AP-2 complex subunit beta-1** |  |
| 2 | | 18 | Ace | | TD | TDTAEAVPKFEEMFASR | 100% Ac- | 100.00 | Q9BTL3 | F103A_HUMAN | **Protein FAM103A1** |  |
| 2 | | 16 | Ace | | TD | TDVETTYADFIASGR | N.D | N.D | P61925 | IPKA_HUMAN | **cAMP-dependent protein kinase inhibitor alpha** |  |
| 2 | | 9 | Ace | | TD | TDYGEEQR | 100% Ac- | 100.00 | Q9H446 | RWDD1_HUMAN | **RWD domain-containing protein 1** |  |
| 2 | | 10 | Ace | | TE | TEESSDVPR | Partial-Ac- | 83.87 | Q5VZK9 | LR16A_HUMAN | **Leucine-rich repeat-containing protein 16A** |  |
| 2 | | 8 | Ace | | TE | TEGTCLR | 100% Ac- | 100.00 | P48449 | ERG7_HUMAN | **Lanosterol synthase** |  |
| 2 | | 11 | Ace | | TE | TELQSALLLR | 100% Ac- | 100.00 | P62253 | UB2G1_HUMAN | **Ubiquitin-conjugating enzyme E2 G1** |  |
| 2 | | 8 | Ace | | TE | TEQMTLR | 100% Ac- | 100.00 | P63244 | GBLP_HUMAN | **Guanine nucleotide-binding protein subunit beta-2-like 1** |  |
| 2 | | 16 | Ace | | TE | TEVVPSSALSEVSLR | N.D | N.D | Q9H7X0 | NAT15_HUMAN | **N-acetyltransferase 15** |  |
| 2 | | 11 | Ace | | TG | TGKSVKDVDR | Partial-Ac- | 61.39 | Q8WU79 | SMAP2_HUMAN | **Stromal membrane-associated protein 2** |  |
| 2 | | 17 | AcD3 | | TG | TGSNMSDALANAVCQR | 100% free | 0.00 | Q7Z4I7 | LIMS2_HUMAN | **LIM and senescent cell antigen-like-containing domain protein 2** |  |
| 2 | | 12 | Ace | | TK | TKAGSKGGNLR |  | 67.60 | Q96AG4 | LRC59_HUMAN | **Leucine-rich repeat-containing protein 59** |  |
| 2 | | 11 | AcD3 | | TK | TKGTSSFGKR | 100% free | 0.00 | P61927 | RL37_HUMAN | **60S ribosomal protein L37** |  |
| 2 | | 22 | AcD3 | | TK | TKIKADPDGPEAQAEACSGER | 100% free | 0.00 | Q9NX24 | NHP2_HUMAN | **H/ACA ribonucleoprotein complex subunit 2** |  |
| 2 | | 20 | Ace | | TM | TMDKSELVQKAKLAEQAER | 100% Ac- | 100.00 | P31946 | 1433B_HUMAN | **14-3-3 protein beta/alpha** |  |
| 2 | | 12 | Ace | | TN | TNEEPLPKKVR | 100% Ac- | 100.00 | Q15007 | FL2D_HUMAN | **Pre-mRA-splicing regulator WTAP** |  |
| 2 | | 8 | AcD3 | | TN | TNTKGKR | 100% free | 0.00 | P46778 | RL21_HUMAN | **60S ribosomal protein L21** |  |
| 2 | | 24 | AcD3 | | TQ | TQAEKGDTENGKEKGGEKEKEQR | 100% free | 0.00 | Q9H981 | ARP8_HUMAN | **Actin-related protein 8** |  |
| 2 | | 26 | Ace | | TQ | TQQGAALQNYNNELVKCIEELCQKR | N.D | N.D | O43805 | SSNA1_HUMAN | **Sjoegren syndrome nuclear autoantigen 1** |  |
| 2 | | 11 | Ace | | TS | TSALENYINR | 100% Ac- | 100.00 | O95777 | LSM8_HUMAN | **U6 snRA-associated Sm-like protein LSm8** |  |
| 2 | | 11 | Ace | | TS | TSALTQGLER | 100% Ac- | 100.00 | Q0VGL1 | CG059_HUMAN | **UPF0539 protein C7orf59** |  |
| 2 | | 26 | Ace | | TS | TSANKAIELQLQVKQNAEELQDFMR | 100% Ac- | 100.00 | Q9H6T3 | RPAP3_HUMAN | **RNA polymerase II-associated protein 3** |  |
| 2 | | 17 | AcD3 | | TS | TSDQDAKVVAEPQTQR | N.D | N.D | Q92615 | LARP5_HUMAN | **La-related protein 5** |  |
| 2 | | 14 | Ace | | TS | TSKQAMSSNEQER | 100% Ac- | 100.00 | Q8NB91 | FANCB_HUMAN | **Fanconi anemia group B protein** |  |
| 2 | | 10 | Ace | | TS | TSLAQQLQR | 100% Ac- | 100.00 | Q9H583 | HEAT1_HUMAN | **HEAT repeat-containing protein 1** |  |
| 2 | | 17 | Ace | | TS | TSMASLFSFTSPAVKR | 100% Ac- | 100.00 | Q99717 | SMAD5_HUMAN | **Mothers against decapentaplegic homolog 5** |  |
| 2 | | 9 | Ace | | TS | TSMTQSLR | 100% Ac- | 100.00 | Q9NPJ3 | THEM2_HUMAN | **Thioesterase superfamily member 2** |  |
| 2 | | 25 | Ace | | TS | TSPAKFKKDKEIIAEYDTQVKEIR | 100% Ac- | 100.00 | O75044 | FNBP2_HUMAN | **SLIT-ROBO Rho GTPase-activating protein 2** |  |
| 2 | | 10 | Ace | | TT | TTASTSQVR | 100% Ac- | 100.00 | P02794 | FRIH_HUMAN | **Ferritin heavy chain** |  |
| 2 | | 31 | AcD3 | | TT | TTDEGAKNNEESPTATVAEQGEDITSKKDR | N.D | N.D | Q13451 | FKBP5_HUMAN | **FK506-binding protein 5** |  |
| 2 | | 18 | Ace | | TT | TTPNKTPPGADPKQLER | 100% Ac- | 100.00 | Q9UM13 | APC10_HUMAN | **Anaphase-promoting complex subunit 10** |  |
| 2 | | 17 | Ace | | TT | TTQQIDLQGPGPWGFR | N.D | N.D | O00151 | PDLI1_HUMAN | **PDZ and LIM domain protein 1** |  |
| 2 | | 16 | Ace | | TT | TTSGALFPSLVPGSR | 100% Ac- | 100.00 | Q16186 | ADRM1_HUMAN | **Proteasomal ubiquitin receptor ADRM1** |  |
| 2 | | 30 | Ace | | TT | TTSTLQKAIDLVTKATEEDKAKNYEEALR | 100% Ac- | 100.00 | Q9UN37 | VPS4A_HUMAN | **Vacuolar protein sorting-associating protein 4A** |  |
| 2 | | 15 | Ace | | TT | TTTTTFKGVDPNSR | 100% Ac- | 100.00 | Q9UK76 | HN1_HUMAN | **Hematological and neurological expressed 1 protein** |  |
| 2 | | 24 | Ace | | TT | TTTVATDYDNIEIQQQYSDVNNR | 100% Ac- | 100.00 | Q01082 | SPTB2_HUMAN | **Spectrin beta chain, brain 1** |  |
| 2 | | 14 | Ace | | TT | TTYLEFIQQNEER | 100% Ac- | 100.00 | Q15436 | SC23A_HUMAN | **Protein transport protein Sec23A** |  |
| 2 | | 18 | Ace | | TA | TAAATATVLKEGVLEKR | N.D | N.D | Q9Y5J5 | PHLA3_HUMAN | **Pleckstrin homology-like domain family A member 3** |  |
| 2 | | 12 | Ace | | TA | TAAPASPQQIR | 100% Ac- | 100.00 | O95402 | MED26_HUMAN | **Mediator of RA polymerase II transcription subunit 26** |  |
| **24. Val-** | | |  | |  |  |  |  |  |  |  |  |
| 2 | | 9 | AcD3 | | VA | VAGMLMPR | 100% free | 0.00 | Q15149 | PLEC1_HUMAN | **Plectin-1** |  |
| 2 | | 9 | Ace | | VD | VDMMDLPR | 100% Ac- | 100.00 | P35244 | RFA3_HUMAN | **Replication protein A 14 kDa subunit** |  |
| 2 | | 12 | AcD3 | | VD | VDYYEVLGVQR | N.D | N.D | O75190 | DNJB6_HUMAN | **DnaJ homolog subfamily B member 6** |  |
| 2 | | 21 | AcD3 | | VE | VEKEEAGGGISEEEAAQYDR | Partial-Ac- | 20.62 | Q9UBE0 | SAE1_HUMAN | **SUMO-activating enzyme subunit 1** |  |
| 2 | | 12 | AcD3 | | VE | VEKGPEVSGKR | Partial-Ac- | 10.28 | Q9UKL0 | RCOR1_HUMAN | **REST corepressor 1** |  |
| 2 | | 9 | AcD3 | | VE | VEKKTSVR | 100% free | 0.00 | O43257 | ZNHI1_HUMAN | **Zinc finger HIT domain-containing protein 1** |  |
| 2 | | 18 | AcD3 | | VG | VGGEAAAAVEELVSGVR | 100% free | 0.00 | Q96HQ2 | C2AIL_HUMAN | **CDK2AIP -terminal-like protein** |  |
| 2 | | 22 | AcD3 | | VG | VGGGGVGGGLLENANPLIYQR | 100% free | 0.00 | Q9Y3D0 | FA96B_HUMAN | **UPF0195 protein FAM96B** |  |
| 2 | | 15 | AcD3 | | VG | VGPGPTAAAAVEER | 100% free | 0.00 | Q8IYA6 | CKP2L_HUMAN | **Cytoskeleton-associated protein 2-like** |  |
| 2 | | 23 | AcD3 | | VG | VGVKPVGSDPDFQPELSGAGSR | 100% free | 0.00 | O43396 | TXNL1_HUMAN | **Thioredoxin-like protein 1** |  |
| 2 | | 13 | AcD3 | | VI | VICCAAVNCSNR | N.D | N.D | Q8WY91 | THAP4_HUMAN | **THAP domain-containing protein 4** |  |
| 2 | | 27 | AcD3 | | VK | VKETQYYDILGVKPSASPEEIKKAYR | 100% free | 0.00 | Q8WW22 | DNJA4_HUMAN | **DnaJ homolog subfamily A member 4** |  |
| 2 | | 27 | AcD3 | | VK | VKETTYYDVLGVKPNATQEELKKAYR | 100% free | 0.00 | P31689 | DNJA1_HUMAN | **DnaJ homolog subfamily A member 1** |  |
| 2 | | 23 | AcD3 | | VK | VKIVTVKTQAYQDQKPGTSGLR | 100% free | 0.00 | P36871 | PGM1_HUMAN | **Phosphoglucomutase-1** |  |
| 2 | | 11 | AcD3 | | VK | VKLFIGNLPR | 100% free | 0.00 | Q9BQ04 | RBM4B_HUMAN | **RNA-binding protein 4B** | Q9BWF3 (2-11) |
| 2 | | 11 | AcD3 | | VK | VKLSKEAKQR | N.D | N.D | Q9P0U1 | TOM7_HUMAN | **Mitochondrial import receptor subunit TOM7 homolog** |  |
| 2 | | 20 | AcD3 | | VK | VKLTAELIEQAAQYTNAVR | 100% free | 0.00 | P09661 | RU2A_HUMAN | **U2 small nuclear ribonucleoprotein A'** |  |
| 2 | | 9 | AcD3 | | VK | VKPKYKGR | 100% free | 0.00 | Q13823 | NOG2_HUMAN | **ucleolar GTP-binding protein 2** |  |
| 2 | | 12 | AcD3 | | VK | VKYFLGQSVLR | 100% free | 0.00 | Q9Y255 | PRLD1_HUMAN | **PRELI domain-containing protein 1, mitochondrial** |  |
| 2 | | 8 | AcD3 | | VL | VLADLGR | 100% free | 0.00 | P61011 | SRP54_HUMAN | **Signal recognition particle 54 kDa protein** |  |
| 2 | | 11 | AcD3 | | VL | VLAELYVSDR | 100% free | 0.00 | O43776 | SYNC_HUMAN | **Asparaginyl-tRA synthetase, cytoplasmic** |  |
| 2 | | 9 | AcD3 | | VL | VLDLDLFR | 100% free | 0.00 | P49591 | SYSC_HUMAN | **Seryl-tRA synthetase, cytoplasmic** |  |
| 2 | | 17 | AcD3 | | VL | VLESTMVCVDNSEYMR | 100% free | 0.00 | P55036 | PSMD4_HUMAN | **26S proteasome non-ATPase regulatory subunit 4** |  |
| 2 | | 8 | AcD3 | | VL | VLESVAR | 100% free | 0.00 | Q8N5K1 | CISD2_HUMAN | **CDGSH iron sulfur domain-containing protein 2** |  |
| 2 | | 13 | AcD3 | | VL | VLESVVADLLNR | 100% free | 0.00 | Q709C8 | VP13C_HUMAN | **Vacuolar protein sorting-associated protein 13C** |  |
| 2 | | 8 | AcD3 | | VL | VLIKEFR | 100% free | 0.00 | P48739 | PIPNB_HUMAN | **Phosphatidylinositol transfer protein beta isoform** |  |
| 2 | | 15 | AcD3 | | VL | VLLESEQFLTELTR | 100% free | 0.00 | P37108 | SRP14_HUMAN | **Signal recognition particle 14 kDa protein** |  |
| 2 | | 8 | AcD3 | | VL | VLLKEYR | 100% free | 0.00 | Q00169 | PIPNA_HUMAN | **Phosphatidylinositol transfer protein alpha isoform** |  |
| 2 | | 9 | AcD3 | | VL | VLLTMIAR | 100% free | 0.00 | O75396 | SC22B_HUMAN | **Vesicle-trafficking protein SEC22b** |  |
| 2 | | 20 | AcD3 | | VL | VLNSLDKMIQLQKNTANIR | 100% free | 0.00 | Q7Z2Z2 | ETUD1_HUMAN | **Elongation factor Tu GTP-binding domain-containing protein 1** |  |
| 2 | | 9 | AcD3 | | VL | VLSELAAR | Partial-Ac- | 2.99 | Q8TB03 | CX038_HUMAN | **Uncharacterized protein CXorf38** |  |
| 2 | | 11 | AcD3 | | VM | VMAEGTAVLR | Partial-Ac- | 11.83 | Q9Y3A3 | MOBL3_HUMAN | **Mps one binder kinase activator-like 3** |  |
| 2 | | 13 | AcD3 | | VM | VMEKPSPLLVGR | Partial-Ac- | 11.01 | Q13283 | G3BP1_HUMAN | **Ras GTPase-activating protein-binding protein 1** | Q9UN86 (2-13) |
| 2 | | 14 | AcD3 | | VM | VMEVGTLDAGGLR | N.D | N.D | P28562 | DUS1_HUMAN | **Dual specificity protein phosphatase 1** |  |
| 2 | | 10 | AcD3 | | VN | VNFTVDQIR | 100% free | 0.00 | P13639 | EF2_HUMAN | **Elongation factor 2** |  |
| 2 | | 9 | AcD3 | | VN | VNLLQIVR | 100% Ac- | 100.00 | O75438 | NDUB1_HUMAN | **ADH dehydrogenase [ubiquinone] 1 beta subcomplex subunit 1** |  |
| 2 | | 19 | AcD3 | | VN | VNPTVFFDIAVDGEPLGR | Partial-Ac- | 27.76 | P62937 | PPIA_HUMAN | **Peptidyl-prolyl cis-trans isomerase A** |  |
| 2 | | 8 | AcD3 | | VN | VNVPKTR | 100% free | 0.00 | P83881 | RL36A_HUMAN | **60S ribosomal protein L36a** | Q969Q0 (2-8) |
| 2 | | 10 | AcD3 | | VQ | VQIVISSAR | 100% free | 0.00 | Q8WV66 | CTF8_HUMAN | **Chromosome transmission fidelity protein 8 homolog** |  |
| 2 | | 13 | AcD3 | | VQ | VQKESQATLEER | 100% free | 0.00 | O60502 | NCOAT_HUMAN | **Bifunctional protein COAT** |  |
| 2 | | 24 | AcD3 | | VT | VTEQEVDAIGQTLVDPKQPLQAR | Partial-Ac- | 9.46 | Q9BU89 | DOHH_HUMAN | **Deoxyhypusine hydroxylase** |  |
| 2 | | 16 | AcD3 | | VV | VVPSLKLQDLIEEIR | N.D | N.D | O75843 | AP1G2_HUMAN | **AP-1 complex subunit gamma-like 2** |  |
| 2 | | 13 | AcD3 | | VV | VVSKMNKDAQMR | 100% free | 0.00 | Q9NPA8 | ENY2_HUMAN | **Enhancer of yellow 2 transcription factor homolog** |  |
| 2 | | 13 | AcD3 | | VY | VYISNGQVLDSR | N.D | N.D | Q9Y6D0 | SELK_HUMAN | **Selenoprotein K** |  |
| 2 | | 17 | AcD3 | | VA | VAAKKTKKSLESINSR | 100% free | 0.00 | P62888 | RL30_HUMAN | **60S ribosomal protein L30** |  |
| **25. Ile** | |  |  | |  |  |  |  |  |  |  |  |
| 1 | | 7 | AcD3 | | II | IIEPSLR | 100% free | 0.00 | P62987 | RL40_HUMAN | **60S ribosomal protein L40** |  |
