# Supplementary material for: NatF Contributes to an Evolutionary Shift in Protein N-Terminal Acetylation and Is Important for Normal Chromosome Segregation
Source: PLoS Genet. 2011 Jul 7;7(7):e1002169. doi: 10.1371/journal.pgen.1002169 (PMC3131286; doi:10.1371/journal.pgen.1002169)
Supplement: Table S3 — Relating the occurrence of N-Ac and different N-termini in yeast and humans. An unbiased estimation of N-Ac for all methionine-starting yeast (6613) and human SwissProt entries (20102) (SwissProt version 57.8) was performed based on the nature of the N-terminal amino acids and the N-terminal acetylation status uncovered in this study. (DOC) [file pgen.1002169.s005.doc]

| **Table S3. Relating the occurrence of N-Ac and different N-termini in yeast and humans.** An unbiased estimation of N-Ac for all methionine-starting yeast (6613) and human SwissProt entries (20102) (SwissProt version 57.8) was performed based on the nature of the N-terminal amino acids and the N-terminal acetylation status uncovered in this study (Table 1). Whenever, for certain types of N-termini, no experimental evidence of N-Ac was obtained, the N-Ac of most related type of N-termini was used for the overall calculation (i.e. MW- N-termini were considered MF-type, while MR- and MH- N-termini were considered MK-type). ID stands for identification.  _______________________________________________________________________________________________________________________________ | | | | | | | | | | | | | | | | | | | | |
| --- | --- | --- | --- | --- | --- | --- | --- | --- | --- | --- | --- | --- | --- | --- | --- | --- | --- | --- | --- | --- |
|  |  |  |  |  |  |  |  |  |  |  |  |  |  |  |  |  |  |  |  |  |
| Swissprot yeast database | | | |  |  | Calculation based on yeast experimental dataset (%Ac) | | | |  | Calculation based on human experimental dataset (%Ac) | | | |  | theoretical yeast Swissprot occurence versus experimental occurence | | | | |
|  |  |  |  |  |  |  |  |  |  |  |  |  |  |  |  |  |  |  |  |  |
| **Nat** | **start with Met** | **AA 1** | # | **%of all IDs** |  | **Nat** | **start with Met** | **AA 1** |  | **%Ac calc.** | **Nat** | **start with Met** | **AA 1** |  | **%Ac calc.** |  |  |  | **theoretical % of IDs** | **experimental % of IDs** |
| **NatA** | 0 | A | 536 | 8.11% |  | **NatA** | 0 | A | 257.7 |  | **NatA** | 0 | A | 510.8 |  | **NatA** | 0 | A | 8.1% | 9.78% |
|  |  | C | 56 | 0.85% |  |  |  | C | 0.0 |  |  |  | C | 42.0 |  |  |  | C | 0.8% | 0.34% |
|  |  | G | 305 | 4.61% |  |  |  | G | 25.4 |  |  |  | G | 0.0 |  |  |  | G | 4.6% | 4.89% |
|  |  | S | 1504 | 22.74% |  |  |  | S | 1463.6 |  |  |  | S | 1473.9 |  |  |  | S | 22.7% | 34.74% |
|  |  | T | 503 | 7.61% |  |  |  | T | 279.4 |  |  |  | T | 451.2 |  |  |  | T | 7.6% | 7.08% |
|  |  | V | 404 | 6.11% |  |  |  | V | 39.1 |  |  |  | V | 78.0 |  |  |  | V | 6.1% | 7.59% |
|  | Total | | 3212 | 48.57% |  |  | Total | |  |  |  | Total | |  |  |  | Total | | 48.6% | 64.42% |
| **NatB** | 1 | D | 319 | 4.82% |  | **NatB** | 1 | D | 319.0 |  | **NatB** | 1 | D | 315.6 |  | **NatB** | 1 | D | 4.8% | 8.09% |
|  |  | E | 341 | 5.16% |  |  |  | E | 341.0 |  |  |  | E | 341.0 |  |  |  | E | 5.2% | 3.71% |
|  |  | N | 341 | 5.16% |  |  |  | N | 341.0 |  |  |  | N | 341.0 |  |  |  | N | 5.2% | 4.38% |
|  | Total | | 1001 | 15.14% |  |  | Total | |  |  |  | Total | |  |  |  | Total | | 15.1% | 16.19% |
| **NatC** | 1 | F | 231 | 3.49% |  | **NatC** | 1 | F | 138.6 |  | **NatC** | 1 | F | 192.5 |  | **NatC** | 1 | F | 3.5% | 1.18% |
|  |  | I | 229 | 3.46% |  |  |  | I | 76.3 |  |  |  | I | 114.5 |  |  |  | I | 3.5% | 2.19% |
|  |  | L | 436 | 6.59% |  |  |  | L | 145.3 |  |  |  | L | 327.0 |  |  |  | L | 6.6% | 3.54% |
|  |  | W | 43 | 0.65% |  |  |  | W | 25.8 |  |  |  | W | 35.8 |  |  |  | W | 0.7% | 0.00% |
|  | Total | | 939 | 14.20% |  |  | Total | |  |  |  | Total | |  |  |  | Total | | 14.2% | 6.91% |
| **Other** | 1 | H | 92 | 1.39% |  | **Other** | 1 | H | 12.0 |  | **Other** | 1 | H | 44.0 |  | **Other** | 1 | H | 1.4% | 2.02% |
|  |  | K | 394 | 5.96% |  |  |  | K | 51.4 |  |  |  | K | 188.5 |  |  |  | K | 6.0% | 4.72% |
|  |  | M | 108 | 1.63% |  |  |  | M | 90.0 |  |  |  | M | 108.0 |  |  |  | M | 1.6% | 0.84% |
|  |  | Q | 176 | 2.66% |  |  |  | Q | 148.9 |  |  |  | Q | 165.0 |  |  |  | Q | 2.7% | 2.02% |
|  |  | R | 202 | 3.05% |  |  |  | R | 26.3 |  |  |  | R | 96.6 |  |  |  | R | 3.1% |  |
|  |  | Y | 104 | 1.57% |  |  |  | Y | 65.0 |  |  |  | Y | 104.0 |  |  |  | Y | 1.6% | 0.51% |
|  | Total | | 1076 | 16.27% |  |  | Total | |  |  |  | Total | |  |  |  | Total | | 16.3% | 10.12% |
| **Free** | 0 | P | 289 | 4.37% |  | **Free** | 0 | P | 0.0 |  | **Free** | 0 | P | 0.0 |  | **Free** | 0 | P | 4.4% | 2.36% |
| **Grand Total** | | | **6613** | 100.00% |  | **Grand Total** | | | **3845.9** | **58.2%** | **Grand Total** | | | **4929.3** | **74.5%** | **Grand Total** | | | **100.0%** | 100.00% |
|  |  |  |  |  |  |  |  |  |  |  |  |  |  |  |  |  |  |  |  |  |
| Swissprot human database | | | |  |  | Calculation based on yeast experimental dataset (%Ac) | | | |  | Calculation based on human experimental dataset (%Ac) | | | |  | theoretical human Swissprot occurence versus experimental occurence | | | | |
|  |  |  |  |  |  |  |  |  |  |  |  |  |  |  |  |  |  |  |  |  |
| **Nat** | **start with Met** | **AA 1** | # | **% of all IDs** |  | **Nat** | **start with Met** | **AA 1** |  | **%Ac calc.** | **Nat** | **start with Met** | **AA 1** |  | **%Ac calc.** |  |  |  | **theoretical % of IDs** | **experimental % of IDs** |
| **NatA** | 0 | A | 4659 | 23.18% |  | **NatA** | 0 | A | 2239.9 |  | **NatA** | 0 | A | 4440.0 |  | **NatA** | 0 | A | 23.18% | 34.41% |
|  |  | C | 175 | 0.87% |  |  |  | C | 0.0 |  |  |  | C | 131.3 |  |  |  | C | 0.87% | 0.38% |
|  |  | G | 1588 | 7.90% |  |  |  | G | 132.3 |  |  |  | G | 0.0 |  |  |  | G | 7.90% | 1.46% |
|  |  | S | 2293 | 11.41% |  |  |  | S | 2231.4 |  |  |  | S | 2247.1 |  |  |  | S | 11.41% | 12.16% |
|  |  | T | 874 | 4.35% |  |  |  | T | 485.6 |  |  |  | T | 784.0 |  |  |  | T | 4.35% | 3.08% |
|  |  | V | 781 | 3.89% |  |  |  | V | 75.6 |  |  |  | V | 150.7 |  |  |  | V | 3.89% | 2.31% |
|  | Total | | 10370 | 51.59% |  |  | Total | |  |  |  | Total | |  |  |  | Total | | 51.59% | 53.81% |
| **NatB** | 1 | D | 1136 | 5.65% |  | **NatB** | 1 | D | 1136.0 |  | **NatB** | 1 | D | 1123.7 |  | **NatB** | 1 | D | 5.65% | 7.93% |
|  |  | E | 1942 | 9.66% |  |  |  | E | 1942.0 |  |  |  | E | 1942.0 |  |  |  | E | 9.66% | 14.47% |
|  |  | N | 646 | 3.21% |  |  |  | N | 646.0 |  |  |  | N | 646.0 |  |  |  | N | 3.21% | 2.69% |
|  | Total | | 3724 | 18.53% |  |  | Total | |  |  |  | Total | |  |  |  | Total | | 18.53% | 25.10% |
| **NatC** | 1 | F | 371 | 1.85% |  | **NatC** | 1 | F | 222.6 |  | **NatC** | 1 | F | 309.2 |  | **NatC** | 1 | F | 1.85% | 1.54% |
|  |  | I | 292 | 1.45% |  |  |  | I | 97.3 |  |  |  | I | 146.0 |  |  |  | I | 1.45% | 0.92% |
|  |  | L | 1067 | 5.31% |  |  |  | L | 355.7 |  |  |  | L | 800.3 |  |  |  | L | 5.31% | 2.62% |
|  |  | W | 272 | 1.35% |  |  |  | W | 163.2 |  |  |  | W | 226.7 |  |  |  | W | 1.35% |  |
|  | Total | | 2002 | 9.96% |  |  | Total | |  |  |  | Total | |  |  |  | Total | | 9.96% | 5.08% |
| **Other** | 1 | H | 206 | 1.02% |  | **Other** | 1 | H | 26.9 |  | **Other** | 1 | H | 98.5 |  | **Other** | 1 | H | 1.02% |  |
|  |  | K | 855 | 4.25% |  |  |  | K | 111.5 |  |  |  | K | 408.9 |  |  |  | K | 4.25% | 0.08% |
|  |  | M | 339 | 1.69% |  |  |  | M | 282.5 |  |  |  | M | 339.0 |  |  |  | M | 1.69% | 1.00% |
|  |  | Q | 464 | 2.31% |  |  |  | Q | 392.6 |  |  |  | Q | 435.0 |  |  |  | Q | 2.31% | 1.15% |
|  |  | R | 897 | 4.46% |  |  |  | R | 117.0 |  |  |  | R | 429.0 |  |  |  | R | 4.46% |  |
|  |  | Y | 163 | 0.81% |  |  |  | Y | 101.9 |  |  |  | Y | 163.0 |  |  |  | Y | 0.81% | 0.69% |
|  | Total | | 2924 | 14.55% |  |  | Total | |  |  |  | Total | |  |  |  | Total | | 14.55% | 2.93% |
| **Free** | 1 | P | 1082 | 5.38% |  | **Free** | 0 | P | 0.0 |  | **Free** | 0 | P | 0.0 |  | **Free** | 1 | P | 5.38% | 4.39% |
| **Grand Total** | | | **20102** | 100.00% |  | **Grand Total** | | | **10759.9** | **53.5%** | **Grand Total** | | | **14820.4** | **73.7%** | **Grand Total** | | | **100.0%** | **100.00%** |
