# Supplementary material for: NatF Contributes to an Evolutionary Shift in Protein N-Terminal Acetylation and Is Important for Normal Chromosome Segregation
Source: PLoS Genet. 2011 Jul 7;7(7):e1002169. doi: 10.1371/journal.pgen.1002169 (PMC3131286; doi:10.1371/journal.pgen.1002169)
Supplement: Table S4 — List of the 72 unique in vivo hNaa60p substrate N-termini identified in yeast. S4A. hNaa60p yeast substrate N-termini (44) which were completely unacetylated in the control setup analyzed. S4B. hNaa60p yeast substrate N-termini (28) which were partially N-Ac in the control setup analyzed. (DOC) [file pgen.1002169.s006.doc]

| **Table S4A/B. hNaa60p in vivo N-Ac substrates identified in yeast.** List of the 72 unique human NatF substrate N-termini identified. **S4A.** hNaa60p yeast substrate N-termini (44) which were completely unacetylated in the control setup analyzed. **S4B.** hNaa60p yeast substrate N-termini (28) which were partially N-Ac in the control setup analyzed. Start and end positions, N-term modification status/states confirmed by MS/MS, corresponding peptide sequence identified, global N-term modification status in control yeast, %Ac of the N-terminus in the control yeast strain, %Ac of the N-terminus in the yeast strain expressing hNaa60p, difference in %Ac, UniProt database primary accession number, Uniprot name, sample(s) in which the N-terminus was/were identified, protein description, number of identified spectra (count), highest Mascot ion score, minimum Mascot identity-threshold and, whenever a peptide matched to multiple members of a protein family (redundancy), isoforms are given for all uniquely identified yeast N-termini. N-termini are ranked according to their difference in N-Ac when comparing the control versus the hNaa60p-expressing setup.  ______________________________________________________________________________________________________________________________________________________________________________________ | | | | | | | | | | | | | | | | |
| --- | --- | --- | --- | --- | --- | --- | --- | --- | --- | --- | --- | --- | --- | --- | --- | --- |
|
|  |  |  |  |  |  |  |  |  |  |  |  |  |  |  |  |  |
| **start** | **end** | **N-term modifications** | **AA 1-2** | **sequence** | **N-term modification status control** | **avg. %Ac control** | **avg %Ac. hNaa60p yeast** | **∆ Ac (hNaa60p - control)** | **accession** | **Uniprot** | **Description** | **isoforms** | **Count (*)** | **max(MACOT score)** | **delta(MASCOT score)** | **identitythreshold** |
| **A.) hNaa60p substrates (unacetylated N-termini in control yeast) (#44)** | | | | | |  |  |  |  |  |  |  |  |  |  |  |
| 1 | 10 | Ace,AcD3C13 | **MV** | MVNELENVPR | 100% free | 0% | 93% | 93% | P39010 | AKR1_YEAST | **Palmitoyltransferase AKR1** |  | 3 | 72 | 36 | 36 |
| 1 | 15 | Ace,AcD3C13 | **MF** | MFGLPQQEVSEEEKR | 100% free | 0% | 88% | 88% | P80967 | TOM5_YEAST | **Mitochondrial import receptor subunit TOM5** |  | 11 | 88 | 53 | 35 |
| 1 | 12 | Ace,AcD3C13 | **MY** | MYFDKDNSMSPR | 100% free | 0% | 88% | 88% | Q12236 | KOK0_YEAST | **Probable serine/threonine-protein kinase YOL100W** |  | 3 | 83 | 54 | 29 |
| 1 | 19 | Ace,AcD3C13 | **MV** | MVPAESNAVQAKLAKTLQR | 100% free | 0% | 85% | 85% | Q12125 | YO164_YEAST | **UPF0363 protein YOR164C** |  | 2 | 78 | 42 | 36 |
| 1 | 9 | Ace,AcD3C13 | **MS** | MSEEKTYKR | 100% free | 0% | 85% | 85% | P33775 | PMT1_YEAST | **Dolichyl-phosphate-mannose--protein mannosyltransferase 1** |  | 3 | 66 | 29 | 37 |
| 1 | 16 | AcD3C13,Ace | **MK** | MKGTGGVVVGTQNPVR | 100% free | 0% | 82% | 82% | Q08954 | YP199_YEAST | **Smr domain-containing protein YPL199C** |  | 11 | 106 | 69 | 37 |
| 1 | 11 | Ace,AcD3C13 | **MI** | MIALPVEKAPR | 100% free | 0% | 78% | 78% | P27614 | CBPS_YEAST | **Carboxypeptidase S** |  | 2 | 94 | 58 | 36 |
| 1 | 17 | Ace,AcD3C13 | **ML** | MLEEGNNVYEIQDLEKR | 100% free | 0% | 78% | 78% | P17064 | FCY2_YEAST | **Purine-cytosine permease FCY2** |  | 6 | 115 | 79 | 36 |
| 1 | 9 | Ace,AcD3C13 | **MI** | MINESVSKR | 100% free | 0% | 70% | 70% | P47050 | CUL8_YEAST | **Cullin-8** |  | 4 | 73 | 36 | 37 |
| 1 | 9 | AcD3C13,Ace | **MK** | MKPPLNMSR | 100% free | 0% | 57% | 57% | Q12407 | YD199_YEAST | **Putative metabolite transport protein YDL199C** |  | 2 | 41 | 4 | 37 |
| 1 | 12 | Ace,AcD3C13 | **MY** | MYVDPMNNNEIR | 100% free | 0% | 56% | 56% | P05986 | KAPC_YEAST | **cAMP-dependent protein kinase type 3** |  | 5 | 61 | 32 | 29 |
| 1 | 17 | Ace,AcD3C13 | **MV** | MVLVQDLLHPTAASEAR | 100% free | 0% | 51% | 51% | P35997 | RS27A_YEAST | **40S ribosomal protein S27-A** | P38711 (1-17) | 9 | 120 | 83 | 37 |
| 1 | 8 | Ace,AcD3C13 | **MT** | MTPEAKKR | 100% free | 0% | 50% | 50% | P48415 | SEC16_YEAST | **COPII coat assembly protein SEC16** |  | 14 | 60 | 24 | 36 |
| 1 | 7 | Ace,AcD3C13 | **ML** | MLFNINR | 100% free | 0% | 48% | 48% | P53278 | YG3A_YEAST | **Uncharacterized protein YGR130C** |  | 3 | 59 | 23 | 36 |
| 1 | 11 | AcD3C13 | **MI** | MIFSLDEELHR | 100% free | 0% | 47% | 47% | P38070 | KBN8_YEAST | **Probable serine/threonine-protein kinase YBR028C** |  | 2 | 63 | 26 | 37 |
| 1 | 10 | Ace,AcD3C13 | **MM** | MMSDLTPIFR | 100% free | 0% | 46% | 46% | P41834 | UFE1_YEAST | **Syntaxin UFE1** |  | 2 | 55 | 20 | 35 |
| 1 | 15 | Ace,AcD3C13 | **MF** | MFESVNLDENSPEDR | 100% free | 0% | 45% | 45% | P40971 | LYS14_YEAST | **Lysine biosynthesis regulatory protein LYS14** |  | 3 | 84 | 55 | 29 |
| 1 | 13 | AcD3C13,Ace | **MY** | MYFTDESSPAMNR | 100% free | 0% | 43% | 43% | P52960 | PIP2_YEAST | **Peroxisome proliferation transcriptional regulator** |  | 3 | 70 | 44 | 26 |
| 1 | 10 | AcD3C13,Ace | **MI** | MIVDYEKDPR | 100% free | 0% | 36% | 36% | Q12347 | HRT3_YEAST | **F-box protein HRT3** |  | 3 | 59 | 23 | 36 |
| 1 | 9 | AcD3C13,Ace | **MK** | MKVDLPESR | 100% free | 0% | 35% | 35% | P47048 | YJE9_YEAST | **Uncharacterized protein YJL049W** |  | 3 | 52 | 15 | 37 |
| 1 | 15 | Ace,AcD3C13 | **MV** | MVTSNVVLVSGEGER | 100% free | 0% | 34% | 34% | P52286 | SKP1_YEAST | **Suppressor of kinetochore protein 1** |  | 7 | 112 | 75 | 37 |
| 1 | 19 | AcD3C13 | **MK** | MKAIDKMTDNPPQEGLSGR | 100% free | 0% | 32% | 32% | P27882 | ERV1_YEAST | **Mitochondrial FAD-linked sulfhydryl oxidase ERV1** |  | 9 | 73 | 36 | 37 |
| 1 | 10 | AcD3C13 | **MV** | MVTVGVFSER | 100% free | 0% | 30% | 30% | A6ZT71 | SOL3_YEAS7 | **6-phosphogluconolactonase 3** | B3LSS7 (1-10)^AB5VK90 (1-10)^AP38858 (1-10) | 2 | 70 | 33 | 37 |
| 1 | 7 | AcD3C13 | **MF** | MFSFVQR | 100% free | 0% | 30% | 30% | Q12133 | SPC3_YEAST | **Signal peptidase complex subunit SPC3** |  | 2 | 55 | 19 | 36 |
| 1 | 11 | AcD3C13,Ace | **MT** | MTEDKSQVKIR | 100% free | 0% | 28% | 28% | Q12024 | YTM1_YEAST | **Microtubule-associated protein YTM1** |  | 3 | 54 | 17 | 37 |
| 1 | 8 | AcD3C13,Ace | **ML** | MLKSLKSR | 100% free | 0% | 24% | 24% | P40549 | MNT3_YEAST | **Alpha-1,3-mannosyltransferase MNT3** |  | 3 | 55 | 22 | 33 |
| 1 | 9 | AcD3C13,Ace | **MK** | MKKTFEQFR | 100% free | 0% | 24% | 24% | P25576 | YCE7_YEAST | **UPF0647 protein YCL047C** |  | 3 | 63 | 26 | 37 |
| 1 | 10 | AcD3C13,Ace | **MQ** | MQLVPLELNR | 100% free | 0% | 21% | 21% | Q12010 | YO092_YEAST | **Uncharacterized membrane protein YOL092W** |  | 3 | 57 | 20 | 37 |
| 1 | 11 | AcD3C13,Ace | **MK** | MKDLQKKSSVR | 100% free | 0% | 18% | 18% | Q02831 | YP077_YEAST | **Uncharacterized protein YPL077C** |  | 4 | 70 | 35 | 35 |
| 1 | 13 | AcD3C13 | **MK** | MKSSIPITEVLPR | 100% free | 0% | 16% | 16% | Q3E841 | YN034_YEAST | **Uncharacterized protein YNR034W-A** |  | 27 | 88 | 52 | 36 |
| 1 | 8 | AcD3C13,Ace | **ML** | MLMPKQER | 100% free | 0% | 15% | 15% | P46784 | RS10B_YEAST | **40S ribosomal protein S10-B** |  | 58 | 63 | 27 | 36 |
| 1 | 9 | Ace,AcD3C13 | **MK** | MKVQITNSR | 100% free | 0% | 15% | 15% | P13574 | STE12_YEAST | **Protein STE12** |  | 3 | 71 | 34 | 37 |
| 1 | 7 | Ace,AcD3C13 | **ML** | MLKDLVR | 100% free | 0% | 15% | 15% | P47133 | YJ58_YEAST | **Uncharacterized protein YJR088C** |  | 5 | 58 | 23 | 35 |
| 1 | 6 | AcD3C13 | **ML** | MLETLR | 100% free | 0% | 13% | 13% | P34077 | NIC96_YEAST | **Nucleoporin NIC96** |  | 2 | 51 | 15 | 36 |
| 1 | 13 | AcD3C13 | **MS** | MSEVIEGNVKIDR | 100% free | 0% | 12% | 12% | P20081 | FKBP_YEAST | **FK506-binding protein 1** |  | 7 | 99 | 62 | 37 |
| 1 | 7 | Ace,AcD3C13 | **MK** | MKVLEER | 100% free | 0% | 12% | 12% | P47076 | RPC9_YEAST | **DNA-directed RNA polymerase III subunit RPC9** |  | 16 | 56 | 19 | 37 |
| 1 | 9 | AcD3C13 | **MK** | MKVGGIEDR | 100% free | 0% | 10% | 10% | Q06549 | CDD_YEAST | **Cytidine deaminase** |  | 4 | 68 | 31 | 37 |
| 1 | 12 | AcD3C13 | **MT** | MTIAPITGTIKR | 100% free | 0% | 9% | 9% | P07255 | COX9_YEAST | **Cytochrome c oxidase subunit 7A** |  | 2 | 59 | 25 | 34 |
| 1 | 15 | AcD3C13 | **MK** | MKTIIISDFDETITR | 100% free | 0% | 9% | 9% | P25616 | YCQ5_YEAST | **UPF0655 protein YCR015C** |  | 2 | 80 | 43 | 37 |
| 1 | 26 | AcD3C13 | **MK** | MKLPVAQYSAPDGVEKSFAPIRDDPR | 100% free | 0% | 7% | 7% | P46984 | GON7_YEAST | **Protein GON7** |  | 2 | 74 | 37 | 37 |
| 1 | 7 | AcD3C13 | **ML** | MLAEKTR | 100% free | 0% | 5% | 5% | P39676 | FHP_YEAST | **Flavohemoprotein** |  | 35 | 59 | 23 | 36 |
| 1 | 11 | AcD3C13 | **MS** | MSEEGPQVKIR | 100% free | 0% | 5% | 5% | P16370 | RPB3_YEAST | **DNA-directed RNA polymerase II subunit RPB3** |  | 2 | 52 | 15 | 37 |
| 2 | 31 | AcD3C13 | AG | AGAPAPPPPPPPPALGGSAPKPAKSVMQGR | 100% free | 0% | 6% | 6% | P37370 | VRP1_YEAST | **Verprolin** |  | 3 | 49 | 13 | 36 |
| 1 | 22 | AcD3C13 | **MK** | MKLPVAQYSAPDGVEKSFAPIR | 100% free | 0% | 8% | 8% | P46984 | GON7_YEAST | **Protein GON7** |  | 3 | 92 | 55 | 37 |
| **B.) hNaa60p substrates (partially N-acetylated N-termini in control yeast) (#28)** | | | | |  |  |  |  |  |  |  |  |  |  |  |  |
| 1 | 9 | Ace,AcD3C13 | **MQ** | MQGNKSTIR | partial Ac- | 18% | 96% | 77% | P01119 | RAS1_YEAST | **Ras-like protein 1** |  | 4 | 92 | 55 | 37 |
| 1 | 11 | Ace,AcD3C13 | **MS** | MSEQESDEVKR | partial Ac- | 11% | 85% | 73% | Q12191 | BUG1_YEAST | **Binder of USO1 and GRH1 protein 1** |  | 2 | 57 | 24 | 33 |
| 1 | 24 | Ace,AcD3C13 | **MM** | MMASTSNDEEKLISTTDKYFIEQR | partial Ac- | 24% | 92% | 68% | Q12248 | DAD1_YEAST | **DASH complex subunit DAD1** |  | 3 | 52 | 18 | 34 |
| 1 | 9 | Ace,AcD3C13 | **MS** | MSDEAKEKR | partial Ac- | 10% | 65% | 55% | A6ZQX9 | CHZ1_YEAS7 | **Histone H2A.Z-specific chaperone CHZ1** | P40019 (1-9) | 3 | 69 | 33 | 36 |
| 1 | 9 | Ace,AcD3C13 | **MV** | MVALISKKR | partial Ac- | 23% | 78% | 55% | P05750 | RS3_YEAST | **40S ribosomal protein S3** |  | 4 | 86 | 55 | 31 |
| 1 | 17 | AcD3C13,Ace | **MM** | MMAKNNKTTEAKMSKKR | partial Ac- | 32% | 88% | 55% | Q04660 | ERB1_YEAST | **Eukaryotic ribosome biogenesis protein 1** |  | 6 | 59 | 21 | 38 |
| 1 | 18 | Ace,AcD3C13 | **MK** | MKNLTTIKQTNKNVKQER | partial Ac- | 8% | 60% | 52% | Q08282 | LCMT2_YEAST | **Leucine carboxyl methyltransferase 2** |  | 2 | 69 | 34 | 35 |
| 1 | 9 | Ace,AcD3C13 | **MA** | MAVSKVYAR | partial Ac- | 2% | 50% | 48% | P00924 | ENO1_YEAST | **Enolase 1** | P00925 (1-9) | 3 | 68 | 31 | 37 |
| 1 | 11 | Ace,AcD3C13 | **MT** | MTKSEQQADSR | partial Ac- | 48% | 91% | 43% | P32582 | CBS_YEAST | **Cystathionine beta-synthase** |  | 4 | 67 | 33 | 34 |
| 1 | 11 | AcD3C13,Ace | **MV** | MVQSAVLGFPR | partial Ac- | 4% | 44% | 40% | P05694 | METE_YEAST | **5-methyltetrahydropteroyltriglutamate--homocysteine methyltransferase** |  | 3 | 80 | 43 | 37 |
| 1 | 12 | AcD3C13,Ace | **MV** | MVGQQYSSAPLR | partial Ac- | 23% | 62% | 39% | P04050 | RPB1_YEAST | **DNA-directed RNA polymerase II subunit RPB1** |  | 2 | 66 | 30 | 36 |
| 1 | 10 | AcD3C13,Ace | **ML** | MLLTPAKTTR | partial Ac- | 45% | 81% | 37% | P53222 | YG1O_YEAST | **Uncharacterized protein YGR035C** |  | 3 | 54 | 18 | 36 |
| 1 | 13 | Ace,AcD3C13 | **MT** | MTPEQKAKLEANR | partial Ac- | 11% | 44% | 33% | P28519 | RAD14_YEAST | **DNA repair protein RAD14** |  | 6 | 66 | 29 | 37 |
| 1 | 15 | Ace,AcD3C13 | **MY** | MYTNYSLTSSDAMPR | partial Ac- | 53% | 85% | 32% | Q03764 | EKI1_YEAST | **Ethanolamine kinase** |  | 3 | 106 | 78 | 28 |
| 1 | 10 | Ace,AcD3C13 | **MS** | MSKITSSQVR | partial Ac- | 37% | 68% | 31% | P53030 | RL1_YEAST | **60S ribosomal protein L1** |  | 4 | 72 | 35 | 37 |
| 1 | 27 | AcD3C13 | **MS** | MSDEEHTFETADAGSSATYPMQCSALR | partial Ac- | 11% | 42% | 31% | P23301 | IF5A2_YEAST | **Eukaryotic translation initiation factor 5A-2** |  | 3 | 72 | 49 | 23 |
| 1 | 12 | Ace,AcD3C13 | **MM** | MMKKKPKCQIAR | partial Ac- | 42% | 69% | 27% | A6ZZE3 | LOT5_YEAS7 | **Protein LOT5** | P34234 (1-12) | 5 | 49 | 13 | 36 |
| 2 | 10 | AcD3C13,Ace | AG | AGATSSIIR | partial Ac- | 8% | 30% | 22% | P40474 | YIM1_YEAST | **Uncharacterized transporter YIL121W** |  | 3 | 72 | 37 | 35 |
| 1 | 9 | AcD3C13 | **MI** | MIASEIFER | partial Ac- | 14% | 33% | 20% | P32488 | MBR3_YEAST | **Protein MBR3** |  | 2 | 46 | 9 | 37 |
| 1 | 13 | Ace,AcD3C13 | **MQ** | MQSQDSCYGVAFR | partial Ac- | 51% | 71% | 19% | P16861 | K6PF1_YEAST | **6-phosphofructokinase subunit alpha** |  | 4 | 69 | 41 | 28 |
| 2 | 24 | Ace | SE | SESPMFAANGMPKVNQGAEEDVR | partial Ac- | 82% | 100% | 18% | P32449 | AROG_YEAST | **Phospho-2-dehydro-3-deoxyheptonate aldolase, tyrosine-inhibited** |  | 53 | 102 | 71 | 31 |
| 1 | 11 | AcD3C13,Ace | **MS** | MSTELTVQSER | partial Ac- | 22% | 38% | 16% | P26781 | RS11_YEAST | **40S ribosomal protein S11** |  | 3 | 74 | 39 | 35 |
| 1 | 9 | AcD3C13 | **MQ** | MQSSLPLCR | partial Ac- | 33% | 48% | 15% | Q02202 | ECM9_YEAST | **Protein ECM9** |  | 2 | 42 | 6 | 36 |
| 1 | 8 | AcD3C13,Ace | **MQ** | MQEGGFIR | partial Ac- | 30% | 44% | 14% | P31244 | RAD16_YEAST | **DNA repair protein RAD16** |  | 3 | 57 | 22 | 35 |
| 1 | 20 | Ace | **MQ** | MQNAQIKSSSKGSGIDGTDR | partial Ac- | 87% | 100% | 13% | P39734 | HPH2_YEAST | **Protein HPH2** |  | 3 | 55 | 18 | 37 |
| 1 | 14 | AcD3C13 | **MY** | MYIKAEQKPQQFER | partial Ac- | 20% | 33% | 13% | P40036 | GIP2_YEAST | **GLC7-interacting protein 2** |  | 2 | 60 | 22 | 38 |
| 1 | 17 | AcD3C13 | **MS** | MSQVYFDVEADGQPIGR | partial Ac- | 6% | 17% | 11% | P14832 | CYPH_YEAST | **Peptidyl-prolyl cis-trans isomerase** |  | 3 | 97 | 62 | 35 |
| 1 | 11 | Ace,AcD3C13 | **MG** | MGKGTPSFGKR | partial Ac- | 4% | 15% | 11% | P49166 | RL37A_YEAST | **60S ribosomal protein L37-A** | P51402 (1-11) | 3 | 55 | 18 | 37 |
