# Supplementary material for: NatF Contributes to an Evolutionary Shift in Protein N-Terminal Acetylation and Is Important for Normal Chromosome Segregation
Source: PLoS Genet. 2011 Jul 7;7(7):e1002169. doi: 10.1371/journal.pgen.1002169 (PMC3131286; doi:10.1371/journal.pgen.1002169)
Supplement: Table S5 — List of N-termini affected in their N-Ac status by knockdown or overexpression of hNaa60p in HeLa cells. (DOC) [file pgen.1002169.s007.doc]

| **Table S5.** **N-termini affected in their N-acetylation by knockdown or overexpression of hNaa60p.** % Ac in control samples, % Ac in knockdown or overexpression samples and Δ Ac, accession number, start and end position, sequence of the identified peptide and the protein description are indicated. A) Overexpression of hNaa60 in Hela cells increases the in vivo acetylation of 7 proteins. Only N-termini where the difference in acetylation was found to be more than 5% are listed. B) Knockdown of hNaa60 in Hela cells shifts (decreases) the in vivo acetylation of 10 proteins. Only N-termini where the difference in acetylation was found to be more than 5% are listed. | | | | | | | | |
| --- | --- | --- | --- | --- | --- | --- | --- | --- |
|  |  |  |  |  |  |  |  |  |
| **% Ac control** | **% Ac hNaa60 overexpression** | **Δ Ac** | **Accession** | **Start** | **End** |  | **Sequence** | **Description** |
|  |  |  |  |  |  |  |  |  |
| 55.59 | 65.16 | 9.57 | O00743 | 1 | 14 |  | MAPLDLDKYVEIAR | **Serine/threonine-protein phosphatase 6 catalytic subunit** |
| 18.23 | 32.16 | 13.93 | Q9UNE7 | 1 | 12 |  | MKGKEEKEGGAR | **STIP1 homology and U box-containing protein 1** |
| 13.17 | 25.13 | 11.96 | Q9BV29 | 1 | 13 |  | MKMFESADSTATR | **Uncharacterized protein C15orf57** |
| 75.80 | 85.04 | 9.24 | Q53GA4 | 1 | 9 |  | MKSPDEVLR | **Pleckstrin homology-like domain family A member 2** |
| 79.86 | 92.33 | 12.47 | Q9NZJ9 | 1 | 10 |  | MMKFKPNQTR | **Diphosphoinositol polyphosphate phosphohydrolase 2** |
| 20.67 | 26.67 | 6 | Q13572 | 1 | 9 |  | MQTFLKGKR | **Inositol-tetrakisphosphate 1-kinase** |
| 32.20 | 41.09 | 8.89 | P13639 | 1 | 10 |  | MVNFTVDQIR | **Elongation factor 2** |
|  |  |  |  |  |  |  |  |  |
|  |  |  |  |  |  |  |  |  |
| **% Ac control** | **% Ac hNaa60 knockdown** | **Δ Ac** | **Accession** | **Start** | **End** |  | **Sequence** | **Description** |
| 71.23 | 65.98 | 5.25 | Q9Y3D0 | 1 | 22 |  | MVGGGGVGGGLLENANPLIYQR | **UPF0195 protein FAM96B** |
| 86.83 | 81.23 | 5.60 | Q9H1Y0 | 2 | 9 |  | TDDKDVLR | **Autophagy protein 5** |
| 93.97 | 86.18 | 7.79 | Q9NXR7 | 1 | 9 |  | MSPEVALNR | **Protein BRE** |
| 77.21 | 70.41 | 6.80 | O00743 | 1 | 14 |  | MAPLDLDKYVEIAR | **Serine/threonine-protein phosphatase 6 catalytic subunit** |
| 74.17 | 67.41 | 6.76 | Q96S19 | 1 | 9 |  | MLVAAAAER | **UPF0585 protein C16orf13** |
| 62.03 | 55.13 | 6.90 | Q8WVM8 | 2 | 16 |  | AAAAAATAAAAASIR | **Sec1 family domain-containing protein 1** |
| 26.16 | 16.62 | 9.54 | Q71SY5 | 1 | 10 |  | MVPGSEGPAR | **Mediator of RNA polymerase II transcription subunit 25** |
| 73.88 | 64.89 | 8.99 | O95620 | 1 | 14 |  | MKSDCMQTTICQER | **tRNA-dihydrouridine synthase 4-like** |
| 30.09 | 23.21 | 6.88 | Q8TB03 | 1 | 9 |  | MVLSELAAR | **Uncharacterized protein CXorf38** |
|  |  |  |  |  |  |  |  |  |
